# A moderate increase in ambient temperature modulates the Atlantic cod (*Gadus morhua*) spleen transcriptome response to intraperitoneal viral mimic injection

**DOI:** 10.1186/1471-2164-13-431

**Published:** 2012-08-28

**Authors:** Tiago S Hori, A Kurt Gamperl, Marije Booman, Gordon W Nash, Matthew L Rise

**Affiliations:** 1 Ocean Sciences Centre, Memorial University of Newfoundland, St. John's, A1C 5S7, NL, Canada

## Abstract

**Background:**

Atlantic cod (*Gadus morhua*) reared in sea-cages can experience large variations in temperature, and these have been shown to affect their immune function. We used the new 20K Atlantic cod microarray to investigate how a water temperature change which, simulates that seen in Newfoundland during the spring-summer (i.e. from 10°C to 16°C, 1°C increase every 5 days) impacted the cod spleen transcriptome response to the intraperitoneal injection of a viral mimic (polyriboinosinic polyribocytidylic acid, pIC).

**Results:**

The temperature regime alone did not cause any significant increases in plasma cortisol levels and only minor changes in spleen gene transcription. However, it had a considerable impact on the fish spleen transcriptome response to pIC [290 and 339 significantly differentially expressed genes between 16°C and 10°C at 6 and 24 hours post-injection (HPI), respectively]. Seventeen microarray-identified transcripts were selected for QPCR validation based on immune-relevant functional annotations. Fifteen of these transcripts (i.e. 88%), including DHX58, STAT1, IRF7, ISG15, RSAD2 and IκBα, were shown by QPCR to be significantly induced by pIC.

**Conclusions:**

The temperature increase appeared to accelerate the spleen immune transcriptome response to pIC. We found 41 and 999 genes differentially expressed between fish injected with PBS vs. pIC at 10°C and sampled at 6HPI and 24HPI, respectively. In contrast, there were 656 and 246 genes differentially expressed between fish injected with PBS vs. pIC at 16°C and sampled at 6HPI and 24HPI, respectively. Our results indicate that the modulation of mRNA expression of genes belonging to the NF-κB and type I interferon signal transduction pathways may play a role in controlling temperature-induced changes in the spleen’s transcript expression response to pIC. Moreover, interferon effector genes such as ISG15 and RSAD2 were differentially expressed between fish injected with pIC at 10°C vs. 16°C at 6HPI. These results substantially increase our understanding of the genes and molecular pathways involved in the negative impacts of elevated ambient temperature on fish health, and may also be valuable to our understanding of how accelerated global climate change could impact cold-water marine finfish species.

## Background

The Atlantic cod (*Gadus morhua*) is an important commercial species in several countries including Canada, USA and Norway, whose supply has been threatened by declining wild stocks [1-3]. In recent years, the aquaculture of cod has emerged as a potential alternative source of fish for these markets [4,5]. Unfortunately, the development of Atlantic cod aquaculture still faces many challenges, including our incomplete understanding of how changes in the environment (e.g. seawater temperature) impact gadoid culture [2,6,7].

Atlantic cod reared in sea-cages are confined within a limited space, and therefore, are more likely than wild cod to be subjected to seasonal fluctuations in temperature. For example, high levels of mortality in farmed cod have been observed when temperatures increase during the summer months [8,9]. The temperatures to which farmed cod are often exposed in the summer months (e.g. 16°C to 20°C [8]) in themselves, however, are unlikely to be lethal for this species. In fact, previous work from our research group indicates that Atlantic cod juveniles can survive an incremental temperature increase of 1°C every 5 days until temperatures reach ~ 22°C (Gamperl et al., unpublished data).

Elevated temperatures have been shown to modulate the immune response of several commercially important fish species such as the rainbow trout (*Oncorhynchus mykiss*) [10-13], sea bass (*Dicentrarchus labrax*) [14], orange-spotted grouper (*Epinephelus coioides*) [15], Atlantic salmon (*Salmo salar*) [16] and Atlantic cod [9,17]. However, the influence of temperature on fish immune function is variable. For example, while constant elevated temperatures can enhance the immune response of salmonids (e.g. improve the protection conferred by vaccines [12]), variable temperatures (i.e. daily fluctuations in temperature) can be immune suppressive in sea bass [14]. Since the thermal regime to which cod are exposed during the spring-summer months in Newfoundland is characterized by a gradual increase in temperature (i.e. variable temperature) and temperatures do not tend to reach the cod’s critical thermal maximum (CTM) [9], we hypothesized that a temperature-dependent modulation of the immune system, which could lead to increased susceptibility to pathogens, may be associated with the losses sometimes observed in cod sea-cages.

**Figure 1:**
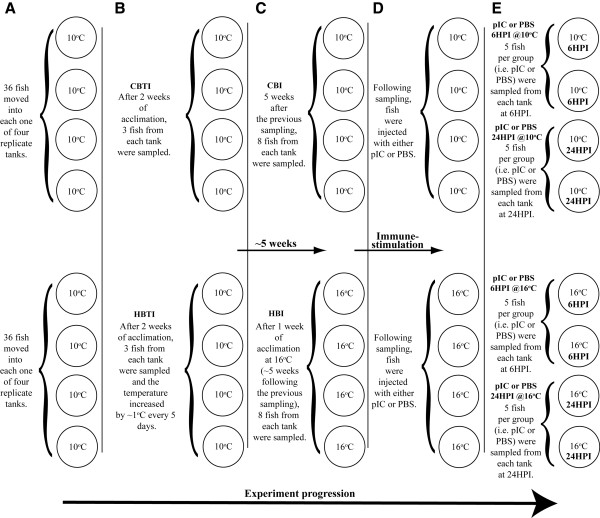
**Overview of the experimental design used to expose fish to changes in temperature simulating those observed in Newfoundland sea-cages in the spring-summer, and to investigate the impact of this temperature change on the Atlantic cod’s anti-viral response to the viral mimic (pIC).** CBTI = Control group with no temperature increase; HBTI = Heat-exposed before temperature increase.

**Figure 2:**
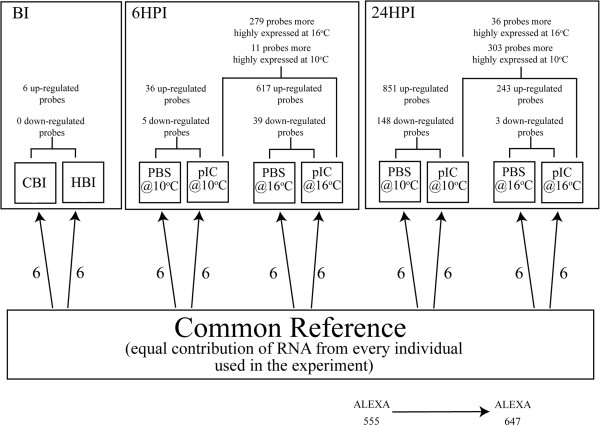
**Oveview of the microarray experimental design and results.** Each bracket connecting 2 boxes represents a direct comparison between these groups using SAM. All genes reported were found to have significant differential expression at FDR = 1%.

Microarrays have been widely used to study the immune-relevant gene expression responses of fish and fish cell lines to pathogens and pathogen-associated molecular patterns (PAMPs) (e.g. Rise et al. [18]; Milev-Milovanovic et al. [19]; Workenhe et al. [20]; Booman et al. [21]). In this study we used the 20,000 element (20K) oligonucleotide microarray platform (GEO accession # GPL10532 [21]), which includes sequences from both suppression subtractive hybridization (SSH) and normalized libraries enriched for immune and heat-stress responsive transcripts [22-26] (as well as sequences from several other normalized libraries), and reverse transcription – quantitative polymerase chain reaction (QPCR), to investigate the effects of an increasing temperature regime (gradually from 10°C to 16°C – Figures 1 and 2) on the Atlantic cod spleen response to the intra-peritoneal (IP) injection of the viral mimic polyriboinosinic polyribocytidylic acid (pIC). A better understanding of how moderately increased ambient temperature affects the genes and pathways involved in the cod’s anti-viral response may shed light on the potential mechanisms of temperature-induced immune suppression [14,17,27-29]. This knowledge will not only be important for the emerging Atlantic cod aquaculture industry, but may also be valuable to our understanding of how accelerated global climate change [30] may impact cold-water marine finfish species.

## Results

### Plasma cortisol

Plasma cortisol averaged (± SE) 15.3 ± 6.4 and 18.2 ± 6.3 ng ml^-1^ in the “control before temperature increase” (CBTI – fish that remained at 10°C throughout the experiment) and “heat-exposed before temperature increase” (HBTI - fish that remained at 10°C up to this sampling point and were subsequently exposed to a gradual increase in temperature) groups, respectively. Cortisol values were approximately two-fold higher ~ 5 weeks later (i.e. one week after temperature had reached 16°C in the heat-exposed tanks) in both sets of fish (Figure 1C) with the “control before injection” (CBI) group showing average cortisol levels of 33.1 ± 6.8 ng ml^-1^ and the “heat-exposed before injection” (HBI) group showing average cortisol levels of 37.7 ± 7.5 ng ml^-1^. However, there were no significant differences in cortisol levels between the groups at either time point, or within the groups between time points.

### Impact of non-lethal chronic temperature elevation on the spleen transcriptome

The first analysis carried out using significance analysis of microarrays (SAM) assessed the impact of the temperature increase alone on the cod spleen transcriptome. This analysis was carried out to investigate whether changes in the spleen transcriptome caused by temperature alone could be indicative of immune suppression. We compared 6 individuals from the CBI group to 6 individuals from the HBI group using a modified *t*-test assuming unequal variances in the SAM algorithm [31] as implemented in the Bioconductor package siggenes. The gradual 6°C increase in temperature had a very minor impact on spleen mRNA expression. Only 6 probes (all up-regulated) were identified as differentially expressed (at FDR = 1%) between the CBI and the HBI groups (Figure 2 and Additional file 1: Table S1).

### Impact of pIC injection on the spleen transcriptome

Polyriboinosinic polyribocytidylic acid (pIC) injected fish were also compared to their time- and temperature-matched phosphate buffered saline (PBS) injected controls using SAM. This gave us the following four comparisons: pIC 6HPI vs. PBS 6HPI (at 10 and 16°C) and pIC 24HPI vs. PBS 24HPI (at 10°C and 16°C). We identified 41 (36 up-regulated and 5 down-regulated) differentially expressed probes (at FDR = 1%) between the pIC 6HPI vs. PBS 6HPI at 10°C (Figure 2; Table 1; Additional file 1: Table S2), 656 probes (617 up-regulated and 39 down-regulated) between the pIC 6HPI vs. PBS 6HPI at 16°C (Figure 2; Table 1; Additional file 1: Table S3), 999 probes (851 up-regulated and 148 down-regulated) between pIC 24HPI vs. PBS 24HPI at 10°C (Figure 2; Table 2; Additional file 1: Table S4), and 246 probes (243 up-regulated and 3 down-regulated) between pIC 24HPI vs. PBS 24HPI at 16°C (Figure 2; Table 2; Additional file 1: Table S5). Selected probes having immune-related functional annotations are presented alongside overall fold-change values [i.e. experimental ratio of (ALEXA 647/ALEXA 555) / control ratio of (ALEXA 647/ALEXA 555)] in Tables 1 and 2. For complete information on microarray-identified informative probes, including raw p-values, d-values and standard deviations, please refer to Additional file 1: Tables S2-S5.

**Table 1:** **Selected probes**^**1 **^**representing immune-relevant genes that were differentially expressed between fish injected with PBS or pIC and sampled at 6 hours post-injection (6HPI) at 10 or 16°C, or between fish injected with pIC but held at 10 vs. 16°C and sampled at 6HPI**

**Probes identified as differentially expressed (FDR = 1%) between PBS 6HPI@ 10°C and pIC 6HPI@ 10°C, and between PBS 6HPI@ 16°C and pIC 6HPI@ 16°C**^**2**^				
**Probe ID**	**Best BLASTx hit**^**3**^	**Fold-change (pIC @ 16°C / PBS @ 16°C)**^**4**^	**Fold-change (pIC @ 10°C / PBS @ 10°C)**^**4**^	**Fold-change (pIC @ 16°C / pIC @ 10°C)**^**4**^
38613	Interferon stimulated gene 15 [*Gadus morhua*]	55.97	5.29	8.45
38285	DExD/H box RNA helicase [*Paralichthys olivaceus*] (Aliases: DHX58, LGP2)	5.13	2.44	2.41
45882	Retinoic acid receptor responder protein 3 [*Osmerus mordax*] (Alias: HRAS-suppressor like 3)	12.17	5.51	-
38260	Phosphoinositide phospholipase C-eta-2 [*Homo sapiens*]	5.08	2.19	2.28
44546	Interferon regulatory factor 1 (IRF1) [*Gadus mohua*]	4.74	1.88	2.48
36426	Cell division cycle 42 [*Sus scrofa*]	4.00	2.16	1.85
38042	Deoxyribonuclease gamma precursor [*Oncorhynchus mykiss*]	3.41	1.45	2.18
41397	Prostaglandin E synthase 3 [*Osmerus mordax*]	2.86	1.87	-
44530	NF-kappa-B inhibitor alpha (IκBα) [*Gadus morhua*]	2.15	3.12	-
40721	Nostrin protein [*Danio rerio*]	2.12	2.04	-
39371	Novel immune-type receptor 4 [*Oncorhynchus mykiss*]	2.03	1.52	-
41181	Ubiquitin carboxyl-terminal hydrolase 5 [*Salmo salar*]	1.88	2.04	-
36482	CXC chemokine [*Psetta maxima*]	1.74	1.71	-
**Probes identified as differentially expressed (FDR = 1%) between PBS 6HPI@10°C and pIC 6HPI@10°C only**^**2**^				
**Probe ID**	**Best BLASTx hit**^**3**^	**Fold-change (pIC @ 16°C / PBS @ 16°C)**^**4**^	**Fold-change (pIC @ 10°C / PBS @ 10°C)**^**4**^	**Fold-change (pIC @ 16°C / pIC @ 10°C)**^**4**^
46031	Synaptic vesicle glycoprotein 2B [*Harpegnathos saltator*]	-	3.35	-
44581	NF-kappa-B inhibitor alpha (IκBα) [*Gadus morhua*]	-	1.46	-
37564	Heat shock protein 90 alpha [*Paralichthys olivaceus*]	-	-1.50	-
49256	Unknown	-	-10.59	-
**Probes identified as differentially expressed (FDR = 1%) between PBS 6HPI@16°C and pIC 6HPI@16°C only**^**2**^				
**Probe ID**	**Best BLASTx hit**^**3**^	**Fold-change (pIC @ 16°C / PBS @ 16°C)**^**4**^	**Fold-change (pIC @ 10°C / PBS @ 10°C)**^**4**^	**Fold-change (pIC @ 16°C / pIC @ 10°C)**^**4**^
44448	Small inducible cytokine SCYA104 [*Paralabidochromis chilotes*] (Alias: gmSCYA123^a^)	24.34	-	7.29
38356	Sacsin [*Homo sapiens*] (Alias: SACS)	12.54	-	11.14
48390	Endonuclease domain-containing 1 protein precursor [*Salmo salar*]	9.94	-	-
39328	Zinc finger, NFX1-type containing 1 (ZNFX1) [*Homo sapiens*]	9.05	-	2.67
43196	VHSV-induced protein [*Oncorhynchus mykiss*]	8.03	-	3.35
38639	Interleukin-8 variant 5 (IL-8) [*Ictalurus punctatus*]	7.60	-	5.88
43201	Viperin [*Niphon spinosus*] (Aliases: Radical S-adenosyl methionine domain containing protein 2, RSAD2)	6.89	-	5.43
50762	Unknown	6.70	-	-
37299	GADD45 alpha [*Anoplopoma fimbria*]	2.27	-	3.19
38617	Interferon-inducible GTPase a [*Salmo salar*]	6.53	-	2.80
47384	Unnamed protein product [*Tetraodon nigroviridis*]	6.50	-	7.71
36121	Anti-apoptotic protein NR-13 [*Gadus morhua*]	4.44	-	-
37609	Hepcidin precursor [*Gadus morhua*]	4.30	-	-
36384	CC chemokine type 3 [*Gadus morhua*]	3.55	-	-
44371	Probable E3 ubiquitin-protein ligase RNF144A-A [*Esox lucius*]	3.43	-	-
36190	Bloodthirsty [*Gadus morhua*]	3.38	-	2.05
37904	Type 2 double stranded RNA activated protein kinase [*Gadus morhua*] (Alias: PKR)	2.98	-	2.52
44579	Mitogen-activated protein kinase kinase 4 [*Gadus morhua*]	2.76	-	1.90
38599	Interferon regulatory factor 10 (IRF10) [*Paralichthys olivaceus*]	2.63	-	-
38655	Interferon regulatory factor 7 (IRF7) [*Psetta maxima*]	2.49	-	2.15
38788	Mannose-specific lectin precursor [*Esox lucius*]	2.39	-	-
39119	Ras homolog gene family, member T1a [*Danio rerio*]	2.25	-	1.75
38625	Interleukin 12 receptor beta 2.b [*Danio rerio*]	2.12	-	-
36407	CD9 antigen [*Salmo salar*]	2.05	-	1.44
44615	Toll-like receptor 9 (TLR9) [*Gadus morhua*]	2.03	-	-
37854	Interferon-inducible protein Gig2 [*Siniperca chuatsi*]	2.01	-	-
35877	Alpha-2-macroglobulin [*Epinephelus coioides*]	1.94	-	-
41703	Ras-related protein Rab-10 [*Salmo salar*]	1.90	-	-
36380	CC chemokine type 2 [*Gadus morhua*]	1.89	-	-
36142	Beta-2-microglobulin [*Gadus morhua*]	1.73	-	-
38629	Interleukin-1 receptor-associated kinase 4 (IRAK4) [*Gadus morhua*]	1.67	-	-
38090	GTPase IMAP family member 7 [*Salmo salar*]	1.64	-	-
36328	FLICE-like inhibitory protein [*Oryzias latipes*]	1.58	-	1.40
45132	DEAD (Asp-Glu-Ala-Asp) box polypeptide 10-like [*Bos taurus*]	1.55	-	-
44434	Type 1 death domain-containing protein [*Gadus morhua*]	1.48	-	-
41613	Dhx33 protein [*Xenopus laevis*]	1.47	-	-
37498	GTP-binding nuclear protein Ran [*Osmerus mordax*]	1.43	-	1.36
36122	Novel protein similar to BCL2-related ovarian killer [*Danio rerio*]	1.43	-	-
44431	Caspase 10 [*Gadus morhua*]	1.33	-	1.34
37561	Stress-70 protein, mitochondrial precursor [*Salmo salar*] (Alias: Mortalin)	1.32	-	-
44606	Toll-like receptor 3 (TLR3) [*Gadus morhua*]	1.32	-	-

^1^Probes presented in this table were selected based on their known roles in vertebrate immune responses. Some genes without a BLASTx hit (e.g. Unknowns) were added because they presented a pIC-induced fold-change higher than 5, and therefore, could represent novel immune-relevant genes. For a complete list of microarray-identified informative probes please refer to Additional file 1: Tables S2-S5 and S7-S8.

^2^Fold-change in probe expression is also shown for pIC @ 16°C vs. pIC @ 10°C when significant differential expression was found in the PBS vs. pIC comparisons.

^3^Informative genes were re-annotated using the contig or EST from which the probe printed in the array was designed by comparing the referred sequence to the NCBI’s nr database using the BLASTx algorithm as implemented by Blast2GO [67]. The best BLASTx hit (E-value cutoff of 10^-5^) with an informative name (i.e. not predicted, probable or unnamed protein) is presented in this table. Synonyms were obtained from SWISS-PROT, GENE CARDS and other BLASTx hits as long as they had E-value < 10^-5^, or from the literature (^a^Borza et al. [58]).

^4^Fold-changes are only reported when probes were differently expressed (FDR = 1%) in a given comparison (e.g. pIC@10°C vs. pIC@16°C) and are shown as outputted by siggenes; otherwise a dash (−) is presented in place of the fold-change. Fold-change values preceded by a minus (“-“) sign represent overall fold down-regulation for a particular comparison. For standard deviation, p- and d-values, please refer to Additional file 1: Tables S2-S5 and S7-S8.

**Table 2:** **Selected probes**^**1 **^**representing immune-relevant genes that were differentially expressed between fish injected with PBS or pIC at 24 hour post-injection (24HPI) at 10 or 16°C, or between fish injected with pIC but held at 10 vs. 16°C and sampled at 24HPI**

**Probes identified as differentially expressed (FDR = 1%) between PBS 24HPI@10°C and pIC 24HPI@10°C, and PBS 24HPI@16°C and pIC 24HPI@16°C**^**2**^				
**Probe ID**	**Best BLASTx hit**^**3**^	**Fold-change (pIC @ 16°C / PBS @ 16°C)**^**4**^	**Fold-change (pIC @ 10°C / PBS @ 10°C)**^**4**^	**Fold-change (pIC @ 16°C / pIC @ 10°C)**^**4**^
38604	Interferon stimulated gene 15 (ISG15) [*Gadus morhua*]	38.35	90.24	-3.05
45882	Retinoic acid receptor responder protein 3 [*Osmerus mordax*] (Alias: HRAS-suppressor like 3)	31.10	27.01	-
44590	RIG-I C-terminal domain-containing protein 1 [*Gadus morhua*] (Aliases: DHX58, LGP2)	12.13	30.48	-
48390	Endonuclease domain-containing 1 protein precursor [*Salmo salar*]	11.53	14.24	-
38356	Sacsin [*Homo sapiens*] (Alias: SACS)	9.88	17.37	-
44448	Small inducible cytokine SCYA104 [*Paralabidochromis chilotes*] (Alias: gmSCYA123^a^)	7.28	66.64	-2.34
38638	Interleukin-8 variant 5 (IL-8) [*Ictalurus punctatus*]	6.92	25.33	-5.66
35718	A disintegrin and metalloproteinase domain 8a [*Danio rerio*]	6.12	3.02	-
47329	Unnamed protein product [*Tetraodon nigroviridis*]	5.81	5.52	-
41269	Regulator of nonsense transcripts 1-like [*Bos taurus*]	5.07	13.93	-2.16
44859	Novel protein [*Danio rerio*]	4.98	11.50	-2.34
37854	Interferon-inducible protein Gig2 [*Siniperca chuatsi*]	3.41	4.14	-
44324	Aminopeptidase N [*Camponotus floridanus*]	3.04	3.45	-
44472	CC chemokine type 3 [*Gadus morhua*]	2.75	1.75	-
55231	Unknown	2.65	5.17	-
47410	Probable E3 ubiquitin-protein ligase RNF144A-A [*Salmo salar*]	2.64	7.10	-2.83
47613	Unnamed protein product [*Tetraodon nigroviridis*]	2.48	5.22	-2.72
37542	10 kDa heat shock protein, mitochondrial [*Esox lucius*]	2.45	1.61	-
44919	Novel protein similar to vertebrate IGSF3 [*Danio rerio*]	2.43	3.12	-
44371	Probable E3 ubiquitin-protein ligase RNF144A-A [*Esox lucius*]	2.35	4.56	-1.92
37299	GADD45 alpha [*Anoplopoma fimbria*]	2.25	4.52	-
44598	STAT1 [*Gadus morhua*]	2.12	1.98	-
36190	Bloodthirsty [*Gadus morhua*]	2.11	3.43	-1.78
36612	Complement component C3 [*Paralichthys olivaceus*]	2.07	2.47	-
43196	VHSV-induced protein [*Oncorhynchus mykiss*]	2.02	4.04	-
44546	Interferon regulatory factor 1 (IRF1) [*Gadus mohua*]	1.86	3.35	-1.84
37566	Heat shock protein HSP 90-alpha [*Salmo salar*]	1.80	1.34	1.55
36407	CD9 antigen [*Salmo salar*]	1.78	1.93	-
36426	Cell division cycle 42 [*Sus scrofa*]	1.66	1.93	-
35957	Apolipoprotein A-IV precursor [*Salmo salar*]	1.60	2.58	-1.53
38545	IgD heavy chain constant region variant b [*Gadus morhua*]	1.60	1.43	-
44865	Hect domain and RLD 5 [*Bos Taurus*]	1.52	1.94	-1.40
36923	DnaJ-like subfamily A member 4 [*Paralichthys olivaceus*]	1.51	1.43	-
**Probes identified as differentially expressed (FDR = 1%) between PBS 24HPI@10°C and pIC 24HPI@10°C only**^**2**^				
**Probe ID**	**Best BLASTx hit**^**3**^	**Fold-change (pIC @ 16°C / PBS @ 16°C)**^**4**^	**Fold-change (pIC @ 10°C / PBS @ 10°C)**^**4**^	**Fold-change (pIC @ 16°C / pIC @ 10°C)**^**4**^
38617	Interferon-inducible GTPase a [*Salmo salar*]	-	8.88	-
49247	Unknown	-	5.74	-2.86
43201	Viperin [*Niphon spinosus*] (Aliases: Radical S-adenosyl methionine domain containing protein 2, RSAD2)	-	4.68	-2.45
38260	Phospholipase C-eta-2 [*Homo sapiens*]	-	4.11	-
39368	Novel immune type receptor protein [*Danio rerio*]	-	3.23	
40397	Deltex-3-like [*Salmo salar*]	-	2.76	-2.18
39119	Ras homolog gene family, member T1a [*Danio rerio*]	-	2.66	-
44579	Mitogen-activated protein kinase kinase 4 [*Gadus morhua*]	-	2.61	-1.66
40174	60 kDa heat shock protein, mitochondrial precursor [*Salmo salar*]	-	2.46	-
37609	Hepcidin precursor [*Gadus morhua*]	-	2.44	-
36339	Caspase-1 [*Dicentrarchus labrax*]	-	2.42	-1.78
38788	Mannose-specific lectin precursor [*Esox lucius*]	-	2.30	-1.80
36364	Cathepsin L precursor [*Anoplopoma fimbria*]	-	2.11	-
38599	Interferon regulatory factor 10 (IRF10) [*Paralichthys olivaceus*]		2.05	-
44503	Type 2 double stranded RNA activated protein kinase [*Gadus morhua*] (Alias: PKR)	-	1.93	-
45146	DNA (cytosine-5-)-methyltransferase 7 [Danio rerio]		1.89	-1.79
44434	Type 1 death domain-containing protein [*Gadus morhua*]	-	1.89	-
36797	Cytotoxic and regulatory T cell protein [*Oncorhynchus mykiss*]	-	1.87	-1.81
40721	Nostrin protein [*Danio rerio*]	-	1.86	-
44839	Disulfide-isomerase A3 precursor [*Salmo salar*]	-	1.79	-
44518	Fas [*Gadus morhua*]	-	1.74	-1.77
36121	Anti-apoptotic protein NR-13 [*Gadus morhua*]	-	1.67	-
37498	GTP-binding nuclear protein Ran [*Osmerus mordax*]	-	1.64	-1.54
44550	Interleukin-1 receptor-associated kinase 4 (IRAK4) [*Gadus morhua*]	-	1.63	-1.37
36328	FLICE-like inhibitory protein [*Oryzias latipes*]	-	1.59	-
38619	Interferon-inducible protein Gig1 [*Psetta maxima*]	-	1.55	-
44615	Toll-like receptor 9 (TLR9) [*Gadus morhua*]	-	1.54	-
42836	Tumor necrosis factor receptor-2 [*Paralichthys olivaceus*]	-	1.54	-
42266	Strawberry notch homologue 1 [*Danio rerio*]	-	1.53	-
35877	Alpha-2-macroglobulin [*Epinephelus coioides*]	-	1.52	-
42501	T-complex protein 1 subunit delta [*Takifugu rubripes*]	-	1.46	-
41397	Prostaglandin E synthase 3 [*Osmerus mordax*]	-	1.45	-1.49
36967	E3 SUMO-protein ligase RanBP2 [*Homo sapiens*]	-	1.42	-
38108	Inhibitor of nuclear factor kappa-B kinase subunit alpha [*Danio rerio*] (Alias: IKKα)	-	1.41	-
38631	Interleukin-11a [*Takifugu rubripes*]	-	1.40	-
40138	78 kDa glucose-regulated protein precursor [*Salmo salar*]	-	1.39	-
37118	Novel protein similar to vertebrate EIF4E [*Danio rerio*]	-	1.35	**1.31**
41690	RAS guanyl-releasing protein 1 [*Xenopus tropicalis*]	-	1.33	-
37568	Glucose-regulated protein 94 [*Paralichthys olivaceus*]	-	1.33	-
39371	Novel immune-type receptor 4 [*Oncorhynchus mykiss*]	-	1.31	-1.33
39063	MHC class Ia antigen [*Gadus morhua*]	-	1.28	-
41711	Rab8b protein [*Xenopus tropicalis*]	-	1.27	-
35876	Alpha-2-macroglobulin receptor-associated protein [*Homo sapiens*]	-	1.26	-
41613	Dhx33 protein [*Xenopus laevis*]	-	1.24	-
45113	Methyltransferase-like protein 2 [*Salmo salar*]	-	-1.48	1.44
38713	KH domain-containing, RNA-binding, signal transduction-associated protein 1 [*Salmo salar*]	-	-1.54	-
41688	Novel protein similar to vertebrate IQGAP2 [*Danio rerio*]	-	-1.65	-
37695	G1/S-specific cyclin-D1 [*Salmo salar*]	-	-1.68	-
35934	Annexin max3 [*Oryzias latipes*]	-	-1.83	-
38596	Interferon induced protein 2 [*Ictalurus punctatus*]	-	-2.38	-
**Probes identified as differentially expressed (FDR 1%) between pIC 24HPI@16°C and PBS 24HPI@16°C only**^**2**^				
**Probe ID**	**Best BLASTx hit**^**3**^	**Fold-change (pIC @ 16°C / PBS @ 16°C)**^**4**^	**Fold-change (pIC @ 10°C / PBS @ 10°C)**^**4**^	**Fold-change (pIC @ 16°C / pIC @ 10°C)**^**4**^
44465	CC chemokine type 2 [*Gadus morhua*]	3.76	-	-
37456	Goose-type lysozyme 2 [*Gadus morhua*]	3.36	-	-
36381	CC chemokine type 2 [*Gadus morhua*]	2.98	-	-
42545	Thioredoxin [*Oncorhynchus mykiss*]	2.35	-	2.17
36388	CEBPA protein [*Bos taurus*]	1.86	-	-
36468	CCT epsilon subunit [*Carassius auratus*] (Alias: CCT5)	1.66	-	1.49
38014	Ctssa protein [*Danio rerio*]	1.66	-	-
35923	Annexin A4 [*Ctenopharyngodon idella*]	1.62	-	-
42502	Tcp1 protein [*Danio rerio*] (Alias: CCT1)	1.56	-	-
36813	DEAD (Asp-Glu-Ala-Asp) box polypeptide 23 [*Xenopus tropicalis*]	1.55	-	-
40508	Glutathione S-transferase Mu 3 [*Anoplopoma fimbria*]	1.53	-	1.61
45010	Heat shock protein 90 alpha [*Paralichthys olivaceus*]	1.52	-	1.66
37573	Heat shock protein 90 alpha [*Paralichthys olivaceus*]	1.42	-	1.65
43222	Warm temperature acclimation protein 65–1 [*Dicentrarchus labrax*]	1.42	-	-
37574	Heat shock protein 90 beta [*Paralichthys olivaceus*]	1.37	-	1.52
39904	Peptidylprolyl isomerase A (cyclophilin A) [*Danio rerio*]	1.33	-	-
**Probes identified as differentially expressed (FDR = 1%) between pIC 24HPI @10°C and pIC 24HPI @16°C only**^**2**^				
**Probe ID**	**Best BLASTx hit**^**3**^	**Fold-change (pIC @ 16°C / PBS @ 16°C)**^**4**^	**Fold-change (pIC @ 10°C / PBS @ 10°C)**^**4**^	**Fold-change (pIC @ 16°C / pIC @ 10°C)**^**4**^
37555	Heat shock protein 47 [*Oncorhynchus mykiss*]	-	-	1.77
40546	Heat shock protein HSP 90-alpha [*Harpegnathos saltator*]	-	-	1.64
36470	Chaperonin containing TCP1 subunit 6A [*Paralichthys olivaceus*] (Alias: CCT6)	-	-	1.54
44902	HSP90 multi-domain protein (pfam 00183)	-	-	1.47
37544	Heat shock cognate protein 70 [*Pelodiscus sinensis*] (Alias: HSC71)	-	-	1.35
42467	TANK-binding kinase 1 [*Gadus morhua*]	**-**	**-**	-1.37
37203	Ferritin heavy subunit [*Epinephelus awoara*]	-	-	-1.47
41479	Protein kinase C, delta [*Danio rerio*]	-	-	-1.47

^1^Probes presented in this table were selected based on their known roles in vertebrate immune responses. Some genes without a BLASTx hit (e.g. Unknowns) were added because they presented a pIC-induced fold-change higher than 5, and therefore, could represent novel immune-relevant genes. For a complete list of microarray-identified informative probes please refer to Additional file 1: Tables S2-S5 and S7-S8.

^2^Fold-change in probe expression is also shown for pIC @ 16°C vs. pIC @ 10°C when significant differential expression was found in the PBS vs. pIC comparisons.

^3^Informative genes were re-annotated using the contig or EST from which the probe printed in the array was designed by comparing the referred sequence to the NCBI’s nr database using the BLASTx algorithm as implemented by Blast2GO [67]. The best BLASTx hit (E-value cutoff of 10^-5^) with an informative name (i.e. not predicted, probable or unnamed protein) is presented in this table. Synonyms were obtained from SWISS-PROT, GENE CARDS other BLASTx hits as long as they had E-value < 10^-5^, or from the literature (^a^Borza et al. [58]). Some genes without a BLASTx hit (e.g. Unknowns) were added because they presented a pIC induced fold-change higher than 5, and therefore, could represent novel immune-relevant genes.

^4^Fold-changes are only reported when probes were differently expressed (FDR = 1%) in a given comparison (e.g. pIC@10°C vs. pIC@16°C) and are shown as outputted by siggenes; otherwise a dash (−) is presented in place of the fold-change. Fold-change values preceded by a minus (“-“) sign represent overall fold down-regulation for a particular comparison. For standard deviation, p- and d-values, please refer to Additional file 1: Tables S2-S5 and S7-S8.

We also compared the PBS-injected fish to the temperature-matched pre-injected fish (i.e. CBI and HBI) to ensure that they were suitable controls. In this analysis, we found 29 probes differentially expressed (with FDR = 1%) between the PBS 6HPI@10°C and CBI groups, 40 differentially expressed probes between the PBS 6HPI@16°C and HBI groups, 18 differentially expressed probes between the PBS 24HPI@10°C and CBI groups, and 97 differentially expressed probes between the PBS 24HPI@16°C and HBI groups (for tables containing information on the above lists see Additional file 1: Table S6A-D). Given that only a small overlap (less than 5%) was observed between the probes identified in these comparisons and the pIC vs. PBS comparisons (Additional file 1: Tables S6A–D), we concluded that the PBS groups were a suitable control for injection stress.

### Impact of increased temperature on pIC-induced mRNA expression

In order to study the effect of the gradual temperature increase on the cod spleen transcript expression response to pIC, we performed two direct comparisons: pIC 6HPI@16°C vs. pIC 6HPI@10°C and pIC 24HPI@16°C vs. pIC 24HPI@10°C. This approach detected 290 differentially expressed probes (with FDR = 1%) (279 more highly expressed at 16°C and 11 more highly expressed at 10°C) between pIC 6HPI@16°C and pIC 6HPI@10°C (Table 1; Additional file 1: Table S7) and 339 differentially expressed probes (36 more highly expressed at 16°C and 303 more highly expressed at 10°C) between pIC 24HPI@16°C and pIC 24HPI@10°C (Table 1; Additional file 1: Table S8). Selected microarray-identified probes having immune-related functional annotations are presented with fold-change values in Tables 1 and 2. For complete information on these probes, including raw p-values, d-values and standard deviations, please refer to Additional file 1: Tables S7 and S8.

**Figure 3:**
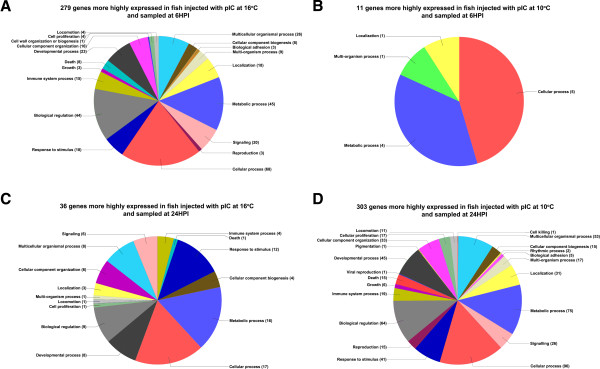
**Summary of GO terms belonging to the biological process branch, represented as pie charts, for genes differentially expressed between fish injected with pIC at 10 and 16°C.** Genes found to have significant differential expression between pIC@10°C and pIC@16°C at both 6HPI and 24 HPI time points were normalized to GO term hierarchy level 2. GO terms that mapped to the 279 genes more highly expressed in pIC@16°C 6HPI compared to pIC@10°C 6HPI are shown in **A**. The terms that mapped to the 11 genes more highly expressed in pIC@10°C 6HPI compared to pIC@16°C 6HPI are shown in **B**. **C** (36 genes) and **D** (303 genes) represent the same analysis, respectively, for the 24HPI time point.

We further analyzed these genes using gene ontology (GO) and hierarchical clustering analyses. Two distinct analyses were used to look at GO terms mapped to genes differentially expressed between fish injected with pIC at 10 and 16°C. One analysis consisted of plotting level 2 GO terms (belonging to the biological process branch) mapped to the genes differentially expressed between fish injected with pIC at 10 and 16°C (Figure 3). The second analysis consisted of using GOSSIP as implemented in Blast2GO to perform Fisher’s exact test to check if any GO terms (belonging to any branch) were significantly (p < 0.01) enriched in the genes responding to pIC (compared to PBS) at 10 and 16°C for both time points. Figure 3 shows four pie charts depicting the proportions of level 2 biological process GO terms of: A) the 279 genes (Figure 2) more highly expressed in fish injected with pIC and sampled 6HPI at 16°C in comparison to the time-matched pIC injected fish at 10°C; B) the 11 genes (Figure 2) more highly expressed in fish injected with pIC at 10°C and sampled 6HPI in comparison to the time-matched pIC injected fish at 16°C; C) the 36 genes (Figure 2) more highly expressed in fish injected with pIC at 16°C and sampled 24HPI in comparison to the time-matched pIC injected fish at 10°C; and D) the 303 genes (Figure 2) more highly expressed in fish injected with pIC at 10°C and sampled 24HPI in comparison with the time-matched pIC injected fish at 16°C.

**Figure 4:**
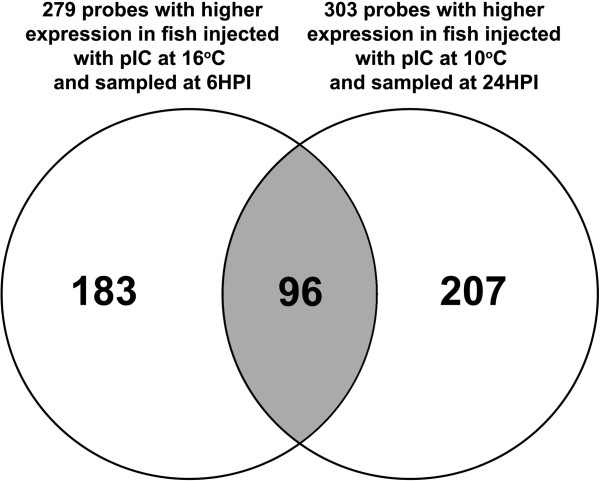
Venn diagram showing the overlap between the genes with significantly higher mRNA expression in pIC@16°C 6HPI compared to pIC@10°C 6HPI and those with significantly higher mRNA expression in pIC @10°C 24HPI compared to pIC@16°C 24HPI.

Several biological processes were highly represented (i.e. by more than 10 sequences) in the 629 genes differentially expressed between fish stimulated with pIC at the different temperatures (i.e. identified in the direct comparisons between fish injected with pIC at 10 and 16°C and sampled at 6HPI and 24HPI). Among these were: cellular component organization, cellular process, developmental process, signaling, biological regulation, response to stimulus, immune system process, and death. Only 4 biological process level 2 GO terms were mapped to the 11 genes more highly expressed in fish stimulated with pIC at 10°C and sampled at 6HPI compared to those stimulated with pIC at 16°C and sampled at the same time point. This is likely a reflection of the low number of probes in this particular gene list. Fifteen of the level 2 biological process GO terms that were mapped to the genes more highly expressed in fish stimulated with pIC at 16°C and sampled at 6HPI were also mapped to the genes that were more highly expressed in fish stimulated with pIC at 10°C and sampled at 24HPI (each compared to its time-matched pIC injected counterpart); we also found that 96 probes were present in both gene lists (Figure 4) (Additional file 1: Table S9). However, many of the GO terms that were in common between these lists had more sequences mapped to them in the list of probes more highly expressed in fish stimulated with pIC at 10°C and sampled at 24HPI compared to the time-matched fish stimulated with pIC at 16°C. Examples are signaling (20 for pIC 6HPI@ 16°C and 26 for pIC 24HPI@10°C), immune system process (15 for pIC 6HPI@16°C and 19 for pIC 24HPI@10°C), death (8 for pIC 6HPI@16°C and 15 for pIC 24HPI@10°C), biological regulation (44 for pIC 6HPI@16°C and 64 for pIC 24HPI@10°C), response to stimulus (18 for pIC 6HPI@16°C and 41 for pIC 24HPI@10°C) and cellular process (68 for pIC 6HPI@16°C and 96 for pIC 24HPI@10°C).

The GO enrichment analysis also identified several GO terms with significantly different over-/under-representation between the genes up-regulated by pIC (relative to PBS) at 24HPI at the two temperatures. Most GO terms (55 out of 57) identified were over-represented in the genes up-regulated by pIC at 16°C compared with those up-regulated by pIC injection at 10°C (all relative to PBS). Examples of these over-represented GO terms in the 24HPI list of pIC responsive genes at 16°C are: protein folding (GO:0006457), negative regulation of cellular process (GO:0048523), negative regulation of protein metabolic process (GO:0051248), regulation of interferon-gamma-mediated-signaling (GO:0060334), regulation of signaling process (GO:0023051), regulation of signal transduction (GO:0009966), regulation of cytokine-mediated signaling pathway (GO:0001959), unfolded protein binding (GO:0051082) and enzyme regulatory activity (GO:0030234). Two GO terms were under-represented in the 24HPI list of pIC responsive genes at 16°C: macromolecule modification (GO:0043412) and protein modification process (GO:0006464).

**Figure 5:**
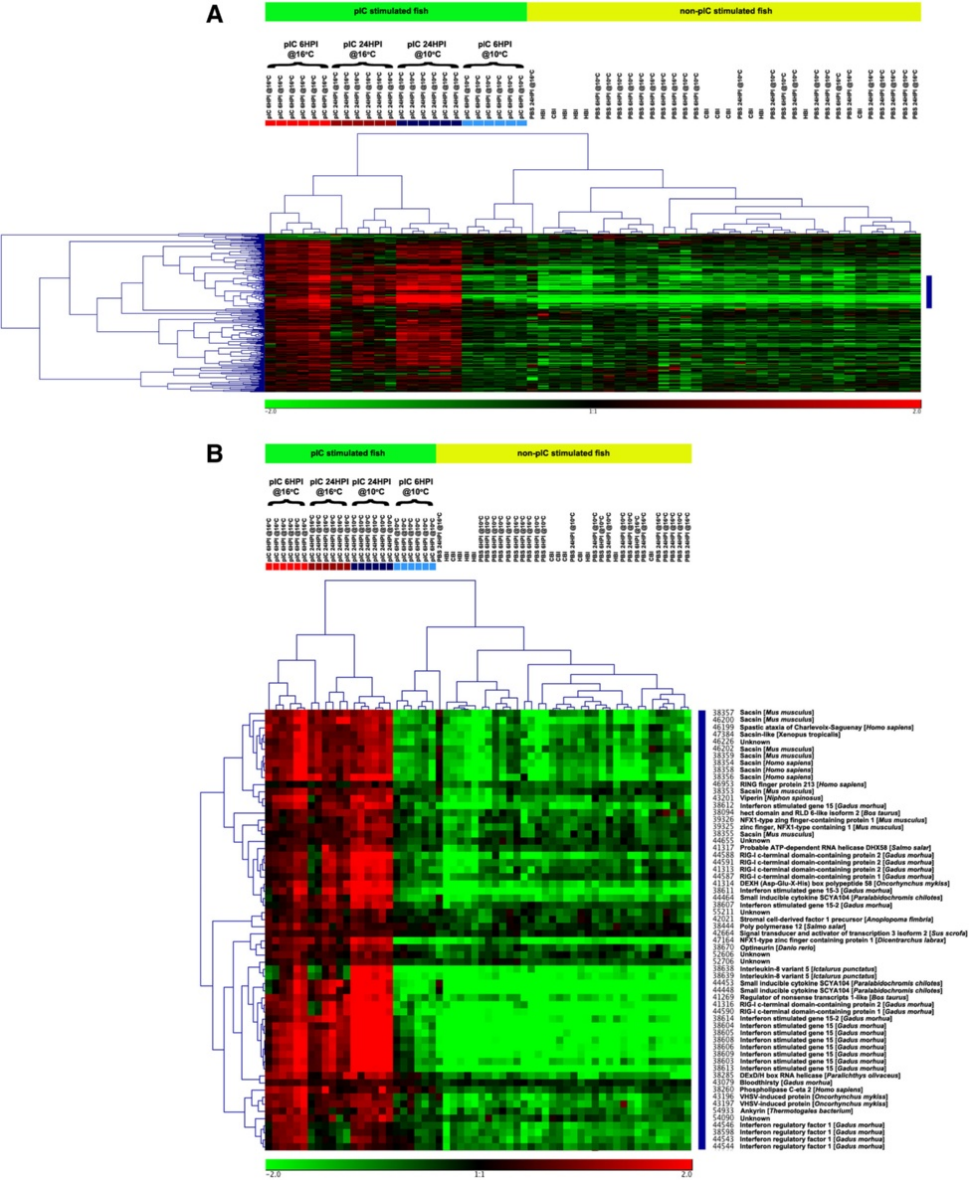
**Heat-map depiction of hierarchical clustering of all samples (i.e. microarrays) and the 290 genes significantly differentially expressed between pIC@10°C and pIC@16°C at 6HPI based on their mRNA expression (see Figure**2**).** The colored boxes below the top legend of each panel (**A** and **B**) represent individual fish from the pIC injected groups at the different temperatures and time points. Light red = pIC@16°C 6HPI; light blue = pIC@10°C 6HPI; dark red = pIC@16°C 24HPI; and dark blue = pIC@10°C 24HPI. Panel **A** shows the complete clustering including all genes. Panel **B** shows only the cluster marked in blue on **A**, which is enriched for genes belonging to the interferon pathway.

**Figure 6:**
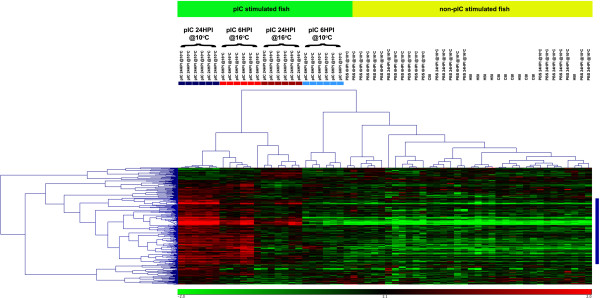
**Heat-map depiction of hierarchical clustering of all samples (i.e. microarrays) and the 339 genes differentialy expressed (FDR = 1%) between pIC@10°C and pIC@16°C at 24HPI based on their mRNA expression (see Figure**2**).** The colored boxes below the top legend represent individual fish from the pIC injected groups at the different temperatures and time points. Light red = pIC@16°C 6HPI; light blue = pIC@10°C 6HPI; dark red = pIC@16°C 24HPI; and dark blue = pIC@10°C 24HPI.

**Figure 7:**
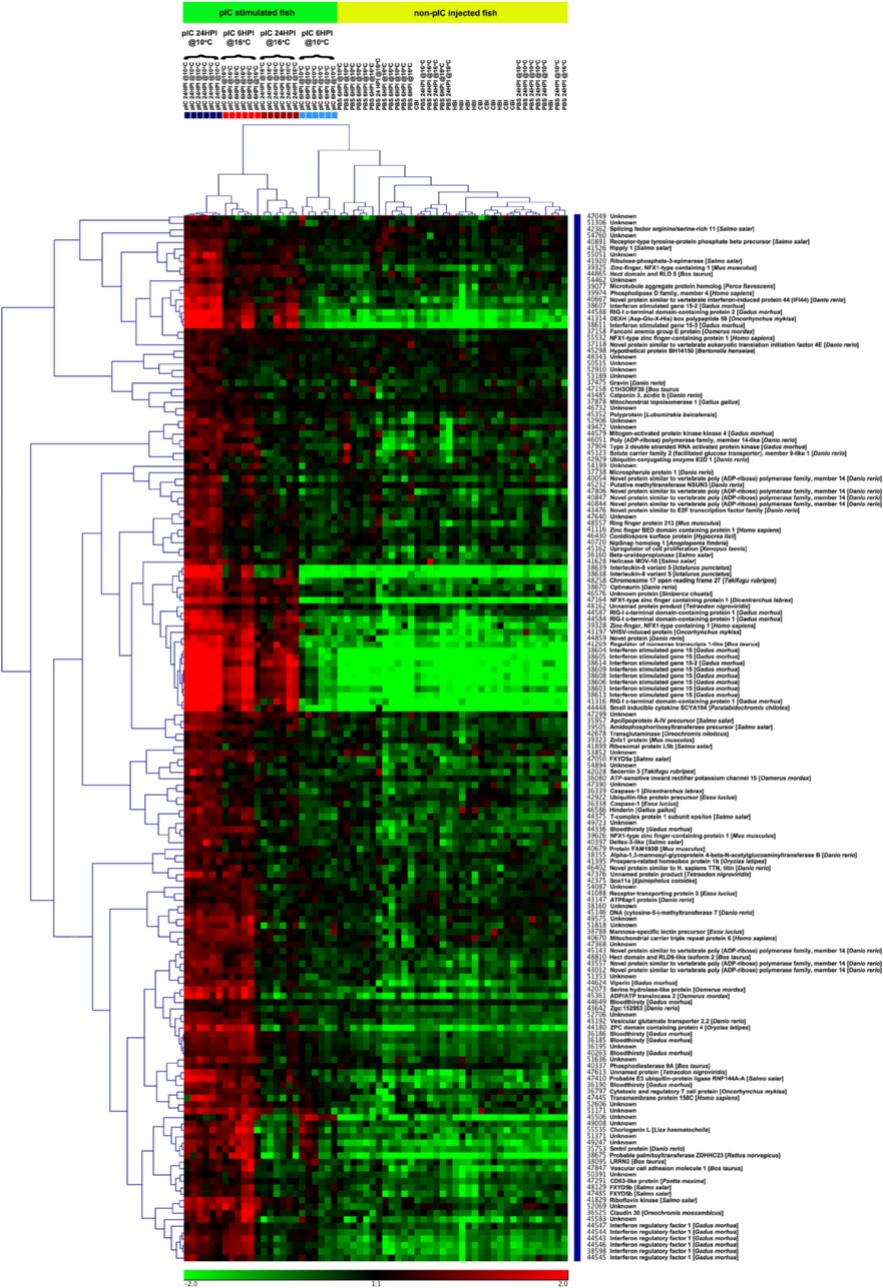
**Detail of a sub-tree (marked in blue on Figure **6**) from the hierarchical clustering of all samples (i.e. microarrays) and the 339 genes differentialy expressed (FDR = 1%) between pIC@10°C and pIC@16°C at 24HPI based on their mRNA expression (see Figures**2**and**6**) enriched for genes belonging to the interferon pathway.** The colored boxes below the top legend represent individual fish from the pIC injected groups at the different temperatures and time points. Light red = pIC@16°C 6HPI; light blue = pIC@10°C 6HPI; dark red = pIC@16°C 24HPI; and dark blue = pIC@10°C 24HPI.

We present 2 separate clustering results displayed as heat-maps (Figures 5, 6 and 7). In these heat-maps, all individuals (60) were clustered based on their expression for the genes identified as differentially expressed between the pIC@16°C and pIC@10°C groups at 6HPI (Figure 5) or at 24HPI (Figure 6 and 7). In Figure 5, panel A shows the entire heat-map for the 6HPI time point, while panel B shows a sub-tree (marked as a blue bar on the right of panel A) dominated by genes belonging to the interferon (IFN) pathway. Figure 6 shows the entire heat-map for the 24HPI time point, while Figure 7 shows a sub-tree (marked as a blue bar on the right of Figure 6) dominated by genes belonging to the interferon (IFN) pathway. Both clustering results show a clear distinction (with a few outliers) between the groups of fish injected with pIC at the different temperatures and time points. Based on the expression of the genes identified in the above-mentioned comparisons, the pIC, 6HPI @10°C group had an expression profile more similar to that of the non-pIC stimulated fish, while the pIC, 6HPI @16°C group’s expression profile was more similar to that of the pIC, 24HPI @10°C group. For the non-pIC stimulated fish (including the PBS-injected and non-injected groups), the clusters were less distinctive.

### QPCR validation of selected immune-relevant genes

**Figure 8:**
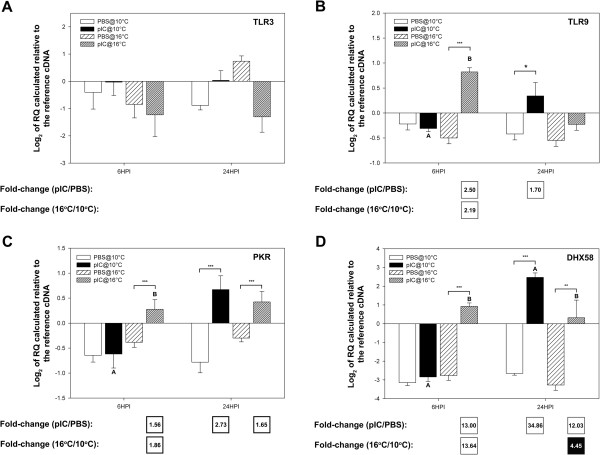
**QPCR results for transcripts with putative roles in the detection of a pathogen (see Additional file ****2****: Figure S1 schema of the TLR/interferon pathways).** Data is presented as mean log_2_ transformed data ± SE. Different letters represent significant differences between fish injected with pIC at different temperatures within each time point. An asterisk (*) represents a difference between a given pIC-injected group and the time- and temperature-matched PBS-injected group (* p < 0.05; ** p < 0.01; *** p < 0.001). Fold-changes in white boxes are always pIC/PBS or 16°C/10°C, and are shown in the boxes beneath each panel. Only significant differences are shown. Black boxes show 1/fold-change for comparisons that yielded fold-change values less than one (for more details on fold-change calculations, refer to the methods section). For example, if white boxes (i.e. black numbers on white background) show 16°C/10°C fold change then a black box would show 10°C/16°C fold change. **A**) TLR3; **B**) TLR9; **C**) PKR; **D**) DHX58.

**Figure 9:**
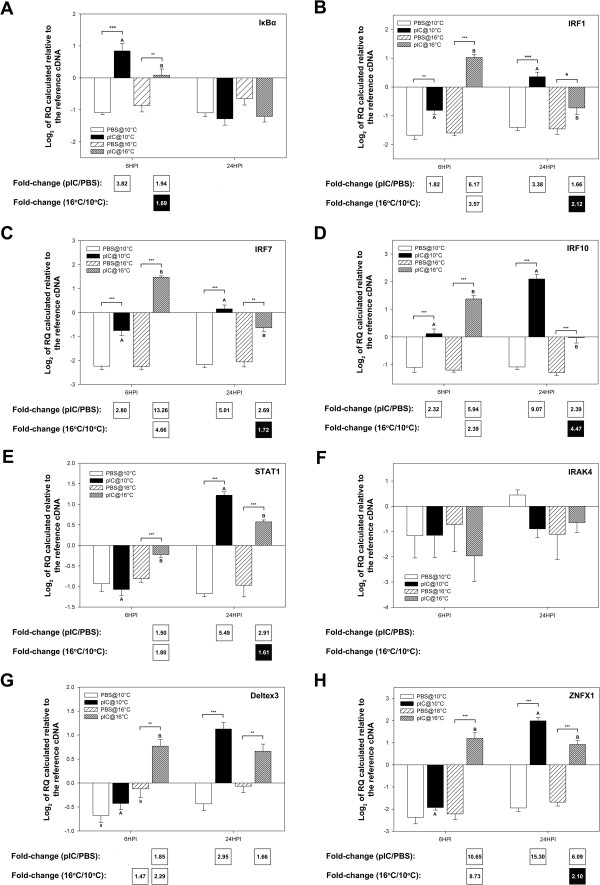
**QPCR results for transcripts with putative roles in signal transduction/transcription control (see Additional file ****2****: Figure S1 schema of the TLR/interferon pathways).** Data is presented as mean log_2_ transformed data ± SE. Different letters represent significant differences between fish injected with pIC at different temperatures within each time point. An asterisk (*) represents a difference between a given pIC-injected group and the time- and temperature-matched PBS-injected group (* p < 0.05; ** p < 0.01; *** p < 0.001). Fold-changes in white boxes are always pIC/PBS or 16°C/10°C and are shown in the boxes beneath each panel. Only significant differences are shown. Black boxes show 1/fold-change for comparisons that yielded values less than one (for more details on fold-change calculations, refer to the methods section). For example, if white boxes (i.e. black numbers on white background) show pIC/PBS fold change then a black box (i.e. white numbers on black background) would show PBS/pIC fold change. **A**) IκBα; **B**) IRF1; **C**) IRF7; **D**) IRF10; **E**) STAT1; **F**) IRAK4; **G**) Deltex3; **H**) ZNFX1.

**Figure 10:**
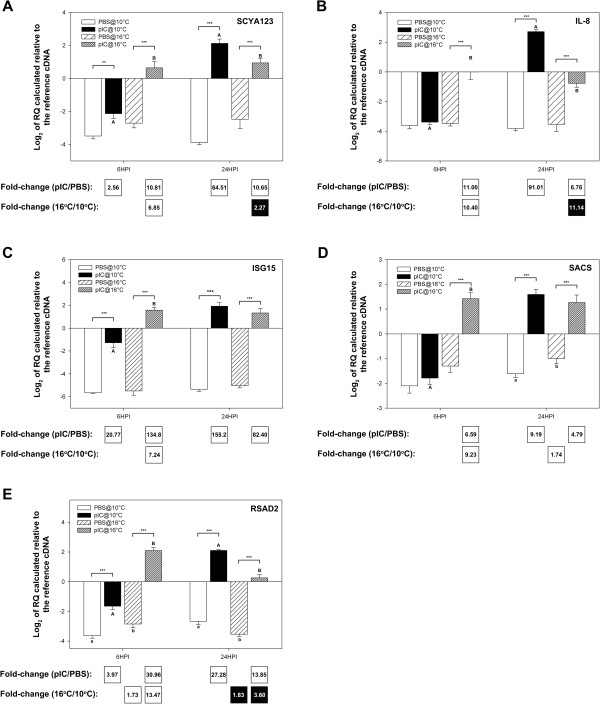
**QPCR results for transcripts with putative roles as immune effectors (see Additional file****2****: Figure S1 schema of the TLR/interferon pathways).** Data is presented as mean log_2_ transformed data ± SE. Different letters represent significant differences between fish injected with pIC at different temperatures within each time point. An asterisk (*) represents a difference between a given pIC-injected group and the time- and temperature-matched PBS-injected group (* p < 0.05; ** p < 0.01; *** p < 0.001). Fold-changes in white boxes are always pIC/PBS or 16°C/10°C and are shown in the boxes underneath each panel. Only significant differences are shown. Black boxes show 1/fold-change for comparisons that yielded values less than one (for more details on fold-change calculations, refer to the methods section). **A**) SCYA123; **B**) IL-8; **C**) ISG15; **D**) SACS; **E**) RSAD2.

Genes of interest (GOI) were selected for QPCR analysis based on their putative roles in pathogen detection, signal transduction/transcription control and as immune effectors; the QPCR data is presented in Figures 8, 9, and 10, respectively, as average log_2_ transformed relative quantities (RQs) ± SE. Fifteen of the immune-relevant genes (88%; all except TLR3 and IRAK4) were validated as being significantly (P < 0.05) differentially expressed in at least one comparison (e.g. pIC 6HPI@10°C vs. PBS 6HPI@10°C) from which they were identified as informative probes in the microarray experiment. There are several possible explanations for disagreement between microarray and QPCR results (e.g. differences between location of QPCR amplicon and microarray probe or possible misassembly of contigs, for more details see the discussion of Booman et al. [21]). For TLR3 and IRAK4 in the current study, there may have been related sequences (e.g. paralogs) binding to these probe in the array and influencing hybridization results. Also, the fact that 3 of the individuals used in the QPCR experiment were not part of the microarray experiment may have contributed to the disagreement between microarray and QPCR results for these two genes. Of the 4 genes with putative pathogen detection roles that were subjected to QPCR, 3 (TLR9, PKR, and DHX58) were validated (i.e. showed a significant difference in at least one of the group comparisons corresponding to the microarray comparison identifying the genes as differentially expressed). In the QPCR experiment for genes potentially involved in pathogen detection, DHX58 showed the highest fold up-regulation in response to pIC (13.00-fold at 6HPI@16°C and 34.86-fold at 24HPI@10°C) and the highest fold differential expression between fish injected with pIC at different temperatures (at 6HPI DHX58 was 13.64-fold more highly expressed in fish injected with pIC at 16°C, and at 24HPI it was 4.45-fold more highly expressed in fish injected with pIC at 10°C) (Figure 8D). PKR showed the same trend but with lower, and yet significant, fold-changes (at 6HPI, 1.56-fold higher in pIC@16°C vs. PBS@16°C and 1.86-fold higher in pIC@16°C vs. pIC@10°C; at 24HPI, 2.73-fold higher in pIC@10°C vs. PBS@10°C and 1.65-fold higher in pIC@16°C vs. PBS@16°C).

Of the 8 microarray-identified genes with putative roles in signal transduction/transcription control analyzed with QPCR, 7 (IκBα, IRF1, IRF7, IRF10, STAT1, Deltex3, ZNFX1) were validated. QPCR analysis showed that IκBα mRNA was only differentially expressed between groups at the 6HPI time point (Figure 9A: 3.82-fold higher in pIC@10°C vs. PBS@10°C, 1.94-fold higher in pIC@16°C vs. PBS@16°C and 1.69-fold higher in pIC@10°C vs. pIC@16°C). All three studied transcripts encoding interferon regulatory factors (IRFs - 1, 7, and 10, Figure 9B, C and D, respectively) showed very similar expression profiles at the mRNA level. These genes responded significantly to pIC at both temperatures and time points, although the magnitude of the fold up-regulation was variable. Of all studied IRFs, IRF7 at the 6HPI time point showed the highest response to pIC (13.26-fold at 16°C) and the highest fold difference between fish injected with pIC at 16 vs. 10°C (4.66-fold). At the 24HPI time point, IRF10 showed the highest response to pIC (9.07-fold at 10°C) and the highest fold difference between fish injected with pIC at 10 vs. 16°C (4.47-fold). Of all the genes with putative roles in signal transduction/transcription control, ZNFX1 showed the highest response to pIC at 10°C (Figure 9H: 15.30 fold up-regulation at 24HPI). As previously mentioned, the microarray results for IRAK4 (Tables 1 and 2) were not validated by QPCR analysis (Figure 9F).

All 5 of the putative immune-effector genes studied with QPCR showed significant differential expression at 6HPI and 24HPI between fish injected with pIC vs. PBS at 10 and/or 16°C (Figure 10A-E). Further, they showed the highest fold-changes of all the transcripts studied with QPCR. At 6HPI, ISG15 showed the highest response to pIC at both temperatures (20.77-fold at 10°C and 134.8-fold at 16°C), and RSAD2 (alias viperin) (Figure 10E) showed the highest differential mRNA expression between fish injected with pIC at the two temperatures (13.47-fold higher in 16°C vs. 10°C). At 24HPI, ISG15 also showed the highest response to pIC at both temperatures (155.2-fold at 10°C and 82.40-fold at 16°C) (Figure 10C), while IL-8 presented the highest fold difference between fish stimulated with pIC at 10 vs. 16°C (11.14-fold higher at 10°C) (Figure 10B). ISG15 was the only microarray identified, QPCR studied putative immune-effector transcript for which not all differences detected on the microarray were validated by QPCR. While ISG15 was identified as being differently expressed (FDR = 1%) between fish injected with pIC at 10 vs. 16°C at 24HPI in the microarray experiment, only a non-significant change in the same direction was observed in the QPCR at 24HPI.

## Discussion

Variations in temperature are an inevitable element of cage-site based aquaculture [8,32,33]. We used a regimen of gradual increases in water temperature that simulated the environmental conditions to which the farmed Atlantic cod can be exposed during the spring-summer transition in sea-cages in Newfoundland, and microarrays and QPCR to study the impact of changes in rearing temperature on the spleen anti-viral transcriptome response of cod. Several studies [34-36] have used functional genomics approaches to study the individual impact of stress or immune stimulation on fish transcriptomes; however, to our knowledge, the current study is the first to use global gene expression analysis to investigate the impact of elevated water temperature on the anti-viral immune transcriptome of a fish species. We were able to demonstrate at the mRNA level that, while a gradual non-lethal increase in water temperature from 10°C to 16°C only caused minor changes in the spleen transcriptome, it had a major influence on the cod spleen gene expression response to IP injection with the viral mimic pIC.

### Influence of elevated temperature on plasma cortisol and the spleen transcriptome

In the present study, plasma cortisol levels did not increase significantly when cod were exposed to the increasing temperature regimen. This finding suggests that our juvenile cod were not stressed under these conditions, and that the differences observed in the pIC response of fish exposed to elevated temperatures may not have been the result of transcriptional changes driven by the glucocorticoid receptor (GR). However, this is in contrast to the results of Pérez-Casanova et al. [9] who reported plasma cortisol values of ~ 52 ng ml^-1^ in similar sized cod that had been warmed from 10°C to 16°C using a similar protocol. This discrepancy is likely due to the fact that Pérez-Casanova et al. [9] sampled their fish as temperature reached 16°C, whereas we sampled the cod after they were given a week to acclimate to this temperature. It is probable that the extra week to which our fish were exposed to 16°C allowed their cortisol (stress) level to return towards basal values. This hypothesis is also supported by Pérez-Casanova et al. [9], as these authors reported that cod plasma cortisol levels decreased on day 40 of their experiment (temperature ~ 17°C) and that values remained at pre-stress levels as temperature was increased further.

Increasing water temperature to 16°C alone had a very small impact on the cod spleen transcriptome (only 6 up-regulated genes were detected at FDR = 1%, Figure 2). Using microarrays, we were unable to detect any differential expression of β2-microglobulin, MHC-I or IgM heavy chain transcripts due to increases in water temperature alone. This result is again different from the results of Pérez-Casanova et al. [9] who showed, using QPCR with blood cDNA templates, that these genes were differentially expressed between cod at 16°C and at 10°C. Although we cannot rule out tissue-specific differences (spleen vs. blood) in the response of these genes to the temperature increase, it is also possible that the different sampling regimes (see above) could explain these disparate findings. When this data is combined with that for plasma cortisol levels, it suggests that prolonged exposure of Atlantic cod juveniles to sea-cage temperatures of 16°C (in the absence of immune challenge) is unlikely to have negative consequences with regards to their stress state or basal immune transcript expression.

### Temperature effects on the Atlantic cod spleen anti-viral transcriptome

Compared to the time- and temperature-matched PBS controls, pIC altered the expression of 41 (37 up-regulated and 4 down-regulated) and 999 (851 up-regulated and 148 down-regulated) transcripts in 10°C fish at 6HPI and 24HPI, respectively, and 656 (617 up-regulated and 39 down-regulated) and 246 (243 up-regulated and 3 down-regulated) transcripts in 16°C fish at 6HPI and 24HPI, respectively (Figure 2). Figure 3 shows a summary of the GO terms that were associated with the genes differentially expressed between fish injected with pIC at the different temperatures and sampled at 6HPI (Figure 3A-B) and 24HPI (Figure 3C-D). Not surprisingly, immune system process, signaling and response to stimulus were found in 3 out of the 4 pie charts in relatively high proportions (Figure 3A, C and D). These results indicate, as previous reports have shown [7,11,12,14], that elevated temperatures can induce changes in the fish’s immune response to an antigen. Furthermore, we suggest that the genes associated with these GO terms (e.g. biological regulation, death, growth, metabolic process; see Figure 3) may be good candidates for the development of molecular markers [e.g. exonic, intronic or regulatory region single nucleotide polymorphisms (SNPs)] to be used in marker assisted selection (MAS) programs aimed at developing elite broodstock (e.g. resistant to potentially negative impacts of elevated temperature on immune response) for the Atlantic cod aquaculture industry.

The sample clustering based on genes differentially expressed between the groups injected with pIC and sampled at 6HPI shows that the expression profile of pIC injected fish held at 10°C and sampled at 6HPI was most similar to that of the non-immune stimulated (i.e. non-injected or injected with PBS) fish than to the profile of any other group of fish injected with pIC (i.e. pIC 6HPI@16°C, pIC 24HPI@10°C and pIC 24HPI@16°C) (Figure 5A). In addition, clustering based on the 339 genes differentially expressed between fish injected with pIC at the different temperatures and sampled at 24HPI (Figures 6 and 7), indicates that the mRNA expression profile of pIC-injected fish held at 16°C and sampled 6HPI was most similar to that of fish injected with pIC at 10°C and sampled at 24HPI. Collectively, these data suggest that the moderate increase in water temperature caused a time shift in the transcriptomic response of cod spleen to pIC, with fish held at elevated temperature (16°C) having an earlier maximum response (i.e. at 6HPI) than fish held at optimal temperature (10°C), which had a later maximum response (i.e. at 24HPI). Raida and Buchmann [11] report similar data for mRNA expression of immune-relevant genes in the spleen of rainbow trout injected with a *Yersinia ruckeri* bacterin. These authors found that peak transcript expression of IL-1β, IL-10 and IFN-γ happened earlier in fish stimulated at 15°C or 25°C when compared to fish stimulated at 5°C. Furthermore, it is clear from our heat-maps that, overall, the magnitude of response to pIC for these genes was greatest in the pIC stimulated fish at 10°C and sampled at 24HPI. This result, however, must be interpreted with caution. Our results suggest that holding cod at 16°C may result in a faster, but weaker, immune response to a viral mimic; this hypothesis could be tested in the future by sampling pIC-injected cod exposed to similar temperature regimes at more frequent intervals post-injection. In future research, more frequent sampling (e.g. every 2 hours post-injection) may allow one to determine if the maximum induction of pIC responsive genes that we observed in the fish held at elevated temperature (16°C) and sampled 6 hours after pIC-injection is the peak response, which would indicate that there is indeed a weaker maximal pIC response in these fish compared with fish held at optimal temperature (10°C). However, if peak spleen transcript expression response to pIC occurs before or after 6HPI for fish held at elevated temperature, and before or after 24HPI for fish held at optimal temperature, this could be determined in future studies incorporating more frequent post-injection sampling.

Using the clustering of genes based on their expression profiles for each sample, we were able to identify many different clusters, including one from each time point that is highly enriched for putative members of the interferon pathway. These clusters are marked in blue on Figures 5A and 6 and shown in detail in Figures 5B and 7. The interferon pathway is a key part of the fish innate response to viruses [20,22,25,37,38], and our results indicate that elevated temperature had a considerable impact on the cod innate immune response to a viral mimic.

### Impacts of the gradual temperature increase on transcript expression

#### Genes with putative viral detection roles

One of the key steps in mounting an anti-viral innate immune response (e.g. expression of type I interferons and proinflammatory cytokines) is the detection of an invading pathogen. This recognition often occurs via the detection of a set of pathogen associated molecular patterns (PAMPs). PAMPs bind specifically to germ-line pattern recognition receptors (PRRs), which in turn activate signaling pathways that induce the innate immune response [39-41]. The immune-stimulant used in this experiment (pIC) is a double-stranded RNA (dsRNA) that mimics the genome and/or RNA intermediates of several viruses and is recognized by PRRs, including the Toll-like receptor 3 (TLR3). Several genes putatively belonging to the TLR pathway (e.g. TLR3) have been identified in fish [36,41] (see Additional file 2: Figure S1 for a schema of a putative type I IFN activation via TLRs pathway in Atlantic cod). While TLR3-like transcript was shown to be slightly up-regulated by pIC in 16°C fish at 6HPI on the microarray (1.32 fold – Table 1), the QPCR analysis did not confirm this result (Figure 8A). In fact, we did not detect any significant changes in TLR3 transcript due to pIC injection using QPCR. This is not surprising as Rise et al. [22] obtained similar QPCR results for TLR3 (using the same primer pair) in spleens from cod stimulated with pIC. However, it suggests that the differences in response to pIC between fish held at 10 vs. 16°C were not caused by an enhanced sensitivity to double-stranded RNA due to an over-expression of TLR3 in the spleens of fish held at 16°C and injected with pIC. The results of Rodriguez et al. [42] for *in vivo* stimulation of rainbow trout with IP injection of pIC also agree with ours, as these authors detected no significant induction of TLR3 mRNA following injection of this viral mimic. Interestingly, these authors and others [43] have shown up to ~30-fold induction of TLR3 transcripts by pIC in isolated rainbow trout cells/cell culture, and this is similar to what has been observed in mammalian macrophages [44].

In the current study, TLR9 transcripts were found by both the microarray and QPCR analyses to be significantly up-regulated by pIC at 6HPI in 16°C fish, and at 24HPI for fish held at 10°C (Table 2; Figure 8B); the QPCR analysis showed that mRNA levels of TLR9 were significantly different between 10 and 16°C pIC injected fish sampled at 6HPI (Figure 8B). Like in mammals, the main ligand of TLR9 in fish is thought to be viral/bacterial unmethylated CpG DNA [41,45]. It is unclear what the roles of TLR9 during the host response to viral dsRNA could be, but it is apparent that temperature can also have an impact on the responses of fish to other pathogens such as bacteria by modulating TLR9 mRNA expression [41]. Further research is warranted to elucidate the modulations of TLRs by antigens and/or changes in the environment in teleost fish.

Other important sensors of viruses are cytosolic PRRs, such as the RIG-I like receptors (RLRs) and the dsRNA activated protein kinase (PKR). DHX58 (alias LGP2) is a RLR that was found to be highly induced at the mRNA level by pIC compared to PBS at both 10°C and 16°C and also differentially expressed (at FDR = 1%) between fish injected with pIC at 10 and 16°C at both time points in the QPCR and microarray experiments (Tables 1 and 2, Figure 8D). Previous studies have reported up-regulation of DHX58 mRNA by pIC in both cod spleen and brain [22,25]. In addition, QPCR was used to show that DHX58 mRNA was significantly up-regulated in the brains [25], but not the spleens [22], of asymptomatic high nodavirus carrier Atlantic cod. As in Rise et al. [22,25] we detected peak up-regulation of DHX58 mRNA at 24HPI for fish injected with pIC at 10°C. In the spleens of fish injected with pIC at 16°C, the fold up-regulation (compared to the PBS control) of DHX58 transcript was similar at 6HPI and 24HPI (13-fold and 12-fold, respectively) (Figure 8D). The roles of DHX58 in the anti-viral response are still unclear [22,46,47]. However, there is some experimental evidence that it may induce or repress RIG-I/MDA5 dependent signal transduction in a virus-dependent manner [46]. Thus, it is still not clear what impacts the observed temperature-dependent modulation of DHX58 response to pIC could have in the anti-viral immunity of cod. We observed similar differences in the expression of PKR mRNA (Figure 8C). Like TLR9, DHX58, STAT1, Deltex3, ZNFX1, IL-8 and SACS, PKR did not respond to pIC at 6HPI in fish held at 10°C, but it was significantly up-regulated at the same time point in the spleens of fish held at 16°C and injected with pIC. In mammals, the main anti-viral function of PKR was originally thought to be the phosphorylation of eIF2α, leading to reduced protein synthesis [48]. More recently, it has been demonstrated that PKR has roles in modulating apoptosis via NF-κB and the growth-inhibitory activity of IRF1 [49]. Therefore the observed influence of pIC, temperature and time on PKR mRNA expression could impact the fish’s ability to fight a viral infection.

#### Genes with putative roles in signal transduction and transcription regulation

In mammals, downstream signaling of TLRs and RLRs involves activation of NF-κB transcription factors [46,50]. There is some evidence of a similar mechanism in fish, since expression constructs containing a constitutively active form of the zebrafish (*Danio rerio*) TLR3 transfected into ZFL cells induced NF-κB mediated luciferase fluorescence [38]. An important step in TLR signaling in mammals is the activation of the Inhibitor of NF-κB Kinases (IKKs), which in turn phosphorylate the Inhibitor of NF-κB proteins (IκBs). Phosphorylation targets IκBs for degradation, and in their absence, the NF-κB proteins accumulate in the nucleus and regulate mRNA transcription [51,52]. In higher vertebrates, IκBα is thought to regulate transient activation of NF-κB. As such it is rapidly degraded in response to stressful stimuli, but then quickly re-synthesized due to the presence of an NF-κB response element in its promoter region [51] (see Additional file 2: Figure S1 for a putative equivalent Atlantic cod pathway). At 6HPI, both the microarray and the QPCR studies showed an significant up-regulation of IκBα mRNA by pIC at both temperatures (Table 1 and Figure 9A). This may be an indication of the process of re-synthesis of this protein in the spleen cells following pIC stimulation. However, the QPCR-detected up-regulation of IκBα mRNA by pIC at 6HPI was higher in fish held at 10°C than in fish held at 16°C (3.82-fold vs. 1.94-fold, respectively). Moreover, the difference in mRNA expression of IκBα between 10°C and 16°C fish injected with pIC at 6HPI was also significant (both in the QPCR and the microarray studies). It is possible that the early response of IFN pathway genes observed in the fish held at 16°C (see Figure 5B and 7) was in part due to a reduced re-synthesis of IκBα, leading to a more pronounced NF-κB-mediated induction of transcription. At 24HPI we detected no significant differences in the mRNA expression of IκBα in fish held at 10°C or 16°C with either the microarray (Table 2) or QPCR (Figure 9A) studies. The microarray analysis indicated that IKKα was significantly up-regulated by pIC at this point only in the spleens of fish held at 10°C (Table 2). Since IKKs are also essential to NF-κB activation, this difference may have contributed to the differences in immune-related gene expression observed at 24HPI (e.g. as shown by the hierarchical clustering; Figures 5, 6 and 7). Collectively, our results suggest that, as in higher vertebrates, timing and intensity of Atlantic cod immune responses are partly regulated by NF-κB transcription factors [51].

The interferon regulatory factor (IRF) genes are also key to the activation of the IFN pathway [53]. In this work all 3 IRFs (i.e. IRF1, IRF7 and IRF10) that had been previously identified as pIC-responsive in cod [22] were shown to be up-regulated in spleen by this viral mimic (Table 1 and 2, Figure 9B, C and D). Moreover, all studied IRFs presented the same overall expression profile as most IFN pathway-related genes identified in this study. This profile is characterized by a higher expression at 6HPI in fish injected with pIC and held at 16°C vs. 10°C, but greater expression in this latter group at 24HPI. In higher vertebrates both IRF1 and IRF7 have been implicated as positive modulators of the type I IFN response to viruses, with IRF7 being essential for interferon-related gene expression [53]. Furthermore, in the current microarray experiment TANK-binding kinase 1 (TBK1), which activates IRF7 by phosphorylation, was found to be more highly expressed in the spleens of fish injected with pIC at 10°C and sampled at 24HPI (1.37 fold – Table 2) than in the spleens of fish injected with pIC at 16°C at this time point. It is likely that the differential up-regulation of the IRFs and TBK1 in fish stimulated with pIC at the different temperatures contributed to the global expression profiles (i.e. with several IFN-related genes peaking at different times in fish injected with pIC at 10°C as compared to those injected with pIC at 16°C) depicted in Figures 5, 6 and 7.

Once type I interferons (e.g. IFN-β) are expressed, they interact with specific cell surface receptors (IFN α/β receptors [IFNAR]) triggering the expression of IFN stimulated genes (ISGs) [54]. This signaling cascade occurs partly through the dimerization of the signal transducer and activator of transcription (STAT) 1 and 2 proteins and their interaction with IRF9. The STAT1/STAT2/IRF9 complex then further induces STAT1 expression [54]. In cod, STAT1 mRNA was previously identified as being significantly more highly expressed in the brains of fish with a high nodavirus carrier status compared to fish with no/low nodavirus; STAT1 mRNA was also shown to be significantly up-regulated in cod brain 24 and 72 h after IP injection with pIC [25]. In the current study we found that: 1) at 6HPI STAT1 was only up-regulated in fish injected with pIC at 16°C; 2) as with the IRFs at 24HPI, both pIC-injected groups showed significant STAT1 mRNA up-regulation; and 3) the STAT1 QPCR-detected up-regulation was more pronounced in the 10°C pIC-injected fish and sampled at 24HPI (5.49-fold at 10°C vs. 2.91-fold at 16°C) (Figure 9E). This suggests that the modulation of the expression of IRFs at the mRNA level could have affected the production of type I IFN, and together with the observed STAT1 transcript expression profiles may also have contributed to the global gene expression profiles depicted in Figures 5, 6 and 7.

#### Genes with putative functions as immune effectors

The shift in the timing of the transcriptome response to pIC between fish stimulated at 10°C vs. 16°C (Figures 5, 6 and 7) was also observed for several immune effector genes (Figure 10A-E). Of the genes studied with QPCR, IL-8 transcript expression is known to be under the control of NF-κB in mammals as evidenced by marked reductions in its expression in IKKε^−/−^ mouse cells [55] (See Additional file 2: Figure S1 for a putative Atlantic cod NF-κB dependent control of immune effector gene transcription). In the present work, IL-8 transcript was 11-fold significantly more highly expressed in the spleens of pIC injected fish held at 16°C and sampled at 6HPI compared with 16°C PBS controls. Further, at 24HPI, IL-8 mRNA was 91-fold up-regulated in the spleens of pIC-injected fish held at 10°C, but only ~ 7-fold up-regulated in the spleens of pIC-injected fish held at 16°C, compared with the time- and temperature-matched PBS controls (Figure 10B). Two of the genes identified in the microarray study and subjected to QPCR validation are known ISGs: ISG15 and RSAD2 (alias viperin) [56]. These genes were previously reported to be pIC-responsive in the cod spleen [22] and significantly up-regulated in the brains of cod by asymptomatic high nodavirus carrier state and IP pIC treatment [25]. It is not surprising that, in the current study, these genes presented the same mRNA expression profiles as the IRFs (Figure 10C and E). For example, ISG15 was ~ 7 fold significantly more highly expressed in 16°C pIC injected fish at 6HPI and 1.51 fold more highly expressed in 10°C pIC-injected fish sampled at 24HPI when compared to the respective time-matched pIC injected fish (Note: This difference was detected in the QPCR experiment but was only significant in the microarray experiment, possibly due to the fact that our ISG15 QPCR primers only amplified 2 of the 3 known ISG15 paralogs [57]). Further, RSAD2 mRNA was 13.5-fold significantly more highly expressed in 16°C pIC-injected fish at 6HPI compared to 10°C pIC-injected cod at 6HPI and 3.6 fold significantly more highly expressed in 10°C pIC-injected fish at 24HPI compared to 16°C pIC-injected fish at the same time point. However, unlike what was observed for IL-8, the peak fold-change in pIC-stimulated fish for ISG15 and RSAD2 mRNA expression (compared with the time- and temperature-matched PBS controls) was similar for 16°C fish at 6HPI as compared with 10°C cod at 24 HPI (Figure 10C and E).

SCYA123 [22] and SACS [25] have both been previously identified as up-regulated by pIC in cod, and as expected both SCYA123 and SACS were significantly up-regulated in the spleen of cod injected with pIC at 10 and 16°C (Figure 10A and D). However, little is known about the roles of these proteins in the anti-viral response. SCYA123 is a small inducible cytokine, but phylogenetic clustering indicates that it is most related to the CCL19 group [58] of higher vertebrates. In mammals, the CCL19 chemokines are constitutively expressed and thought to have a minor role in immunity (see [58] and references therein). Nonetheless, it is clear that SCYA123 transcripts are up-regulated by pIC and other antigens (e.g. formalin-killed atypical *Aeromonas salmonicida*) in the spleen of cod [21-23,58], and therefore, at least in this species, have some role in finfish immunity. Mutations in the SACS gene lead to the human disease spastic ataxia of Charlevoix-Saguenay [59], and it is known that the very large sacsin protein (4579 amino acid residues) encoded by SACS possesses heat shock protein (HSP)-like ATPase domains and HSP-like activity [60]. However, the function of this gene in immunity remains unclear [25]. SACS mRNA was shown by QPCR to be up-regulated by asymptomatic high nodavirus carrier state and IP pIC treatment in brains of cod [25] and by ISA (Infectious Salmon Anemia) virus infection in salmonid TO cells [20]. Moreover, SCYA123 and SACS presented mRNA expression profiles similar to other known immune-related genes reported here (Figures 5, 6 and 7). Interestingly, the transcript expression profiles of SCYA123 and SACS at 6HPI were more similar to those of IL-8 and RSAD2 but at 24HPI was more similar to that of ISG15. Therefore, it is possible that these genes share some of their regulatory mechanisms, and they likely play important roles in Atlantic cod responses to viruses.

#### Cellular response to stress and innate immunity

The mild increase in temperature alone did not cause up-regulation of the genes considered to be part of the conserved cellular stress response (e.g. HSP40, HSP70 and peptidyl-prolyl-isomerase [61]). In contrast, several chaperone-like genes were more highly expressed at 24HPI in the fish injected with pIC at 16°C compared to fish injected with pIC at 10°C. In fact, at this time point 2 GO terms related to protein folding (protein folding and unfolded protein binding) were significantly enriched in the list of genes up-regulated by pIC at 16°C when compared to the list of genes up-regulated by pIC at 10°C. In the microarray experiment, chaperone-like genes such as HSP90-alpha, HSP90-beta, HSC71, HSP47, peptidyl-prolyl-isomerase A, CCT1 and CCT6 (Table 2) were identified as either being induced by pIC at 24HPI in fish injected at 16°C but not in fish injected at 10°C, or significantly more highly expressed at the mRNA level in the pIC@16°C fish at 24HPI when compared to the pIC@10°C (Table 2). Many of these genes (e.g. HSP90-alpha, HSP47 and CCT1) were previously identified as heat shock-responsive in the cod liver [24] and in a multiple-tissue RNA-seq study in rainbow trout [35]. This suggests that even though heat alone did not elicit a robust cellular stress response, the combination of heat and pIC injection did induce molecular biomarkers of cellular stress.

There is evidence from the mammalian literature that HSPs play a key role in modulating the innate immune response [62]. Interestingly at 24HPI, the GO terms “regulation of interferon-gamma-mediated-signaling”, “regulation of signaling process”, “regulation of signal transduction”, and “regulation of cytokine-mediated signaling pathway” were significantly over-represented in the list of genes up-regulated by pIC at 16°C compared to those up-regulated by pIC at 10°C. These GO terms were largely associated with probes representing HSP90-like transcripts or HSP70. It is known that, at least in higher vertebrates, the cellular response to stress prevents innate immune signaling [62], and these effects are to some extent mediated by HSPs. It is possible that the significantly reduced magnitude of induction by pIC observed for some immune-relevant genes (e.g. DHX58, STAT1, SCYA123, IL-8) in Atlantic cod held at 16°C and sampled at 24HPI compared with pIC-injected fish held at 10°C and sampled at 24HPI may have been a result of the activation of the cellular stress response in these animals prior to the 24HPI time point.

## Conclusions

In this study we used microarrays, followed by QPCR, to investigate the interaction between a moderate temperature increase and the innate immune response of cod after pIC stimulation. This is the first time, to our knowledge, that the global gene expression analysis has been used to investigate the interaction between changes in environmental conditions and the antiviral immune response of this commercially important species. We have been able to demonstrate that a moderate increase in seawater temperature, similar to those experienced by farmed Atlantic cod in sea-cages in Newfoundland during the summer, causes a massive shift in the spleen transcriptome response to a viral mimic. Furthermore, we present evidence to suggest that these changes may be, in part, regulated by changes in signal transduction (particularly in the NF-κB and IFN pathways) and by interactions between the cellular stress response and the innate immune response. Collectively, this new information enhances our understanding of the interactions between temperature and the Atlantic cod immune system, and will be valuable in global efforts to improve cod aquaculture practices and broodstock.

## Methods

All experimental procedures were conducted with approval of Memorial University’s Institutional Animal Care Committee, and followed the guidelines of the Canadian Council on Animal Care.

### Experimental animals

Passive integrated transponder (PIT)-tagged Atlantic cod belonging to 10 different families (~ 30 fish per family) from the Atlantic Cod Genomics and Broodstock Development Project (CGP) year-class 3 (YC3) (52.6 ± 2.5 g) were obtained from the Huntsman Marine Science Centre in St. Andrew’s, New Brunswick. The fish were transported to the Ocean Sciences Centre of Memorial University of Newfoundland in October of 2008, and placed in a 3000 l tank supplied with flow-through seawater (at 10°C and > 90% oxygen saturation). After one month of acclimation, the fish were distributed amongst eight 500 l tanks (~ 36 fish per tank) (Figure 1A), each with approximately equal numbers of fish from each family in each tank. Fish were fed to apparent satiation throughout the acclimation period.

### Experimental design

Figure 1 provides an overview of the complete experimental design. Four tanks out of the 8 were randomly assigned as temperature control tanks (i.e. kept at 10°C for the entire duration of the experiment), while the remaining 4 tanks were assigned as heat-exposed (Figure 1A). After the fish were allowed to acclimate to the 500 l tanks for 2 weeks (Figure 1B), 3 fish from each tank (N = 12 per group) had their blood sampled for the determination of plasma cortisol levels. These groups were designated “control before temperature increase” (CBTI) and “heat-exposed before temperature increase” (HBTI). Thereafter, water temperature in the heat-exposed tanks was increased by 1°C every ~ 5 days. These tanks reached 16°C after approximately 1 month, and were held for another ~1 week at that temperature (Figure 1C). Fish were fed to apparent satiation during this ~ 5 week period and no significant differences in food consumption were detected between groups (data not shown). At this point, 8 fish per tank were sampled for plasma cortisol, 2 fish per tank had their spleens removed for subsequent microarray and QPCR analyses, and the remaining fish were given an IP injection of either polyriboinosinic polyribocytidylic acid (pIC) (Sigma Co, St. Louis, MO) [2 μg of pIC g^-1^ wet mass, 0.5 μg μl^-1^ in ice-cold 0.2 μm-filtered phosphate buffered saline (PBS)] or an equivalent volume of ice-cold 0.2 μm filtered PBS after recording their PIT tag ID (Figure 1D). The fish sampled prior to pIC injection were designated as “control before injection” (CBI) and “heat-exposed before injection” (HBI). Finally, at both temperatures (10 and 16°C), 5 fish from each of two tanks (N = 10 per group / temperature) were sampled at 6 hours post-injection (6HPI) and at 24HPI (Figure 1E) to examine the impact of elevated rearing temperature on the spleen’s transcriptome response to the viral mimic pIC.

### Immune stimulation

Prior to immune stimulation, fish were starved for 24 hours and feeding was not resumed thereafter. After being lightly anesthetized using a non-lethal dose (100 mg l^-1^) of tricaine-methane-sulphonate (TMS) (Syndel Laboratories, Qualicum Beach, BC), equal numbers of fish were injected with pIC or PBS. Thereafter, they were allowed to recover (i.e. re-establish normal swimming behavior) in a “recovery-tank” filled with seawater from the same system and temperature as before injection, and then returned to their original tanks. Oxygen levels in the recovery tanks were constantly monitored and maintained above 90% of air saturation.

### Tissue and blood sampling

At all sampling points, the fish were quickly netted from their tanks and placed in an anaesthetic bath containing a lethal dose of TMS (400 mg l^-1^). For cortisol analysis, whole blood was quickly obtained from the caudal vein using heparinized (100 U of sodium heparin ml^-1^) 1 ml syringes (BD Medical Supplies, Franklin Lakes, NJ), centrifuged at 5000 x *g* for 10 minutes (4°C), and the resulting plasma was placed in separate 1.5 ml microcentrifuge tubes and frozen in liquid nitrogen. Spleen samples were collected using standard aseptic molecular biology techniques, placed in certified RNase-free 1.5 ml microcentrifuge tubes and flash-frozen in liquid nitrogen. All spleen samples were stored at −80°C until RNA isolations were performed.

Twelve fish (3 per tank) were sampled for plasma cortisol before the temperature increase (BTI), and compared with values before injection (BI), to allow us to determine the degree of stress induced by the increasing temperature regimen and being held at 16°C for 1 week. Thirty-two fish were sampled prior to the pIC and PBS injections (BI) to allow for correlation analysis of the relationship between temperature-induced plasma cortisol levels and constitutive (pre-injection) spleen immune-related gene expression using QPCR. However, because holding the fish at 10 vs. 16°C only resulted in 6 genes being differentially expressed (see results), this analysis was never performed.

### Plasma cortisol assay

An enzyme-linked immunosorbent assay (ELISA) kit (Neogen, Lexington, KY) was used to measure plasma cortisol levels, and the methods are described in detail elsewhere [24]. The cortisol data was subjected to a Kolmogorov-Smirnov normality test, and a two-way ANOVA (with temperature and time point as main effects). However, neither of these factors had a significant impact on cortisol data, and thus, no further statistical analyses were carried out.

### RNA extractions, DNase-I treatment and column purification

RNA extractions, DNase-I treatment and column purification were performed using the TRIzol reagent (Invitrogen, Carlsbad, CA), RNase-free DNase Set (Qiagen, Valencia, CA) and RNeasy MinElute Clean-up Kit (Qiagen), respectively. All procedures were done according to the manufacturers’ instructions with minor changes as described in Hori et al. [24].

### Microarray hybridizations

A schema of the microarray experimental design is presented in Figure 2. In summary, 6 individuals from each of the following groups were used for microarray analysis: CBI; HBI; pIC 6HPI@10°C; PBS 6HPI@10°C; pIC 6HPI@16°C; PBS 6HPI@16°C; pIC 24HPI@10°C; PBS 24HPI@10°C; pIC 24HPI@16°C; PBS 24HPI@16°C. In these abbreviations, the “@” followed by a number indicates the temperature at which fish were held, pIC refers to fish immune-stimulated with the viral mimic pIC, PBS refers to sham-injected fish and HPI stands for “hours post-injection”.

For all of the above groups, 3 individuals from each of 2 different replicate tanks (N = 6) were selected based on RNA quantity and quality for microarray analysis. Seven μg of spleen DNase I-treated and column-purified total RNA from individuals involved in immune stimulation experiment that included pIC and formalin-killed *Aeromonas salmonicida* injections were pooled to generate a “common reference” sample. The resulting “common reference” pool included all of the individuals used in the present microarray experiment. After pooling, the common reference quantity was measured using spectrophotometry and its quality was checked by 1% agarose gel electrophoresis. For each experimental sample (e.g. pIC 6HPI@10°C, individual 1) or the common reference, 5 μg of DNase I-treated, column-purified RNA were used for target synthesis. Complementary DNA (cDNA) synthesis and labeling were performed using Invitrogen’s SuperScript Direct Labeling kit following the manufacturer’s instructions. The experimental sample was always labeled with ALEXA 647 fluorophore and the common reference was always labeled with ALEXA 555 fluorophore (Figure 2). Labeled cDNA from one experimental sample and the common reference were pooled together and hybridizations were performed at 42°C for ~ 16 hours as described in Booman et al. [21]. Detailed protocols on all hybridizations and washing are available in Booman et al. [21].

### Microarray data acquisition and pre-processing

Microarrays were scanned at 5 μm using a ScanArray Gx Plus scanner and ScanExpress software v4.0 (Perkin Elmer, Woodbridge, ON) with photomultiplier tube (PMT) settings adjusted for each channel of each array and laser power set at 90%. For each channel (i.e. ALEXA 647 or 555) on a given array, the average signal was measured in a circle of diameter ~ 2000 pixels. PMT settings were adjusted in subsequent scans until the average signal was between 600 and 1000 signal units and there was no more than 200 signal units difference between channels. Raw microarray data was saved as TIFF images and probe intensities were extracted using Imagene v7.5 (BioDiscovery, El Segundo, CA). In R, flagged and control spots were removed and the mArray package of Bioconductor was used to log_2_ transform and Loess normalize the data by sub-grid (i.e. print-tip Loess). Thresholding and averaging of the probes were performed as described in Booman et al. [21]. The R scripts are described in full in the supplemental materials of Booman et al. [21]. This microarray dataset is described in the Gene Expression Omnibus (GEO) series GSE27299, and individual samples are available under GEO accession numbers GSM675013-GSM675072 (including both pre- and post-normalization data).

### Microarray data analysis

The Significance Analysis of Microarrays (SAM) algorithm [31] was used to perform two-class comparisons between groups (e.g. pIC 6HPI@10°C vs. pIC 6HPI@16°C) in order to identify differentially expressed genes (at FDR = 1%). Prior to SAM analysis, all probes that were absent in more the 25% of the arrays in the totality of the experiment (i.e. 15 or more out of 60 arrays) were removed from the dataset. This procedure generated a final dataset containing 12,154 probes. Any remaining missing values were imputed using the EM_array method from the LSimpute algorithm [63,64]. We used the Bioconductor implementation of SAM available in the siggenes package [65] and set our False Discovery Rate (FDR) threshold to 1% to generate gene lists. Detailed R scripts are available in the supplemental materials of Booman et al. [21]. Resulting data was then clustered in Genesis [66] using Pearson uncentered correlation and complete linkage hierarchical clustering.

Resulting gene lists were re-annotated using the expressed sequence tags (ESTs) or contiguous sequences (contigs) from which 50 mer oligonucleotide probes on the array were designed. For details on probe design please refer to Booman et al. [21]. Automated BLASTx alignment of these sequences against the NCBI nr database was performed using the Blast2GO tool [67] with thresholds set as follows: E-value <10^-5^, maximum number of hits = 100 and high scoring pairs (HSP) >33. BLASTx results for each probe were mapped to Gene Ontology (GO) terms using Blast2GO. For detailed information on how BLASTx results are mapped to GO terms, please refer to the Blast2GO manual and Conesa et al. [67]. Biological process GO mapping results for the genes differentially expressed between fish injected with pIC at the different temperatures (i.e. 10 vs. 16°C) were normalized to GO term hierarchy level 2 and plotted as pie charts (Figure 3 A-D). GO term enrichment analysis was performed to compare the pIC response at different temperatures using Fisher’s exact test as implemented by GOSSIP in the Blast2GO software with a p-value cutoff of <0.01. We compared the distribution of GO terms between lists of genes differentially expressed in the spleens of fish injected with pIC to those injected with PBS at the different temperatures [e.g. (genes up-regulated in pIC injected fish @10°C vs. time-matched PBS injected controls @10°C) compared to (genes up-regulated in pIC injected fish @16°C vs. time-matched PBS-injected controls @16°C)].

### QPCR analysis

From the informative gene lists that were generated using SAM analysis, the following 4 genes of interest (GOI) were selected for QPCR analysis based on their known transcript expression responses in fish exposed to viruses or viral mimics [20,22,25]: TLR9 (Toll-like receptor 9), PKR (double-stranded RNA activated protein kinase), IRAK4 (interleukin-1 receptor-associated kinase 4), and IκBα (NF-κB inhibitor alpha). The following 13 microarray-identified genes were selected for QPCR because they were previously identified as up-regulated in the spleen of Atlantic cod injected with pIC and/or in brains of cod with high nodavirus carrier state [22,25]: Deltex3, DHX58 (DExD/H box RNA helicase; aliases LGP2, RIG-I-c terminal domain containing protein), IL-8 (interleukin-8 variant 5), IRF1 (interferon regulatory factor 1), IRF7 (interferon regulatory factor 7), IRF10 (interferon regulatory factor 10), ISG15 (interferon stimulated gene 15, paralogs 1 and 3), RSAD2 (radical S-adenosyl methionine domain containing protein 2; alias Viperin), SACS (sacsin; alias spastic ataxia of Charlevoix-Saguenay), SCYA123 [small inducible cytokine 123; this follows the nomenclature for cod CC chemokines from Borza et al. [58]], STAT1 (signal transducer and activator of transcription 1), TLR3 (Toll-like receptor 3), and ZNFX1 (zinc finger, NFX1-type containing 1). Nine of the 17 microarray-identified genes subjected to QPCR (DHX58, IL-8, IRAK4, IRF1, ISG15, PKR, RSAD2, SCYA123 and ZNFX1) were amongst the 96 overlapping probes (Additional file 1: Table S9) between the list of 279 genes more highly expressed in the pIC 6HPI@16°C vs. pIC 6HPI@10°C comparison and the list of 303 genes more highly expressed in the pIC 24HPI@10°C vs. pIC 24HPI@16°C comparison (Figures 2 and 4). QPCR primers were either chosen from previous publications from our laboratory [22,25] (for IRF1, IRF7, IRF10, SACS, Deltex3, STAT1 and ZNFX1) or designed based on sequences representing informative microarray probes using Primer 3 ( http://frodo.wi.mit.edu) (for DHX58, IκBα, IL-8, IRAK4, ISG15, PKR, RSAD2, SCYA123, TLR3, TLR9 and ATPS). Every primer set was quality-checked (QC) using reference cDNA templates generated by reverse transcription of the microarray experiment reference RNA. The QPCR primer QC process was carried out as described in Booman et al. [21]. Briefly, a 5-fold dilution (starting with 10 ng of input RNA) standard curve consisting of 5 points was used to calculate amplification efficiencies [68], and melt-curve analysis was carried out to ensure primer sets amplified a single product and there were no detectable primer dimers. The primer sequences, amplification efficiencies and amplicon sizes are shown in Table 3.

**Table 3:** QPCR Primers

**Gene**		**Primer sequence 5’ to 3’**	**Amplification Efficiency (%)**	**R**^**2**^	**Amplicon Size (bp)**
Deltex3^b^	Forward	TCCACCACAAGACCAGCATCA	98.5	0.99	110
	Reverse	ACTTCACTCGATGCCTTTCGC			
DHX58^c^	Forward	ACAGAAGCCATCGCAGAAAT	92.9	0.99	105
	Reverse	TTTTGCAGCACGAATCAAAC			
IκBα^c^	Forward	GCCAGCAACTGATAAAGCATC	92.1	0.99	132
	Reverse	GGTCACAGAGGGAGACAGAAAA			
IL-8^c^	Forward	GTGTTTCCAGCAGATCACTCG	94.9	0.99	118
	Reverse	TGTTCCACTTGGTGAGGAGTC			
IRAK4^c^	Forward	CGTGGATTACAAGATGGATAAGC	95.8	0.99	102
	Reverse	TCGTCGGGGTCTAAAAAGTC			
IRF1^a^	Forward	AGAAGGACGCCAGTCTGTTCAA	88.5	0.99	100
	Reverse	GCGGAAGTTGGCTTTCCATT			
IRF7^a^	Forward	GGTCGTCGGAGTTCTTGGAGTT	95.1	0.99	102
	Reverse	CCAAACGACAAGGCCAAATG			
IRF10^a^	Forward	CGAGGCGGTAGACCTTGTAG	104.6	0.94	161
	Reverse	GGGCAGGTACAAAGGGAAAT			
ISG15^c^	Forward	AGGACCAACAAAGGCTGATG	94.7	0.99	110
	Reverse	CAGCCGTCCGTTAAGGTAGA			
PKR^c^	Forward	ATTGCATCAGGTTCCCAATC	94.4	0.99	174
	Reverse	GCCGTTACCAGACCAAAATC			
RSAD2^c^	Forward	TGTTTCCACACAGCGAAGAC	95.8	0.99	108
	Reverse	TCCGCCAGAGAAGTTGATCT			
SACS^b^	Forward	CTCCCACTGCCAATGTCATTC	88.5	0.99	102
	Reverse	TCAAGAAAACGTCCCAAGGC			
SCYA123^c^	Forward	GCTCTGGGTCGTGTACCTCT	94.1	1.00	189
	Reverse	TCTCTCTGGACGAACAAGCA			
STAT1^b^	Forward	GCCAATGCCATGTGTTTATG	97.5	0.99	100
	Reverse	ACCTGGAGCAGTTCGTCAGT			
TLR3^c^	Forward	CCTGAAACGCAACTCTATCTCC	90.6	0.99	121
	Reverse	GCCATCAAACATACCCTTCTTT			
TLR9^c^	Forward	TTGCTCGCCAAAACACTATG	99.5	0.99	150
	Reverse	GGAATCCAGTCCCTCTCCTC			
ZNFX1^b^	Forward	ATGCCACTATCGGTGGACAGA	87.3	0.99	108
	Reverse	TCAACAGATTATTGCCCTCGG			
ATPS^c^	Forward	ACATGGATAAATGGCTTTTTGC	99.6	0.98	155
	Reverse	TTGAAGAAGTAGTGTGGCTGGA			

^a^These primers were previously designed and used in Rise et al. [22] but were still quality checked for the present experiment using the reference cDNA as template.

^b^These primers were previously designed and used in Rise et al. [25] but were still quality checked for the present experiment using the reference cDNA as template.

^c^These primers were designed based on ESTs or contigs representing probes that were identified as differentially expressed in the microarray experiment.

Gene expression was normalized to ATPS [ATP synthase H^+^ transporting, mitochondrial Fo complex, subunit F2; CGP microarray probe identifier (ID) #36304] mRNA levels. Several steps were taken to ensure that this gene was a suitable normalizer for this QPCR study. Initially, several candidate probes that had overall normalized ratios (i.e. log_2_ transformed ALEXA 647/ALEXA 555) close to 0 across all 60 microarrays in this study were selected as potential normalizers. For these, the probe IDs for the ones with the lowest standard deviation were searched in all generated gene lists of differentially expressed genes (with FDR = 1%) (Additional files 1: Tables S1-S8), and the ones that were not present in any gene lists were tested with QPCR in a subset of individuals (3 from each condition and time point). ATPS had the lowest threshold cycle (C_T_) range (1.2). Lastly, in every multi-plate experiment C_T_ values for ATPS were always checked; if their range was >1.5 cycles, the samples more distant from the overall C_T_ mean were removed from the study. For any given GOI, no more than 2 individuals had to be removed from the study.

All cDNAs were reverse transcribed from 1 μg of DNase-I treated, column purified RNA using the RETROScript kit (Ambion, Austin, TX) following the manufacturer’s instructions for the protocol that includes template denaturation. Six individuals from each group were analyzed in triplicate reactions. To QPCR validate the microarray experiment-identified GOI, we used 3 individuals that had been used in the microarray and 3 individuals that were not used for microarray hybridizations. Fish included in the QPCR experiment were also selected based on RNA quality and quantity. PCR amplification and cycling were performed as in Booman et al. [21]. In all plates a linker sample consisting of cDNA made from the reference RNA was included for every target (including the normalizer). Prior to analysis, all linker C_T_ values were checked; inter-plate variation was never >0.5 cycles. All thresholds were set automatically, and relative quantities (RQ) were calculated using actual amplification efficiencies for each primer pair and the linker as a calibrator using the Applied Biosystems (Foster City, CA) 7500 Software Relative Quantification Study Application (v2.0) [69].

Resulting RQs were log_2_ transformed in Excel (Microsoft Co., Seattle, WA) and analyzed statistically using Prism (v5.0, GraphPad Software Inc, La Jolla. CA). All groups were initially subjected to a Kolmogorov-Smirnov normality test. For each time point (e.g. 6HPI) a two-way ANOVA with injection (i.e. pIC or PBS) and temperature (10°C or 16°C) as factors was then carried out. When the effects of a given factor were statistically significant (p < 0.05), t-tests were carried out to compare pIC-injected groups to their temperature-matched controls (i.e. PBS injected) at a particular time point, PBS-injected fish held at different temperatures, or pIC-injected fish held at different temperatures. Overall fold-change values were calculated as 2^A-B^ as suggested by Cui and Churchill [70], where A is the average log_2_ transformed RQ from an experimental group (pIC always considered experimental relative to PBS and 16°C always considered experimental relative to 10°C), and B is the average log_2_ transformed RQ from the time- or temperature-matched control.

## Competing interests

The authors declare that they have no competing interests.

## Authors’ contributions

TSH took a lead role in the design and implementation of the heat exposure/immune stimulation experiments, design and execution of microarray and QPCR experiments, data analysis, data interpretation and the writing of this manuscript. AKG was involved in the design of the heat exposure/immune stimulation experiments, data analysis, data interpretation and took an active part in the writing of the manuscript. MB took an active part in the design of the microarray experiments and microarray data analysis and data interpretation. GWN took an active role in the design and implementation of the heat exposure/immune stimulation experiment and sample preparation for the microarray analysis. MLR was involved in the design of the heat exposure/immune stimulation experiment, design of microarray and QPCR experiment data analysis, data interpretation and the writing of this manuscript. All authors read and approved the final manuscript.

## Supplementary Material

Additional file 1 **Tables S1-S9.** Detailed information, including mean fold-change, SAM statistics, probe ID (and CGP EST or contig name of the sequence on which a given probe was based) for the microarray features identified as significantly differentially expressed in the microarray experiment (Figure 2**).** Click here for file

Additional file 2 **Figure S1.** Putative type I interferon activation via Toll-like receptors (TLRs) in Atlantic cod. Click here for file
