# Supplementary material for: A moderate increase in ambient temperature modulates the Atlantic cod (Gadus morhua) spleen transcriptome response to intraperitoneal viral mimic injection
Source: BMC Genomics. 2012 Aug 28;13:431. doi: 10.1186/1471-2164-13-431 (PMC3534563; doi:10.1186/1471-2164-13-431)
Supplement: Additional file 1 — Tables S1-S9. Detailed information, including mean fold-change, SAM statistics, probe ID (and CGP EST or contig name of the sequence on which a given probe was based) for the microarray features identified as significantly differentially expressed in the microarray experiment (Figure 2). [file 1471-2164-13-431-S1.pdf]

Supplemental Table S1. 6 genes differentially expressed between fish exposed to chronic increases in temperature (up to 16°C) compared to fish held at 10°C

Probe ID	d-value	Stdev	Raw p	q-value	Fold Change[a]	Sequence Nane	Gene Name
47777	4.55	0.04	0.0001	0.1762	1.25	sb_gmnlla_0011o17.t7	Unassigned protein
54607	4.37	0.08	0.0001	0.1762	1.40	all_v2.0.3026.C1	unclassified
48342	4.28	0.09	0.0001	0.1762	1.44	sb_gmnlem_0014f04.t7	Unassigned protein
49541	4.17	0.05	0.0001	0.1762	1.26	all_v2.0.17651.C1	unclassified
43904	4.10	0.10	0.0002	0.1762	1.42	all_v2.0.16778.C1	Zgc:65861; n=1; Danio rerio|Rep: Zgc:65861 - Danio rerio (Zebrafish) (Brachydanio rerio)
41261	4.08	0.08	0.0002	0.1762	1.35	sb_gmnlkfic_0004f09.t7	PREDICTED: similar to zinc finger protein; n=3; Danio rerio|Rep: PREDICTED: similar to zinc finger protein - Danio rerio
54419	3.98	0.20	0.0002	0.1762	1.87	all_v2.0.4355.C1	unclassified

[a]Fold-changes are presented as outputted by siggenes and were calculated as 16°C/10°C

Supplemental Table S2. 41 genes differentially expressed between fish injected with pIC or PBS at 10°C and sampled at 6HPI

Probe Id	d-value	Stdev	Raw p	q-value	Fold Change[a]	Sequence Name	Gene Name
45506	11.95	0.25	0.0000	0.0017	10.21	all_v2.0.9616.C1.a	non-directed
38604	6.50	0.46	0.0000	0.0055	8.30	all_v2.0.14.CB2	Interferon stimulated gene 15; n=1; Gadus morhua|Rep: Interferon stimulated gene 15 - Gadus morhua (Atlantic cod)
45882	5.28	0.48	0.0000	0.0111	5.51	all_v2.0.5246.C1	non-directed
38605	7.52	0.28	0.0000	0.0037	5.40	all_v2.0.14.C13	Interferon stimulated gene 15; n=1; Gadus morhua|Rep: Interferon stimulated gene 15 - Gadus morhua (Atlantic cod)
38613	5.19	0.46	0.0000	0.0113	5.29	all_v2.0.14.CB5	Interferon stimulated gene 15; n=1; Gadus morhua|Rep: Interferon stimulated gene 15 - Gadus morhua (Atlantic cod)
38608	5.38	0.28	0.0000	0.0099	3.30	all_v2.0.14.C12	Interferon stimulated gene 15; n=1; Gadus morhua|Rep: Interferon stimulated gene 15 - Gadus morhua (Atlantic cod)
38609	5.03	0.30	0.0000	0.0127	3.29	all_v2.0.14.C3	Interferon stimulated gene 15; n=1; Gadus morhua|Rep: Interferon stimulated gene 15 - Gadus morhua (Atlantic cod)
44530	8.06	0.18	0.0000	0.0037	3.12	IKKAlikeS1	custom - IKKA-like protein
44532	4.39	0.32	0.0001	0.0223	2.71	IKKAlikeS3	custom - IKKA-like protein
38606	5.23	0.23	0.0000	0.0112	2.69	all_v2.0.14.CB4	Interferon stimulated gene 15; n=1; Gadus morhua|Rep: Interferon stimulated gene 15 - Gadus morhua (Atlantic cod)
38285	5.94	0.18	0.0000	0.0075	2.44	sb_gmnlbfic_0007k16.t7	Homolog of Homo sapiens Probable ATP-dependent helicase LGP2; n=1; Takifugu rubripes|Rep: Homolog of Homo sapiens Probable ATP-dependent helicase LGP2 - Takifugu rubripes
44580	7.40	0.13	0.0000	0.0037	2.23	NFkappaB1	custom - NFkappaB - nuclear factor kappa-B, subunit 1
47560	4.76	0.22	0.0000	0.0160	2.22	sb_gmapte_0001n01.pDNRm13r	Unassigned protein
38260	5.90	0.16	0.0000	0.0075	2.19	all_v2.0.16638.C1	Homolog of Homo sapiens PhosPholiPase C-like 3; n=1; Takifugu rubripes|Rep: Homolog of Homo sapiens PhosPholiPase C-like 3 - Takifugu rubripes
36426	4.98	0.19	0.0000	0.0127	2.16	all_v2.0.8306.C2	Cell division cycle 42; n=12; Coelomata|Rep: Cell division cycle 42 - Danio rerio (Zebrafish) (Brachydanio rerio)
51332	4.88	0.20	0.0000	0.0142	2.11	all_v2.0.4629.C1	unclassified
41181	4.32	0.21	0.0001	0.0247	2.04	all_v2.0.14519.C1	PREDICTED: similar to Ubiquitin carboxyl-terminal hydrolase 5 (Ubiquitin thioesterase 5) (Ubiquitin-specific-processing protease 5) (Deubiquitinating enzyme 5) (Isopeptidase T);

							n=2; Homo/Pan/Gorilla group|Rep: PREDICTED: similar to Ubiquitin carboxyl-terminal hydrolase 5 (Ubiquitin thioesterase 5) (Ubiquitin-specific-processing protease 5) (Deubiquitinating enzyme 5) (Isopeptidase T) - Homo sapiens
40721	5.37	0.16	0.0000	0.0099	2.04	sb_gmnbpcic_0001i03.pDNRF2	PREDICTED: similar to Nitric oxide synthase trafficker; n=1; Danio rerio|Rep: PREDICTED: similar to Nitric oxide synthase trafficker - Danio rerio
44546	4.68	0.17	0.0001	0.0171	1.88	IRF1OK4	custom - Interferon regulatory factor 10K
41397	4.56	0.17	0.0001	0.0193	1.87	all_v2.0.847.C1	Prostaglandin E synthase 3; n=5; Danio rerio|Rep: Prostaglandin E synthase 3 - Danio rerio (Zebrafish) (Brachydanio rerio)
44531	4.56	0.18	0.0001	0.0193	1.86	IKKAlikeS2	custom - IKKA-like protein
45633	7.80	0.07	0.0000	0.0037	1.77	all_v2.0.10248.C1	non-directed
55108	4.51	0.15	0.0001	0.0198	1.73	all_v2.0.8631.C1	unclassified
36482	6.69	0.09	0.0000	0.0055	1.71	all_v2.0.1745.C2	Chemokine CXC-like protein; n=1; Psetta maxima|Rep: Chemokine CXC-like protein - Scophthalmus maximus (Turbot)
48012	4.12	0.13	0.0002	0.0330	1.60	sb_gmnlla_0015l21.t7	Unassigned protein
36286	4.75	0.11	0.0000	0.0160	1.58	sb_gmnbgi_0003c04.pDNRF2	Calumenin b; n=3; Otophysi|Rep: Calumenin b - Danio rerio (Zebrafish) (Brachydanio rerio)
50297	5.01	0.10	0.0000	0.0127	1.57	all_v2.0.12538.C1	unclassified
39371	4.86	0.09	0.0000	0.0142	1.52	sb_gmnbhkas_0007i12.pDNRF2	Novel immune-type receptor 4; n=2; Oncorhynchus mykiss|Rep: Novel immune-type receptor 4 - Oncorhynchus mykiss (Rainbow trout) (Salmo gairdneri)
37749	4.99	0.09	0.0000	0.0127	1.50	sb_gmapov_0007g07.pDNRm13r	Homolog of Brachydanio rerio Nucleolin.; n=1; Takifugu rubripes|Rep: Homolog of Brachydanio rerio Nucleolin. - Takifugu rubripes
48214	4.52	0.09	0.0001	0.0198	1.47	sb_gmnbhkas_0014f23.pDNRm13r	Unassigned protein
48591	5.03	0.08	0.0000	0.0127	1.46	sb_gmnlem_0025k19.t7	Unassigned protein
44581	4.64	0.09	0.0001	0.0179	1.46	NFkappaB2	custom - NFkappaB - nuclear factor kappa-B, subunit 2
38042	4.44	0.09	0.0001	0.0210	1.45	all_v2.0.894.C5	Homolog of Homo sapiens Deoxyribonuclease gamma precursor; n=1; Takifugu rubripes|Rep: Homolog of Homo sapiens Deoxyribonuclease gamma precursor - Takifugu rubripes
49081	4.29	0.10	0.0001	0.0260	1.45	all_v2.0.4460.C1	unclassified
52297	4.23	0.09	0.0001	0.0285	1.42	all_v2.0.2007.C1	unclassified
44822	4.87	0.05	0.0000	0.0142	1.30	sb_gmnbmd_0010f21.pDNRF2	G-cadherin [Lytechinus variegatus]

37564	-5.47	0.08	0.0000	0.0099	0.67	all_v2.0.16020.C1	Heat shock protein 90; n=13; Euteleostomi|Rep: Heat shock protein 90 - Scophthalmus maximus (Turbot)
41731	-6.17	0.09	0.0000	0.0065	0.60	sb_gmnblits_0001b02.pDNRF2	Renin binding protein; n=2; Danio rerio|Rep: Renin binding protein - Brachydanio rerio (Zebrafish) (Danio rerio)
46438	-5.43	0.31	0.0000	0.0099	0.30	all_v2.0.15803.C1	Unassigned protein
46031	-5.01	0.33	0.0000	0.0127	0.30	sb_gmnbspic_0004i16.pDNRF2	PREDICTED: similar to CG3168-PA, isoform A [Tribolium castaneum]
49256	-6.60	0.46	0.0000	0.0055	0.09	all_v2.0.8761.C1	unclassified

[a]Fold-changes are presented as outputted by siggenes and were calculated as pIC/PBS

Supplemental Table S3. 656 genes differentially expressed between fish injected with pIC or PBS at 16°C and sampled at 6HPI

Probe ID	d-value	Stdev	Raw p	q-value	Fold Change[a]	Sequence Name	Gene Name
38613	7.59	0.64	0.0000	0.0013	55.97	all_v2.0.14.CB5	Interferon stimulated gene 15; n=1; Gadus morhua|Rep: Interferon stimulated gene 15 - Gadus morhua (Atlantic cod)
38604	9.23	0.56	0.0000	0.0013	46.95	all_v2.0.14.CB2	Interferon stimulated gene 15; n=1; Gadus morhua|Rep: Interferon stimulated gene 15 - Gadus morhua (Atlantic cod)
38605	11.84	0.34	0.0000	0.0013	33.66	all_v2.0.14.C13	Interferon stimulated gene 15; n=1; Gadus morhua|Rep: Interferon stimulated gene 15 - Gadus morhua (Atlantic cod)
44448	8.01	0.47	0.0000	0.0013	24.34	CK10ad	custom - CC chemokine
38608	11.94	0.28	0.0000	0.0013	20.46	all_v2.0.14.C12	Interferon stimulated gene 15; n=1; Gadus morhua|Rep: Interferon stimulated gene 15 - Gadus morhua (Atlantic cod)
44453	7.42	0.49	0.0000	0.0013	19.44	CK10a	custom - CC chemokine
38609	8.60	0.40	0.0000	0.0013	17.72	all_v2.0.14.C3	Interferon stimulated gene 15; n=1; Gadus morhua|Rep: Interferon stimulated gene 15 - Gadus morhua (Atlantic cod)
38606	9.14	0.34	0.0000	0.0013	15.91	all_v2.0.14.CB4	Interferon stimulated gene 15; n=1; Gadus morhua|Rep: Interferon stimulated gene 15 - Gadus morhua (Atlantic cod)
38356	4.85	0.69	0.0000	0.0017	12.54	all_v2.0.415.C6	Homolog of Homo sapiens Spastic ataxia of Charlevoix-Saguenay; n=1; Takifugu rubripes|Rep: Homolog of Homo sapiens Spastic ataxia of Charlevoix-Saguenay - Takifugu rubripes
45882	8.24	0.35	0.0000	0.0013	12.17	all_v2.0.5246.C1	non-directed
44590	8.83	0.33	0.0000	0.0013	11.97	DXH4a	custom - probable ATP-dependent RNA helicase DHX
38675	5.71	0.56	0.0000	0.0014	11.87	all_v2.0.9761.C1	Isoform S of Q76IC6 ; n=1; Rattus norvegicus|Rep: Isoform S of Q76IC6 - Rattus norvegicus (Rat)
38611	6.93	0.39	0.0000	0.0013	10.84	all_v2.0.1767.C1	Interferon stimulated gene 15; n=1; Gadus morhua|Rep: Interferon stimulated gene 15 - Gadus morhua (Atlantic cod)
38614	10.91	0.23	0.0000	0.0013	10.76	all_v2.0.7062.C2	Interferon stimulated gene 15; n=1; Gadus morhua|Rep: Interferon stimulated gene 15 - Gadus morhua (Atlantic cod)
38603	6.14	0.50	0.0000	0.0013	10.35	all_v2.0.14.CB7	Interferon stimulated gene 15; n=1; Gadus morhua|Rep: Interferon stimulated gene 15 - Gadus morhua (Atlantic cod)
44180	6.44	0.47	0.0000	0.0013	10.20	all_v2.0.1266.C1	ZPC4; n=2; Oryzias latipes|Rep: ZPC4 - Oryzias latipes (Medaka fish) (Japanese ricefish)
48390	4.12	0.65	0.0001	0.0025	9.94	all_v2.0.15354.C1	Unassigned protein
40869	2.75	0.91	0.0012	0.0178	9.54	sb_gmnbspic_0014p07.pDNRF2	PREDICTED: similar to Pre-mRNA processing splicing factor 8 (Splicing factor Prp8) (PRP8 homolog) (220 kDa U5 snRNP-specific protein) (p220) isoform 13; n=4; Deuterostomia|Rep: PREDICTED: similar to Pre-mRNA processing splicing factor 8 (Splicing factor Prp8) (PRP8 homolog) (220 kDa U5 snRNP-specific protein) (p220) isoform 13 - Canis familiaris
39328	4.61	0.71	0.0000	0.0018	9.05	all_v2.0.2213.C1	NFX1-type zinc finger-containing protein 1; n=5; Eutheria|Rep: NFX1-type zinc finger-containing protein 1 - Homo sapiens (Human)
41316	7.29	0.38	0.0000	0.0013	8.89	all_v2.0.1009.C5	Probable ATP-dependent helicase LGP2 (EC 3.6.1.-) (Protein D11Lgp2 homolog).; n=1; Takifugu rubripes|Rep: Probable ATP-dependent helicase LGP2 (EC 3.6.1.-) (Protein D11Lgp2 homolog). - Takifugu rubripes
44589	3.57	1.12	0.0001	0.0047	8.69	DXH3b	custom - probable ATP-dependent RNA helicase DHX

38651	5.24	0.50	0.0000	0.0016	8.51	sb_gmapht_0023a03.pDNRF2	Intraflagellar transport protein 52; n=4; Danio rerio|Rep: Intraflagellar transport protein 52 - Brachydanio rerio (Zebrafish) (Danio rerio)
45361	6.50	0.39	0.0000	0.0013	8.25	all_v2.0.34.C2	LOC797694; similar to solute carrier family 25 member 5 protein
41269	6.26	0.44	0.0000	0.0013	8.18	sb_gmnlbfic_0004o20.t7	PREDICTED: similar to zinc finger, NFX1-type containing 1; n=3; Laurasiatheria|Rep: PREDICTED: similar to zinc finger, NFX1-type containing 1 - Equus caballus
43196	7.47	0.33	0.0000	0.0013	8.03	all_v2.0.3448.C3	VHSV-induced protein; n=1; Oncorhynchus mykiss|Rep: VHSV-induced protein - Oncorhynchus mykiss (Rainbow trout) (Salmo gairdneri)
38639	5.19	0.44	0.0000	0.0016	7.60	all_v2.0.638.CB1	Interleukin-8 variant 2; n=3; Ictalurus punctatus|Rep: Interleukin-8 variant 2 - Ictalurus punctatus (Channel catfish)
38638	3.42	0.86	0.0002	0.0059	7.30	all_v2.0.638.C1	Interleukin-8 variant 2; n=3; Ictalurus punctatus|Rep: Interleukin-8 variant 2 - Ictalurus punctatus (Channel catfish)
38354	5.87	0.41	0.0000	0.0014	7.26	all_v2.0.707.C4	Homolog of Homo sapiens Spastic ataxia of Charlevoix-Saguenay; n=1; Takifugu rubripes|Rep: Homolog of Homo sapiens Spastic ataxia of Charlevoix-Saguenay - Takifugu rubripes
38358	4.87	0.53	0.0000	0.0017	6.97	all_v2.0.9190.C1	Homolog of Homo sapiens Spastic ataxia of Charlevoix-Saguenay; n=1; Takifugu rubripes|Rep: Homolog of Homo sapiens Spastic ataxia of Charlevoix-Saguenay - Takifugu rubripes
46200	5.00	0.51	0.0000	0.0016	6.94	all_v2.0.14575.C1	SACS; sacsin
49247	8.01	0.26	0.0000	0.0013	6.92	all_v2.0.7226.C1	unclassified
43201	6.31	0.35	0.0000	0.0013	6.89	all_v2.0.9330.C1	Viperin-like protein; n=1; Channa argus|Rep: Viperin-like protein - Channa argus (snakehead)
44588	8.41	0.25	0.0000	0.0013	6.74	DXH2b	custom - probable ATP-dependent RNA helicase DHX
50762	4.05	0.58	0.0001	0.0026	6.70	all_v2.0.1609.C1	unclassified
38612	8.09	0.25	0.0000	0.0013	6.65	all_v2.0.14.CB3	Interferon stimulated gene 15; n=1; Gadus morhua|Rep: Interferon stimulated gene 15 - Gadus morhua (Atlantic cod)
41314	7.62	0.26	0.0000	0.0013	6.60	all_v2.0.5598.C1	Probable ATP-dependent helicase LGP2 (EC 3.6.1.-) (Protein D11Lgp2 homolog).; n=1; Takifugu rubripes|Rep: Probable ATP-dependent helicase LGP2 (EC 3.6.1.-) (Protein D11Lgp2 homolog). - Takifugu rubripes
38617	5.51	0.43	0.0000	0.0014	6.53	all_v2.0.13188.C1	Interferon-inducible GTPase 5 (EC 3.6.5.-) (Immunity-related GTPase cinema 1).; n=3; Takifugu rubripes|Rep: Interferon-inducible GTPase 5 (EC 3.6.5.-) (Immunity-related GTPase cinema 1). - Takifugu rubripes
47384	4.97	0.48	0.0000	0.0016	6.50	all_v2.0.707.C1	Unassigned protein
43197	4.77	0.54	0.0000	0.0017	6.41	all_v2.0.3448.C4	VHSV-induced protein; n=1; Oncorhynchus mykiss|Rep: VHSV-induced protein - Oncorhynchus mykiss (Rainbow trout) (Salmo gairdneri)
47164	4.98	0.52	0.0000	0.0016	6.26	all_v2.0.6027.C6	Unassigned protein
38357	4.77	0.52	0.0000	0.0017	6.04	all_v2.0.8243.C1	Homolog of Homo sapiens Spastic ataxia of Charlevoix-Saguenay; n=1; Takifugu rubripes|Rep: Homolog of Homo sapiens Spastic ataxia of Charlevoix-Saguenay - Takifugu rubripes
46199	7.50	0.25	0.0000	0.0013	5.65	all_v2.0.2412.C1	SACS; sacsin
44543	7.56	0.24	0.0000	0.0013	5.57	IRF1OK1	custom - Interferon regulatory factor 10K
38359	4.64	0.47	0.0000	0.0018	5.43	all_v2.0.415.C5	Homolog of Homo sapiens Spastic ataxia of Charlevoix-Saguenay; n=1; Takifugu rubripes|Rep: Homolog of Homo sapiens Spastic ataxia of Charlevoix-Saguenay - Takifugu rubripes

45506	6.13	0.32	0.0000	0.0013	5.39	all_v2.0.9616.C1.a	non-directed
44859	3.79	0.70	0.0001	0.0036	5.28	all_v2.0.314.C1	HECT domain containing protein
38285	9.14	0.18	0.0000	0.0013	5.13	sb_gmnlbfic_0007k16.t7	Homolog of Homo sapiens Probable ATP-dependent helicase LGP2; n=1; Takifugu rubripes|Rep: Homolog of Homo sapiens Probable ATP-dependent helicase LGP2 - Takifugu rubripes
38598	7.11	0.25	0.0000	0.0013	5.09	all_v2.0.1580.C7	Interferon regulatory factor 1; n=1; Channa argus|Rep: Interferon regulatory factor 1 - Channa argus (snakehead)
38260	8.18	0.20	0.0000	0.0013	5.08	all_v2.0.16638.C1	Homolog of Homo sapiens PhosPholiPase C-like 3; n=1; Takifugu rubripes|Rep: Homolog of Homo sapiens PhosPholiPase C-like 3 - Takifugu rubripes
42073	6.13	0.28	0.0000	0.0013	5.04	all_v2.0.15254.C1	Serine hydrolase-like protein 2 (EC 3.1.-.-).; n=1; Takifugu rubripes|Rep: Serine hydrolase-like protein 2 (EC 3.1.-.-). - Takifugu rubripes
48563	2.75	0.96	0.0012	0.0177	5.03	sb_gmnlla_0011n07.t7	Unassigned protein
47410	6.83	0.26	0.0000	0.0013	4.95	sb_gmnlsfic_0004h02.t7	Unassigned protein
47329	3.44	0.77	0.0002	0.0057	4.88	all_v2.0.415.C3	Unassigned protein
46226	4.71	0.40	0.0000	0.0018	4.79	all_v2.0.415.C1	Serpentine_recp domain containing protein
44584	3.81	0.62	0.0001	0.0036	4.76	DXH1a	custom - probable ATP-dependent RNA helicase DHX
36311	5.07	0.40	0.0000	0.0016	4.75	all_v2.0.12206.C1	Cardiac troponin T; n=14; Danio rerio|Rep: Cardiac troponin T - Danio rerio (Zebrafish) (Brachydanio rerio)
44546	5.08	0.38	0.0000	0.0016	4.74	IRF1OK4	custom - Interferon regulatory factor 10K
55535	2.50	0.64	0.0025	0.0264	4.68	all_v2.0.15359.C1	zp3a.1; zona pellucida glycoprotein 3a.1
46202	6.05	0.27	0.0000	0.0013	4.62	sb_gmnbmd_0010a21.pDNRm13r	Sacs_predicted; sacsin (predicted)
35753	5.35	0.31	0.0000	0.0015	4.59	sb_gmnlsras_0003g09.t7	Actin-binding vascular maintenance protein; n=5; Danio rerio|Rep: Actin-binding vascular maintenance protein - Danio rerio (Zebrafish) (Brachydanio rerio)
40939	3.55	0.46	0.0002	0.0049	4.47	all_v2.0.910.C2	PREDICTED: similar to Rhomboid, veinlet-like 7 (Drosophila); n=1; Danio rerio|Rep: PREDICTED: similar to Rhomboid, veinlet-like 7 (Drosophila) - Danio rerio
36121	4.71	0.41	0.0000	0.0018	4.44	all_v2.0.5784.C1	Bcl2l10 protein; n=2; Danio rerio|Rep: Bcl2l10 protein - Danio rerio (Zebrafish) (Brachydanio rerio)
41892	2.44	0.83	0.0030	0.0296	4.34	sb_gmlbgits_0002g20.t7	Ribosomal protein L32; n=4; Euteleostei|Rep: Ribosomal protein L32 - Epinephelus coioides (Orange-spotted grouper)
37609	3.06	0.58	0.0005	0.0105	4.30	all_v2.0.796.C2	Hepcidin; n=1; Pseudosciaena crocea (Croceine croaker)|Rep: Hepcidin - Pseudosciaena crocea (Croceine croaker)
43007	7.56	0.20	0.0000	0.0013	4.28	sb_gmapov_0013f18.pDNRF2	UPI000065EC34 related cluster; n=1; Takifugu rubripes|Rep: UPI000065EC34 UniRef100 entry - Takifugu rubripes
44909	2.95	0.74	0.0007	0.0125	4.27	all_v2.0.3619.C1	Ifi-6-16 multi-domain protein
51332	6.51	0.24	0.0000	0.0013	4.25	all_v2.0.4629.C1	unclassified
49317	4.01	0.44	0.0001	0.0027	4.13	all_v2.0.13805.C1	unclassified
51171	5.27	0.33	0.0000	0.0015	4.08	all_v2.0.12475.C1	unclassified
44464	5.07	0.30	0.0000	0.0016	4.07	CK10add	custom - CC chemokine
38791	3.49	0.52	0.0002	0.0053	4.01	all_v2.0.8167.C1	LIM and senescent cell antigen-like-containing domain protein 1; n=22; Euteleostomi|Rep: LIM and senescent cell antigen-like-containing domain

38791	3.49	0.52	0.0002	0.0053	4.01	all_v2.0.8167.C1	LIM and senescent cell antigen-like-containing domain protein 1; n=22; Euteleostomi|Rep: LIM and senescent cell antigen-like-containing domain protein 1 - Homo sapiens (Human)
36426	9.88	0.13	0.0000	0.0013	4.00	all_v2.0.8306.C2	Cell division cycle 42; n=12; Coelomata|Rep: Cell division cycle 42 - Danio rerio (Zebrafish) (Brachydanio rerio)
39490	7.41	0.18	0.0000	0.0013	3.86	all_v2.0.5879.C2	Novel protein similar to vertebrate nebulin; n=3; Danio rerio|Rep: Novel protein similar to vertebrate nebulin - Danio rerio (Zebrafish) (Brachydanio rerio)
41313	4.58	0.40	0.0000	0.0018	3.85	all_v2.0.1009.C1	Probable ATP-dependent helicase LGP2 (EC 3.6.1.-) (Protein D11Lgp2 homolog).; n=1; Takifugu rubripes|Rep: Probable ATP-dependent helicase LGP2 (EC 3.6.1.-) (Protein D11Lgp2 homolog). - Takifugu rubripes
47050	4.70	0.33	0.0000	0.0018	3.78	sb_gmapte_0012e14.pDNRF2	Unassigned protein
45583	3.28	0.48	0.0003	0.0072	3.77	all_v2.0.2772.C15	non-directed
54090	7.28	0.18	0.0000	0.0013	3.77	all_v2.0.2294.C1	unclassified
45325	6.70	0.21	0.0000	0.0013	3.76	all_v2.0.14677.C1	LOC764254; similar to transposase
47445	6.27	0.23	0.0000	0.0013	3.75	sb_gmnbmd_0004h11.t7	Unassigned protein
38095	3.46	0.41	0.0002	0.0055	3.67	all_v2.0.6540.C1	Homolog of Homo sapiens Hemicentin; n=1; Takifugu rubripes|Rep: Homolog of Homo sapiens Hemicentin - Takifugu rubripes
44591	8.96	0.13	0.0000	0.0013	3.67	DXH4b	custom - probable ATP-dependent RNA helicase DHX
42784	3.39	0.54	0.0002	0.0061	3.66	sb_gmnblits_0022m22.pDNRF2	Transposase; n=27; Anopheles gambiae|Rep: Transposase - Anopheles gambiae (African malaria mosquito)
41027	3.17	0.68	0.0004	0.0087	3.66	sb_gmlbgits_0004k02.sp6	PREDICTED: similar to SPAC167.01; n=1; Danio rerio|Rep: PREDICTED: similar to SPAC167.01 - Danio rerio
47847	3.26	0.42	0.0003	0.0075	3.65	all_v2.0.14003.C2	Unassigned protein
51629	2.97	0.66	0.0007	0.0122	3.59	all_v2.0.11166.C1	unclassified
45139	2.80	0.69	0.0011	0.0164	3.56	sb_gmnbhkic_0005c17.pDNRF2	LOC539080; similar to immunity-related GTPase family, cinema 1
36384	5.29	0.25	0.0000	0.0015	3.55	sb_gmnbhkas_0018b23.pDNRm13r	CC chemokine type 3; n=1; Gadus morhua|Rep: CC chemokine type 3 - Gadus morhua (Atlantic cod)
42223	3.88	0.42	0.0001	0.0032	3.55	all_v2.0.3958.C1	Single-stranded DNA-binding protein, mitochondrial precursor (Mt-SSB) (MtSSB) (PWP1-interacting protein 17).; n=1; Takifugu rubripes|Rep: Single-stranded DNA-binding protein, mitochondrial precursor (Mt-SSB) (MtSSB) (PWP1-interacting protein 17). - Takifugu rubripes
36525	3.10	0.44	0.0005	0.0098	3.49	all_v2.0.16620.C1	Claudin 30; n=1; Oreochromis mossambicus|Rep: Claudin 30 - Oreochromis mossambicus (Mozambique tilapia) (Tilapia mossambica)
36186	6.92	0.18	0.0000	0.0013	3.48	all_v2.0.661.C2	Bloodthirsty; n=2; Notothenioidei|Rep: Bloodthirsty - Chaenocephalus aceratus (White crocodile fish)
37831	5.04	0.28	0.0000	0.0016	3.45	sb_gmnlsfas_0002e11.t7	Homolog of Brachydanio rerio Transmembrane 9 superfamily member 2.; n=1; Takifugu rubripes|Rep: Homolog of Brachydanio rerio Transmembrane 9 superfamily member 2 - Takifugu rubripes
44371	2.70	0.65	0.0014	0.0191	3.43	all_v2.0.2805.C1	caspc; caspase c
							Homolog of Homo sapiens Deoxyribonuclease [illegible]

39112	3.37	0.51	0.0002	0.0062	3.40	all_v2.0.1343 .C1	Mitochondrial import inner membrane translocase subunit Tim23; n=3; Otophysi|Rep: Mitochondrial import inner membrane translocase subunit Tim23 - Danio rerio (Zebrafish) (Brachydanio rerio)
51371	5.03	0.26	0.0000	0.0016	3.39	all_v2.0.5978 .C1	unclassified
36190	4.67	0.32	0.0000	0.0018	3.38	sb_gmapht_0 014i15.pDNR F2	Bloodthirsty; n=2; Notothenioidei|Rep: Bloodthirsty - Chaenocephalus aceratus (White crocodile fish)
52069	4.25	0.33	0.0000	0.0023	3.36	all_v2.0.6954 .C2	unclassified
54933	4.33	0.34	0.0000	0.0021	3.35	all_v2.0.5297 .C2	unclassified
41902	3.31	0.60	0.0003	0.0070	3.32	all_v2.0.1081 .C6	Ribosomal protein L9e; n=4; Endopterygota|Rep: Ribosomal protein L9e - Meladema coriacea
38552	6.74	0.17	0.0000	0.0013	3.23	all_v2.0.1004 6.C1	ilvB (bacterial acetolactate synthase)-like; n=1; Takifugu rubripes|Rep: ilvB (bacterial acetolactate synthase)-like - Takifugu rubripes
41153	3.12	0.41	0.0004	0.0096	3.21	all_v2.0.7408 .C1	PREDICTED: similar to transposase; n=2; Strongylocentrotus purpuratus|Rep: PREDICTED: similar to transposase - Strongylocentrotus purpuratus
36195	5.46	0.23	0.0000	0.0015	3.16	sb_gmnlbfic_ 0007a23.t7	Bloodthirsty; n=2; Notothenioidei|Rep: Bloodthirsty - Chaenocephalus aceratus (White crocodile fish)
48085	3.46	0.51	0.0002	0.0055	3.14	sb_gmnlla_00 19f11.t7	Unassigned protein
42925	2.78	0.49	0.0011	0.0169	3.12	all_v2.0.9463 .C1	Ubiquitin-conjugating enzyme E2 D2 (EC 6.3.2.19) (Ubiquitin-protein ligase D2) (Ubiquitin carrier protein D2) (Ubiquitin-conjugating enzyme E2-17 kDa 2) (E2(17)KB 2); n=32; Euteleostomi|Rep: Ubiquitin-conjugating enzyme E2 D2 (EC 6.3.2.19) (Ubiquitin-protein ligase D2) (Ubiquitin carrier protein D2) (Ubiquitin-conjugating enzyme E2-17 kDa 2) (E2(17)KB 2) - Homo sapiens (Human)
44624	3.33	0.45	0.0003	0.0067	3.12	Codviperin1	custom - viperin
48451	5.15	0.24	0.0000	0.0016	3.10	sb_gmnlsfic_ 0001c01.t7	Unassigned protein
38618	3.08	0.42	0.0005	0.0101	3.10	all_v2.0.3462 .C1	Interferon-inducible GTPase 5 (EC 3.6.5.-) (Immunity-related GTPase cinema 1).; n=3; Takifugu rubripes|Rep: Interferon-inducible GTPase 5 (EC 3.6.5.-) (Immunity-related GTPase cinema 1). - Takifugu rubripes
45684	2.61	0.55	0.0018	0.0222	3.09	all_v2.0.6038 .C1.a	non-directed
44336	5.29	0.24	0.0000	0.0015	3.08	sb_gmapht_0 037b08.pDN RF2	bloodthirsty [Notothenia coriiceps]
49575	4.41	0.27	0.0000	0.0020	3.07	all_v2.0.3263 .C1	unclassified
47485	4.09	0.33	0.0001	0.0025	3.06	all_v2.0.1027 6.C1	Unassigned protein
51818	4.65	0.25	0.0000	0.0018	3.02	all_v2.0.1145 5.C1	unclassified
45917	6.01	0.19	0.0000	0.0014	3.01	all_v2.0.132. C2.a	non-directed
49008	3.29	0.37	0.0003	0.0072	3.00	sb_gmnlla_00 02h23.t7	Unassigned protein
37904	3.03	0.57	0.0006	0.0109	2.98	sb_gmnbbrts_ 0003k13.pDN RF2	Homolog of Gallus gallus Double-stranded RNA-activated protein kinase.; n=1; Takifugu rubripes|Rep: Homolog of Gallus gallus Double-stranded RNA-activated protein kinase. - Takifugu rubripes
41730	4.74	0.25	0.0000	0.0018	2.98	sb_gmapov_0 017j04.pDNR m13r	Regulatory-associated protein of mTOR (Raptor) (P150 target of rapamycin (TOR)-scaffold protein); n=24; Tetrapoda|Rep: Regulatory-associated protein of mTOR (Raptor) (P150 target of rapamycin (TOR)-scaffold protein) - Homo

							sapiens (Human)
50715	4.93	0.23	0.0000	0.0017	2.96	all_v2.0.4215.C1	unclassified
42748	6.41	0.16	0.0000	0.0013	2.96	all_v2.0.9596.C1	Transmembrane protein 7; n=1; Siniperca chuatsi|Rep: Transmembrane protein 7 - Siniperca chuatsi (Chinese perch)
40875	3.01	0.39	0.0006	0.0116	2.95	sb_gmnbhkic_0015m19.pDNRm13r	PREDICTED: similar to protein BAP28 (FLJ10359); n=1; Monodelphis domestica|Rep: PREDICTED: similar to protein BAP28 (FLJ10359) - Monodelphis domestica
53251	3.76	0.31	0.0001	0.0038	2.95	all_v2.0.13404.C1	unclassified
37289	5.61	0.20	0.0000	0.0014	2.94	sb_gmnbhkas_0019c20.pDNRF2	G protein-coupled receptor 155; n=1; Takifugu rubripes|Rep: G protein-coupled receptor 155 - Fugu rubripes (Japanese pufferfish) (Takifugu rubripes)
36193	2.52	0.74	0.0024	0.0258	2.92	all_v2.0.661.C39	Bloodthirsty; n=2; Notothenioidei|Rep: Bloodthirsty - Chaenocephalus aceratus (White crocodile fish)
47613	4.66	0.27	0.0000	0.0018	2.92	sb_gmnbbr_0013m15.pDNRm13r	Unassigned protein
45753	3.55	0.33	0.0002	0.0049	2.91	all_v2.0.4658.C1.a	non-directed
36185	6.08	0.17	0.0000	0.0013	2.91	all_v2.0.661.C20	Bloodthirsty; n=2; Notothenioidei|Rep: Bloodthirsty - Chaenocephalus aceratus (White crocodile fish)
40809	5.71	0.18	0.0000	0.0014	2.91	sb_gmnbpcic_0012f05.pDNRm13r	PREDICTED: similar to pol polyprotein; n=1; Danio rerio|Rep: PREDICTED: similar to pol polyprotein - Danio rerio
37392	2.99	0.53	0.0006	0.0119	2.90	all_v2.0.5847.C1	Glutaredoxin-1 (Thioltransferase-1) (TTase-1).; n=1; Takifugu rubripes|Rep: Glutaredoxin-1 (Thioltransferase-1) (TTase-1). - Takifugu rubripes
55096	3.04	0.41	0.0005	0.0108	2.87	all_v2.0.1736.C2	unclassified
41397	4.18	0.27	0.0001	0.0024	2.86	all_v2.0.847.C1	Prostaglandin E synthase 3; n=5; Danio rerio|Rep: Prostaglandin E synthase 3 - Danio rerio (Zebrafish) (Brachydanio rerio)
43888	5.46	0.19	0.0000	0.0015	2.86	all_v2.0.15537.C1	Zgc:64031; n=3; Danio rerio|Rep: Zgc:64031 - Danio rerio (Zebrafish) (Brachydanio rerio)
44544	5.40	0.20	0.0000	0.0015	2.85	IRF1OK2	custom - Interferon regulatory factor 10K
45747	2.62	0.41	0.0018	0.0216	2.84	all_v2.0.16019.C1.a	non-directed
38352	4.48	0.24	0.0000	0.0019	2.84	all_v2.0.272.C2	Homolog of Homo sapiens SOUL protein; n=1; Takifugu rubripes|Rep: Homolog of Homo sapiens SOUL protein - Takifugu rubripes
48129	3.66	0.31	0.0001	0.0042	2.83	all_v2.0.14003.C1	Unassigned protein
40489	3.40	0.39	0.0002	0.0060	2.80	sb_gmnbbr_0003e16.pDNRF2	PREDICTED: similar to G protein-coupled receptor 97; n=2; Canis familiaris|Rep: PREDICTED: similar to G protein-coupled receptor 97 - Canis familiaris
41014	6.71	0.14	0.0000	0.0013	2.78	all_v2.0.14158.C1	PREDICTED: similar to Snf2-related CBP activator protein; n=1; Mus musculus|Rep: PREDICTED: similar to Snf2-related CBP activator protein - Mus musculus
43192	6.07	0.16	0.0000	0.0013	2.78	sb_gmnlbric_0006j07.t7	Vesicular glutamate transporter 2.2; n=3; Danio rerio|Rep: Vesicular glutamate transporter 2.2 - Danio rerio (Zebrafish) (Brachydanio rerio)
44579	4.15	0.31	0.0001	0.0025	2.76	3MKK4_MAP2K3	custom - Mitogen-activated protein kinase kinase 4
49851	3.20	0.44	0.0004	0.0084	2.75	all_v2.0.2837.C3	unclassified
41829	5.40	0.19	0.0000	0.0015	2.74	sb_gmnblits_0018p08.pDNRF2	RFK protein; n=2; Bos taurus|Rep: RFK protein - Bos taurus (Bovine)
38589	3.12	0.39	0.0004	0.0095	2.74	sb_gmnbpcic_0005b07.pDNRF2	Integrin alpha-2 precursor (Platelet membrane glycoprotein Ia) (GPIa) (Collagen receptor) (VLA-2 alpha chain) (CD49b antigen).; n=1; Takifugu

38589	3.12	0.39	0.0004	0.0095	2.74	sb_gmnbpcic_0005b07.pDNRF2	Integrin alpha-2 precursor (Platelet membrane glycoprotein Ia) (GPIa) (Collagen receptor) (VLA-2 alpha chain) (CD49b antigen).; n=1; Takifugu rubripes|Rep: Integrin alpha-2 precursor (Platelet membrane glycoprotein Ia) (GPIa) (Collagen receptor) (VLA-2 alpha chain) (CD49b antigen). - Takifugu rubripes
41103	5.29	0.20	0.0000	0.0015	2.73	sb_gmnbhkic_0006n17.pDNRF2	PREDICTED: similar to transposase, partial; n=1; Strongylocentrotus purpuratus|Rep: PREDICTED: similar to transposase, partial - Strongylocentrotus purpuratus
44454	2.85	0.47	0.0009	0.0149	2.72	1AY614590_Gadus morhua CC chemokine type 1 mRNA, complete cds	custom - CC chemokine
52032	2.57	0.68	0.0020	0.0237	2.70	all_v2.0.5496.C1	unclassified
46452	4.83	0.21	0.0000	0.0017	2.68	all_v2.0.3031.C1	Unassigned protein
44585	4.97	0.21	0.0000	0.0016	2.67	DXH2a	custom - probable ATP-dependent RNA helicase DHX
39325	5.43	0.19	0.0000	0.0015	2.66	all_v2.0.9595.C2	NFX1-type zinc finger-containing protein 1.; n=1; Takifugu rubripes|Rep: NFX1-type zinc finger-containing protein 1 - Takifugu rubripes
44470	2.65	0.50	0.0016	0.0209	2.66	CK2c1d	custom - CC chemokine
55231	3.88	0.28	0.0001	0.0033	2.66	all_v2.0.8766.C1	unclassified
42946	2.51	0.88	0.0025	0.0261	2.64	all_v2.0.4194.C1	UDP-glucuronic acid/UDP-N-acetylgalactosamine transporter (UDP- GlcA/UDP-GalNAc transporter) (Solute carrier family 35 member D1) (UDP-galactose transporter-related protein 7) (UGTrel7).; n=1; Takifugu rubripes|Rep: UDP-glucuronic acid/UDP-N-acetylgalactosamine transporter (UDP- GlcA/UDP-GalNAc transporter) (Solute carrier family 35 member D1) (UDP-galactose transporter-related protein 7) (UGTrel7). - Takifugu rubripes
52606	6.03	0.15	0.0000	0.0013	2.64	all_v2.0.5996.C2	unclassified
55108	4.03	0.26	0.0001	0.0027	2.64	all_v2.0.8631.C1	unclassified
40930	3.52	0.37	0.0002	0.0051	2.64	sb_gmnbspic_0012b01.pDNRF2	PREDICTED: similar to retinoblastoma-associated protein 140; n=2; Canis lupus familiaris|Rep: PREDICTED: similar to retinoblastoma-associated protein 140 - Canis familiaris
47156	4.14	0.28	0.0001	0.0025	2.63	all_v2.0.617.C5	Unassigned protein
38353	4.10	0.25	0.0001	0.0025	2.63	all_v2.0.415.C2	Homolog of Homo sapiens Spastic ataxia of Charlevoix-Saguenay; n=1; Takifugu rubripes|Rep: Homolog of Homo sapiens Spastic ataxia of Charlevoix-Saguenay - Takifugu rubripes
51062	6.72	0.13	0.0000	0.0013	2.63	all_v2.0.8508.C1	unclassified
38599	4.79	0.20	0.0000	0.0017	2.63	all_v2.0.16145.C1	Interferon regulatory factor 10; n=7; Danio rerio|Rep: Interferon regulatory factor 10 - Danio rerio (Zebrafish) (Brachydanio rerio)
41827	6.16	0.15	0.0000	0.0013	2.62	sb_gmapov_0017j24.pDNRF2	Rfc5 protein; n=5; Clupeocephala|Rep: Rfc5 protein - Brachydanio rerio (Zebrafish) (Danio rerio)
48344	3.06	0.34	0.0005	0.0105	2.59	sb_gmnlkfas_0003n17.t7	Unassigned protein
53056	3.25	0.34	0.0003	0.0077	2.58	all_v2.0.4192.C1	unclassified
51636	4.64	0.23	0.0000	0.0018	2.58	all_v2.0.1410.C1	unclassified
							phosphatidylinositol-specific phospholipase C, X

40846	2.96	0.43	0.0007	0.0123	2.57	all_v2.0.4119 .C1	PREDICTED: similar to Poly [ADP-ribose] polymerase 14 (PARP-14) (B aggressive lymphoma protein 2); n=1; Danio rerio|Rep: PREDICTED: similar to Poly [ADP-ribose] polymerase 14 (PARP-14) (B aggressive lymphoma protein 2) - Danio rerio
46920	3.44	0.36	0.0002	0.0057	2.57	sb_gmnlla_00 48f01.t7	Unassigned protein
44465	2.41	0.54	0.0033	0.0314	2.55	1AY614591_ Gadus morhua CC chemokine type 2 mRNA, complete cds	custom - CC chemokine
46358	3.73	0.26	0.0001	0.0038	2.51	all_v2.0.7414 .C2	TNIP3, LOC483847; TNFAIP3 interacting protein 3
40216	4.12	0.25	0.0001	0.0025	2.51	sb_gmapte_0 010k12.pDN Rm13r	PREDICTED: similar to ankyrin 2, neuronal,; n=1; Danio rerio|Rep: PREDICTED: similar to ankyrin 2, neuronal, - Danio rerio
44456	2.73	0.44	0.0013	0.0182	2.50	CK2c5	custom - CC chemokine
38655	4.21	0.23	0.0000	0.0023	2.49	all_v2.0.1909 .C3	IRF7; n=1; Siniperca chuatsi|Rep: IRF7 - Siniperca chuatsi (Chinese perch)
51817	2.69	0.39	0.0015	0.0197	2.47	all_v2.0.4419 .C1	unclassified
44545	4.30	0.21	0.0000	0.0021	2.46	IRF1OK3	custom - Interferon regulatory factor 10K
48091	2.45	0.58	0.0029	0.0288	2.46	all_v2.0.1469 6.C1	Unassigned protein
55397	4.71	0.20	0.0000	0.0018	2.46	all_v2.0.1490 0.C1	unclassified
40905	2.95	0.32	0.0007	0.0126	2.45	sb_gmnlskic_ 0005p12.pDN RF2	PREDICTED: similar to putative pol protein; n=1; Danio rerio|Rep: PREDICTED: similar to putative pol protein - Danio rerio
51340	4.54	0.20	0.0000	0.0019	2.44	all_v2.0.1299 5.C1	unclassified
38094	3.02	0.40	0.0006	0.0112	2.44	sb_gmnbhkic _0005c22.pD NRF2	Homolog of Homo sapiens HECT domain and RCC1-like domain protein 3; n=1; Takifugu rubripes|Rep: Homolog of Homo sapiens HECT domain and RCC1-like domain protein 3 - Takifugu rubripes
36194	5.59	0.15	0.0000	0.0014	2.43	all_v2.0.1204 9.C1	Bloodthirsty; n=2; Notothenioidei|Rep: Bloodthirsty - Chaenocephalus aceratus (White crocodile fish)
54821	3.09	0.36	0.0005	0.0099	2.43	all_v2.0.1066 8.C2	unclassified
44463	2.78	0.40	0.0011	0.0169	2.43	CK2c4	custom - CC chemokine
37060	4.52	0.21	0.0000	0.0019	2.43	sb_gmnlsras_ 0003k23.t7	Eph-like receptor tyrosine kinase rtk5; n=4; Danio rerio|Rep: Eph-like receptor tyrosine kinase rtk5 - Danio rerio (Zebrafish) (Brachydanio rerio)
47039	2.64	0.48	0.0017	0.0214	2.42	sb_gmnbhkas _0001d21.pD NRF2	Unassigned protein
44446	2.50	0.49	0.0025	0.0264	2.42	CK2c3	custom - CC chemokine
53530	2.47	0.63	0.0028	0.0282	2.42	all_v2.0.498. C1	unclassified
40019	3.69	0.28	0.0001	0.0040	2.42	sb_gmnbhkas _0006d14.pD NRF2	Pol polyprotein; n=2; Walleye epidermal hyperplasia virus 2|Rep: Pol polyprotein - Walleye epidermal hyperplasia virus 2
45204	2.63	0.45	0.0018	0.0216	2.40	all_v2.0.1178 5.C1	LOC575702; similar to reverse transcriptase-like protein
44587	3.76	0.27	0.0001	0.0038	2.40	DXH3a	custom - probable ATP-dependent RNA helicase DHX
36317	4.28	0.21	0.0000	0.0022	2.39	all_v2.0.1528 0.C1	Carnitine palmitoyltransferase II; n=2; Danio rerio|Rep: Carnitine palmitoyltransferase II - Danio rerio (Zebrafish) (Brachydanio rerio)
38788	4.63	0.19	0.0000	0.0018	2.39	all_v2.0.1155 .C2	Lily-type lectin; n=1; Platycephalus indicus|Rep: Lily-type lectin - Platycephalus indicus (bartail flathead)

43362	2.68	0.49	0.0015	0.0197	2.39	sb_gmnbmu_0012l15.pDNRF2	Zgc:103426; n=2; Danio rerio|Rep: Zgc:103426 - Danio rerio (Zebrafish) (Brachydanio rerio)
53587	5.58	0.14	0.0000	0.0014	2.38	all_v2.0.7025.C2	unclassified
55314	2.78	0.46	0.0011	0.0171	2.37	all_v2.0.4690.C2	unclassified
53739	3.93	0.24	0.0001	0.0030	2.37	all_v2.0.354.C2	unclassified
53350	3.31	0.32	0.0003	0.0070	2.36	all_v2.0.16720.C1	unclassified
38670	4.47	0.20	0.0000	0.0019	2.36	all_v2.0.3891.C1	Isoform 2 of Q5RI56 ; n=2; Danio rerio|Rep: Isoform 2 of Q5RI56 - Danio rerio (Zebrafish) (Brachydanio rerio)
46707	3.45	0.31	0.0002	0.0056	2.36	sb_gmnbhkic_0016o08.pDNRm13r	Unassigned protein
44283	4.05	0.22	0.0001	0.0026	2.35	sb_gmnlmfta_0004i04.t7	ARL6IP6; ADP-ribosylation-like factor 6 interacting protein 6
43481	3.32	0.27	0.0003	0.0069	2.33	all_v2.0.17274.C1	Zgc:112001; n=1; Danio rerio|Rep: Zgc:112001 - Danio rerio (Zebrafish) (Brachydanio rerio)
45046	4.73	0.17	0.0000	0.0018	2.32	sb_gmapht_0020j08.pDNRF2	LOC420518; similar to putative tyrosine phosphatase
39326	3.11	0.34	0.0004	0.0097	2.32	all_v2.0.9595.C1	NFX1-type zinc finger-containing protein 1.; n=1; Takifugu rubripes|Rep: NFX1-type zinc finger-containing protein 1 - Takifugu rubripes
55269	4.60	0.18	0.0000	0.0018	2.31	all_v2.0.9958.C1	unclassified
49081	8.52	0.06	0.0000	0.0013	2.31	all_v2.0.4460.C1	unclassified
36177	3.48	0.31	0.0002	0.0054	2.29	sb_gmnlem_0010g13.t7	Birc4 protein; n=7; Danio rerio|Rep: Birc4 protein - Danio rerio (Zebrafish) (Brachydanio rerio)
36962	4.35	0.19	0.0000	0.0021	2.29	all_v2.0.12651.C1	Dysbindin; n=4; Danio rerio|Rep: Dysbindin - Danio rerio (Zebrafish) (Brachydanio rerio)
41393	4.56	0.18	0.0000	0.0018	2.28	sb_gmnbhkic_0014b13.pDNRF2	Proprotein convertase subtilisin/kexin type 5 precursor (EC 3.4.21.-) (Proprotein convertase PC5) (Subtilisin/kexin-like protease PC5) (PC6) (hPC6).; n=1; Takifugu rubripes|Rep: Proprotein convertase subtilisin/kexin type 5 precursor (EC 3.4.21.-) (Proprotein convertase PC5) (Subtilisin/kexin-like protease PC5) (PC6) (hPC6). - Takifugu rubripes
46223	4.80	0.17	0.0000	0.0017	2.27	all_v2.0.5976.C1	SERINC3; serine incorporator 3
37299	2.49	0.34	0.0026	0.0268	2.27	all_v2.0.3679.C1	Gadd45al protein; n=2; Danio rerio|Rep: Gadd45al protein - Danio rerio (Zebrafish) (Brachydanio rerio)
48214	6.70	0.10	0.0000	0.0013	2.27	sb_gmnbhkas_0014f23.pDNRm13r	Unassigned protein
38444	4.15	0.20	0.0001	0.0025	2.26	all_v2.0.10457.C1	Homolog of Homo sapiens Zinc finger CCCH type domain containing protein 1; n=2; Takifugu rubripes|Rep: Homolog of Homo sapiens Zinc finger CCCH type domain containing protein 1 - Takifugu rubripes
44547	3.80	0.24	0.0001	0.0036	2.26	IRF1OK5	custom - Interferon regulatory factor 10K
36797	2.86	0.39	0.0009	0.0145	2.26	all_v2.0.2166.C2	Cytotoxic and regulatory T cell protein; n=1; Oncorhynchus mykiss|Rep: Cytotoxic and regulatory T cell protein - Oncorhynchus mykiss (Rainbow trout) (Salmo gairdneri)
42375	4.80	0.17	0.0000	0.0017	2.25	sb_gmnbspic_0005a23.pDNRF2	SRY-box containing gene 11a; n=1; Gnathonemus petersii|Rep: SRY-box containing gene 11a - Gnathonemus petersii
47376	2.90	0.35	0.0008	0.0136	2.25	all_v2.0.15415.C1	Unassigned protein
47560	3.24	0.32	0.0003	0.0077	2.25	sb_gmapte_0001n01.pDNRm13r	Unassigned protein

38339	4.48	0.19	0.0000	0.0019	2.25	sb_gmapov_0019k11.pDNRF2	Homolog of Homo sapiens Sodium- and chloride-dependent GABA transporter 3; n=2; Takifugu rubripes|Rep: Homolog of Homo sapiens Sodium- and chloride-dependent GABA transporter 3 - Takifugu rubripes
39119	4.78	0.17	0.0000	0.0017	2.25	all_v2.0.5993.C1	Mitochondrial Rho GTPase 1-A; n=1; Danio rerio|Rep: Mitochondrial Rho GTPase 1-A - Danio rerio (Zebrafish) (Brachydanio rerio)
50297	5.48	0.13	0.0000	0.0014	2.24	all_v2.0.12538.C1	unclassified
39763	2.67	0.44	0.0015	0.0202	2.23	all_v2.0.3451.C1	Nucleoporin GLE1; n=1; Bos taurus|Rep: Nucleoporin GLE1 - Bos taurus (Bovine)
54819	3.81	0.22	0.0001	0.0036	2.23	all_v2.0.14839.C1	unclassified
41096	3.34	0.26	0.0003	0.0067	2.23	all_v2.0.1554.C7	PREDICTED: similar to transposase (putative); n=1; Danio rerio|Rep: PREDICTED: similar to transposase (putative) - Danio rerio
49072	2.83	0.35	0.0010	0.0153	2.23	all_v2.0.16355.C1	unclassified
50117	2.83	0.34	0.0010	0.0154	2.22	all_v2.0.6258.C1	unclassified
42842	5.52	0.13	0.0000	0.0014	2.22	sb_gmnlmfta_0005n11.t7	Twinfilin-2; n=2; Danio rerio|Rep: Twinfilin-2 - Danio rerio (Zebrafish) (Brachydanio rerio)
52909	2.54	0.58	0.0022	0.0248	2.22	all_v2.0.7059.C2	unclassified
48479	4.10	0.19	0.0001	0.0025	2.21	all_v2.0.6321.C4	Unassigned protein
41761	2.81	0.30	0.0010	0.0162	2.21	sb_gmapht_0030b14.pDNRm13r	Reverse transcriptase; n=1; Anguilla japonica|Rep: Reverse transcriptase - Anguilla japonica (Japanese eel)
44459	2.78	0.34	0.0011	0.0169	2.20	CK2c5d	custom - CC chemokine
44532	2.86	0.35	0.0009	0.0145	2.19	IKKAlikeS3	custom - IKKA-like protein
36994	3.52	0.22	0.0002	0.0051	2.19	sb_gmnlkfta_0002p12.t7	EIF4G-related protein NAT1A; n=4; Danio rerio|Rep: EIF4G-related protein NAT1A - Danio rerio (Zebrafish) (Brachydanio rerio)
49711	3.12	0.30	0.0004	0.0095	2.18	all_v2.0.15207.C1	unclassified
44458	2.65	0.38	0.0016	0.0207	2.18	CK2c1	custom - CC chemokine
45629	2.63	0.44	0.0017	0.0215	2.18	all_v2.0.9479.C1	non-directed
50482	2.78	0.40	0.0011	0.0169	2.17	all_v2.0.9590.C1	unclassified
51382	2.72	0.32	0.0013	0.0187	2.17	all_v2.0.5897.C3	unclassified
40670	3.04	0.32	0.0005	0.0108	2.17	all_v2.0.14446.C1	PREDICTED: similar to mitochondrial carrier triple repeat 6; n=1; Monodelphis domestica|Rep: PREDICTED: similar to mitochondrial carrier triple repeat 6 - Monodelphis domestica
40263	4.46	0.18	0.0000	0.0020	2.16	all_v2.0.661.C24	PREDICTED: similar to bloodthirsty; n=3; Danio rerio|Rep: PREDICTED: similar to bloodthirsty - Danio rerio
44396	5.04	0.14	0.0000	0.0016	2.15	all_v2.0.6100.C1	CHCHD5; coiled-coil-helix-coiled-coil-helix domain containing 5
44530	3.01	0.26	0.0006	0.0115	2.15	IKKAlikeS1	custom - IKKA-like protein
43195	4.36	0.17	0.0000	0.0021	2.14	all_v2.0.3448.C1	VHSV-induced protein; n=1; Oncorhynchus mykiss|Rep: VHSV-induced protein - Oncorhynchus mykiss (Rainbow trout) (Salmo gairdneri)
42591	3.15	0.24	0.0004	0.0090	2.14	sb_gmapht_0021e11.pDNRF2	Titin; n=2; Danio rerio|Rep: Titin - Brachydanio rerio (Zebrafish) (Danio rerio)
55171	4.22	0.19	0.0000	0.0023	2.14	all_v2.0.14663.C1	unclassified
46402	3.37	0.25	0.0002	0.0063	2.14	sb_gmapht_0004b19.pDNRm13r	TTN; cardiac titin
41595	2.76	0.37	0.0012	0.0177	2.13	sb_gmapov_0028l22.pDNR	PRP4 pre-mRNA processing factor 4 homolog; n=2; Danio rerio|Rep: PRP4 pre-mRNA processing

41595	2.76	0.37	0.0012	0.0177	2.13	sb_gmapov_0028l22.pDNRF2	PRP4 pre-mRNA processing factor 4 homolog; n=2; Danio rerio|Rep: PRP4 pre-mRNA processing factor 4 homolog - Brachydanio rerio (Zebrafish) (Danio rerio)
54239	3.01	0.27	0.0006	0.0116	2.12	all_v2.0.11344.C1	unclassified
54855	2.58	0.45	0.0020	0.0233	2.12	all_v2.0.8904.C1	unclassified
38625	2.71	0.32	0.0014	0.0189	2.12	all_v2.0.4114.C1	Interleukin 12 receptor beta 2.b; n=1; Danio rerio|Rep: Interleukin 12 receptor beta 2.b - Danio rerio (Zebrafish) (Brachydanio rerio)
52906	2.78	0.34	0.0011	0.0169	2.12	all_v2.0.2709.C1	unclassified
39291	5.45	0.12	0.0000	0.0015	2.12	sb_gmnbmu_0014b24.pDNRF2	Nebulin.; n=1; Takifugu rubripes|Rep: Nebulin. - Takifugu rubripes
40721	4.20	0.18	0.0000	0.0024	2.12	sb_gmnbpcic_0001i03.pDNRF2	PREDICTED: similar to Nitric oxide synthase trafficker; n=1; Danio rerio|Rep: PREDICTED: similar to Nitric oxide synthase trafficker - Danio rerio
44117	2.73	0.30	0.0013	0.0181	2.11	sb_gmnbgi_0002m22.pDNRF2	Zinc finger CCCH type domain containing protein 1.; n=1; Xenopus tropicalis|Rep: Zinc finger CCCH type domain containing protein 1 - Xenopus tropicalis
41562	5.24	0.13	0.0000	0.0016	2.10	sb_gmnlmfta_0004n10.t7	Protein-tyrosine phosphatase-like member A; n=9; Eutheria|Rep: Protein-tyrosine phosphatase-like member A - Homo sapiens (Human)
48574	2.96	0.29	0.0007	0.0123	2.09	sb_gmnbhkas_0007c06.pDNRF2	Unassigned protein
52428	4.52	0.15	0.0000	0.0019	2.08	all_v2.0.14177.C1	unclassified
40667	2.91	0.31	0.0008	0.0135	2.08	all_v2.0.431.C8	PREDICTED: similar to microtubule aggregate protein homolog; n=2; Danio rerio|Rep: PREDICTED: similar to microtubule aggregate protein homolog - Danio rerio
41317	5.09	0.13	0.0000	0.0016	2.08	all_v2.0.1165.C4	Probable ATP-dependent helicase LGP2 (EC 3.6.1.-) (Protein D11Lgp2 homolog).; n=1; Takifugu rubripes|Rep: Probable ATP-dependent helicase LGP2 (EC 3.6.1.-) (Protein D11Lgp2 homolog). - Takifugu rubripes
37073	2.84	0.30	0.0009	0.0150	2.08	sb_gmnbspic_0005b03.pDNRF2	EPSTI1 protein; n=3; Bos taurus|Rep: EPSTI1 protein - Bos taurus (Bovine)
46192	3.77	0.21	0.0001	0.0037	2.08	all_v2.0.8585.C1	RVT_1 domain containing protein
48258	2.69	0.37	0.0015	0.0197	2.07	all_v2.0.14926.C1	Unassigned protein
45976	3.85	0.19	0.0001	0.0034	2.06	sb_gmnlkfta_0007d22.t7	pbef1; pre-B-cell colony enhancing factor 1
54223	2.92	0.32	0.0007	0.0132	2.06	all_v2.0.6859.C3	unclassified
54489	2.42	0.30	0.0032	0.0309	2.06	all_v2.0.776.C1	unclassified
36201	2.97	0.26	0.0007	0.0123	2.05	all_v2.0.1758.C1	Blue-sensitive pigment; n=1; Gadus morhua|Rep: Blue-sensitive pigment - Gadus morhua (Atlantic cod)
36625	3.28	0.27	0.0003	0.0073	2.05	sb_gmnbpcic_0006c06.pDNRm13r	Complement component C9; n=1; Paralichthys olivaceus|Rep: Complement component C9 - Paralichthys olivaceus (Japanese flounder)
36407	2.57	0.42	0.0021	0.0237	2.05	all_v2.0.1864.C7	CD9 protein; n=1; Oncorhynchus mykiss|Rep: CD9 protein - Oncorhynchus mykiss (Rainbow trout) (Salmo gairdneri)
42585	4.13	0.18	0.0001	0.0025	2.05	all_v2.0.2335.C1	Titin a; n=3; Danio rerio|Rep: Titin a - Danio rerio (Zebrafish) (Brachydanio rerio)
37226	2.92	0.26	0.0008	0.0132	2.04	all_v2.0.556.C2	Fibroblast growth factor (Acidic) intracellular binding protein; n=2; Danio rerio|Rep: Fibroblast growth factor (Acidic) intracellular binding protein - Danio rerio (Zebrafish) (Brachydanio rerio)

51314	2.47	0.35	0.0027	0.0277	2.04	all_v2.0.6744.C1	unclassified
54924	2.93	0.30	0.0007	0.0130	2.04	all_v2.0.12344.C1	unclassified
37698	4.95	0.13	0.0000	0.0016	2.04	sb_gmnbpcic_0001e04.pDNRm13r	Homolog of Brachydanio rerio Cyclin L ania-6a.; n=2; Takifugu rubripes|Rep: Homolog of Brachydanio rerio Cyclin L ania-6a. - Takifugu rubripes
39363	3.80	0.18	0.0001	0.0036	2.03	all_v2.0.17706.C1	Novel gene; n=1; Danio rerio|Rep: Novel gene - Danio rerio
39371	2.89	0.25	0.0008	0.0138	2.03	sb_gmnbhkas_0007i12.pDNRF2	Novel immune-type receptor 4; n=2; Oncorhynchus mykiss|Rep: Novel immune-type receptor 4 - Oncorhynchus mykiss (Rainbow trout) (Salmo gairdneri)
45510	2.76	0.36	0.0012	0.0176	2.03	all_v2.0.18479.C1.a	non-directed
49472	3.73	0.19	0.0001	0.0038	2.03	all_v2.0.12909.C1	unclassified
44615	3.41	0.21	0.0002	0.0060	2.03	TLR9Cend	custom - toll-like receptor 9
46309	4.08	0.17	0.0001	0.0026	2.02	sb_gmapht_0002g19.pDNRF2	TatC domain containing protein
43354	2.55	0.35	0.0022	0.0245	2.02	all_v2.0.772.C1	Zgc:101851; n=2; Danio rerio|Rep: Zgc:101851 - Danio rerio (Zebrafish) (Brachydanio rerio)
40018	5.35	0.11	0.0000	0.0015	2.01	all_v2.0.7260.C1	Pol polyprotein; n=1; Nosema bombycis|Rep: Pol polyprotein - Nosema bombycis
51588	3.32	0.22	0.0003	0.0069	2.01	all_v2.0.14701.C1	unclassified
51377	2.88	0.28	0.0008	0.0140	2.01	all_v2.0.2972.C1	unclassified
37854	2.94	0.25	0.0007	0.0127	2.01	sb_gmnlsfic_0002b12.t7	Homolog of Carassius auratus Interferon-inducible protein Gig2.; n=4; Takifugu rubripes|Rep: Homolog of Carassius auratus Interferon-inducible protein Gig2. - Takifugu rubripes
50391	2.53	0.28	0.0023	0.0253	2.01	all_v2.0.2069.C2	unclassified
55249	3.69	0.20	0.0001	0.0040	2.00	all_v2.0.3039.C5	unclassified
40091	2.92	0.27	0.0007	0.0131	2.00	sb_gmapov_0008b19.pDNRF2	Polyribonucleotide nucleotidyltransferase 1, mitochondrial precursor; n=18; Euarchontoglires|Rep: Polyribonucleotide nucleotidyltransferase 1, mitochondrial precursor - Homo sapiens (Human)
43990	2.54	0.41	0.0023	0.0250	2.00	sb_gmnbpcic_0010b03.pDNRm13r	Zgc:85932; n=5; Danio rerio|Rep: Zgc:85932 - Danio rerio (Zebrafish) (Brachydanio rerio)
53347	2.96	0.26	0.0007	0.0123	1.99	all_v2.0.9197.C1	unclassified
44665	3.87	0.19	0.0001	0.0033	1.99	all_v2.0.13567.C1	Drf_FH1 multi-domain protein
48557	3.01	0.25	0.0006	0.0116	1.99	sb_gmnbspic_0009m06.pDNRm13r	Unassigned protein
45372	2.54	0.27	0.0022	0.0249	1.98	all_v2.0.433.C1	LYR motif-containing protein ENSP00000368165
44351	3.43	0.22	0.0002	0.0058	1.98	all_v2.0.693.C1	C2 domain containing protein
40902	4.30	0.15	0.0000	0.0021	1.98	sb_gmapov_0017b09.pDNRF2	PREDICTED: similar to purple acid phosphatase, putative, partial; n=2; Danio rerio|Rep: PREDICTED: similar to purple acid phosphatase, putative, partial - Danio rerio
42172	4.15	0.17	0.0001	0.0025	1.98	sb_gmnlla_0045h19.t7	Si:dkey-21k10.1 protein; n=3; Danio rerio|Rep: Si:dkey-21k10.1 protein - Danio rerio (Zebrafish) (Brachydanio rerio)
40238	4.42	0.14	0.0000	0.0020	1.98	all_v2.0.13256.C1	PREDICTED: similar to ATPase, histidine kinase-, DNA gyrase B-, and HSP90-like domain containing protein; n=2; Danio rerio|Rep: PREDICTED: similar to ATPase, histidine kinase-, DNA gyrase B-, and HSP90-like domain

40238	4.42	0.14	0.0000	0.0020	1.98	all_v2.0.13256.C1	PREDICTED: similar to ATPase, histidine kinase-, DNA gyrase B-, and HSP90-like domain containing protein; n=2; Danio rerio|Rep: PREDICTED: similar to ATPase, histidine kinase-, DNA gyrase B-, and HSP90-like domain containing protein - Danio rerio
36895	3.12	0.24	0.0004	0.0096	1.98	all_v2.0.11426.C2	DNA polymerase; n=2; Tetraodontidae|Rep: DNA polymerase - Tetraodon nigroviridis (Green puffer)
51058	2.46	0.37	0.0028	0.0284	1.98	all_v2.0.11781.C2	unclassified
38695	2.66	0.27	0.0016	0.0204	1.97	all_v2.0.7632.C1	K14 protein; n=1; Xenopus laevis|Rep: K14 protein - Xenopus laevis (African clawed frog)
53650	3.21	0.21	0.0003	0.0081	1.97	all_v2.0.17219.C1	unclassified
40456	2.62	0.36	0.0018	0.0216	1.97	all_v2.0.3858.C1	PREDICTED: similar to factor activating exoenzyme S; n=1; Ornithorhynchus anatinus|Rep: PREDICTED: similar to factor activating exoenzyme S - Ornithorhynchus anatinus
43897	3.98	0.17	0.0001	0.0028	1.96	all_v2.0.8033.C1	Zgc:64137; n=2; Danio rerio|Rep: Zgc:64137 - Danio rerio (Zebrafish) (Brachydanio rerio)
49652	2.66	0.30	0.0016	0.0206	1.95	all_v2.0.9970.C1	unclassified
43681	2.52	0.29	0.0024	0.0258	1.95	all_v2.0.6826.C2	Zgc:153440; n=3; Danio rerio|Rep: Zgc:153440 - Danio rerio (Zebrafish) (Brachydanio rerio)
45487	2.60	0.34	0.0019	0.0226	1.95	all_v2.0.11021.C1	non-directed
47368	2.90	0.30	0.0008	0.0136	1.95	sb_gmnlla_0036m01.t7	Unassigned protein
36189	5.17	0.11	0.0000	0.0016	1.95	all_v2.0.661.C31	Bloodthirsty; n=2; Notothenioidei|Rep: Bloodthirsty - Chaenocephalus aceratus (White crocodile fish)
52110	5.57	0.09	0.0000	0.0014	1.95	all_v2.0.8876.C1	unclassified
35877	3.51	0.19	0.0002	0.0052	1.94	all_v2.0.2896.C1	Alpha-2-macroglobulin; n=1; Sparus aurata|Rep: Alpha-2-macroglobulin - Sparus aurata (Gilthead sea bream)
39240	2.42	0.29	0.0032	0.0306	1.94	all_v2.0.11987.C1	N-acylneuraminate-9-phosphatase (EC 3.1.3.29) (Neu5Ac-9-Pase) (Haloacid dehalogenase-like hydrolase domain-containing protein 4).; n=1; Takifugu rubripes|Rep: N-acylneuraminate-9-phosphatase (EC 3.1.3.29) (Neu5Ac-9-Pase) (Haloacid dehalogenase-like hydrolase domain-containing protein 4). - Takifugu rubripes
43876	2.42	0.38	0.0032	0.0306	1.93	all_v2.0.1930.C13	Zgc:63602; n=2; Danio rerio|Rep: Zgc:63602 - Danio rerio (Zebrafish) (Brachydanio rerio)
38330	3.16	0.21	0.0004	0.0090	1.93	sb_gmnlsfas_0002f20.t7	Homolog of Homo sapiens Similar to SH3-containing protein p4015; n=1; Takifugu rubripes|Rep: Homolog of Homo sapiens Similar to SH3-containing protein p4015 - Takifugu rubripes
48640	3.54	0.20	0.0002	0.0049	1.93	sb_gmnlla_0018p21.t7	Unassigned protein
47411	4.37	0.14	0.0000	0.0020	1.92	sb_gmnlskic_0010l20.pDNRm13r	Unassigned protein
41894	4.25	0.15	0.0000	0.0023	1.92	all_v2.0.2051.C1	Ribosomal protein L32; n=5; Euteleostei|Rep: Ribosomal protein L32 - Oncorhynchus masou formosanus
49387	2.53	0.29	0.0023	0.0254	1.91	all_v2.0.12515.C1	unclassified
43767	3.47	0.18	0.0002	0.0055	1.91	all_v2.0.8336.C1	Zgc:158652 protein; n=3; Danio rerio|Rep: Zgc:158652 protein - Danio rerio (Zebrafish) (Brachydanio rerio)
36552	2.85	0.23	0.0009	0.0149	1.90	all_v2.0.1104.C1	Cofilin-1-A; n=2; Xenopus laevis|Rep: Cofilin-1-A - Xenopus laevis (African clawed frog)
41703	5.38	0.09	0.0000	0.0015	1.90	all_v2.0.1130.C1	Ras-related protein Rab-10; n=18; Gnathostomata|Rep: Ras-related protein Rab-10 - Homo sapiens (Human)
							Homolog of Homo sapiens Spastic ataxia of [illegible]

51766	3.09	0.24	0.0005	0.0099	1.89	all_v2.0.2438.C2	unclassified
42781	2.89	0.26	0.0008	0.0136	1.89	all_v2.0.15196.C1	Transposase; n=1; Strongylocentrotus purpuratus|Rep: Transposase - Strongylocentrotus purpuratus (Purple sea urchin)
36380	4.33	0.14	0.0000	0.0021	1.89	all_v2.0.132.C8	CC chemokine type 2; n=1; Gadus morhua|Rep: CC chemokine type 2 - Gadus morhua (Atlantic cod)
43012	2.99	0.21	0.0006	0.0119	1.88	all_v2.0.9212.C1	UPI0000D8BA1C related cluster; n=1; Danio rerio|Rep: UPI0000D8BA1C UniRef100 entry - Danio rerio
54050	4.10	0.14	0.0001	0.0025	1.88	all_v2.0.9224.C1	unclassified
41181	4.00	0.15	0.0001	0.0028	1.88	all_v2.0.14519.C1	PREDICTED: similar to Ubiquitin carboxyl-terminal hydrolase 5 (Ubiquitin thioesterase 5) (Ubiquitin-specific-processing protease 5) (Deubiquitinating enzyme 5) (Isopeptidase T); n=2; Homo/Pan/Gorilla group|Rep: PREDICTED: similar to Ubiquitin carboxyl-terminal hydrolase 5 (Ubiquitin thioesterase 5) (Ubiquitin-specific-processing protease 5) (Deubiquitinating enzyme 5) (Isopeptidase T) - Homo sapiens
46431	2.63	0.32	0.0017	0.0215	1.88	all_v2.0.15076.C1	Unassigned protein
42754	3.72	0.17	0.0001	0.0039	1.87	sb_gmnbhkas_0008c11.pDNRF2	Transport-associated protein; n=3; Salmonidae|Rep: Transport-associated protein - Salmo salar (Atlantic salmon)
52391	3.88	0.15	0.0001	0.0032	1.87	all_v2.0.8469.C1	unclassified
53177	2.77	0.26	0.0012	0.0173	1.86	all_v2.0.9872.C1	unclassified
53400	2.75	0.25	0.0012	0.0178	1.86	all_v2.0.1718.C1	unclassified
43487	3.56	0.17	0.0002	0.0049	1.86	all_v2.0.5034.C1	Zgc:112064; n=4; Danio rerio|Rep: Zgc:112064 - Danio rerio (Zebrafish) (Brachydanio rerio)
41942	3.16	0.20	0.0004	0.0089	1.86	all_v2.0.8660.C1	RING finger protein 213; n=3; Homo sapiens|Rep: RING finger protein 213 - Homo sapiens (Human)
48323	2.41	0.35	0.0032	0.0310	1.86	sb_gmnbhkic_0005f18.pDNRF2	Unassigned protein
52620	2.99	0.23	0.0006	0.0119	1.85	all_v2.0.12870.C1	unclassified
51093	4.61	0.12	0.0000	0.0018	1.85	all_v2.0.15266.C2	unclassified
43935	2.57	0.27	0.0020	0.0237	1.85	all_v2.0.9690.C1	Zgc:73238; n=1; Danio rerio|Rep: Zgc:73238 - Danio rerio (Zebrafish) (Brachydanio rerio)
49744	2.62	0.23	0.0018	0.0219	1.85	all_v2.0.9839.C1	unclassified
38607	2.64	0.24	0.0017	0.0214	1.84	all_v2.0.7062.C1	Interferon stimulated gene 15; n=1; Gadus morhua|Rep: Interferon stimulated gene 15 - Gadus morhua (Atlantic cod)
51924	2.47	0.26	0.0027	0.0277	1.84	all_v2.0.7974.C1	unclassified
40262	3.47	0.18	0.0002	0.0055	1.84	sb_gmapht_0030a18.pDNRF2	PREDICTED: similar to bloodthirsty; n=2; Danio rerio|Rep: PREDICTED: similar to bloodthirsty - Danio rerio
47614	2.42	0.37	0.0032	0.0306	1.84	sb_gmnlla_0037d01.t7	Unassigned protein
54046	4.04	0.14	0.0001	0.0026	1.83	all_v2.0.5705.C1	unclassified
35894	4.17	0.13	0.0001	0.0024	1.82	all_v2.0.16939.C1	Aminopeptidase N; n=2; Pseudopleuronectes americanus|Rep: Aminopeptidase N - Pseudopleuronectes americanus (Winter flounder) (Pleuronectesamericanus)
35555	2.42	0.26	0.0032	0.0310	1.82	all_v2.0.384.C1	1MDa_1 protein; n=3; Caenorhabditis elegans|Rep: 1MDa_1 protein - Caenorhabditis elegans
45954	2.50	0.32	0.0025	0.0266	1.82	sb_gmnbhkas_0006c23.pD	NSL1, MIND kinetochore complex component, homolog [Gallus gallus] dbj|BAE93420.1| Nsl1

						NRF2	protein [Gallus gallus]
42145	2.43	0.24	0.0031	0.0300	1.82	sb_gmnlkfic_0001b01.t7	Shmt2 protein; n=3; Xenopus|Rep: Shmt2 protein - Xenopus tropicalis (Western clawed frog) (Silurana tropicalis)
39975	3.31	0.19	0.0003	0.0070	1.81	all_v2.0.4370.C3	Phospholipase D4; n=3; Murinae|Rep: Phospholipase D4 - Mus musculus (Mouse)
36381	2.56	0.29	0.0021	0.0244	1.81	all_v2.0.132.C9	CC chemokine type 2; n=1; Gadus morhua|Rep: CC chemokine type 2 - Gadus morhua (Atlantic cod)
38619	3.78	0.14	0.0001	0.0037	1.81	all_v2.0.3448.C5	Interferon-inducible protein Gig1; n=1; Psetta maxima|Rep: Interferon-inducible protein Gig1 - Scophthalmus maximus (Turbot)
45146	4.33	0.12	0.0000	0.0021	1.81	sb_gmnbhkic_0005i22.pDNRm13r	LOC555735; similar to DNA methyltransferase
51265	2.47	0.25	0.0027	0.0278	1.81	all_v2.0.14468.C1	unclassified
52488	3.68	0.15	0.0001	0.0041	1.81	all_v2.0.10916.C1	unclassified
51788	2.55	0.26	0.0022	0.0245	1.81	all_v2.0.10463.C1	unclassified
44197	2.99	0.22	0.0006	0.0119	1.80	sb_gmnlskic_0013o14.pDNRm13r	[E] COG2008 Threonine aldolase
40187	2.59	0.26	0.0019	0.0228	1.80	sb_gmapht_0020g02.pDNRm13r	PREDICTED: similar to actin-related protein 3-beta; n=1; Bos taurus|Rep: PREDICTED: similar to actin-related protein 3-beta - Bos taurus
46606	3.43	0.16	0.0002	0.0058	1.80	sb_gmnlem_0011g08.t7	Unassigned protein
43314	3.12	0.18	0.0004	0.0096	1.79	all_v2.0.8980.C1	Zgc:101649; n=3; Danio rerio|Rep: Zgc:101649 - Danio rerio (Zebrafish) (Brachydanio rerio)
42231	2.98	0.22	0.0006	0.0121	1.79	all_v2.0.3901.C1	Skeletal muscle myosin heavy chain light meromyosin; n=10; Clupeocephala|Rep: Skeletal muscle myosin heavy chain light meromyosin - Clupea harengus (Atlantic herring)
37820	2.57	0.24	0.0021	0.0237	1.79	all_v2.0.2700.C3	Homolog of Brachydanio rerio Sulfotransferase family, cytosolic sulfotransferase 2.; n=1; Takifugu rubripes|Rep: Homolog of Brachydanio rerio Sulfotransferase family, cytosolic sulfotransferase 2 - Takifugu rubripes
48342	3.74	0.14	0.0001	0.0038	1.79	sb_gmnlem_0014f04.t7	Unassigned protein
54421	2.99	0.20	0.0006	0.0119	1.79	all_v2.0.1129.C1	unclassified
38436	3.63	0.16	0.0001	0.0043	1.79	sb_gmapov_0005h22.pDNRF2	Homolog of Homo sapiens Vacuolar sorting protein 54 long isoform; n=1; Takifugu rubripes|Rep: Homolog of Homo sapiens Vacuolar sorting protein 54 long isoform - Takifugu rubripes
37232	2.96	0.20	0.0007	0.0123	1.79	all_v2.0.4884.C1	FK506 binding protein 4; n=3; Danio rerio|Rep: FK506 binding protein 4 - Danio rerio (Zebrafish) (Brachydanio rerio)
38828	3.62	0.15	0.0001	0.0044	1.79	sb_gmnbmu_0008m08.pDNRm13r	LOC553437 protein; n=2; Danio rerio|Rep: LOC553437 protein - Brachydanio rerio (Zebrafish) (Danio rerio)
40442	2.62	0.22	0.0018	0.0219	1.78	all_v2.0.10294.C1	PREDICTED: similar to EPB49 protein; n=2; Danio rerio|Rep: PREDICTED: similar to EPB49 protein - Danio rerio
51579	2.96	0.19	0.0007	0.0124	1.78	all_v2.0.17299.C1	unclassified
41628	3.13	0.18	0.0004	0.0094	1.78	all_v2.0.10802.C1	Putative helicase MOV-10 (EC 3.6.1.-) (Moloney leukemia virus 10 protein).; n=1; Takifugu rubripes|Rep: Putative helicase MOV-10 (EC 3.6.1.-) (Moloney leukemia virus 10 protein). - Takifugu rubripes
43147	2.90	0.22	0.0008	0.0136	1.78	all_v2.0.4262.C1	Vacuolar ATP synthase subunit S1 precursor (EC 3.6.3.14) (V-ATPase subunit S1) (V-ATPase S1 accessory protein) (V-ATPase Ac45 subunit) (XAP-3).; n=1; Takifugu rubripes|Rep: Vacuolar ATP synthase subunit S1 precursor (EC 3.6.3.14)

							(V-ATPase subunit S1) (V-ATPase S1 accessory protein) (V-ATPase Ac45 subunit) (XAP-3). - Takifugu rubripes
40304	2.51	0.24	0.0024	0.0260	1.77	all_v2.0.2720.C1	PREDICTED: similar to CDK2 (cyclin-dependent kinase 2)-associated protein 1; n=2; Canis lupus familiaris|Rep: PREDICTED: similar to CDK2 (cyclin-dependent kinase 2)-associated protein 1 - Canis familiaris
49026	3.04	0.20	0.0005	0.0108	1.77	all_v2.0.270.C2	Unassigned protein
50350	3.24	0.17	0.0003	0.0077	1.77	all_v2.0.6687.C1	unclassified
54199	2.60	0.22	0.0019	0.0226	1.77	all_v2.0.7185.C1	unclassified
51423	3.03	0.20	0.0006	0.0109	1.77	all_v2.0.7996.C1	unclassified
52306	2.52	0.22	0.0024	0.0256	1.76	all_v2.0.14732.C1	unclassified
46576	2.85	0.21	0.0009	0.0148	1.76	all_v2.0.4.C213	Unassigned protein
55434	3.81	0.14	0.0001	0.0036	1.76	all_v2.0.8005.C1	unclassified
41414	2.91	0.21	0.0008	0.0133	1.76	sb_gmnlsfic_0008p05.t7	Proteasome subunit alpha type 6-like protein; n=1; Xenopsylla cheopis|Rep: Proteasome subunit alpha type 6-like protein - Xenopsylla cheopis (oriental rat flea)
52513	2.66	0.25	0.0016	0.0206	1.75	all_v2.0.14032.C1	unclassified
42246	2.61	0.24	0.0018	0.0221	1.75	all_v2.0.14045.C1	Small nuclear ribonucleoprotein polypeptide A; n=2; Danio rerio|Rep: Small nuclear ribonucleoprotein polypeptide A - Danio rerio (Zebrafish) (Brachydanio rerio)
48810	2.77	0.21	0.0012	0.0172	1.75	all_v2.0.7149.C2	Unassigned protein
44092	3.74	0.14	0.0001	0.0038	1.74	all_v2.0.3277.C1	Zgc:92698; n=3; Danio rerio|Rep: Zgc:92698 - Danio rerio (Zebrafish) (Brachydanio rerio)
51254	2.88	0.19	0.0008	0.0140	1.74	all_v2.0.9392.C1	unclassified
46150	2.72	0.25	0.0013	0.0187	1.74	sb_gmnbpcic_0001l22.pDNRm13r	RGD1311345; similar to CG9752-PA
36482	3.74	0.13	0.0001	0.0038	1.74	all_v2.0.1745.C2	Chemokine CXC-like protein; n=1; Psetta maxima|Rep: Chemokine CXC-like protein - Scophthalmus maximus (Turbot)
44663	4.21	0.11	0.0000	0.0023	1.74	all_v2.0.7931.C1	Drf_FH1 multi-domain protein
47291	3.33	0.15	0.0003	0.0068	1.74	all_v2.0.606.C2	Unassigned protein
51908	3.54	0.14	0.0002	0.0049	1.74	all_v2.0.14478.C1	unclassified
36142	4.90	0.08	0.0000	0.0017	1.73	all_v2.0.2387.C5	Beta2-microglobulin precursor; n=3; Gadus morhua|Rep: Beta2-microglobulin precursor - Gadus morhua (Atlantic cod)
43282	2.44	0.30	0.0030	0.0294	1.73	all_v2.0.12393.C1	Zgc:100817; n=1; Danio rerio|Rep: Zgc:100817 - Danio rerio (Zebrafish) (Brachydanio rerio)
54087	2.42	0.24	0.0032	0.0310	1.72	all_v2.0.4115.C1	unclassified
53644	2.69	0.23	0.0014	0.0194	1.72	all_v2.0.10774.C1	unclassified
47988	4.13	0.11	0.0001	0.0025	1.72	all_v2.0.12900.C1	Unassigned protein
47750	2.49	0.25	0.0026	0.0272	1.72	sb_gmnbhkic_0009m05.pDNRF2	Unassigned protein
39974	3.18	0.17	0.0004	0.0087	1.72	all_v2.0.16283.C1	Phospholipase D4 (EC 3.1.4.4) (PLD 4) (Choline phosphatase 4) (Phosphatidylcholine-hydrolyzing phospholipase D4).; n=1; Takifugu rubripes|Rep: Phospholipase D4 (EC 3.1.4.4) (PLD 4) (Choline phosphatase 4) (Phosphatidylcholine-hydrolyzing

39974	3.18	0.17	0.0004	0.0087	1.72	all_v2.0.16283.C1	Phospholipase D4 (EC 3.1.4.4) (PLD 4) (Choline phosphatase 4) (Phosphatidylcholine-hydrolyzing phospholipase D4).; n=1; Takifugu rubripes|Rep: Phospholipase D4 (EC 3.1.4.4) (PLD 4) (Choline phosphatase 4) (Phosphatidylcholine-hydrolyzing phospholipase D4). - Takifugu rubripes
37879	3.11	0.18	0.0004	0.0097	1.72	all_v2.0.14669.C1	Homolog of Fugu rubripes Zygote arrest 1 (Oocyte-specific maternal effect factor).; n=1; Takifugu rubripes|Rep: Homolog of Fugu rubripes Zygote arrest 1 (Oocyte-specific maternal effect factor). - Takifugu rubripes
46742	2.68	0.21	0.0015	0.0200	1.72	all_v2.0.12237.C1	Unassigned protein
47985	2.65	0.25	0.0016	0.0209	1.72	sb_gmnlla_0045c07.t7	Unassigned protein
36789	3.37	0.14	0.0002	0.0062	1.72	sb_gmnlkfic_0001a07.t7	Cytosolic nonspecific dipeptidase; n=3; Percomorpha|Rep: Cytosolic nonspecific dipeptidase - Oreochromis niloticus (Nile tilapia) (Tilapia nilotica)
52097	2.39	0.27	0.0035	0.0328	1.71	all_v2.0.10732.C1	unclassified
40281	3.39	0.15	0.0002	0.0061	1.71	sb_gmnbhkic_0001g19.pDNRF2	PREDICTED: similar to calcium-transporting ATPase; n=1; Monodelphis domestica|Rep: PREDICTED: similar to calcium-transporting ATPase - Monodelphis domestica
41403	2.42	0.22	0.0032	0.0310	1.71	sb_gmnlsfic_0011p03.t7	Proteasome (Prosome, macropain) subunit, alpha type, 6b; n=2; Danio rerio|Rep: Proteasome (Prosome, macropain) subunit, alpha type, 6b - Brachydanio rerio (Zebrafish) (Danio rerio)
39469	2.89	0.19	0.0008	0.0138	1.71	all_v2.0.12276.C1	Novel protein similar to vertebrate EF-hand domain family, member D2; n=2; Danio rerio|Rep: Novel protein similar to vertebrate EF-hand domain family, member D2 - Danio rerio (Zebrafish) (Brachydanio rerio)
43079	3.82	0.13	0.0001	0.0036	1.71	sb_gmapht_0004g05.pDNRF2	UPI0000E4E275 related cluster; n=1; Danio rerio|Rep: UPI0000E4E275 UniRef100 entry - Danio rerio
37853	3.69	0.13	0.0001	0.0040	1.70	all_v2.0.7494.C1	Homolog of Carassius auratus Interferon-inducible protein Gig2.; n=4; Takifugu rubripes|Rep: Homolog of Carassius auratus Interferon-inducible protein Gig2. - Takifugu rubripes
46967	2.65	0.23	0.0016	0.0210	1.70	all_v2.0.2449.C1	Unassigned protein
37178	2.81	0.18	0.0010	0.0162	1.69	all_v2.0.2512.C1	Fatty acid-binding protein, brain (B-FABP) (Brain lipid-binding protein) (BLBP) (Mammary-derived growth inhibitor related).; n=5; Takifugu rubripes|Rep: Fatty acid-binding protein, brain (B-FABP) (Brain lipid-binding protein) (BLBP) (Mammary-derived growth inhibitor related). - Takifugu rubripes
52553	2.55	0.24	0.0022	0.0245	1.69	all_v2.0.10157.C1	unclassified
38441	3.55	0.13	0.0002	0.0049	1.69	sb_gmnbpcic_0007k03.pDNRm13r	Homolog of Homo sapiens VPS13B-1A protein; n=1; Takifugu rubripes|Rep: Homolog of Homo sapiens VPS13B-1A protein - Takifugu rubripes
44531	3.17	0.16	0.0004	0.0088	1.69	IKKAlikeS2	custom - IKKA-like protein
48527	2.49	0.22	0.0026	0.0269	1.69	all_v2.0.14079.C1	Unassigned protein
41821	2.51	0.25	0.0024	0.0261	1.69	all_v2.0.2602.C1	Reverse transcriptase-like protein; n=1; Takifugu rubripes|Rep: Reverse transcriptase-like protein - Fugu rubripes (Japanese pufferfish) (Takifugu rubripes)
42941	2.59	0.22	0.0019	0.0228	1.69	all_v2.0.3501.C1	UBX domain-containing protein 8-A; n=4; Xenopus|Rep: UBX domain-containing protein 8-A - Xenopus laevis (African clawed frog)
							Homolog of Homo sapiens Mannosyl (Alpha-1,3-)-glycoprotein beta-1,4-N-acetylglucosaminyltransferase, isoenzyme B, [illegible]

38160	3.01	0.17	0.0006	0.0116	1.68	sb_gmnlsfic_0003i11.t7	Homolog of Homo sapiens Membrane interacting protein of RGS16; n=1; Takifugu rubripes|Rep: Homolog of Homo sapiens Membrane interacting protein of RGS16 - Takifugu rubripes
49047	3.70	0.12	0.0001	0.0040	1.68	all_v2.0.11108.C4	unclassified
40840	4.47	0.09	0.0000	0.0019	1.68	sb_gmnbhkas_0018n05.pDNRF2	PREDICTED: similar to poly (ADP-ribose) polymerase family, member 11,; n=1; Monodelphis domestica|Rep: PREDICTED: similar to poly (ADP-ribose) polymerase family, member 11, - Monodelphis domestica
49642	3.30	0.16	0.0003	0.0071	1.68	all_v2.0.7220.C1	unclassified
52706	2.75	0.20	0.0012	0.0178	1.68	all_v2.0.17371.C1	unclassified
52646	2.79	0.18	0.0011	0.0165	1.67	all_v2.0.3726.C1	unclassified
47952	3.65	0.13	0.0001	0.0042	1.67	sb_gmnbhkas_0008b05.pDNRF2	Unassigned protein
52340	2.80	0.21	0.0011	0.0164	1.67	all_v2.0.3337.C1	unclassified
46741	3.10	0.17	0.0005	0.0099	1.67	all_v2.0.5124.C2	Unassigned protein
45507	3.40	0.13	0.0002	0.0060	1.67	all_v2.0.13496.C1	non-directed
38629	2.48	0.23	0.0027	0.0274	1.67	sb_gmnlskic_0014o15.pDNRm13r	Interleukin-1 receptor-associated kinase 4 (EC 2.7.11.1) (IRAK-4) (NY- REN-64 antigen).; n=1; Takifugu rubripes|Rep: Interleukin-1 receptor-associated kinase 4 (EC 2.7.11.1) (IRAK-4) (NY-REN-64 antigen). - Takifugu rubripes
54894	2.62	0.21	0.0018	0.0219	1.66	all_v2.0.2012.C2	unclassified
43170	3.39	0.14	0.0002	0.0062	1.66	all_v2.0.3828.C1	Vacuolar protein-sorting-associated protein 36; n=3; Clupeocephala|Rep: Vacuolar protein-sorting-associated protein 36 - Danio rerio (Zebrafish) (Brachydanio rerio)
37655	3.57	0.13	0.0001	0.0047	1.66	sb_gmapov_0006b08.pDNRm13r	Homeobox protein Hox-C13a; n=9; Clupeocephala|Rep: Homeobox protein Hox-C13a - Brachydanio rerio (Zebrafish) (Danio rerio)
51059	3.10	0.15	0.0005	0.0099	1.66	all_v2.0.690.C5	unclassified
40089	2.97	0.17	0.0007	0.0121	1.66	sb_gmnbmu_0008a12.pDNRF2	Polyprotein; n=1; Atlantic salmon swim bladder sarcoma virus|Rep: Polyprotein - Atlantic salmon swim bladder sarcoma virus
49321	2.76	0.18	0.0012	0.0176	1.66	all_v2.0.2925.C1	unclassified
39327	3.23	0.16	0.0003	0.0079	1.66	all_v2.0.12281.C1	NFX1-type zinc finger-containing protein 1.; n=1; Takifugu rubripes|Rep: NFX1-type zinc finger-containing protein 1 - Takifugu rubripes
43609	2.81	0.20	0.0010	0.0162	1.65	sb_gmnbmd_0004p22.t7	Zgc:136585; n=3; Danio rerio|Rep: Zgc:136585 - Brachydanio rerio (Zebrafish) (Danio rerio)
52211	2.60	0.20	0.0019	0.0225	1.65	all_v2.0.1978.C1	unclassified
44338	2.73	0.21	0.0013	0.0184	1.65	sb_gmnlskic_0011d22.pDNRm13r	Borrelia_orfA multi-domain protein
47390	2.43	0.23	0.0031	0.0303	1.65	sb_gmnlem_0005d01.t7	Unassigned protein
44865	2.49	0.22	0.0026	0.0269	1.64	all_v2.0.8362.C1	HERC5, LOC478474; hect domain and RLD 5
47640	2.63	0.19	0.0017	0.0215	1.64	sb_gmnbhkic_0015l18.pDNRm13r	Unassigned protein
48591	2.48	0.20	0.0027	0.0274	1.64	sb_gmnlem_0025k19.t7	Unassigned protein
51581	2.64	0.19	0.0017	0.0212	1.64	all_v2.0.15506.C1	unclassified
38090	3.07	0.15	0.0005	0.0103	1.64	all_v2.0.4946	Homolog of Homo sapiens GTPase, IMAP family

						.C3	member 4; n=1; Takifugu rubripes|Rep: Homolog of Homo sapiens GTPase, IMAP family member 4 - Takifugu rubripes
55211	2.45	0.23	0.0029	0.0290	1.64	all_v2.0.6615.C1	unclassified
47948	3.58	0.12	0.0001	0.0047	1.64	all_v2.0.17446.C1	Unassigned protein
48556	2.93	0.16	0.0007	0.0128	1.63	sb_gmnbmd_0002p01.pDNRm13r	Unassigned protein
41734	2.42	0.23	0.0032	0.0306	1.63	all_v2.0.6458.C1	Repetitive proline-rich cell wall protein 1-related protein; n=7; Trichomonas vaginalis G3|Rep: Repetitive proline-rich cell wall protein 1-related protein - Trichomonas vaginalis G3
39505	2.69	0.20	0.0015	0.0196	1.63	sb_gmapov_0018l09.pDNRF2	Novel protein similar to vertebrate phosphoribosyl pyrophosphate amidotransferase; n=3; Danio rerio|Rep: Novel protein similar to vertebrate phosphoribosyl pyrophosphate amidotransferase - Brachydanio rerio (Zebrafish) (Danio rerio)
41548	2.38	0.25	0.0036	0.0330	1.63	all_v2.0.2626.C1	Protein YIPF6; n=4; Danio rerio|Rep: Protein YIPF6 - Danio rerio (Zebrafish) (Brachydanio rerio)
39586	3.81	0.11	0.0001	0.0036	1.63	all_v2.0.14243.C1	Novel protein; n=2; Danio rerio|Rep: Novel protein - Danio rerio (Zebrafish) (Brachydanio rerio)
54420	2.66	0.18	0.0016	0.0206	1.63	all_v2.0.8985.C1	unclassified
50069	3.62	0.12	0.0001	0.0045	1.63	all_v2.0.17582.C1	unclassified
48511	3.37	0.13	0.0002	0.0063	1.63	sb_gmlbgits_0004e15.sp6	Unassigned protein
49159	2.84	0.16	0.0009	0.0151	1.63	all_v2.0.16350.C1	unclassified
50298	3.35	0.13	0.0002	0.0066	1.62	all_v2.0.17232.C1	unclassified
55152	3.24	0.13	0.0003	0.0077	1.62	all_v2.0.5857.C1	unclassified
40571	2.55	0.18	0.0022	0.0245	1.62	all_v2.0.1957.C1	PREDICTED: similar to intercellular adhesion molecule 5, telencephalin; n=1; Monodelphis domestica|Rep: PREDICTED: similar to intercellular adhesion molecule 5, telencephalin - Monodelphis domestica
48752	2.67	0.18	0.0016	0.0203	1.62	sb_gmnbbrts_0005o12.pDNRm13r	Unassigned protein
38114	4.74	0.07	0.0000	0.0018	1.61	all_v2.0.3580.C6	Homolog of Homo sapiens Inter-alpha (globulIn) InhIbItor H3; n=1; Takifugu rubripes|Rep: Homolog of Homo sapiens Inter-alpha (globulIn) InhIbItor H3 - Takifugu rubripes
39658	2.74	0.17	0.0013	0.0181	1.61	all_v2.0.5240.C2	Novel protein; n=3; Danio rerio|Rep: Novel protein - Danio rerio (Zebrafish) (Brachydanio rerio)
46917	2.69	0.18	0.0015	0.0197	1.61	sb_gmapht_0024c21.pDNRF2	Unassigned protein
44256	3.59	0.11	0.0001	0.0046	1.61	all_v2.0.1221.C3	AIG1 domain containing protein
50024	3.31	0.13	0.0003	0.0070	1.61	all_v2.0.11830.C1	unclassified
39445	2.40	0.19	0.0034	0.0318	1.61	all_v2.0.10010.C1	Novel protein similar to vertebrate angiopoietin family; n=1; Danio rerio|Rep: Novel protein similar to vertebrate angiopoietin family - Danio rerio (Zebrafish) (Brachydanio rerio)
38832	2.48	0.19	0.0026	0.0273	1.61	all_v2.0.17701.C1	LOC558134 protein; n=2; Danio rerio|Rep: LOC558134 protein - Danio rerio (Zebrafish) (Brachydanio rerio)
48696	3.43	0.12	0.0002	0.0058	1.60	all_v2.0.40.C3	Unassigned protein
43951	4.11	0.09	0.0001	0.0025	1.60	sb_gmnbpcic	Zgc:77292; n=2; Danio rerio|Rep: Zgc:77292 -

43951	4.11	0.09	0.0001	0.0025	1.60	sb_gmnbpcic_0010g12.pDNRF2	Zgc:77292; n=2; Danio rerio|Rep: Zgc:77292 - Danio rerio (Zebrafish) (Brachydanio rerio)
51540	2.90	0.16	0.0008	0.0135	1.60	all_v2.0.15577.C1	unclassified
42021	2.56	0.19	0.0021	0.0240	1.59	all_v2.0.9962.C1	Sdf1a; n=2; Danio rerio|Rep: Sdf1a - Danio rerio (Zebrafish) (Brachydanio rerio)
43027	2.87	0.17	0.0009	0.0142	1.59	sb_gmapht_0004p08.pDNRm13r	UPI0000D8C4B2 related cluster; n=2; Danio rerio|Rep: UPI0000D8C4B2 UniRef100 entry - Danio rerio
40401	2.80	0.16	0.0011	0.0164	1.59	all_v2.0.9509.C1	PREDICTED: similar to Developmentally regulated GTP binding protein 1; n=1; Danio rerio|Rep: PREDICTED: similar to Developmentally regulated GTP binding protein 1 - Danio rerio
36150	3.12	0.13	0.0004	0.0095	1.59	sb_gmapov_0026e24.pDNRF2	Beta-glucuronidase precursor; n=1; Sus scrofa|Rep: Beta-glucuronidase precursor - Sus scrofa (Pig)
50815	3.11	0.13	0.0005	0.0097	1.59	all_v2.0.10148.C1	unclassified
46909	2.62	0.17	0.0018	0.0218	1.58	all_v2.0.16431.C1	Unassigned protein
36328	3.96	0.09	0.0001	0.0029	1.58	all_v2.0.16926.C1	CASP8 and FADD-like apoptosis regulator precursor (Cellular FLICE-like inhibitory protein) (c-FLIP) (Caspase-eight-related protein) (Casper) (Caspase-like apoptosis regulatory protein) (CLARP) (MACH-related inducer of toxicity) (MRIT) (Caspase homolog) (C; n=1; Takifugu rubripes|Rep: CASP8 and FADD-like apoptosis regulator precursor (Cellular FLICE-like inhibitory protein) (c-FLIP) (Caspase-eight-related protein) (Casper) (Caspase-like apoptosis regulatory protein) (CLARP) (MACH-related inducer of toxicity) (MRIT) (Caspase homolog) (C - Takifugu rubripes
46167	2.84	0.16	0.0010	0.0151	1.58	all_v2.0.10896.C1	Ripk3; receptor-interacting serine-threonine kinase 3 [EC:2.7.11.1]; K08847 receptor-interacting serine/threonine-protein kinase 3
44580	3.30	0.12	0.0003	0.0070	1.58	NFkappaB1	custom - NFkappaB - nuclear factor kappa-B, subunit 1
41233	2.87	0.16	0.0009	0.0144	1.58	sb_gmapov_0002e13.pDNRF2	PREDICTED: similar to Zinc finger protein 319; n=1; Danio rerio|Rep: PREDICTED: similar to Zinc finger protein 319 - Danio rerio
46412	2.48	0.20	0.0027	0.0277	1.58	all_v2.0.10562.C1	Ubiquitin family protein
43267	2.46	0.21	0.0028	0.0284	1.58	sb_gmapov_0013b17.pDNRF2	XL; n=1; Saimiri boliviensis|Rep: XL - Saimiri boliviensis (Bolivian squirrel monkey)
40679	2.58	0.18	0.0020	0.0232	1.58	all_v2.0.4387.C1	PREDICTED: similar to mKIAA1931 protein; n=1; Monodelphis domestica|Rep: PREDICTED: similar to mKIAA1931 protein - Monodelphis domestica
40005	2.56	0.18	0.0021	0.0241	1.57	all_v2.0.17201.C1	Plasminogen precursor; n=1; Oncorhynchus mykiss|Rep: Plasminogen precursor - Oncorhynchus mykiss (Rainbow trout) (Salmo gairdneri)
46450	2.80	0.16	0.0011	0.0164	1.57	all_v2.0.18464.C1	Unassigned protein
44550	2.48	0.20	0.0027	0.0277	1.56	IRAK41	custom - interleukin-1 receptor-associated kinase 4
47363	2.72	0.17	0.0013	0.0187	1.56	all_v2.0.5685.C1	Unassigned protein
53449	2.75	0.15	0.0012	0.0177	1.56	all_v2.0.10745.C1	unclassified
54782	3.39	0.11	0.0002	0.0062	1.56	all_v2.0.13581.C1	unclassified
48348	2.66	0.16	0.0016	0.0207	1.56	sb_gmnbhkas_0017l24.pDNRF2	Unassigned protein
						all_v2.0.1843	

47408	2.46	0.17	0.0028	0.0284	1.56	sb_gmnbhkic_0012e23.pDNRm13r	Unassigned protein
44015	2.94	0.13	0.0007	0.0127	1.56	all_v2.0.11085.C1	Zgc:86905; n=1; Danio rerio|Rep: Zgc:86905 - Danio rerio (Zebrafish) (Brachydanio rerio)
49961	3.53	0.10	0.0002	0.0051	1.56	all_v2.0.12262.C1	unclassified
45132	2.55	0.18	0.0022	0.0245	1.55	all_v2.0.6091.C1	LOC519254; similar to ATP-dependent RNA helicase DDX18 (DEAD-box protein 18) (Myc-regulated DEAD-box protein) (MrDb)
47124	2.46	0.17	0.0028	0.0283	1.55	sb_gmnbhkic_0014e02.pDNRm13r	Unassigned protein
54462	2.84	0.14	0.0010	0.0152	1.55	all_v2.0.4285.C1	unclassified
52357	2.49	0.17	0.0026	0.0269	1.55	all_v2.0.2097.C1	unclassified
53074	2.63	0.18	0.0018	0.0216	1.55	all_v2.0.10788.C1	unclassified
41088	2.75	0.16	0.0012	0.0177	1.55	all_v2.0.3752.C1	PREDICTED: similar to transmembrane protein 7; n=1; Equus caballus|Rep: PREDICTED: similar to transmembrane protein 7 - Equus caballus
49903	2.67	0.17	0.0015	0.0202	1.55	all_v2.0.17159.C1	unclassified
51595	2.86	0.14	0.0009	0.0145	1.55	all_v2.0.6499.C1	unclassified
43496	2.75	0.16	0.0012	0.0178	1.54	all_v2.0.13664.C1	Zgc:112175; n=3; Danio rerio|Rep: Zgc:112175 - Danio rerio (Zebrafish) (Brachydanio rerio)
53364	2.76	0.14	0.0012	0.0175	1.54	all_v2.0.3851.C1	unclassified
47248	2.40	0.21	0.0034	0.0319	1.54	all_v2.0.3503.C1	Unassigned protein
40503	2.54	0.18	0.0023	0.0250	1.54	sb_gmnbmu_0013g03.pDNRm13r	PREDICTED: similar to gamma-aminobutyric acid (GABA-A) receptor, subunit epsilon; n=1; Homo sapiens|Rep: PREDICTED: similar to gamma-aminobutyric acid (GABA-A) receptor, subunit epsilon - Homo sapiens
39626	2.81	0.15	0.0010	0.0160	1.54	all_v2.0.4.CB27	Novel protein; n=2; Danio rerio|Rep: Novel protein - Danio rerio (Zebrafish) (Brachydanio rerio)
37903	2.45	0.19	0.0029	0.0290	1.53	all_v2.0.11951.C1	Homolog of Gallus gallus Cytochrome P450.; n=1; Takifugu rubripes|Rep: Homolog of Gallus gallus Cytochrome P450. - Takifugu rubripes
49071	2.52	0.17	0.0024	0.0258	1.53	all_v2.0.3825.C1	unclassified
39488	2.69	0.16	0.0014	0.0194	1.53	all_v2.0.16790.C1	Novel protein similar to vertebrate myotubularin related protein 9; n=1; Danio rerio|Rep: Novel protein similar to vertebrate myotubularin related protein 9 - Danio rerio (Zebrafish) (Brachydanio rerio)
54137	2.66	0.15	0.0016	0.0204	1.53	all_v2.0.17649.C1	unclassified
54671	2.79	0.14	0.0011	0.0164	1.53	all_v2.0.13670.C1	unclassified
53832	3.82	0.08	0.0001	0.0036	1.52	all_v2.0.7066.C3	unclassified
43476	3.03	0.13	0.0005	0.0109	1.52	all_v2.0.11065.C1	Zgc:111879 protein; n=4; Danio rerio|Rep: Zgc:111879 protein - Danio rerio (Zebrafish) (Brachydanio rerio)
55493	2.46	0.18	0.0029	0.0285	1.52	sb_gmnblits_0024n20.pDNRF2	Zfp184_predicted; zinc finger protein 184 (Kruppel-like) (predicted); K09228 KRAB domain-containing zinc finger protein
45737	2.51	0.17	0.0024	0.0261	1.52	all_v2.0.15887.C1.a	non-directed
42664	2.81	0.15	0.0010	0.0160	1.52	sb_gmnlskic_0011d03.pDNRm13r	Transcription factor; n=8; Clupeocephala|Rep: Transcription factor - Danio rerio (Zebrafish) (Brachydanio rerio)
39035	3.15	0.11	0.0004	0.0091	1.52	all_v2.0.7743.C1	MGC85251 protein; n=4; Xenopus|Rep: MGC85251 protein - Xenopus laevis (African

							clawed frog)
41492	2.91	0.12	0.0008	0.0135	1.51	sb_gmnbgi_0004b13.pDNRF2	Protein MAK10 homolog; n=4; Clupeocephala|Rep: Protein MAK10 homolog - Danio rerio (Zebrafish) (Brachydanio rerio)
39322	2.58	0.15	0.0020	0.0233	1.51	all_v2.0.6027.C1	NFX1-type zinc finger-containing protein 1.; n=1; Takifugu rubripes|Rep: NFX1-type zinc finger-containing protein 1 - Takifugu rubripes
47515	2.84	0.13	0.0010	0.0152	1.51	sb_gmnlem_0024e22.t7	Unassigned protein
49801	2.64	0.15	0.0017	0.0212	1.51	all_v2.0.5035.C2	unclassified
45101	2.88	0.13	0.0008	0.0139	1.51	all_v2.0.12734.C1	LOC480945; similar to LINE-1 reverse transcriptase homolog
46953	3.04	0.12	0.0005	0.0108	1.51	sb_gmnbhkic_0007o03.pDNRm13r	Unassigned protein
55175	2.74	0.13	0.0013	0.0181	1.50	all_v2.0.17454.C1	unclassified
45411	3.06	0.11	0.0005	0.0105	1.50	sb_gmnlkfic_0003g13.t7	MGC127071; similar to eukaryotic translation initiation factor 3, subunit 12
49465	2.88	0.13	0.0009	0.0141	1.50	all_v2.0.16061.C1	unclassified
35834	3.27	0.10	0.0003	0.0073	1.50	all_v2.0.14969.C1	Alanine-glyoxylate aminotransferase; n=3; Danio rerio|Rep: Alanine-glyoxylate aminotransferase - Danio rerio (Zebrafish) (Brachydanio rerio)
48267	2.40	0.18	0.0034	0.0320	1.50	sb_gmlbgits_0004d03.sp6	Unassigned protein
49860	3.01	0.12	0.0006	0.0116	1.50	all_v2.0.5197.C1	unclassified
49538	2.45	0.18	0.0029	0.0288	1.50	all_v2.0.17441.C1	unclassified
47110	2.62	0.14	0.0018	0.0218	1.50	sb_gmnbhkic_0016l15.pDNRF2	Unassigned protein
52470	3.45	0.09	0.0002	0.0056	1.49	all_v2.0.6174.C1	unclassified
35864	2.98	0.12	0.0006	0.0120	1.49	all_v2.0.6990.C1	Alpha-1,2-mannosyltransferase ALG9 (EC 2.4.1.-) (Asparagine-linked glycosylation protein 9 homolog) (Disrupted in bipolar disorder protein 1).; n=1; Takifugu rubripes|Rep: Alpha-1,2-mannosyltransferase ALG9 (EC 2.4.1.-) (Asparagine-linked glycosylation protein 9 homolog) (Disrupted in bipolar disorder protein 1). - Takifugu rubripes
52914	3.07	0.11	0.0005	0.0102	1.49	all_v2.0.9711.C1	unclassified
50112	3.23	0.10	0.0003	0.0079	1.49	all_v2.0.1796.C1	unclassified
50189	2.50	0.15	0.0025	0.0264	1.49	all_v2.0.6434.C1	unclassified
41888	2.54	0.15	0.0022	0.0248	1.49	all_v2.0.6878.C2	Ribosomal protein L22; n=4; Percomorpha|Rep: Ribosomal protein L22 - Solea senegalensis (Sole)
44434	2.57	0.14	0.0021	0.0237	1.48	CASP82	custom - caspase 8, apoptosis-related cysteine peptidase
48227	2.50	0.15	0.0025	0.0264	1.48	all_v2.0.17837.C1	Unassigned protein
39349	2.50	0.14	0.0025	0.0267	1.48	all_v2.0.3973.C3	N-myc-interactor (Nmi) (N-myc and STAT interactor).; n=1; Takifugu rubripes|Rep: N-myc-interactor (Nmi) (N-myc and STAT interactor). - Takifugu rubripes
50525	2.88	0.12	0.0009	0.0141	1.48	all_v2.0.832.C1	unclassified
49408	2.64	0.14	0.0017	0.0212	1.48	all_v2.0.12168.C3	unclassified
35804	2.60	0.15	0.0019	0.0224	1.48	sb_gmnlem_0013c12.t7	ADP/ATP translocase-like protein; n=3; Arabidopsis thaliana|Rep: ADP/ATP translocase-like protein - Arabidopsis thaliana (Mouse-ear cress)
48403	3.07	0.11	0.0005	0.0102	1.48	sb_gmnlla_00	Unassigned protein

48403	3.07	0.11	0.0005	0.0102	1.48	sb_gmnlla_0048g04.t7	Unassigned protein
45447	2.64	0.14	0.0017	0.0212	1.48	all_v2.0.858.C1	MPPN domain containing protein
46466	2.49	0.16	0.0026	0.0269	1.48	all_v2.0.6161.C1	Unassigned protein
42177	2.38	0.17	0.0036	0.0329	1.48	all_v2.0.13203.C1	Si:dkey-24p1.1; n=3; Danio rerio|Rep: Si:dkey-24p1.1 - Danio rerio (Zebrafish) (Brachydanio rerio)
54746	2.40	0.16	0.0034	0.0321	1.48	all_v2.0.12292.C1	unclassified
41613	2.39	0.17	0.0035	0.0322	1.47	sb_gmnbspic_0003h16.pDNRF2	Putative ATP-dependent RNA helicase DHX33 (EC 3.6.1.-) (DEAH box protein 33).; n=1; Takifugu rubripes|Rep: Putative ATP-dependent RNA helicase DHX33 (EC 3.6.1.-) (DEAH box protein 33). - Takifugu rubripes
53174	2.50	0.15	0.0025	0.0266	1.47	all_v2.0.4725.C1	unclassified
53439	2.40	0.15	0.0034	0.0320	1.47	all_v2.0.6014.C1	unclassified
36422	2.67	0.13	0.0015	0.0200	1.47	all_v2.0.13040.C1	Cell division control protein 42 homolog precursor (G25K GTP-binding protein).; n=1; Takifugu rubripes|Rep: Cell division control protein 42 homolog precursor (G25K GTP-binding protein). - Takifugu rubripes
39878	2.63	0.13	0.0017	0.0215	1.46	all_v2.0.17977.C1	Pentraxin precursor; n=1; Salmo salar|Rep: Pentraxin precursor - Salmo salar (Atlantic salmon)
39449	2.72	0.12	0.0013	0.0187	1.46	all_v2.0.18269.C1	Novel protein similar to vertebrate apolipoprotein B (Including Ag(X) antigen); n=3; Danio rerio|Rep: Novel protein similar to vertebrate apolipoprotein B (Including Ag(X) antigen) - Danio rerio (Zebrafish) (Brachydanio rerio)
43738	2.72	0.12	0.0013	0.0187	1.46	sb_gmnlskic_0013e11.pDNRm13r	Zgc:158229; n=2; Danio rerio|Rep: Zgc:158229 - Danio rerio (Zebrafish) (Brachydanio rerio)
47677	2.58	0.13	0.0020	0.0233	1.46	sb_gmnbhkic_0004l22.pDNRF2	Unassigned protein
51542	2.38	0.15	0.0035	0.0329	1.46	all_v2.0.6459.C1	unclassified
44439	2.57	0.14	0.0020	0.0237	1.45	CASP85	custom - caspase 8, apoptosis-related cysteine peptidase
47982	2.57	0.13	0.0020	0.0237	1.45	sb_gmapht_0015h06.pDNRF2	Unassigned protein
55051	2.46	0.14	0.0028	0.0284	1.45	all_v2.0.9309.C1	unclassified
49076	2.92	0.11	0.0008	0.0133	1.45	all_v2.0.12705.C1	unclassified
36676	2.50	0.14	0.0025	0.0267	1.45	all_v2.0.3061.C1	crumbs homolog 3a; n=1; Danio rerio|Rep: crumbs homolog 3a - Danio rerio
36451	2.57	0.14	0.0021	0.0237	1.45	all_v2.0.18657.C1	Centromere protein T (CENP-T) (Interphase centromere complex protein 22).; n=2; Gallus gallus|Rep: Centromere protein T (CENP-T) (Interphase centromere complex protein 22). - Gallus gallus
42324	3.08	0.09	0.0005	0.0101	1.45	all_v2.0.1037.C1	Sorting nexin 10; n=2; Takifugu rubripes|Rep: Sorting nexin 10 - Fugu rubripes (Japanese pufferfish) (Takifugu rubripes)
39731	2.86	0.11	0.0009	0.0146	1.44	all_v2.0.7197.C1	Nuclear cap binding protein subunit 2; n=4; Clupeocephala|Rep: Nuclear cap binding protein subunit 2 - Danio rerio (Zebrafish) (Brachydanio rerio)
39219	2.62	0.12	0.0018	0.0216	1.44	sb_gmnlmfta_0004d02.t7	Myosin light chain 3; n=1; Theragra chalcogramma|Rep: Myosin light chain 3 - Theragra chalcogramma (Alaska pollock)
[illegible]	2.84	0.11	0.0009	0.0151	1.44	all_v2.0.1080[illegible]	phospholipase C delta 3; n=1; Takifugu rubripes|Rep: phospholipase C delta 3 - Takifugu [illegible]

46836	2.50	0.14	0.0025	0.0267	1.44	sb_gmnlla_0031d20.t7	Unassigned protein
39266	2.38	0.14	0.0036	0.0331	1.44	all_v2.0.1166.C1	NADH dehydrogenase 1 alpha; n=1; Psetta maxima|Rep: NADH dehydrogenase 1 alpha - Scophthalmus maximus (Turbot)
51622	2.63	0.12	0.0017	0.0215	1.43	all_v2.0.13212.C1	unclassified
52038	2.53	0.13	0.0023	0.0251	1.43	all_v2.0.17560.C1	unclassified
43788	2.64	0.12	0.0017	0.0212	1.43	all_v2.0.11056.C1	Zgc:162641 protein; n=3; Danio rerio|Rep: Zgc:162641 protein - Danio rerio (Zebrafish) (Brachydanio rerio)
55134	2.86	0.11	0.0009	0.0145	1.43	all_v2.0.392.C2	unclassified
51980	3.15	0.09	0.0004	0.0090	1.43	all_v2.0.703.C1	unclassified
37498	2.90	0.10	0.0008	0.0136	1.43	all_v2.0.3198.C3	GTP-binding nuclear protein Ran; n=35; Euteleostomi|Rep: GTP-binding nuclear protein Ran - Homo sapiens (Human)
36122	2.41	0.14	0.0033	0.0314	1.43	all_v2.0.4102.C1	Bcl-2-related ovarian killer protein homolog B; n=3; Danio rerio|Rep: Bcl-2-related ovarian killer protein homolog B - Danio rerio (Zebrafish) (Brachydanio rerio)
35854	2.52	0.13	0.0024	0.0259	1.43	all_v2.0.1645.C1	Alkylated repair protein alkB homolog 5.; n=2; Gallus gallus|Rep: Alkylated repair protein alkB homolog 5 - Gallus gallus
40337	2.69	0.11	0.0014	0.0194	1.42	sb_gmnlbric_0003k18.t7	PREDICTED: similar to cGMP phosphodiesterase A2; n=1; Ornithorhynchus anatinus|Rep: PREDICTED: similar to cGMP phosphodiesterase A2 - Ornithorhynchus anatinus
50893	2.52	0.13	0.0024	0.0259	1.42	all_v2.0.1794.C1	unclassified
52806	2.48	0.12	0.0027	0.0277	1.42	all_v2.0.3338.C1	unclassified
46261	2.54	0.13	0.0022	0.0248	1.42	all_v2.0.10360.C1	si:rp71-45k5.3; si:rp71-45k5.3
42425	2.40	0.14	0.0034	0.0320	1.42	sb_gmnlkfic_0004n14.t7	Survival motor neuron domain containing 1; n=1; Danio rerio|Rep: Survival motor neuron domain containing 1 - Brachydanio rerio (Zebrafish) (Danio rerio)
38293	3.65	0.06	0.0001	0.0042	1.42	all_v2.0.15532.C1	Homolog of Homo sapiens Proprotein convertase subtilisin/kexin type 5 precursor; n=1; Takifugu rubripes|Rep: Homolog of Homo sapiens Proprotein convertase subtilisin/kexin type 5 precursor - Takifugu rubripes
52528	2.55	0.12	0.0022	0.0246	1.41	all_v2.0.15651.C1	unclassified
40559	2.72	0.11	0.0013	0.0187	1.41	sb_gmapht_0017j13.pDNRF2	PREDICTED: similar to Importin alpha-1 subunit (Karyopherin alpha-1 subunit) (SRP1-beta) (RAG cohort protein 2) (Nucleoprotein interactor 1) (NPI-1), partial; n=1; Danio rerio|Rep: PREDICTED: similar to Importin alpha-1 subunit (Karyopherin alpha-1 subunit) (SRP1-beta) (RAG cohort protein 2) (Nucleoprotein interactor 1) (NPI-1), partial - Danio rerio
50493	3.17	0.08	0.0004	0.0088	1.41	all_v2.0.7812.C1	unclassified
36675	2.49	0.13	0.0026	0.0268	1.41	sb_gmapov_0024k13.pDNRF2	CRSP complex subunit 6; n=14; Amniota|Rep: CRSP complex subunit 6 - Homo sapiens (Human)
44649	2.99	0.09	0.0006	0.0120	1.41	all_v2.0.661.C6	DKEY-218F9.5, LOC571141; novel protein similar to bloodthirsty (bty)
44066	2.74	0.10	0.0013	0.0180	1.41	all_v2.0.7765.C1	Zgc:92254; n=2; Danio rerio|Rep: Zgc:92254 - Danio rerio (Zebrafish) (Brachydanio rerio)
55284	3.01	0.09	0.0006	0.0116	1.40	all_v2.0.2423.C1	unclassified
45088	3.16	0.07	0.0004	0.0090	1.40	sb_gmnlkric_0002a08.t7	LOC477822; similar to hyaluronan binding protein 2 [EC:3.4.21.-]; K08648 hyaluronan binding protein 2

54293	3.21	0.07	0.0003	0.0081	1.40	all_v2.0.5441.C1	unclassified
39077	2.81	0.09	0.0010	0.0162	1.39	all_v2.0.431.C7	Microtubule aggregate protein homolog; n=1; Perca flavescens|Rep: Microtubule aggregate protein homolog - Perca flavescens (Yellow perch)
47382	2.54	0.11	0.0023	0.0249	1.39	sb_gmapht_0030g24.pDNRF2	Unassigned protein
51078	2.90	0.09	0.0008	0.0135	1.39	all_v2.0.2037.C3	unclassified
44931	2.57	0.11	0.0021	0.0237	1.39	all_v2.0.11243.C1	I-set domain containing protein
42023	2.54	0.10	0.0023	0.0250	1.38	sb_gmapov_0025i02.pDNRF2	SEC13 homolog; n=5; Clupeocephala|Rep: SEC13 homolog - Brachydanio rerio (Zebrafish) (Danio rerio)
38263	2.47	0.11	0.0028	0.0280	1.38	all_v2.0.10484.C1	Homolog of Homo sapiens Placenta-specific gene 8 protein; n=1; Takifugu rubripes|Rep: Homolog of Homo sapiens Placenta-specific gene 8 protein - Takifugu rubripes
47516	3.20	0.07	0.0003	0.0082	1.38	all_v2.0.5207.C1	Unassigned protein
53247	2.75	0.09	0.0012	0.0177	1.38	all_v2.0.1141.C1	unclassified
48825	2.74	0.09	0.0013	0.0180	1.38	sb_gmnlla_0004a24.t7	Unassigned protein
35985	2.99	0.08	0.0006	0.0119	1.38	all_v2.0.17834.C1	Armadillo repeat protein deleted in velo-cardio-facial syndrome.; n=1; Takifugu rubripes|Rep: Armadillo repeat protein deleted in velo-cardio-facial syndrome. - Takifugu rubripes
39321	2.43	0.12	0.0031	0.0301	1.38	all_v2.0.6027.C5	NFX1-type zinc finger-containing protein 1.; n=1; Takifugu rubripes|Rep: NFX1-type zinc finger-containing protein 1 - Takifugu rubripes
35556	2.61	0.10	0.0018	0.0220	1.38	sb_gmnbspic_0013g06.pDNRF2	1-phosphatidylinositol-4,5-bisphosphate phosphodiesterase gamma 2 (EC 3.1.4.11) (Phosphoinositide phospholipase C) (PLC-gamma-2) (Phospholipase C-gamma-2) (PLC-IV).; n=1; Takifugu rubripes|Rep: 1-phosphatidylinositol-4,5-bisphosphate phosphodiesterase gamma 2 (EC 3.1.4.11) (Phosphoinositide phospholipase C) (PLC-gamma-2) (Phospholipase C-gamma-2) (PLC-IV). - Takifugu rubripes
49764	2.41	0.10	0.0033	0.0312	1.37	all_v2.0.1447.C1	unclassified
48148	2.48	0.10	0.0027	0.0275	1.37	sb_gmnlbric_0007c23.t7	Unassigned protein
53784	2.61	0.09	0.0019	0.0223	1.36	all_v2.0.124.C1	unclassified
51902	2.79	0.08	0.0011	0.0165	1.36	all_v2.0.11621.C1	unclassified
37024	2.71	0.08	0.0014	0.0188	1.36	sb_gmnllrta_0004d06.t7	Embryonic alpha-type globin; n=1; Oncorhynchus mykiss|Rep: Embryonic alpha-type globin - Oncorhynchus mykiss (Rainbow trout) (Salmo gairdneri)
49834	3.08	0.06	0.0005	0.0100	1.35	all_v2.0.12788.C1	unclassified
50913	2.50	0.09	0.0025	0.0267	1.35	all_v2.0.3093.C1	unclassified
46509	2.42	0.10	0.0032	0.0306	1.35	sb_gmnbhkic_0006d08.pDNRF2	Unassigned protein
48700	2.55	0.09	0.0022	0.0246	1.34	sb_gmnbhkic_0016p03.pDNRm13r	Unassigned protein
44431	2.72	0.07	0.0013	0.0187	1.33	CASP106	custom - caspase 10, apoptosis-related cysteine peptidase
49004	2.39	0.09	0.0035	0.0323	1.33	sb_gmnbpcic_0005h18.pDNRF2	Unassigned protein
44606	2.45	0.09	0.0029	0.0290	1.32	5toll_like_rec	custom - toll-like receptor 3 (TLR3)

44606	2.45	0.09	0.0029	0.0290	1.32	5toll_like_receptor_3	custom - toll-like receptor 3 (TLR3)
37561	2.45	0.09	0.0029	0.0291	1.32	sb_gmnbbr_0004n04.pDNRm13r	Heat shock protein 9; n=5; Clupeocephala|Rep: Heat shock protein 9 - Brachydanio rerio (Zebrafish) (Danio rerio)
39587	2.48	0.08	0.0027	0.0274	1.31	all_v2.0.5876.C1	Novel protein; n=2; Danio rerio|Rep: Novel protein - Danio rerio (Zebrafish) (Brachydanio rerio)
49818	2.55	0.07	0.0022	0.0246	1.30	all_v2.0.3977.C1	unclassified
50205	2.56	0.07	0.0021	0.0240	1.30	all_v2.0.9219.C1	unclassified
38379	2.68	0.05	0.0015	0.0197	1.28	all_v2.0.3343.C1	Homolog of Homo sapiens TiTin isoform novex-2; n=1; Takifugu rubripes|Rep: Homolog of Homo sapiens TiTin isoform novex-2 - Takifugu rubripes
46732	2.45	0.06	0.0029	0.0290	1.28	sb_gmnlem_0027j18.t7	Unassigned protein
39664	2.45	0.03	0.0030	0.0293	1.20	all_v2.0.3408.C1	Novel protein; n=3; Danio rerio|Rep: Novel protein - Danio rerio (Zebrafish) (Brachydanio rerio)
44125	-3.28	0.06	0.0003	0.0072	0.72	all_v2.0.11869.C2	Zinc finger CCHC-type and RNA-binding motif-containing protein 1; n=2; Xenopus|Rep: Zinc finger CCHC-type and RNA-binding motif-containing protein 1 - Xenopus laevis (African clawed frog)
52833	-3.78	0.05	0.0001	0.0037	0.72	all_v2.0.4044.C2	unclassified
46639	-3.29	0.08	0.0003	0.0071	0.70	sb_gmnlla_0005b20.t7	Unassigned protein
47851	-3.35	0.08	0.0002	0.0065	0.70	all_v2.0.12891.C1	Unassigned protein
42385	-3.75	0.08	0.0001	0.0038	0.66	all_v2.0.1135.C1	Stathmin; n=2; Percomorpha|Rep: Stathmin - Tetraodon nigroviridis (Green puffer)
46891	-3.27	0.11	0.0003	0.0074	0.65	sb_gmapov_0009k19.pDNRm13r	Unassigned protein
51867	-4.15	0.08	0.0001	0.0025	0.64	all_v2.0.4395.C1	unclassified
46395	-3.27	0.12	0.0003	0.0074	0.64	sb_gmnbmu_0007h07.pDNRm13r	TT_ORF1 multi-domain protein
43634	-3.38	0.12	0.0002	0.0062	0.64	all_v2.0.13742.C1	Zgc:152897; n=3; Danio rerio|Rep: Zgc:152897 - Danio rerio (Zebrafish) (Brachydanio rerio)
45113	-4.67	0.07	0.0000	0.0018	0.63	sb_gmapov_0018o17.pDNRF2	LOC488397; similar to Methyltransferase-like protein 2
40487	-4.32	0.08	0.0000	0.0021	0.62	sb_gmapte_0012e12.pDNRF2	PREDICTED: similar to G protein-coupled receptor 142; n=1; Ornithorhynchus anatinus|Rep: PREDICTED: similar to G protein-coupled receptor 142 - Ornithorhynchus anatinus
45017	-3.34	0.12	0.0002	0.0066	0.62	sb_gmnlbric_0004p23.t7	LOC100022136; similar to Dedicator of cytokinesis protein 10 (Zizimin-3)
44380	-3.66	0.12	0.0001	0.0042	0.61	sb_gmapte_0004f14.pDNRm13r	CDC10; cell division cycle 10
54441	-3.70	0.12	0.0001	0.0040	0.61	all_v2.0.10503.C1	unclassified
38516	-4.10	0.11	0.0001	0.0025	0.59	all_v2.0.14475.C1	Hsp90 co-chaperone Cdc37; n=3; Tetraodontidae|Rep: Hsp90 co-chaperone Cdc37 - Tetraodon fluviatilis (Puffer fish)
50398	-3.36	0.15	0.0002	0.0063	0.58	all_v2.0.3774.C1	unclassified
47293	-3.38	0.16	0.0002	0.0062	0.58	all_v2.0.8868.C1	Unassigned protein
36157	-3.44	0.15	0.0002	0.0057	0.58	all_v2.0.10467.C2	Betaine--homocysteine S-methyltransferase 1; n=3; Danio rerio|Rep: Betaine--homocysteine S-methyltransferase 1 - Danio rerio (Zebrafish) (Brachydanio rerio)
[illegible]	[illegible]	[illegible]	[illegible]	[illegible]	[illegible]	all_v2.0.6017 [illegible]	Zgc:92811; n=1; Danio rerio|Rep: Zgc:92811 - [illegible]

44373	-3.75	0.15	0.0001	0.0038	0.56	sb_gmapov_0002h23.pDNRm13r	CBX4; chromobox homolog 4 (Pc class homolog, Drosophila)
46942	-4.55	0.10	0.0000	0.0019	0.56	all_v2.0.18242.C1	Unassigned protein
39550	-3.25	0.18	0.0003	0.0076	0.55	all_v2.0.4405.C1	Novel protein simlar to vertebrate vacuolar protein sorting 4 homolog B; n=4; Danio rerio|Rep: Novel protein simlar to vertebrate vacuolar protein sorting 4 homolog B - Danio rerio (Zebrafish) (Brachydanio rerio)
44357	-4.78	0.11	0.0000	0.0017	0.54	all_v2.0.4599.C1	Caldesmon multi-domain protein
48035	-3.49	0.19	0.0002	0.0053	0.53	sb_gmnlsfic_0006g23.t7	Unassigned protein
41581	-4.76	0.12	0.0000	0.0017	0.52	sb_gmnlla_0036k22.sp6	Protocadherin 2G24; n=2; Tetraodontidae|Rep: Protocadherin 2G24 - Fugu rubripes (Japanese pufferfish) (Takifugu rubripes)
55012	-4.07	0.15	0.0001	0.0026	0.52	all_v2.0.365.C1	unclassified
51726	-4.85	0.12	0.0000	0.0017	0.51	all_v2.0.447.C1	unclassified
44053	-4.95	0.12	0.0000	0.0016	0.50	all_v2.0.9640.C1	Zgc:92082; n=4; Clupeocephala|Rep: Zgc:92082 - Danio rerio (Zebrafish) (Brachydanio rerio)
55146	-4.67	0.14	0.0000	0.0018	0.49	all_v2.0.712.C1	unclassified
46599	-3.59	0.21	0.0001	0.0046	0.48	sb_gmnlem_0013c08.sp6	Unassigned protein
54442	-3.31	0.28	0.0003	0.0070	0.46	all_v2.0.3297.C1	unclassified
41600	-3.42	0.25	0.0002	0.0059	0.45	all_v2.0.18698.C1	PSMC3 interacting protein isoform 1; n=1; Takifugu rubripes|Rep: PSMC3 interacting protein isoform 1 - Takifugu rubripes
53859	-3.32	0.26	0.0003	0.0069	0.44	all_v2.0.5016.C2	unclassified
49988	-3.67	0.25	0.0001	0.0042	0.44	all_v2.0.9043.C1	unclassified
44480	-3.35	0.30	0.0002	0.0065	0.39	2HBA3	custom - cod hemoglobin
43204	-3.26	0.38	0.0003	0.0075	0.37	all_v2.0.4026.C3	Viral A-type inclusion protein repeat; n=2; Entamoeba histolytica HM-1:IMSS|Rep: Viral A-type inclusion protein repeat - Entamoeba histolytica HM-1:IMSS
47836	-3.25	0.36	0.0003	0.0076	0.33	all_v2.0.6287.C1	Unassigned protein
43729	-3.67	0.37	0.0001	0.0041	0.30	all_v2.0.6906.C1	Zgc:154086; n=1; Danio rerio|Rep: Zgc:154086 - Danio rerio (Zebrafish) (Brachydanio rerio)
44381	-4.73	0.33	0.0000	0.0018	0.25	all_v2.0.200.C3	CDC27 multi-domain protein

[a]Fold-changes are presented as outputted by siggenes and were calculated as pIC/PBS

Supplemental Table S4. 999 genes differentially expressed between fish injected with pIC or PBS at 10°C and sampled at 24HPI

Probe ID	d-value	Stdev	Raw p	q-value	Fold Change[a]	Sequence Name	Gene Name
38604	23.85	0.21	0.0000	0.0012	90.24	all_v2.0.14.CB2	Interferon stimulated gene 15; n=1; Gadus morhua|Rep: Interferon stimulated gene 15 - Gadus morhua (Atlantic cod)
44453	21.28	0.23	0.0000	0.0012	70.53	CK10a	custom - CC chemokine
44448	14.96	0.35	0.0000	0.0012	66.64	CK10ad	custom - CC chemokine
38605	18.23	0.26	0.0000	0.0012	50.90	all_v2.0.14.C13	Interferon stimulated gene 15; n=1; Gadus morhua|Rep: Interferon stimulated gene 15 - Gadus morhua (Atlantic cod)
38613	10.54	0.44	0.0000	0.0012	40.99	all_v2.0.14.CB5	Interferon stimulated gene 15; n=1; Gadus morhua|Rep: Interferon stimulated gene 15 - Gadus morhua (Atlantic cod)
41316	13.20	0.33	0.0000	0.0012	31.96	all_v2.0.1009.C5	Probable ATP-dependent helicase LGP2 (EC 3.6.1.-) (Protein D11Lgp2 homolog).; n=1; Takifugu rubripes|Rep: Probable ATP-dependent helicase LGP2 (EC 3.6.1.-) (Protein D11Lgp2 homolog). - Takifugu rubripes
44590	15.94	0.25	0.0000	0.0012	30.48	DXH4a	custom - probable ATP-dependent RNA helicase DHX
45882	10.24	0.42	0.0000	0.0012	27.01	all_v2.0.5246.C1	non-directed
38611	11.57	0.37	0.0000	0.0012	26.98	all_v2.0.1767.C1	Interferon stimulated gene 15; n=1; Gadus morhua|Rep: Interferon stimulated gene 15 - Gadus morhua (Atlantic cod)
38638	11.59	0.35	0.0000	0.0012	25.33	all_v2.0.638.C1	Interleukin-8 variant 2; n=3; Ictalurus punctatus|Rep: Interleukin-8 variant 2 - Ictalurus punctatus (Channel catfish)
38609	17.37	0.21	0.0000	0.0012	25.28	all_v2.0.14.C3	Interferon stimulated gene 15; n=1; Gadus morhua|Rep: Interferon stimulated gene 15 - Gadus morhua (Atlantic cod)
38608	20.16	0.17	0.0000	0.0012	24.81	all_v2.0.14.C12	Interferon stimulated gene 15; n=1; Gadus morhua|Rep: Interferon stimulated gene 15 - Gadus morhua (Atlantic cod)
38606	23.91	0.13	0.0000	0.0012	23.34	all_v2.0.14.CB4	Interferon stimulated gene 15; n=1; Gadus morhua|Rep: Interferon stimulated gene 15 - Gadus morhua (Atlantic cod)
38614	14.96	0.25	0.0000	0.0012	22.96	all_v2.0.7062.C2	Interferon stimulated gene 15; n=1; Gadus morhua|Rep: Interferon stimulated gene 15 - Gadus morhua (Atlantic cod)
38639	16.33	0.20	0.0000	0.0012	20.00	all_v2.0.638.CB1	Interleukin-8 variant 2; n=3; Ictalurus punctatus|Rep: Interleukin-8 variant 2 - Ictalurus punctatus (Channel catfish)
38356	11.32	0.32	0.0000	0.0012	17.37	all_v2.0.415.C6	Homolog of Homo sapiens Spastic ataxia of Charlevoix-Saguenay; n=1; Takifugu rubripes|Rep: Homolog of Homo sapiens Spastic ataxia of Charlevoix-Saguenay - Takifugu rubripes
47164	9.84	0.36	0.0000	0.0012	16.08	all_v2.0.6027.C6	Unassigned protein
44589	13.88	0.24	0.0000	0.0012	15.92	DXH3b	custom - probable ATP-dependent RNA helicase DHX
44588	14.79	0.21	0.0000	0.0012	14.50	DXH2b	custom - probable ATP-dependent RNA helicase DHX
48390	8.42	0.42	0.0000	0.0012	14.24	all_v2.0.15354.C1	Unassigned protein
41269	14.19	0.21	0.0000	0.0012	13.93	sb_gmnlbfic_0004o20.t7	PREDICTED: similar to zinc finger, NFX1-type containing 1; n=3; Laurasiatheria|Rep: PREDICTED: similar to zinc finger, NFX1-type containing 1 - Equus caballus
41314	13.39	0.21	0.0000	0.0012	11.71	all_v2.0.5598.C1	Probable ATP-dependent helicase LGP2 (EC 3.6.1.-) (Protein D11Lgp2 homolog).; n=1; Takifugu rubripes|Rep: Probable ATP-dependent helicase LGP2 (EC 3.6.1.-

							) (Protein D11Lgp2 homolog). - Takifugu rubripes
44859	19.78	0.12	0.0000	0.0012	11.50	all_v2.0.314.C1	HECT domain containing protein
38603	16.08	0.16	0.0000	0.0012	11.33	all_v2.0.14.CB7	Interferon stimulated gene 15; n=1; Gadus morhua|Rep: Interferon stimulated gene 15 - Gadus morhua (Atlantic cod)
44180	9.45	0.29	0.0000	0.0012	9.91	all_v2.0.1266.C1	ZPC4; n=2; Oryzias latipes|Rep: ZPC4 - Oryzias latipes (Medaka fish) (Japanese ricefish)
38675	8.68	0.32	0.0000	0.0012	9.64	all_v2.0.9761.C1	Isoform S of Q76IC6 ; n=1; Rattus norvegicus|Rep: Isoform S of Q76IC6 - Rattus norvegicus (Rat)
38617	6.17	0.47	0.0000	0.0013	8.88	all_v2.0.13188.C1	Interferon-inducible GTPase 5 (EC 3.6.5.-) (Immunity-related GTPase cinema 1).; n=3; Takifugu rubripes|Rep: Interferon-inducible GTPase 5 (EC 3.6.5.-) (Immunity-related GTPase cinema 1). - Takifugu rubripes
38651	9.23	0.27	0.0000	0.0012	8.50	sb_gmapht_0023a03.pDNRF2	Intraflagellar transport protein 52; n=4; Danio rerio|Rep: Intraflagellar transport protein 52 - Brachydanio rerio (Zebrafish) (Danio rerio)
39328	9.23	0.29	0.0000	0.0012	8.43	all_v2.0.2213.C1	NFX1-type zinc finger-containing protein 1; n=5; Eutheria|Rep: NFX1-type zinc finger-containing protein 1 - Homo sapiens (Human)
45361	8.84	0.27	0.0000	0.0012	7.77	all_v2.0.34.C2	LOC797694; similar to solute carrier family 25 member 5 protein
44464	13.27	0.16	0.0000	0.0012	7.60	CK10add	custom - CC chemokine
38612	13.33	0.16	0.0000	0.0012	7.48	all_v2.0.14.CB3	Interferon stimulated gene 15; n=1; Gadus morhua|Rep: Interferon stimulated gene 15 - Gadus morhua (Atlantic cod)
44584	10.78	0.21	0.0000	0.0012	7.33	DXH1a	custom - probable ATP-dependent RNA helicase DHX
47410	6.78	0.38	0.0000	0.0012	7.10	sb_gmnlsfic_0004h02.t7	Unassigned protein
40667	9.73	0.23	0.0000	0.0012	6.90	all_v2.0.431.C8	PREDICTED: similar to microtubule aggregate protein homolog; n=2; Danio rerio|Rep: PREDICTED: similar to microtubule aggregate protein homolog - Danio rerio
46200	7.92	0.30	0.0000	0.0012	6.76	all_v2.0.14575.C1	SACS; sacsin
44591	13.29	0.14	0.0000	0.0012	6.45	DXH4b	custom - probable ATP-dependent RNA helicase DHX
38285	6.70	0.35	0.0000	0.0012	6.35	sb_gmnlbfic_0007k16.t7	Homolog of Homo sapiens Probable ATP-dependent helicase LGP2; n=1; Takifugu rubripes|Rep: Homolog of Homo sapiens Probable ATP-dependent helicase LGP2 - Takifugu rubripes
41313	12.34	0.15	0.0000	0.0012	6.07	all_v2.0.1009.C1	Probable ATP-dependent helicase LGP2 (EC 3.6.1.-) (Protein D11Lgp2 homolog).; n=1; Takifugu rubripes|Rep: Probable ATP-dependent helicase LGP2 (EC 3.6.1.-) (Protein D11Lgp2 homolog). - Takifugu rubripes
44909	3.03	0.75	0.0008	0.0082	5.81	all_v2.0.3619.C1	Ifi-6-16 multi-domain protein
49247	10.72	0.18	0.0000	0.0012	5.74	all_v2.0.7226.C1	unclassified
38354	9.00	0.23	0.0000	0.0012	5.68	all_v2.0.707.C4	Homolog of Homo sapiens Spastic ataxia of Charlevoix-Saguenay; n=1; Takifugu rubripes|Rep: Homolog of Homo sapiens Spastic ataxia of Charlevoix-Saguenay - Takifugu rubripes
40939	8.04	0.25	0.0000	0.0012	5.66	all_v2.0.910.C2	PREDICTED: similar to Rhomboid, veinlet-like 7 (Drosophila); n=1; Danio rerio|Rep: PREDICTED: similar to Rhomboid, veinlet-like 7 (Drosophila) -

							Danio rerio
38358	7.66	0.28	0.0000	0.0012	5.57	all_v2.0.9190.C1	Homolog of Homo sapiens Spastic ataxia of Charlevoix-Saguenay; n=1; Takifugu rubripes|Rep: Homolog of Homo sapiens Spastic ataxia of Charlevoix-Saguenay - Takifugu rubripes
47329	8.10	0.25	0.0000	0.0012	5.52	all_v2.0.415.C3	Unassigned protein
38359	11.04	0.17	0.0000	0.0012	5.50	all_v2.0.415.C5	Homolog of Homo sapiens Spastic ataxia of Charlevoix-Saguenay; n=1; Takifugu rubripes|Rep: Homolog of Homo sapiens Spastic ataxia of Charlevoix-Saguenay - Takifugu rubripes
47613	8.38	0.23	0.0000	0.0012	5.22	sb_gmnbbr_0013m15.pDNRm13r	Unassigned protein
55231	6.73	0.30	0.0000	0.0012	5.17	all_v2.0.8766.C1	unclassified
38357	8.53	0.22	0.0000	0.0012	5.14	all_v2.0.8243.C1	Homolog of Homo sapiens Spastic ataxia of Charlevoix-Saguenay; n=1; Takifugu rubripes|Rep: Homolog of Homo sapiens Spastic ataxia of Charlevoix-Saguenay - Takifugu rubripes
55535	9.38	0.19	0.0000	0.0012	5.08	all_v2.0.15359.C1	zp3a.1; zona pellucida glycoprotein 3a.1
38607	11.62	0.14	0.0000	0.0012	4.76	all_v2.0.7062.C1	Interferon stimulated gene 15; n=1; Gadus morhua|Rep: Interferon stimulated gene 15 - Gadus morhua (Atlantic cod)
43201	6.89	0.26	0.0000	0.0012	4.68	all_v2.0.9330.C1	Viperin-like protein; n=1; Channa argus|Rep: Viperin-like protein - Channa argus (snakehead)
48258	14.70	0.09	0.0000	0.0012	4.63	all_v2.0.14926.C1	Unassigned protein
44371	7.72	0.22	0.0000	0.0012	4.56	all_v2.0.2805.C1	caspc; caspase c
37299	8.62	0.19	0.0000	0.0012	4.52	all_v2.0.3679.C1	Gadd45al protein; n=2; Danio rerio|Rep: Gadd45al protein - Danio rerio (Zebrafish) (Brachydanio rerio)
47384	10.57	0.15	0.0000	0.0012	4.48	all_v2.0.707.C1	Unassigned protein
46199	5.61	0.34	0.0000	0.0013	4.35	all_v2.0.2412.C1	SACS; sacsin
46226	9.55	0.16	0.0000	0.0012	4.30	all_v2.0.415.C1	Serpentine_recp domain containing protein
45139	2.90	0.63	0.0012	0.0103	4.19	sb_gmnbhkic_0005c17.pDNRF2	LOC539080; similar to immunity-related GTPase family, cinema 1
35753	7.49	0.21	0.0000	0.0012	4.17	sb_gmnlsras_0003g09.t7	Actin-binding vascular maintenance protein; n=5; Danio rerio|Rep: Actin-binding vascular maintenance protein - Danio rerio (Zebrafish) (Brachydanio rerio)
37854	6.17	0.27	0.0000	0.0013	4.14	sb_gmnlsfic_0002b12.t7	Homolog of Carassius auratus Interferon-inducible protein Gig2.; n=4; Takifugu rubripes|Rep: Homolog of Carassius auratus Interferon-inducible protein Gig2. - Takifugu rubripes
55502	8.29	0.18	0.0000	0.0012	4.14	all_v2.0.7892.C1	zgc:110548; zgc:110548
38260	2.78	0.49	0.0016	0.0127	4.11	all_v2.0.16638.C1	Homolog of Homo sapiens PhosPholiPase C-like 3; n=1; Takifugu rubripes|Rep: Homolog of Homo sapiens PhosPholiPase C-like 3 - Takifugu rubripes
41942	8.64	0.18	0.0000	0.0012	4.11	all_v2.0.8660.C1	RING finger protein 213; n=3; Homo sapiens|Rep: RING finger protein 213 - Homo sapiens (Human)
38378	3.54	0.43	0.0003	0.0037	4.04	all_v2.0.5950.C3	Homolog of Homo sapiens TiTin isoform novex-2; n=1; Takifugu rubripes|Rep: Homolog of Homo sapiens TiTin isoform novex-2 - Takifugu rubripes

43196	6.85	0.24	0.0000	0.0012	4.04	all_v2.0.3448.C3	VHSV-induced protein; n=1; Oncorhynchus mykiss|Rep: VHSV-induced protein - Oncorhynchus mykiss (Rainbow trout) (Salmo gairdneri)
44254	4.49	0.35	0.0001	0.0017	4.01	sb_gmnbhkas_0009a09.pDNRF2	AhpC-TSA domain containing protein
38042	9.38	0.15	0.0000	0.0012	3.94	all_v2.0.894.C5	Homolog of Homo sapiens Deoxyribonuclease gamma precursor; n=1; Takifugu rubripes|Rep: Homolog of Homo sapiens Deoxyribonuclease gamma precursor - Takifugu rubripes
39490	8.48	0.17	0.0000	0.0012	3.91	all_v2.0.5879.C2	Novel protein similar to vertebrate nebulin; n=3; Danio rerio|Rep: Novel protein similar to vertebrate nebulin - Danio rerio (Zebrafish) (Brachydanio rerio)
51382	4.64	0.34	0.0001	0.0016	3.89	all_v2.0.5897.C3	unclassified
46202	7.12	0.21	0.0000	0.0012	3.85	sb_gmnbmd_0010a21.pDNRm13r	Sacs_predicted; sacsin (predicted)
41014	10.09	0.13	0.0000	0.0012	3.80	all_v2.0.14158.C1	PREDICTED: similar to Snf2-related CBP activator protein; n=1; Mus musculus|Rep: PREDICTED: similar to Snf2-related CBP activator protein - Mus musculus
44283	5.48	0.27	0.0000	0.0013	3.77	sb_gmnlmfta_0004i04.t7	ARL6IP6; ADP-ribosylation-like factor 6 interacting protein 6
46412	6.35	0.24	0.0000	0.0013	3.77	all_v2.0.10562.C1	Ubiquitin family protein
45583	5.27	0.31	0.0000	0.0014	3.74	all_v2.0.2772.C15	non-directed
54821	6.92	0.21	0.0000	0.0012	3.71	all_v2.0.10668.C2	unclassified
39509	3.07	0.46	0.0008	0.0077	3.68	all_v2.0.15398.C1	Novel protein similar to vertebrate plectin 1, intermediate filament binding protein 500kDa; n=2; Danio rerio|Rep: Novel protein similar to vertebrate plectin 1, intermediate filament binding protein 500kDa - Danio rerio (Zebrafish) (Brachydanio rerio)
44587	9.54	0.14	0.0000	0.0012	3.66	DXH3a	custom - probable ATP-dependent RNA helicase DHX
38670	5.86	0.27	0.0000	0.0013	3.66	all_v2.0.3891.C1	Isoform 2 of Q5RI56 ; n=2; Danio rerio|Rep: Isoform 2 of Q5RI56 - Danio rerio (Zebrafish) (Brachydanio rerio)
41595	4.46	0.32	0.0001	0.0017	3.64	sb_gmapov_0028l22.pDNRF2	PRP4 pre-mRNA processing factor 4 homolog; n=2; Danio rerio|Rep: PRP4 pre-mRNA processing factor 4 homolog - Brachydanio rerio (Zebrafish) (Danio rerio)
47806	9.35	0.14	0.0000	0.0012	3.64	sb_gmnsul1_0001h03.pDNRF2	Unassigned protein
38552	10.07	0.13	0.0000	0.0012	3.62	all_v2.0.10046.C1	ilvB (bacterial acetolactate synthase)-like; n=1; Takifugu rubripes|Rep: ilvB (bacterial acetolactate synthase)-like - Takifugu rubripes
49852	3.71	0.37	0.0002	0.0030	3.57	all_v2.0.15227.C1	unclassified
42073	10.25	0.12	0.0000	0.0012	3.56	all_v2.0.15254.C1	Serine hydrolase-like protein 2 (EC 3.1.-.-).; n=1; Takifugu rubripes|Rep: Serine hydrolase-like protein 2 (EC 3.1.-.-). - Takifugu rubripes
42028	5.31	0.28	0.0000	0.0014	3.53	sb_gmnbhkic_0012l24.pDNRF2	Secernin 3; n=3; Tetraodontidae|Rep: Secernin 3 - Fugu rubripes (Japanese pufferfish) (Takifugu rubripes)
37221	3.18	0.43	0.0006	0.0064	3.53	all_v2.0.8506.C1	Fibrinogen beta chain; n=1; Pseudosciaena crocea (Croceine croaker)|Rep: Fibrinogen beta chain - Pseudosciaena crocea (Croceine croaker)

53239	3.02	0.62	0.0009	0.0084	3.52	all_v2.0.5097.C2	unclassified
39189	5.06	0.29	0.0000	0.0014	3.51	sb_gmnbhkic_0017j02.pDNRm13r	Myelin transcription factor 1-like protein (MyT1L protein) (MyT1-L).; n=1; Takifugu rubripes|Rep: Myelin transcription factor 1-like protein (MyT1L protein) (MyT1-L). - Takifugu rubripes
37232	7.03	0.20	0.0000	0.0012	3.50	all_v2.0.4884.C1	FK506 binding protein 4; n=3; Danio rerio|Rep: FK506 binding protein 4 - Danio rerio (Zebrafish) (Brachydanio rerio)
44375	10.52	0.11	0.0000	0.0012	3.49	all_v2.0.16663.C1	cct5; chaperonin containing TCP1, subunit 5 (epsilon); K09497 T-complex protein 1 subunit epsilon
42842	7.88	0.17	0.0000	0.0012	3.46	sb_gmnlmfta_0005n11.t7	Twinfilin-2; n=2; Danio rerio|Rep: Twinfilin-2 - Danio rerio (Zebrafish) (Brachydanio rerio)
47445	3.33	0.43	0.0004	0.0049	3.46	sb_gmnbmd_0004h11.t7	Unassigned protein
44324	5.69	0.26	0.0000	0.0013	3.45	all_v2.0.1123.C1	Atrophin-1 multi-domain protein
39974	10.92	0.11	0.0000	0.0012	3.45	all_v2.0.16283.C1	Phospholipase D4 (EC 3.1.4.4) (PLD 4) (Choline phosphatase 4) (Phosphatidylcholine-hydrolyzing phospholipase D4).; n=1; Takifugu rubripes|Rep: Phospholipase D4 (EC 3.1.4.4) (PLD 4) (Choline phosphatase 4) (Phosphatidylcholine-hydrolyzing phospholipase D4). - Takifugu rubripes
51171	7.85	0.17	0.0000	0.0012	3.44	all_v2.0.12475.C1	unclassified
36190	5.51	0.28	0.0000	0.0013	3.43	sb_gmapht_0014i15.pDNRF2	Bloodthirsty; n=2; Notothenioidei|Rep: Bloodthirsty - Chaenocephalus aceratus (White crocodile fish)
54090	6.62	0.21	0.0000	0.0012	3.42	all_v2.0.2294.C1	unclassified
39975	8.99	0.14	0.0000	0.0012	3.42	all_v2.0.4370.C3	Phospholipase D4; n=3; Murinae|Rep: Phospholipase D4 - Mus musculus (Mouse)
40846	5.22	0.29	0.0000	0.0014	3.41	all_v2.0.4119.C1	PREDICTED: similar to Poly [ADP-ribose] polymerase 14 (PARP-14) (B aggressive lymphoma protein 2); n=1; Danio rerio|Rep: PREDICTED: similar to Poly [ADP-ribose] polymerase 14 (PARP-14) (B aggressive lymphoma protein 2) - Danio rerio
39325	8.14	0.16	0.0000	0.0012	3.40	all_v2.0.9595.C2	NFX1-type zinc finger-containing protein 1.; n=1; Takifugu rubripes|Rep: NFX1-type zinc finger-containing protein 1 - Takifugu rubripes
54933	5.64	0.25	0.0000	0.0013	3.39	all_v2.0.5297.C2	unclassified
45506	7.01	0.20	0.0000	0.0012	3.37	all_v2.0.9616.C1.a	non-directed
44546	7.46	0.18	0.0000	0.0012	3.35	IRF1OK4	custom - Interferon regulatory factor 10K
47050	10.19	0.11	0.0000	0.0012	3.34	sb_gmapte_0012e14.pDNRF2	Unassigned protein
38598	9.86	0.12	0.0000	0.0012	3.34	all_v2.0.1580.C7	Interferon regulatory factor 1; n=1; Channa argus|Rep: Interferon regulatory factor 1 - Channa argus (snakehead)
40236	3.30	0.39	0.0004	0.0053	3.32	sb_gmapov_0016j14.pDNRF2	PREDICTED: similar to ATPase, H+ transporting, lysosomal V0 subunit a isoform 1 isoform 2; n=1; Canis familiaris|Rep: PREDICTED: similar to ATPase, H+ transporting, lysosomal V0 subunit a isoform 1 isoform 2 - Canis familiaris
36186	12.62	0.08	0.0000	0.0012	3.30	all_v2.0.661.C2	Bloodthirsty; n=2; Notothenioidei|Rep: Bloodthirsty - Chaenocephalus aceratus (White crocodile fish)

41829	6.70	0.20	0.0000	0.0012	3.29	sb_gmnblits_0018p08.pDNRF2	RFK protein; n=2; Bos taurus|Rep: RFK protein - Bos taurus (Bovine)
41317	9.36	0.12	0.0000	0.0012	3.27	all_v2.0.1165.C4	Probable ATP-dependent helicase LGP2 (EC 3.6.1.-) (Protein D11Lgp2 homolog).; n=1; Takifugu rubripes|Rep: Probable ATP-dependent helicase LGP2 (EC 3.6.1.-) (Protein D11Lgp2 homolog). - Takifugu rubripes
51371	10.10	0.11	0.0000	0.0012	3.25	all_v2.0.5978.C1	unclassified
43197	4.96	0.33	0.0000	0.0014	3.24	all_v2.0.3448.C4	VHSV-induced protein; n=1; Oncorhynchus mykiss|Rep: VHSV-induced protein - Oncorhynchus mykiss (Rainbow trout) (Salmo gairdneri)
45076	4.46	0.30	0.0001	0.0017	3.24	sb_gmapov_0017b11.pDNRm13r	LOC465562; similar to thymopoietin beta
39368	2.86	0.46	0.0013	0.0112	3.23	all_v2.0.11577.C1	Novel immune type receptor protein; n=1; Danio rerio|Rep: Novel immune type receptor protein - Danio rerio (Zebrafish) (Brachydanio rerio)
44543	6.20	0.21	0.0000	0.0013	3.23	IRF1OK1	custom - Interferon regulatory factor 10K
41027	4.03	0.42	0.0001	0.0022	3.19	sb_gmlbgits_0004k02.sp6	PREDICTED: similar to SPAC167.01; n=1; Danio rerio|Rep: PREDICTED: similar to SPAC167.01 - Danio rerio
54050	4.98	0.28	0.0000	0.0014	3.19	all_v2.0.9224.C1	unclassified
45749	6.02	0.22	0.0000	0.0013	3.19	all_v2.0.17951.C1.a	non-directed
38695	9.57	0.11	0.0000	0.0012	3.15	all_v2.0.7632.C1	K14 protein; n=1; Xenopus laevis|Rep: K14 protein - Xenopus laevis (African clawed frog)
55051	5.59	0.23	0.0000	0.0013	3.14	all_v2.0.9309.C1	unclassified
39627	7.93	0.15	0.0000	0.0012	3.14	all_v2.0.3732.C3	Novel protein; n=2; Danio rerio|Rep: Novel protein - Danio rerio (Zebrafish) (Brachydanio rerio)
44919	4.11	0.32	0.0001	0.0021	3.12	all_v2.0.17051.C1	Igsf2_predicted; immunoglobulin superfamily, member 2 (predicted); K06522 immunoglobulin superfamily, member 2/3/2008
44586	4.93	0.28	0.0000	0.0014	3.11	DXH1b	custom - probable ATP-dependent RNA helicase DHX
46586	7.12	0.17	0.0000	0.0012	3.10	sb_gmnlem_0013j07.sp6	Unassigned protein
45162	6.99	0.18	0.0000	0.0012	3.09	all_v2.0.7368.C2	LOC561785; similar to very large inducible GTPase 1
39505	7.50	0.16	0.0000	0.0012	3.08	sb_gmapov_0018l09.pDNRF2	Novel protein similar to vertebrate phosphoribosyl pyrophosphate amidotransferase; n=3; Danio rerio|Rep: Novel protein similar to vertebrate phosphoribosyl pyrophosphate amidotransferase - Brachydanio rerio (Zebrafish) (Danio rerio)
45325	6.07	0.21	0.0000	0.0013	3.06	all_v2.0.14677.C1	LOC764254; similar to transposase
38618	3.02	0.40	0.0009	0.0084	3.04	all_v2.0.3462.C1	Interferon-inducible GTPase 5 (EC 3.6.5.-) (Immunity-related GTPase cinema 1).; n=3; Takifugu rubripes|Rep: Interferon-inducible GTPase 5 (EC 3.6.5.-) (Immunity-related GTPase cinema 1). - Takifugu rubripes
37611	3.60	0.35	0.0002	0.0034	3.04	all_v2.0.2730.C1	Heterogeneous nuclear ribonucleoprotein A3; n=2; Takifugu rubripes|Rep: Heterogeneous nuclear ribonucleoprotein A3 - Fugu rubripes (Japanese pufferfish) (Takifugu rubripes)
52906	7.72	0.15	0.0000	0.0012	3.04	all_v2.0.2709.C1	unclassified

43192	10.14	0.10	0.0000	0.0012	3.03	sb_gmnlbric_0006j07.t7	Vesicular glutamate transporter 2.2; n=3; Danio rerio|Rep: Vesicular glutamate transporter 2.2 - Danio rerio (Zebrafish) (Brachydanio rerio)
35718	2.64	0.47	0.0023	0.0165	3.02	sb_gmnbhkas_0013h13.pDNRF2	A disintegrin and metalloproteinase domain 8; n=1; Danio rerio|Rep: A disintegrin and metalloproteinase domain 8 - Brachydanio rerio (Zebrafish) (Danio rerio)
40543	6.46	0.19	0.0000	0.0013	3.01	all_v2.0.1220.C1	PREDICTED: similar to hCG28999,; n=1; Monodelphis domestica|Rep: PREDICTED: similar to hCG28999, - Monodelphis domestica
51636	7.24	0.16	0.0000	0.0012	3.01	all_v2.0.1410.C1	unclassified
47462	6.02	0.21	0.0000	0.0013	2.99	sb_gmnbhkas_0017n11.pDNRF2	Unassigned protein
54507	2.99	0.39	0.0009	0.0088	2.97	all_v2.0.6751.C1	unclassified
44624	8.29	0.13	0.0000	0.0012	2.95	Codviperin1	custom - viperin
45046	9.14	0.11	0.0000	0.0012	2.89	sb_gmapht_0020j08.pDNRF2	LOC420518; similar to putative tyrosine phosphatase
39077	9.18	0.11	0.0000	0.0012	2.88	all_v2.0.431.C7	Microtubule aggregate protein homolog; n=1; Perca flavescens|Rep: Microtubule aggregate protein homolog - Perca flavescens (Yellow perch)
47156	5.16	0.25	0.0000	0.0014	2.88	all_v2.0.617.C5	Unassigned protein
47244	3.15	0.36	0.0006	0.0067	2.88	sb_gmnbpcic_0009j16.pDNRm13r	Unassigned protein
48764	6.54	0.18	0.0000	0.0012	2.88	all_v2.0.3141.C3	Unassigned protein
39326	5.12	0.24	0.0000	0.0014	2.85	all_v2.0.9595.C1	NFX1-type zinc finger-containing protein 1.; n=1; Takifugu rubripes|Rep: NFX1-type zinc finger-containing protein 1 - Takifugu rubripes
45784	6.33	0.18	0.0000	0.0013	2.85	all_v2.0.17073.C1	non-directed
47376	3.88	0.30	0.0002	0.0025	2.83	all_v2.0.15415.C1	Unassigned protein
45351	6.09	0.19	0.0000	0.0013	2.83	all_v2.0.1010.C8	LOC793474; similar to stonustoxin alpha-subunit
47847	5.31	0.22	0.0000	0.0014	2.79	all_v2.0.14003.C2	Unassigned protein
43557	6.31	0.18	0.0000	0.0013	2.78	all_v2.0.2666.C5	Zgc:113527; n=4; Danio rerio|Rep: Zgc:113527 - Danio rerio (Zebrafish) (Brachydanio rerio)
43639	3.01	0.51	0.0009	0.0085	2.77	all_v2.0.8922.C1	Zgc:152929; n=2; Danio rerio|Rep: Zgc:152929 - Danio rerio (Zebrafish) (Brachydanio rerio)
40397	3.74	0.34	0.0002	0.0029	2.76	all_v2.0.6028.C2	PREDICTED: similar to deltex 3-like (Drosophila); n=1; Danio rerio|Rep: PREDICTED: similar to deltex 3-like (Drosophila) - Danio rerio
38094	3.96	0.35	0.0001	0.0023	2.75	sb_gmnbhkic_0005c22.pDNRF2	Homolog of Homo sapiens HECT domain and RCC1-like domain protein 3; n=1; Takifugu rubripes|Rep: Homolog of Homo sapiens HECT domain and RCC1-like domain protein 3 - Takifugu rubripes
41418	2.94	0.36	0.0010	0.0096	2.75	all_v2.0.659.C1	Proteasome subunit alpha type; n=8; Euteleostomi|Rep: Proteasome subunit alpha type - Danio rerio (Zebrafish) (Brachydanio rerio)
46411	4.01	0.29	0.0001	0.0022	2.75	all_v2.0.4647.C1	ubiquitin domain containing protein
44117	5.97	0.18	0.0000	0.0013	2.74	sb_gmnbgi_0002m22.pDNRF2	Zinc finger CCCH type domain containing protein 1.; n=1; Xenopus tropicalis|Rep: Zinc finger CCCH type domain containing

							protein 1 - Xenopus tropicalis
36311	6.52	0.16	0.0000	0.0012	2.73	all_v2.0.12206.C1	Cardiac troponin T; n=14; Danio rerio|Rep: Cardiac troponin T - Danio rerio (Zebrafish) (Brachydanio rerio)
37853	7.22	0.14	0.0000	0.0012	2.73	all_v2.0.7494.C1	Homolog of Carassius auratus Interferon-inducible protein Gig2.; n=4; Takifugu rubripes|Rep: Homolog of Carassius auratus Interferon-inducible protein Gig2. - Takifugu rubripes
53370	6.28	0.18	0.0000	0.0013	2.72	all_v2.0.11557.C2	unclassified
40980	6.35	0.17	0.0000	0.0013	2.71	sb_gmapov_0018g11.pDNRF2	PREDICTED: similar to scavenger receptor cysteine-rich protein precursor, partial; n=1; Strongylocentrotus purpuratus|Rep: PREDICTED: similar to scavenger receptor cysteine-rich protein precursor, partial - Strongylocentrotus purpuratus
55194	5.49	0.21	0.0000	0.0013	2.71	all_v2.0.9305.C1	unclassified
44585	3.29	0.51	0.0005	0.0053	2.71	DXH2a	custom - probable ATP-dependent RNA helicase DHX
52706	12.44	0.06	0.0000	0.0012	2.70	all_v2.0.17371.C1	unclassified
43476	5.73	0.20	0.0000	0.0013	2.68	all_v2.0.11065.C1	Zgc:111879 protein; n=4; Danio rerio|Rep: Zgc:111879 protein - Danio rerio (Zebrafish) (Brachydanio rerio)
54924	7.69	0.13	0.0000	0.0012	2.68	all_v2.0.12344.C1	unclassified
48557	4.05	0.31	0.0001	0.0022	2.67	sb_gmnbspic_0009m06.pDNRm13r	Unassigned protein
52606	6.47	0.16	0.0000	0.0013	2.67	all_v2.0.5996.C2	unclassified
47640	4.94	0.24	0.0000	0.0014	2.67	sb_gmnbhkic_0015l18.pDNRm13r	Unassigned protein
39119	4.51	0.25	0.0001	0.0017	2.66	all_v2.0.5993.C1	Mitochondrial Rho GTPase 1-A; n=1; Danio rerio|Rep: Mitochondrial Rho GTPase 1-A - Danio rerio (Zebrafish) (Brachydanio rerio)
49270	3.12	0.38	0.0007	0.0070	2.66	all_v2.0.10470.C1	unclassified
38353	7.26	0.14	0.0000	0.0012	2.66	all_v2.0.415.C2	Homolog of Homo sapiens Spastic ataxia of Charlevoix-Saguenay; n=1; Takifugu rubripes|Rep: Homolog of Homo sapiens Spastic ataxia of Charlevoix-Saguenay - Takifugu rubripes
38024	4.19	0.26	0.0001	0.0020	2.64	sb_gmnlsfas_0004l17.t7	Homolog of Homo sapiens Chloride intracellular channel protein 4; n=1; Takifugu rubripes|Rep: Homolog of Homo sapiens Chloride intracellular channel protein 4 - Takifugu rubripes
39694	2.56	0.39	0.0029	0.0191	2.64	all_v2.0.14399.C1	Novel protein; n=4; Danio rerio|Rep: Novel protein - Danio rerio (Zebrafish) (Brachydanio rerio)
38171	10.39	0.08	0.0000	0.0012	2.64	all_v2.0.4661.C1	Homolog of Homo sapiens Microtubule-associated protein 2 isoforM 3; n=1; Takifugu rubripes|Rep: Homolog of Homo sapiens Microtubule-associated protein 2 isoforM 3 - Takifugu rubripes
40226	3.06	0.35	0.0008	0.0077	2.63	all_v2.0.8272.C1	PREDICTED: similar to APG16L beta; n=1; Monodelphis domestica|Rep: PREDICTED: similar to APG16L beta - Monodelphis domestica
36195	12.18	0.06	0.0000	0.0012	2.62	sb_gmnlbfic_0007a23.t7	Bloodthirsty; n=2; Notothenioidei|Rep: Bloodthirsty - Chaenocephalus aceratus (White crocodile fish)
45862	5.52	0.20	0.0000	0.0013	2.61	all_v2.0.16576.C1	non-directed

44579	5.06	0.22	0.0000	0.0014	2.61	3MKK4_MAP2K3	custom - Mitogen-activated protein kinase kinase 4
46519	2.62	0.39	0.0025	0.0172	2.60	sb_gmnbhkas_0012b05.pDNRF2	Unassigned protein
49008	7.38	0.13	0.0000	0.0012	2.60	sb_gmnlla_0002h23.t7	Unassigned protein
44336	6.43	0.16	0.0000	0.0013	2.59	sb_gmapht_0037b08.pDNRF2	bloodthirsty [Notothenia coriiceps]
36160	5.58	0.19	0.0000	0.0013	2.59	all_v2.0.6011.C2	Beta-ureidopropionase (EC 3.5.1.6) (Beta-alanine synthase) (N- carbamoyl-beta-alanine amidohydrolase) (BUP-1).; n=1; Takifugu rubripes|Rep: Beta-ureidopropionase (EC 3.5.1.6) (Beta-alanine synthase) (N- carbamoyl-beta-alanine amidohydrolase) (BUP-1). - Takifugu rubripes
41810	6.52	0.16	0.0000	0.0012	2.59	sb_gmnbhkic_0006h11.pDNRF2	Reverse transcriptase-like protein; n=1; Paralichthys olivaceus|Rep: Reverse transcriptase-like protein - Paralichthys olivaceus (Japanese flounder)
36185	10.76	0.07	0.0000	0.0012	2.58	all_v2.0.661.C20	Bloodthirsty; n=2; Notothenioidei|Rep: Bloodthirsty - Chaenocephalus aceratus (White crocodile fish)
35957	6.27	0.17	0.0000	0.0013	2.58	all_v2.0.15919.C1	Apolipoprotein A-IV4; n=3; Takifugu rubripes|Rep: Apolipoprotein A-IV4 - Fugu rubripes (Japanese pufferfish) (Takifugu rubripes)
55269	5.68	0.18	0.0000	0.0013	2.56	all_v2.0.9958.C1	unclassified
37073	5.02	0.21	0.0000	0.0014	2.56	sb_gmnbspic_0005b03.pDNRF2	EPSTI1 protein; n=3; Bos taurus|Rep: EPSTI1 protein - Bos taurus (Bovine)
47605	4.06	0.28	0.0001	0.0022	2.56	sb_gmnlla_0044f23.t7	Unassigned protein
36317	5.44	0.20	0.0000	0.0013	2.56	all_v2.0.15280.C1	Carnitine palmitoyltransferase II; n=2; Danio rerio|Rep: Carnitine palmitoyltransferase II - Danio rerio (Zebrafish) (Brachydanio rerio)
38512	3.81	0.27	0.0002	0.0027	2.55	all_v2.0.8649.C1	HRAS-like suppressor 3; n=1; Perca flavescens|Rep: HRAS-like suppressor 3 - Perca flavescens (Yellow perch)
46887	4.65	0.24	0.0001	0.0015	2.54	sb_gmnbpcic_0013o02.pDNRF2	Unassigned protein
44910	3.79	0.30	0.0002	0.0028	2.54	all_v2.0.3619.C2	Ifi-6-16 multi-domain protein
54925	3.61	0.32	0.0002	0.0034	2.54	all_v2.0.6896.C1	unclassified
45229	2.62	0.41	0.0025	0.0172	2.53	all_v2.0.5468.C1	LOC593299; similar to zinc finger protein
41940	5.60	0.18	0.0000	0.0013	2.51	sb_gmnbspic_0015c08.pDNRF2	RING finger protein 213.; n=1; Gallus gallus|Rep: RING finger protein 213 - Gallus gallus
42226	3.56	0.33	0.0003	0.0036	2.51	all_v2.0.11820.C1	Sjogren syndrome antigen B; n=1; Danio rerio|Rep: Sjogren syndrome antigen B - Danio rerio (Zebrafish) (Brachydanio rerio)
52488	7.30	0.12	0.0000	0.0012	2.51	all_v2.0.10916.C1	unclassified
54239	3.84	0.26	0.0002	0.0026	2.50	all_v2.0.11344.C1	unclassified
40054	4.45	0.25	0.0001	0.0017	2.49	all_v2.0.2666.C4	Poly [ADP-ribose] polymerase 15 (EC 2.4.2.30) (PARP-15) (B-aggressive lymphoma protein 3).; n=1; Takifugu rubripes|Rep: Poly [ADP-ribose] polymerase 15 (EC 2.4.2.30) (PARP-15) (B-aggressive lymphoma protein 3). - Takifugu rubripes
50539	4.76	0.22	0.0001	0.0015	2.48	all_v2.0.16996.	unclassified

						C1	
46576	8.54	0.10	0.0000	0.0012	2.48	all_v2.0.4.C213	Unassigned protein
36612	5.45	0.18	0.0000	0.0013	2.47	all_v2.0.12481.C1	Complement component C3; n=1; Paralichthys olivaceus|Rep: Complement component C3 - Paralichthys olivaceus (Japanese flounder)
40174	4.75	0.22	0.0001	0.0015	2.46	all_v2.0.11060.C1	PREDICTED: similar to 60 kDa heat shock protein, mitochondrial precursor (Hsp60) (60 kDa chaperonin) (CPN60) (Heat shock protein 60) (HSP-60) (Mitochondrial matrix protein P1) (HSP-65); n=1; Rattus norvegicus|Rep: PREDICTED: similar to 60 kDa heat shock protein, mitochondrial precursor (Hsp60) (60 kDa chaperonin) (CPN60) (Heat shock protein 60) (HSP-60) (Mitochondrial matrix protein P1) (HSP-65) - Rattus norvegicus
48810	7.47	0.12	0.0000	0.0012	2.46	all_v2.0.7149.C2	Unassigned protein
40489	6.54	0.14	0.0000	0.0012	2.46	sb_gmnbbr_0003e16.pDNRF2	PREDICTED: similar to G protein-coupled receptor 97; n=2; Canis familiaris|Rep: PREDICTED: similar to G protein-coupled receptor 97 - Canis familiaris
41164	6.57	0.14	0.0000	0.0012	2.44	sb_gmnbspic_0006i05.pDNRm13r	PREDICTED: similar to tubby super-family protein isoform 2; n=2; Monodelphis domestica|Rep: PREDICTED: similar to tubby super-family protein isoform 2 - Monodelphis domestica
39204	6.33	0.15	0.0000	0.0013	2.44	sb_gmnlmfta_0003l11.t7	Myomesin-2 (M-protein) (165 kDa titin-associated protein) (165 kDa connectin-associated protein).; n=1; Takifugu rubripes|Rep: Myomesin-2 (M-protein) (165 kDa titin-associated protein) (165 kDa connectin-associated protein). - Takifugu rubripes
37609	4.36	0.25	0.0001	0.0018	2.44	all_v2.0.796.C2	Hepcidin; n=1; Pseudosciaena crocea (Croceine croaker)|Rep: Hepcidin - Pseudosciaena crocea (Croceine croaker)
47485	5.06	0.20	0.0000	0.0014	2.44	all_v2.0.10276.C1	Unassigned protein
47020	5.46	0.18	0.0000	0.0013	2.43	all_v2.0.526.C2	Unassigned protein
36718	2.97	0.36	0.0010	0.0090	2.43	sb_gmnlkfas_0004d19.t7	Cyclin-L1; n=3; Xenopus|Rep: Cyclin-L1 - Xenopus laevis (African clawed frog)
36339	4.53	0.23	0.0001	0.0017	2.42	all_v2.0.7453.C3	Caspase-1; n=1; Dicentrarchus labrax|Rep: Caspase-1 - Dicentrarchus labrax (European sea bass)
45045	5.96	0.16	0.0000	0.0013	2.42	all_v2.0.7551.C1	LOC420294; similar to cylindromatosis (turban tumor syndrome); cylindromatosis 1, turban tumor syndrome
48417	6.36	0.15	0.0000	0.0013	2.41	sb_gmnbpcic_0009d03.pDNRm13r	Unassigned protein
54421	7.97	0.10	0.0000	0.0012	2.41	all_v2.0.1129.C1	unclassified
46414	5.38	0.17	0.0000	0.0013	2.40	sb_gmnlsfic_0011a05.t7	UCH domain containing protein
46051	6.59	0.14	0.0000	0.0012	2.39	sb_gmnbhkic_0019j15.pDNRF2	PREDICTED: similar to Poly [ADP-ribose] polymerase 14 (PARP-14) (B aggressive lymphoma protein 2), partial [Danio rerio]
45542	6.94	0.13	0.0000	0.0012	2.39	all_v2.0.16396.C1.a	non-directed
41920	5.72	0.16	0.0000	0.0013	2.39	sb_gmapov_0012l04.pDNRF2	Ribulose-5-phosphate-3-epimerase; n=1; Danio rerio|Rep: Ribulose-5-phosphate-3-epimerase - Brachydanio rerio (Zebrafish) (Danio rerio)

44655	4.31	0.22	0.0001	0.0018	2.39	all_v2.0.392.C1	DMP1 domain containing protein
55211	4.70	0.21	0.0001	0.0015	2.39	all_v2.0.6615.C1	unclassified
40905	3.17	0.31	0.0006	0.0065	2.38	sb_gmnlskic_0005p12.pDNRF2	PREDICTED: similar to putative pol protein; n=1; Danio rerio|Rep: PREDICTED: similar to putative pol protein - Danio rerio
48537	5.19	0.19	0.0000	0.0014	2.38	sb_gmnbhkas_0017n11.pDNRm13r	Unassigned protein
46805	3.71	0.28	0.0002	0.0030	2.36	all_v2.0.14762.C1	Unassigned protein
41202	2.45	0.46	0.0039	0.0232	2.36	all_v2.0.3558.C3	PREDICTED: similar to Vacuolar protein sorting 26 homolog (VPS26 protein homolog); n=1; Canis lupus familiaris|Rep: PREDICTED: similar to Vacuolar protein sorting 26 homolog (VPS26 protein homolog) - Canis familiaris
42908	3.43	0.27	0.0003	0.0043	2.34	all_v2.0.2624.C2	Ubiquitin carrier protein; n=1; Tetrahymena thermophila SB210|Rep: Ubiquitin carrier protein - Tetrahymena thermophila SB210
40983	2.47	0.39	0.0037	0.0222	2.34	sb_gmnbbr_0020h08.pDNRF2	PREDICTED: similar to Sec61, alpha subunit 2; n=1; Canis familiaris|Rep: PREDICTED: similar to Sec61, alpha subunit 2 - Canis familiaris
44665	4.75	0.20	0.0001	0.0015	2.34	all_v2.0.13567.C1	Drf_FH1 multi-domain protein
46543	4.13	0.24	0.0001	0.0020	2.33	sb_gmnbspic_0010p09.pDNRm13r	Unassigned protein
41335	4.21	0.23	0.0001	0.0019	2.32	all_v2.0.2624.C3	Probable bifunctional E2/E3 enzyme R795 [Includes: E3 ubiquitin- protein ligase (EC 6.3.2.-); Ubiquitin-conjugating enzyme E2 (EC 6.3.2.19)]; n=1; Acanthamoeba polyphaga mimivirus|Rep: Probable bifunctional E2/E3 enzyme R795 [Includes: E3 ubiquitin- protein ligase (EC 6.3.2.-); Ubiquitin-conjugating enzyme E2 (EC 6.3.2.19)] - Acanthamoeba polyphaga mimivirus (APMV)
42748	4.11	0.26	0.0001	0.0021	2.32	all_v2.0.9596.C1	Transmembrane protein 7; n=1; Siniperca chuatsi|Rep: Transmembrane protein 7 - Siniperca chuatsi (Chinese perch)
36093	2.60	0.33	0.0026	0.0176	2.31	sb_gmapte_0002b21.pDNRm13r	Baculoviral IAP repeat-containing 3; n=4; Otophysi|Rep: Baculoviral IAP repeat-containing 3 - Brachydanio rerio (Zebrafish) (Danio rerio)
38788	5.60	0.16	0.0000	0.0013	2.30	all_v2.0.1155.C2	Lily-type lectin; n=1; Platycephalus indicus|Rep: Lily-type lectin - Platycephalus indicus (bartail flathead)
41043	4.43	0.20	0.0001	0.0017	2.30	all_v2.0.1010.C10	PREDICTED: similar to stonustoxin alpha-subunit; n=3; Danio rerio|Rep: PREDICTED: similar to stonustoxin alpha-subunit - Danio rerio
43598	5.94	0.15	0.0000	0.0013	2.29	sb_gmnbmu_0001b07.pDNRm13r	Zgc:136346; n=7; Danio rerio|Rep: Zgc:136346 - Brachydanio rerio (Zebrafish) (Danio rerio)
43767	6.99	0.12	0.0000	0.0012	2.29	all_v2.0.8336.C1	Zgc:158652 protein; n=3; Danio rerio|Rep: Zgc:158652 protein - Danio rerio (Zebrafish) (Brachydanio rerio)
43007	4.01	0.24	0.0001	0.0022	2.28	sb_gmapov_0013f18.pDNRF2	UPI000065EC34 related cluster; n=1; Takifugu rubripes|Rep: UPI000065EC34 UniRef100 entry - Takifugu rubripes
53251	4.36	0.20	0.0001	0.0018	2.28	all_v2.0.13404.C1	unclassified
55397	5.60	0.15	0.0000	0.0013	2.27	all_v2.0.14900.C1	unclassified

43453	3.54	0.29	0.0003	0.0037	2.27	all_v2.0.13478.C1	Zgc:110652; n=1; Danio rerio|Rep: Zgc:110652 - Danio rerio (Zebrafish) (Brachydanio rerio)
39798	3.76	0.26	0.0002	0.0028	2.27	all_v2.0.8253.C1	Opioid growth factor receptor; n=2; Salmo salar|Rep: Opioid growth factor receptor - Salmo salar (Atlantic salmon)
48344	4.83	0.18	0.0001	0.0015	2.27	sb_gmnlkfas_0003n17.t7	Unassigned protein
44544	6.07	0.14	0.0000	0.0013	2.26	IRF1OK2	custom - Interferon regulatory factor 10K
47008	4.08	0.24	0.0001	0.0021	2.25	all_v2.0.13597.C1	Unassigned protein
51340	4.65	0.19	0.0001	0.0016	2.24	all_v2.0.12995.C1	unclassified
42585	5.27	0.16	0.0000	0.0014	2.23	all_v2.0.2335.C1	Titin a; n=3; Danio rerio|Rep: Titin a - Danio rerio (Zebrafish) (Brachydanio rerio)
37060	5.48	0.15	0.0000	0.0013	2.23	sb_gmnlsras_0003k23.t7	Eph-like receptor tyrosine kinase rtk5; n=4; Danio rerio|Rep: Eph-like receptor tyrosine kinase rtk5 - Danio rerio (Zebrafish) (Brachydanio rerio)
44884	3.68	0.30	0.0002	0.0031	2.22	sb_gmapov_0019d14.pDNRF2	Hexb; hexosaminidase B [EC:3.2.1.52]; K01207 beta-N-acetylhexosaminidase
41937	3.90	0.22	0.0001	0.0025	2.20	all_v2.0.7491.C2	RING finger protein 213.; n=1; Gallus gallus|Rep: RING finger protein 213 - Gallus gallus
36193	5.23	0.16	0.0000	0.0014	2.19	all_v2.0.661.C39	Bloodthirsty; n=2; Notothenioidei|Rep: Bloodthirsty - Chaenocephalus aceratus (White crocodile fish)
36525	4.12	0.21	0.0001	0.0021	2.19	all_v2.0.16620.C1	Claudin 30; n=1; Oreochromis mossambicus|Rep: Claudin 30 - Oreochromis mossambicus (Mozambique tilapia) (Tilapia mossambica)
37525	4.05	0.22	0.0001	0.0022	2.18	all_v2.0.3167.C1	H/ACA ribonucleoprotein complex subunit 3; n=1; Danio rerio|Rep: H/ACA ribonucleoprotein complex subunit 3 - Danio rerio (Zebrafish) (Brachydanio rerio)
47390	3.40	0.33	0.0004	0.0045	2.18	sb_gmnlem_0005d01.t7	Unassigned protein
45155	2.63	0.35	0.0024	0.0169	2.18	all_v2.0.13710.C1	LOC559696; similar to very large inducible GTPase 1
52620	2.91	0.33	0.0012	0.0103	2.17	all_v2.0.12870.C1	unclassified
46324	6.06	0.12	0.0000	0.0013	2.16	all_v2.0.16151.C1	TFIIS domain containing protein
35828	6.17	0.13	0.0000	0.0013	2.16	all_v2.0.10754.C1	AHA1, activator of heat shock protein ATPase homolog 1, like; n=2; Danio rerio|Rep: AHA1, activator of heat shock protein ATPase homolog 1, like - Danio rerio (Zebrafish) (Brachydanio rerio)
42822	2.39	0.34	0.0045	0.0259	2.15	all_v2.0.1349.C6	Tubulin beta-2C chain; n=84; Eukaryota|Rep: Tubulin beta-2C chain - Homo sapiens (Human)
45571	3.28	0.28	0.0005	0.0054	2.15	all_v2.0.16142.C1	non-directed
41954	3.78	0.24	0.0002	0.0028	2.15	all_v2.0.12540.C1	RNA polymerase II subunit 5-mediating protein (RPB5-mediating protein).; n=1; Takifugu rubripes|Rep: RNA polymerase II subunit 5-mediating protein (RPB5-mediating protein). - Takifugu rubripes
36338	5.47	0.14	0.0000	0.0013	2.14	all_v2.0.7453.C2	Caspase-1; n=1; Dicentrarchus labrax|Rep: Caspase-1 - Dicentrarchus labrax (European sea bass)
42676	3.56	0.27	0.0003	0.0036	2.13	all_v2.0.9799.C1	Transglutaminase; n=1; Oncorhynchus keta|Rep: Transglutaminase - Oncorhynchus keta (Chum salmon)
51517	4.74	0.18	0.0001	0.0015	2.13	all_v2.0.6615.C2	unclassified
41421	4.00	0.23	0.0001	0.0023	2.12	all_v2.0.1046.C1	Proteasome subunit alpha type-2; n=3; Xenopus|Rep: Proteasome subunit alpha

							type-2 - Xenopus laevis (African clawed frog)
44450	3.17	0.27	0.0006	0.0065	2.12	CK2c6	custom - CC chemokine
49723	7.11	0.10	0.0000	0.0012	2.12	all_v2.0.4732.C1	unclassified
42929	3.89	0.22	0.0001	0.0025	2.12	all_v2.0.473.C1	Ubiquitin-conjugating enzyme E2 D4; n=11; Euteleostomi|Rep: Ubiquitin-conjugating enzyme E2 D4 - Homo sapiens (Human)
36364	2.79	0.36	0.0016	0.0126	2.11	all_v2.0.8187.C1	Cathepsin L.1; n=1; Danio rerio|Rep: Cathepsin L.1 - Danio rerio (Zebrafish) (Brachydanio rerio)
40670	5.80	0.13	0.0000	0.0013	2.11	all_v2.0.14446.C1	PREDICTED: similar to mitochondrial carrier triple repeat 6; n=1; Monodelphis domestica|Rep: PREDICTED: similar to mitochondrial carrier triple repeat 6 - Monodelphis domestica
42754	4.12	0.22	0.0001	0.0021	2.11	sb_gmnbhkas_0008c11.pDNRF2	Transport-associated protein; n=3; Salmonidae|Rep: Transport-associated protein - Salmo salar (Atlantic salmon)
38355	5.68	0.13	0.0000	0.0013	2.11	all_v2.0.8243.C2	Homolog of Homo sapiens Spastic ataxia of Charlevoix-Saguenay; n=1; Takifugu rubripes|Rep: Homolog of Homo sapiens Spastic ataxia of Charlevoix-Saguenay - Takifugu rubripes
42021	4.29	0.19	0.0001	0.0018	2.11	all_v2.0.9962.C1	Sdf1a; n=2; Danio rerio|Rep: Sdf1a - Danio rerio (Zebrafish) (Brachydanio rerio)
44458	3.50	0.24	0.0003	0.0039	2.11	CK2c1	custom - CC chemokine
47827	5.15	0.15	0.0000	0.0014	2.11	sb_gmnbgi_0007a11.pDNRm13r	Unassigned protein
39240	4.05	0.20	0.0001	0.0022	2.10	all_v2.0.11987.C1	N-acylneuraminate-9-phosphatase (EC 3.1.3.29) (Neu5Ac-9-Pase) (Haloacid dehalogenase-like hydrolase domain-containing protein 4).; n=1; Takifugu rubripes|Rep: N-acylneuraminate-9-phosphatase (EC 3.1.3.29) (Neu5Ac-9-Pase) (Haloacid dehalogenase-like hydrolase domain-containing protein 4). - Takifugu rubripes
45143	7.37	0.09	0.0000	0.0012	2.10	all_v2.0.8322.C2	LOC540789; similar to poly (ADP-ribose) polymerase family, member 14
39845	3.58	0.24	0.0002	0.0035	2.09	all_v2.0.787.C2	Pallidin (Pallid protein homolog) (Syntaxin 13-interacting protein).; n=1; Takifugu rubripes|Rep: Pallidin (Pallid protein homolog) (Syntaxin 13-interacting protein). - Takifugu rubripes
54850	4.00	0.19	0.0001	0.0023	2.08	all_v2.0.2527.C1	unclassified
51353	4.55	0.18	0.0001	0.0016	2.08	all_v2.0.10898.C1	unclassified
37197	5.40	0.14	0.0000	0.0013	2.08	all_v2.0.12541.C1	Fc11b09; n=4; Danio rerio|Rep: Fc11b09 - Danio rerio (Zebrafish) (Brachydanio rerio)
36194	5.42	0.14	0.0000	0.0013	2.06	all_v2.0.12049.C1	Bloodthirsty; n=2; Notothenioidei|Rep: Bloodthirsty - Chaenocephalus aceratus (White crocodile fish)
37318	4.29	0.20	0.0001	0.0018	2.06	all_v2.0.10149.C6	Gamma-aminobutyric-acid receptor subunit alpha-1 precursor (GABA(A) receptor subunit alpha-1).; n=1; Takifugu rubripes|Rep: Gamma-aminobutyric-acid receptor subunit alpha-1 precursor (GABA(A) receptor subunit alpha-1). - Takifugu rubripes
40187	4.41	0.19	0.0001	0.0017	2.06	sb_gmapht_0020g02.pDNRm13r	PREDICTED: similar to actin-related protein 3-beta; n=1; Bos taurus|Rep: PREDICTED: similar to actin-related protein 3-beta - Bos taurus

39327	4.44	0.18	0.0001	0.0017	2.06	all_v2.0.12281.C1	NFX1-type zinc finger-containing protein 1.; n=1; Takifugu rubripes|Rep: NFX1-type zinc finger-containing protein 1 - Takifugu rubripes
41565	3.89	0.20	0.0001	0.0025	2.06	sb_gmnbhkic_0002c04.pDNRF2	Protein-tyrosine sulfotransferase 1; n=2; Danio rerio|Rep: Protein-tyrosine sulfotransferase 1 - Brachydanio rerio (Zebrafish) (Danio rerio)
35894	5.40	0.13	0.0000	0.0013	2.05	all_v2.0.16939.C1	Aminopeptidase N; n=2; Pseudopleuronectes americanus|Rep: Aminopeptidase N - Pseudopleuronectes americanus (Winter flounder) (Pleuronectesamericanus)
52667	5.92	0.12	0.0000	0.0013	2.05	all_v2.0.11918.C1	unclassified
38599	4.48	0.18	0.0001	0.0017	2.05	all_v2.0.16145.C1	unclassified
48556	4.94	0.16	0.0000	0.0014	2.05	sb_gmnbmd_0002p01.pDNRm13r	Unassigned protein
49763	4.95	0.16	0.0000	0.0014	2.05	all_v2.0.7999.C1	unclassified
39971	5.67	0.12	0.0000	0.0013	2.05	all_v2.0.10807.C1	phospholipase C delta 3; n=1; Takifugu rubripes|Rep: phospholipase C delta 3 - Takifugu rubripes
41116	5.87	0.12	0.0000	0.0013	2.04	all_v2.0.2090.C1	PREDICTED: similar to transposase; n=1; Danio rerio|Rep: PREDICTED: similar to transposase - Danio rerio
48803	2.73	0.28	0.0018	0.0142	2.04	sb_gmapte_0006b21.pDNRF2	Unassigned protein
41628	4.15	0.19	0.0001	0.0020	2.03	all_v2.0.10802.C1	Putative helicase MOV-10 (EC 3.6.1.-) (Moloney leukemia virus 10 protein).; n=1; Takifugu rubripes|Rep: Putative helicase MOV-10 (EC 3.6.1.-) (Moloney leukemia virus 10 protein). - Takifugu rubripes
44545	2.99	0.28	0.0009	0.0088	2.02	IRF1OK3	custom - Interferon regulatory factor 10K
43845	3.70	0.24	0.0002	0.0030	2.02	all_v2.0.18770.C1	Zgc:55983; n=5; Danio rerio|Rep: Zgc:55983 - Danio rerio (Zebrafish) (Brachydanio rerio)
44641	4.59	0.16	0.0001	0.0016	2.02	all_v2.0.3885.C1	DEC-1_N domain containing protein
44372	2.68	0.31	0.0021	0.0156	2.02	all_v2.0.2805.C3	caspc; caspase c
45349	5.37	0.13	0.0000	0.0013	2.02	all_v2.0.16273.C1	LOC793474; similar to stonustoxin alpha-subunit
50731	3.52	0.24	0.0003	0.0038	2.01	all_v2.0.17096.C1	unclassified
36994	3.01	0.28	0.0009	0.0085	2.01	sb_gmnlkfta_0002p12.t7	EIF4G-related protein NAT1A; n=4; Danio rerio|Rep: EIF4G-related protein NAT1A - Danio rerio (Zebrafish) (Brachydanio rerio)
40263	8.16	0.07	0.0000	0.0012	2.01	all_v2.0.661.C24	PREDICTED: similar to bloodthirsty; n=3; Danio rerio|Rep: PREDICTED: similar to bloodthirsty - Danio rerio
54894	4.49	0.18	0.0001	0.0017	2.01	all_v2.0.2012.C2	unclassified
42930	2.85	0.26	0.0014	0.0114	2.01	all_v2.0.1622.C1	Ubiquitin-conjugating enzyme E2 L3; n=31; Euteleostomi|Rep: Ubiquitin-conjugating enzyme E2 L3 - Homo sapiens (Human)
44911	4.11	0.18	0.0001	0.0021	2.01	all_v2.0.3709.C2	Ifi-6-16 multi-domain protein
48162	5.53	0.13	0.0000	0.0013	2.00	sb_gmnlpbia_0002b13.t7	Unassigned protein
46953	4.85	0.15	0.0001	0.0015	2.00	sb_gmnbhkic_0007o03.pDNRm13r	Unassigned protein
47493	3.85	0.19	0.0002	0.0026	2.00	sb_gmapht_0017g04.pDNRF2	Unassigned protein

47411	4.81	0.15	0.0001	0.0015	2.00	sb_gmnlskic_0010l20.pDNRm13r	Unassigned protein
49575	3.15	0.31	0.0006	0.0066	1.99	all_v2.0.3263.C1	unclassified
52110	4.21	0.17	0.0001	0.0019	1.99	all_v2.0.8876.C1	unclassified
36040	4.23	0.18	0.0001	0.0019	1.99	all_v2.0.11287.C1	ATPase family AAA domain-containing protein 1-B; n=2; Danio rerio|Rep: ATPase family AAA domain-containing protein 1-B - Danio rerio (Zebrafish) (Brachydanio rerio)
47749	4.63	0.15	0.0001	0.0016	1.99	sb_gmnbmu_0003o07.pDNRF2	Unassigned protein
38095	4.45	0.17	0.0001	0.0017	1.99	all_v2.0.6540.C1	Homolog of Homo sapiens Hemicentin; n=1; Takifugu rubripes|Rep: Homolog of Homo sapiens Hemicentin - Takifugu rubripes
41020	3.90	0.19	0.0001	0.0025	1.99	all_v2.0.6042.C1	PREDICTED: similar to sodium channel associated protein 1A; n=1; Strongylocentrotus purpuratus|Rep: PREDICTED: similar to sodium channel associated protein 1A - Strongylocentrotus purpuratus
50655	4.44	0.16	0.0001	0.0017	1.98	all_v2.0.5679.C1	unclassified
42555	5.36	0.13	0.0000	0.0014	1.98	sb_gmlbgits_0003f16.sp6	Tho2; n=5; Danio rerio|Rep: Tho2 - Danio rerio (Zebrafish) (Brachydanio rerio)
44598	6.07	0.11	0.0000	0.0013	1.98	STAT14	custom - Signal transducer and activator of transcription 1
53704	2.59	0.31	0.0026	0.0178	1.98	all_v2.0.1006.C1	unclassified
40019	3.07	0.30	0.0008	0.0077	1.98	sb_gmnbhkas_0006d14.pDNRF2	Pol polyprotein; n=2; Walleye epidermal hyperplasia virus 2|Rep: Pol polyprotein - Walleye epidermal hyperplasia virus 2
44713	3.61	0.20	0.0002	0.0034	1.98	all_v2.0.8260.C1	DUF1777 multi-domain protein
41181	3.76	0.22	0.0002	0.0029	1.97	all_v2.0.14519.C1	PREDICTED: similar to Ubiquitin carboxyl-terminal hydrolase 5 (Ubiquitin thioesterase 5) (Ubiquitin-specific-processing protease 5) (Deubiquitinating enzyme 5) (Isopeptidase T); n=2; Homo/Pan/Gorilla group|Rep: PREDICTED: similar to Ubiquitin carboxyl-terminal hydrolase 5 (Ubiquitin thioesterase 5) (Ubiquitin-specific-processing protease 5) (Deubiquitinating enzyme 5) (Isopeptidase T) - Homo sapiens
40304	4.17	0.17	0.0001	0.0020	1.97	all_v2.0.2720.C1	PREDICTED: similar to CDK2 (cyclin-dependent kinase 2)-associated protein 1; n=2; Canis lupus familiaris|Rep: PREDICTED: similar to CDK2 (cyclin-dependent kinase 2)-associated protein 1 - Canis familiaris
42085	2.89	0.25	0.0012	0.0105	1.97	all_v2.0.889.C1	Serine/threonine-protein kinase Chk2 (EC 2.7.11.1) (Cds1).; n=1; Takifugu rubripes|Rep: Serine/threonine-protein kinase Chk2 (EC 2.7.11.1) (Cds1). - Takifugu rubripes
52069	4.32	0.17	0.0001	0.0018	1.96	all_v2.0.6954.C2	unclassified
39626	6.77	0.09	0.0000	0.0012	1.96	all_v2.0.4.CB27	Novel protein; n=2; Danio rerio|Rep: Novel protein - Danio rerio (Zebrafish) (Brachydanio rerio)
39251	4.94	0.13	0.0000	0.0014	1.95	all_v2.0.4751.C2	NADH dehydrogenase (Ubiquinone) flavoprotein 1; n=3; Clupeocephala|Rep: NADH dehydrogenase (Ubiquinone) flavoprotein 1 - Danio rerio (Zebrafish) (Brachydanio rerio)

41936	3.46	0.22	0.0003	0.0041	1.95	all_v2.0.7491.C3	RING finger protein 213.; n=1; Gallus gallus|Rep: RING finger protein 213 - Gallus gallus
41491	6.55	0.09	0.0000	0.0012	1.95	all_v2.0.871.C1	Protein mago nashi homolog 2; n=25; Coelomata|Rep: Protein mago nashi homolog 2 - Homo sapiens (Human)
43221	4.04	0.19	0.0001	0.0022	1.95	all_v2.0.10924.C1	Voltage-dependent N-type calcium channel subunit alpha-1B (Voltage- gated calcium channel subunit alpha Cav2.2) (Calcium channel, L type, alpha-1 polypeptide isoform 5) (Brain calcium channel III) (BIII).; n=1; Takifugu rubripes|Rep: Voltage-dependent N-type calcium channel subunit alpha-1B (Voltage- gated calcium channel subunit alpha Cav2.2) (Calcium channel, L type, alpha-1 polypeptide isoform 5) (Brain calcium channel III) (BIII). - Takifugu rubripes
36988	4.44	0.16	0.0001	0.0017	1.95	all_v2.0.3557.C1	Eif4a1b protein; n=2; Danio rerio|Rep: Eif4a1b protein - Danio rerio (Zebrafish) (Brachydanio rerio)
45068	4.40	0.16	0.0001	0.0017	1.94	all_v2.0.16837.C1	LOC428660; similar to very large inducible GTPase-1
51421	5.35	0.12	0.0000	0.0014	1.94	all_v2.0.4626.C1	unclassified
44865	6.05	0.10	0.0000	0.0013	1.94	all_v2.0.8362.C1	HERC5, LOC478474; hect domain and RLD 5
52910	4.40	0.16	0.0001	0.0017	1.94	all_v2.0.999.C1	unclassified
39348	3.38	0.20	0.0004	0.0046	1.94	all_v2.0.3973.C2	N-myc-interactor (Nmi) (N-myc and STAT interactor).; n=1; Takifugu rubripes|Rep: N-myc-interactor (Nmi) (N-myc and STAT interactor). - Takifugu rubripes
36407	6.64	0.09	0.0000	0.0012	1.93	all_v2.0.1864.C7	CD9 protein; n=1; Oncorhynchus mykiss|Rep: CD9 protein - Oncorhynchus mykiss (Rainbow trout) (Salmo gairdneri)
47299	4.49	0.16	0.0001	0.0017	1.93	sb_gmnbhkic_0005d21.pDNRF2	Unassigned protein
51818	4.71	0.15	0.0001	0.0015	1.93	all_v2.0.11455.C1	unclassified
36426	3.72	0.19	0.0002	0.0029	1.93	all_v2.0.8306.C2	Cell division cycle 42; n=12; Coelomata|Rep: Cell division cycle 42 - Danio rerio (Zebrafish) (Brachydanio rerio)
44503	4.02	0.18	0.0001	0.0022	1.93	PKRvA2	custom - double-stranded RNA-dependent protein kinase A
41109	4.87	0.14	0.0001	0.0014	1.92	sb_gmnbmd_0005c12.pDNRm13r	PREDICTED: similar to transposase; n=1; Danio rerio|Rep: PREDICTED: similar to transposase - Danio rerio
41315	2.76	0.30	0.0017	0.0134	1.92	all_v2.0.5598.C2	Probable ATP-dependent helicase LGP2 (EC 3.6.1.-) (Protein D11Lgp2 homolog).; n=1; Takifugu rubripes|Rep: Probable ATP-dependent helicase LGP2 (EC 3.6.1.-) (Protein D11Lgp2 homolog). - Takifugu rubripes
37946	2.91	0.30	0.0011	0.0102	1.92	all_v2.0.2234.C1	Homolog of Gallus gallus Voltage-dependent anion channel.; n=1; Takifugu rubripes|Rep: Homolog of Gallus gallus Voltage-dependent anion channel. - Takifugu rubripes
38178	5.46	0.12	0.0000	0.0013	1.91	all_v2.0.14775.C1	Homolog of Homo sapiens Mucin 2 precursor; n=1; Takifugu rubripes|Rep: Homolog of Homo sapiens Mucin 2 precursor - Takifugu rubripes
38352	4.95	0.13	0.0000	0.0014	1.90	all_v2.0.272.C2	Homolog of Homo sapiens SOUL protein; n=1; Takifugu rubripes|Rep: Homolog of Homo sapiens SOUL protein - Takifugu rubripes

42922	5.43	0.11	0.0000	0.0013	1.90	sb_gmnlpras_0001f12.t7	Ubiquitin; n=6; Eukaryota|Rep: Ubiquitin - Tetrahymena pyriformis
42678	4.33	0.15	0.0001	0.0018	1.90	all_v2.0.9799.C2	Transglutaminase; n=1; Oreochromis niloticus|Rep: Transglutaminase - Oreochromis niloticus (Nile tilapia) (Tilapia nilotica)
43012	5.77	0.10	0.0000	0.0013	1.90	all_v2.0.9212.C1	UPI0000D8BA1C related cluster; n=1; Danio rerio|Rep: UPI0000D8BA1C UniRef100 entry - Danio rerio
49141	4.05	0.17	0.0001	0.0022	1.90	all_v2.0.14559.C1	unclassified
49317	4.89	0.13	0.0001	0.0014	1.90	all_v2.0.13805.C1	unclassified
38891	3.13	0.22	0.0007	0.0069	1.90	all_v2.0.1124.C3	Lysyl-tRNA synthetase; n=3; Danio rerio|Rep: Lysyl-tRNA synthetase - Danio rerio (Zebrafish) (Brachydanio rerio)
38436	5.31	0.12	0.0000	0.0014	1.90	sb_gmapov_0005h22.pDNRF2	Homolog of Homo sapiens Vacuolar sorting protein 54 long isoform; n=1; Takifugu rubripes|Rep: Homolog of Homo sapiens Vacuolar sorting protein 54 long isoform - Takifugu rubripes
39454	4.77	0.14	0.0001	0.0015	1.90	sb_gmapov_0007h10.pDNRF2	Novel protein similar to vertebrate ATPase, H+ transporting, lysosomal V0 subunit a isoform 2; n=3; Danio rerio|Rep: Novel protein similar to vertebrate ATPase, H+ transporting, lysosomal V0 subunit a isoform 2 - Brachydanio rerio (Zebrafish) (Danio rerio)
53326	2.66	0.26	0.0022	0.0160	1.90	all_v2.0.9151.C1	unclassified
45375	4.10	0.17	0.0001	0.0021	1.89	sb_gmnbmu_0008h11.pDNRF2	Macro domain containing protein
46296	3.86	0.18	0.0002	0.0026	1.89	sb_gmnbhkic_0001i02.pDNRF2	ST7 domain containing protein
45146	5.24	0.11	0.0000	0.0014	1.89	sb_gmnbhkic_0005i22.pDNRm13r	LOC555735; similar to DNA methyltransferase
36949	2.69	0.28	0.0020	0.0151	1.89	all_v2.0.3352.C2	Dual specificity protein phosphatase 21 (EC 3.1.3.48) (EC 3.1.3.16) (Low molecular weight dual specificity phosphatase 21).; n=1; Takifugu rubripes|Rep: Dual specificity protein phosphatase 21 (EC 3.1.3.48) (EC 3.1.3.16) (Low molecular weight dual specificity phosphatase 21). - Takifugu rubripes
44434	4.70	0.14	0.0001	0.0015	1.89	CASP82	custom - caspase 8, apoptosis-related cysteine peptidase
44509	4.08	0.16	0.0001	0.0021	1.88	PKRvB5	custom - double-stranded RNA-dependent protein kinase B
43029	3.72	0.20	0.0002	0.0029	1.88	sb_gmapht_0005e07.pDNRF2	UPI0000D8C4C4 related cluster; n=2; Danio rerio|Rep: UPI0000D8C4C4 UniRef100 entry - Danio rerio
51332	5.02	0.13	0.0000	0.0014	1.88	all_v2.0.4629.C1	unclassified
47248	2.67	0.38	0.0022	0.0159	1.88	all_v2.0.3503.C1	Unassigned protein
41526	4.50	0.15	0.0001	0.0017	1.88	sb_gmapte_0011n16.pDNRF2	Protein ripply1; n=2; Danio rerio|Rep: Protein ripply1 - Danio rerio (Zebrafish) (Brachydanio rerio)
40844	5.49	0.11	0.0000	0.0013	1.88	all_v2.0.8322.C3	PREDICTED: similar to Poly [ADP-ribose] polymerase 14 (PARP-14) (B aggressive lymphoma protein 2); n=1; Danio rerio|Rep: PREDICTED: similar to Poly [ADP-ribose] polymerase 14 (PARP-14) (B aggressive lymphoma protein 2) - Danio rerio
45719	2.85	0.27	0.0013	0.0114	1.88	all_v2.0.15866.C1.a	non-directed

53053	4.58	0.14	0.0001	0.0016	1.88	all_v2.0.393.C1	unclassified
47291	6.55	0.08	0.0000	0.0012	1.87	all_v2.0.606.C2	Unassigned protein
41395	3.88	0.17	0.0002	0.0025	1.87	all_v2.0.7668.C1	prospero homeobox 2; n=1; Takifugu rubripes|Rep: prospero homeobox 2 - Takifugu rubripes
36797	5.23	0.11	0.0000	0.0014	1.87	all_v2.0.2166.C2	Cytotoxic and regulatory T cell protein; n=1; Oncorhynchus mykiss|Rep: Cytotoxic and regulatory T cell protein - Oncorhynchus mykiss (Rainbow trout) (Salmo gairdneri)
40875	2.71	0.28	0.0019	0.0147	1.86	sb_gmnbhkic_0015m19.pDNRm13r	PREDICTED: similar to protein BAP28 (FLJ10359); n=1; Monodelphis domestica|Rep: PREDICTED: similar to protein BAP28 (FLJ10359) - Monodelphis domestica
42292	4.20	0.16	0.0001	0.0020	1.86	sb_gmnbbr_0003o15.pDNRF2	Solute carrier family 25 (Carnitine/acylcarnitine translocase), member 20; n=1; Danio rerio|Rep: Solute carrier family 25 (Carnitine/acylcarnitine translocase), member 20 - Brachydanio rerio (Zebrafish) (Danio rerio)
51931	3.48	0.20	0.0003	0.0040	1.86	all_v2.0.6205.C1	unclassified
49851	3.02	0.27	0.0009	0.0084	1.86	all_v2.0.2837.C3	unclassified
40721	2.60	0.29	0.0026	0.0176	1.86	sb_gmnbpcic_0001i03.pDNRF2	PREDICTED: similar to Nitric oxide synthase trafficker; n=1; Danio rerio|Rep: PREDICTED: similar to Nitric oxide synthase trafficker - Danio rerio
41414	5.04	0.12	0.0000	0.0014	1.86	sb_gmnlsfic_0008p05.t7	Proteasome subunit alpha type 6-like protein; n=1; Xenopsylla cheopis|Rep: Proteasome subunit alpha type 6-like protein - Xenopsylla cheopis (oriental rat flea)
41104	4.07	0.17	0.0001	0.0021	1.86	all_v2.0.11473.C1	PREDICTED: similar to transposase, partial; n=2; Strongylocentrotus purpuratus|Rep: PREDICTED: similar to transposase, partial - Strongylocentrotus purpuratus
41047	2.78	0.31	0.0016	0.0127	1.85	sb_gmapht_0032p07.pDNRm13r	PREDICTED: similar to stonustoxin alpha-subunit; n=3; Danio rerio|Rep: PREDICTED: similar to stonustoxin alpha-subunit - Danio rerio
54199	2.87	0.26	0.0013	0.0110	1.85	all_v2.0.7185.C1	unclassified
40337	3.96	0.17	0.0001	0.0023	1.85	sb_gmnlbric_0003k18.t7	PREDICTED: similar to cGMP phosphodiesterase A2; n=1; Ornithorhynchus anatinus|Rep: PREDICTED: similar to cGMP phosphodiesterase A2 - Ornithorhynchus anatinus
54583	2.80	0.28	0.0015	0.0124	1.85	all_v2.0.1321.C2	unclassified
47434	2.43	0.28	0.0041	0.0239	1.85	sb_gmnbhkas_0017h13.pDNRm13r	Unassigned protein
43297	4.96	0.12	0.0000	0.0014	1.84	all_v2.0.11507.C2	Zgc:101040; n=1; Danio rerio|Rep: Zgc:101040 - Danio rerio (Zebrafish) (Brachydanio rerio)
39658	3.02	0.24	0.0008	0.0083	1.84	all_v2.0.5240.C2	Novel protein; n=3; Danio rerio|Rep: Novel protein - Danio rerio (Zebrafish) (Brachydanio rerio)
45753	3.22	0.22	0.0005	0.0059	1.84	all_v2.0.4658.C1.a	non-directed
55314	4.74	0.13	0.0001	0.0015	1.83	all_v2.0.4690.C2	unclassified
45838	3.64	0.19	0.0002	0.0032	1.83	all_v2.0.13440.C1	non-directed
35725	4.65	0.13	0.0001	0.0015	1.83	all_v2.0.15785.	Abhydrolase domain containing 4; n=2;

						C1	Danio rerio|Rep: Abhydrolase domain containing 4 - Danio rerio (Zebrafish) (Brachydanio rerio)
40171	3.41	0.22	0.0003	0.0044	1.83	sb_gmnbhkas_0001a01.pDNRF2	PREDICTED: similar to 5-amp-activated protein kinase, beta subunit; n=1; Strongylocentrotus purpuratus|Rep: PREDICTED: similar to 5-amp-activated protein kinase, beta subunit - Strongylocentrotus purpuratus
47211	2.49	0.30	0.0034	0.0213	1.82	all_v2.0.8780.C1	Unassigned protein
39246	3.54	0.18	0.0003	0.0037	1.82	all_v2.0.7214.C1	NADH dehydrogenase (Ubiquinone) 1 beta subcomplex, 10; n=2; Danio rerio|Rep: NADH dehydrogenase (Ubiquinone) 1 beta subcomplex, 10 - Danio rerio (Zebrafish) (Brachydanio rerio)
39011	2.61	0.27	0.0026	0.0176	1.82	sb_gmapht_0006h11.pDNRF2	MGC69539 protein; n=2; Xenopus|Rep: MGC69539 protein - Xenopus tropicalis (Western clawed frog) (Silurana tropicalis)
45986	5.92	0.09	0.0000	0.0013	1.82	all_v2.0.10798.C1	pfkp; phosphofructokinase, platelet [EC:2.7.1.11]; K00850 6-phosphofructokinase
43487	4.96	0.12	0.0000	0.0014	1.82	all_v2.0.5034.C1	Zgc:112064; n=4; Danio rerio|Rep: Zgc:112064 - Danio rerio (Zebrafish) (Brachydanio rerio)
47368	5.87	0.09	0.0000	0.0013	1.81	sb_gmnlla_0036m01.t7	Unassigned protein
35854	4.90	0.12	0.0001	0.0014	1.81	all_v2.0.1645.C1	Alkylated repair protein alkB homolog 5.; n=2; Gallus gallus|Rep: Alkylated repair protein alkB homolog 5 - Gallus gallus
50348	3.66	0.18	0.0002	0.0032	1.81	all_v2.0.9352.C1	unclassified
41899	4.72	0.13	0.0001	0.0015	1.81	all_v2.0.16108.C1	Ribosomal protein L5b; n=1; Ictalurus punctatus|Rep: Ribosomal protein L5b - Ictalurus punctatus (Channel catfish)
51453	3.98	0.17	0.0001	0.0023	1.81	all_v2.0.1144.C1	unclassified
37698	5.19	0.10	0.0000	0.0014	1.81	sb_gmnbpcic_0001e04.pDNRm13r	Homolog of Brachydanio rerio Cyclin L ania-6a.; n=2; Takifugu rubripes|Rep: Homolog of Brachydanio rerio Cyclin L ania-6a. - Takifugu rubripes
36366	3.85	0.16	0.0002	0.0026	1.81	all_v2.0.1055.C1	Cathepsin L; n=2; Percomorpha|Rep: Cathepsin L - Lates calcarifer (Barramundi)
40660	2.55	0.29	0.0029	0.0192	1.80	sb_gmnlpbia_0002a22.t7	PREDICTED: similar to MGC81823 protein isoform 1; n=2; Strongylocentrotus purpuratus|Rep: PREDICTED: similar to MGC81823 protein isoform 1 - Strongylocentrotus purpuratus
41633	5.46	0.10	0.0000	0.0013	1.80	sb_gmnbspic_0007m23.pDNRF2	Putative methyltransferase METT10D; n=3; Danio rerio|Rep: Putative methyltransferase METT10D - Danio rerio (Zebrafish) (Brachydanio rerio)
48076	3.29	0.21	0.0005	0.0053	1.80	sb_gmnbspic_0005b22.pDNRF2	Unassigned protein
43642	4.33	0.14	0.0001	0.0018	1.80	all_v2.0.5071.C1	Zgc:152953; n=2; Danio rerio|Rep: Zgc:152953 - Danio rerio (Zebrafish) (Brachydanio rerio)
50281	2.51	0.30	0.0033	0.0209	1.80	all_v2.0.7444.C1	unclassified
53852	3.70	0.18	0.0002	0.0030	1.80	all_v2.0.6339.C1	unclassified
44839	2.92	0.26	0.0011	0.0100	1.79	all_v2.0.5182.C2	GRP58; glucose regulated protein 58kD [EC:5.3.4.1]; K08056 protein disulfide isomerase family A, member 3
50391	5.67	0.09	0.0000	0.0013	1.79	all_v2.0.2069.C2	unclassified
37904	3.62	0.18	0.0002	0.0033	1.79	sb_gmnbbrts_0	Homolog of Gallus gallus Double-

						003k13.pDNRF2	stranded RNA-activated protein kinase.; n=1; Takifugu rubripes|Rep: Homolog of Gallus gallus Double-stranded RNA-activated protein kinase. - Takifugu rubripes
44396	2.72	0.24	0.0019	0.0145	1.79	all_v2.0.6100.C1	CHCHD5; coiled-coil-helix-coiled-coil-helix domain containing 5
43362	4.26	0.14	0.0001	0.0019	1.79	sb_gmnbmu_0012l15.pDNRF2	Zgc:103426; n=2; Danio rerio|Rep: Zgc:103426 - Danio rerio (Zebrafish) (Brachydanio rerio)
52340	4.96	0.11	0.0000	0.0014	1.78	all_v2.0.3337.C1	unclassified
46157	2.47	0.35	0.0037	0.0223	1.78	sb_gmnbhkas_0009m02.pDNRm13r	RGD1564081_predicted; similar to novel protein similar to human oligophrenin 1 (OPHN1) (predicted)
44355	2.78	0.23	0.0016	0.0127	1.78	sb_gmnblits_0009a24.pDNRm13r	Caldesmon domain containing protein
40847	4.70	0.12	0.0001	0.0015	1.78	all_v2.0.8322.C1	PREDICTED: similar to Poly [ADP-ribose] polymerase 14 (PARP-14) (B aggressive lymphoma protein 2); n=1; Danio rerio|Rep: PREDICTED: similar to Poly [ADP-ribose] polymerase 14 (PARP-14) (B aggressive lymphoma protein 2) - Danio rerio
49782	4.42	0.14	0.0001	0.0017	1.78	all_v2.0.17197.C1	unclassified
46402	4.79	0.11	0.0001	0.0015	1.78	sb_gmapht_0004b19.pDNRm13r	TTN; cardiac titin
45976	3.28	0.19	0.0005	0.0054	1.77	sb_gmnlkfta_0007d22.t7	pbef1; pre-B-cell colony enhancing factor 1
39321	3.36	0.18	0.0004	0.0047	1.77	all_v2.0.6027.C5	NFX1-type zinc finger-containing protein 1.; n=1; Takifugu rubripes|Rep: NFX1-type zinc finger-containing protein 1 - Takifugu rubripes
40679	3.71	0.16	0.0002	0.0030	1.77	all_v2.0.4387.C1	PREDICTED: similar to mKIAA1931 protein; n=1; Monodelphis domestica|Rep: PREDICTED: similar to mKIAA1931 protein - Monodelphis domestica
45352	3.15	0.19	0.0006	0.0067	1.77	sb_gmnbmd_0007i19.pDNRF2	LOC793817; similar to pol polyprotein
43795	3.63	0.16	0.0002	0.0033	1.77	all_v2.0.2978.C1	Zgc:163136 protein; n=1; Danio rerio|Rep: Zgc:163136 protein - Danio rerio (Zebrafish) (Brachydanio rerio)
53004	5.48	0.09	0.0000	0.0013	1.77	all_v2.0.547.C1	unclassified
43200	4.42	0.13	0.0001	0.0017	1.77	sb_gmnlsfic_0002a03.t7	Viperin-like protein; n=1; Channa argus|Rep: Viperin-like protein - Channa argus
50754	5.59	0.09	0.0000	0.0013	1.77	all_v2.0.5172.C1	unclassified
43361	3.34	0.18	0.0004	0.0049	1.77	all_v2.0.2975.C2	Zgc:103420; n=3; Cyprinidae|Rep: Zgc:103420 - Danio rerio (Zebrafish) (Brachydanio rerio)
44956	4.11	0.14	0.0001	0.0021	1.77	all_v2.0.18221.C1	KRAS, LOC418207; v-Ki-ras2 Kirsten rat sarcoma viral oncogene homolog; K07827 GTPase Kras
42223	3.30	0.20	0.0004	0.0053	1.77	all_v2.0.3958.C1	Single-stranded DNA-binding protein, mitochondrial precursor (Mt-SSB) (MtSSB) (PWP1-interacting protein 17).; n=1; Takifugu rubripes|Rep: Single-stranded DNA-binding protein, mitochondrial precursor (Mt-SSB) (MtSSB) (PWP1-interacting protein 17). - Takifugu rubripes
55446	2.93	0.22	0.0011	0.0098	1.77	sb_gmapov_0017j09.pDNRF2	USP22, USP3L; ubiquitin specific peptidase 22
48129	3.42	0.18	0.0003	0.0043	1.77	all_v2.0.14003.C1	Unassigned protein

39405	4.19	0.14	0.0001	0.0020	1.76	all_v2.0.6252.C1	Novel protein similar to caspase 8; n=3; Danio rerio|Rep: Novel protein similar to caspase 8 - Danio rerio (Zebrafish) (Brachydanio rerio)
49744	3.13	0.21	0.0007	0.0069	1.76	all_v2.0.9839.C1	unclassified
54951	3.39	0.18	0.0004	0.0045	1.76	all_v2.0.1643.C2	unclassified
44123	3.07	0.19	0.0008	0.0077	1.75	all_v2.0.8502.C1	Zinc finger CCHC domain-containing protein 9.; n=1; Takifugu rubripes|Rep: Zinc finger CCHC domain-containing protein 9 - Takifugu rubripes
44472	2.69	0.25	0.0020	0.0152	1.75	2AY614592_Gadus morhua CC chemokine type 3 mRNA, complete cds	custom - CC chemokine
37707	6.54	0.06	0.0000	0.0012	1.75	sb_gmapov_0029c16.pDNRF2	Homolog of Brachydanio rerio Fem1c.; n=1; Takifugu rubripes|Rep: Homolog of Brachydanio rerio Fem1c. - Takifugu rubripes
53189	4.33	0.13	0.0001	0.0018	1.74	all_v2.0.11205.C1	unclassified
41492	4.28	0.13	0.0001	0.0019	1.74	sb_gmnbgi_0004b13.pDNRF2	Protein MAK10 homolog; n=4; Clupeocephala|Rep: Protein MAK10 homolog - Danio rerio (Zebrafish) (Brachydanio rerio)
54741	3.77	0.16	0.0002	0.0028	1.74	all_v2.0.735.C1	unclassified
44518	3.83	0.15	0.0002	0.0027	1.74	FAAD5c	custom - Fas (TNFRSF6)-associated via death domain
48549	3.47	0.17	0.0003	0.0041	1.74	all_v2.0.8578.C1	Unassigned protein
42224	2.72	0.23	0.0019	0.0145	1.74	sb_gmnbhkic_0017j15.pDNRm13r	Sister chromatid cohesion protein PDS5 homolog A; n=8; Danio rerio|Rep: Sister chromatid cohesion protein PDS5 homolog A - Danio rerio (Zebrafish) (Brachydanio rerio)
41409	3.16	0.19	0.0006	0.0066	1.74	all_v2.0.379.C1	Proteasome activator subunit 2; n=3; Clupeocephala|Rep: Proteasome activator subunit 2 - Danio rerio (Zebrafish) (Brachydanio rerio)
40930	3.29	0.19	0.0005	0.0053	1.74	sb_gmnbspic_0012b01.pDNRF2	PREDICTED: similar to retinoblastoma-associated protein 140; n=2; Canis lupus familiaris|Rep: PREDICTED: similar to retinoblastoma-associated protein 140 - Canis familiaris
41393	3.24	0.19	0.0005	0.0057	1.73	sb_gmnbhkic_0014b13.pDNRF2	Proprotein convertase subtilisin/kexin type 5 precursor (EC 3.4.21.-) (Proprotein convertase PC5) (Subtilisin/kexin-like protease PC5) (PC6) (hPC6).; n=1; Takifugu rubripes|Rep: Proprotein convertase subtilisin/kexin type 5 precursor (EC 3.4.21.-) (Proprotein convertase PC5) (Subtilisin/kexin-like protease PC5) (PC6) (hPC6). - Takifugu rubripes
46415	3.17	0.19	0.0006	0.0065	1.73	sb_gmnlla_0040g13.t7	U-edititng enzyme APOBEC-2 gb|AAD45361.1|AF161699_1 APOBEC-2 protein [Mus musculus] gb|AAH27530.1| Apolipoprotein B editing complex 2 [Mus musculus] gb|EDL23623.1| apolipoprotein B editing complex 2, isoform CRA_b [Mus musculus]
52140	4.30	0.13	0.0001	0.0018	1.73	all_v2.0.2506.C1	unclassified
53350	3.38	0.17	0.0004	0.0045	1.73	all_v2.0.16720.C1	unclassified
44520	3.19	0.19	0.0006	0.0063	1.73	FAAD7c	custom - Fas (TNFRSF6)-associated via death domain

50298	3.74	0.15	0.0002	0.0029	1.73	all_v2.0.17232.C1	unclassified
38536	4.11	0.13	0.0001	0.0021	1.72	all_v2.0.5336.C3	Hypoxanthine-guanine phosphoribosyltransferase; n=11; Euteleostomi|Rep: Hypoxanthine-guanine phosphoribosyltransferase - Gallus gallus (Chicken)
51377	3.96	0.15	0.0001	0.0023	1.72	all_v2.0.2972.C1	unclassified
38155	4.12	0.14	0.0001	0.0021	1.72	sb_gmapov_0016d17.pDNRF2	Homolog of Homo sapiens Mannosyl (Alpha-1,3-)-glycoprotein beta-1,4-N-acetylglucosaminyltransferase, isoenzyme B, isoform 1; n=2; Clupeocephala|Rep: Homolog of Homo sapiens Mannosyl (Alpha-1,3-)-glycoprotein beta-1,4-N-acetylglucosaminyltransferase, isoenzyme B, isoform 1 - Takifugu rubripes
41943	4.46	0.12	0.0001	0.0017	1.72	all_v2.0.4717.C1	Ring finger protein 4; n=4; Xenopus tropicalis|Rep: Ring finger protein 4 - Xenopus tropicalis (Western clawed frog) (Silurana tropicalis)
45461	3.91	0.15	0.0001	0.0024	1.71	sb_gmnlskic_0001f09.pDNRF2	Mucin multi-domain protein
37349	3.91	0.14	0.0001	0.0024	1.71	all_v2.0.996.C1	Geminin; n=1; Oryzias latipes|Rep: Geminin - Oryzias latipes (Medaka fish) (Japanese ricefish)
38781	2.85	0.22	0.0014	0.0114	1.71	all_v2.0.5859.C3	Leukotriene A-4 hydrolase (EC 3.3.2.6) (LTA-4 hydrolase) (Leukotriene A(4) hydrolase).; n=1; Takifugu rubripes|Rep: Leukotriene A-4 hydrolase (EC 3.3.2.6) (LTA-4 hydrolase) (Leukotriene A(4) hydrolase). - Takifugu rubripes
54462	3.35	0.17	0.0004	0.0049	1.71	all_v2.0.4285.C1	unclassified
36901	6.41	0.06	0.0000	0.0013	1.70	all_v2.0.5271.C1	DNA-directed RNA polymerase I subunit RPA49 (RNA polymerase I- associated factor 53) (RNA polymerase I polypeptide E) (RNA polymerase I-associated factor 1) (A49).; n=1; Takifugu rubripes|Rep: DNA-directed RNA polymerase I subunit RPA49 (RNA polymerase I- associated factor 53) (RNA polymerase I polypeptide E) (RNA polymerase I-associated factor 1) (A49). - Takifugu rubripes
48330	3.11	0.20	0.0007	0.0071	1.70	sb_gmnlla_0031j13.t7	Unassigned protein
51243	3.82	0.14	0.0002	0.0027	1.70	all_v2.0.1415.C3	unclassified
46430	4.23	0.12	0.0001	0.0019	1.70	all_v2.0.14645.C1	Unassigned protein
45980	3.67	0.15	0.0002	0.0031	1.70	sb_gmnbmd_0011d22.pDNRF2	PDZ domain containing 2 [Mus musculus]
40262	5.57	0.08	0.0000	0.0013	1.70	sb_gmapht_0030a18.pDNRF2	PREDICTED: similar to bloodthirsty; n=2; Danio rerio|Rep: PREDICTED: similar to bloodthirsty - Danio rerio
49026	3.30	0.16	0.0004	0.0052	1.70	all_v2.0.270.C2	Unassigned protein
43079	6.93	0.05	0.0000	0.0012	1.69	sb_gmapht_0004g05.pDNRF2	UPI0000E4E275 related cluster; n=1; Danio rerio|Rep: UPI0000E4E275 UniRef100 entry - Danio rerio
42012	3.51	0.17	0.0003	0.0038	1.69	sb_gmnbbrts_0003p08.pDNRF2	Sarcoglycan, beta; n=2; Danio rerio|Rep: Sarcoglycan, beta - Brachydanio rerio (Zebrafish) (Danio rerio)
41827	3.85	0.15	0.0002	0.0026	1.69	sb_gmapov_0017j24.pDNRF2	Rfc5 protein; n=5; Clupeocephala|Rep: Rfc5 protein - Brachydanio rerio (Zebrafish) (Danio rerio)
43941	3.57	0.16	0.0003	0.0036	1.69	all_v2.0.1382.C1	Zgc:73375; n=1; Danio rerio|Rep: Zgc:73375 - Danio rerio (Zebrafish) (Brachydanio rerio)
48479	3.15	0.18	0.0006	0.0067	1.69	all_v2.0.6321.C4	Unassigned protein

36757	3.54	0.15	0.0003	0.0036	1.68	all_v2.0.1015.C1	Cytochrome c oxidase subunit VIa polypeptide 1; n=2; Danio rerio|Rep: Cytochrome c oxidase subunit VIa polypeptide 1 - Danio rerio (Zebrafish) (Brachydanio rerio)
49516	4.09	0.13	0.0001	0.0021	1.68	all_v2.0.14907.C1	unclassified
43681	3.19	0.19	0.0006	0.0063	1.68	all_v2.0.6826.C2	Zgc:153440; n=3; Danio rerio|Rep: Zgc:153440 - Danio rerio (Zebrafish) (Brachydanio rerio)
36051	5.16	0.09	0.0000	0.0014	1.68	all_v2.0.15356.C1	ATPase, Na+/K+ transporting, alpha 3 polypeptide; n=5; Tetrapoda|Rep: ATPase, Na+/K+ transporting, alpha 3 polypeptide - Mus musculus (Mouse)
45123	2.88	0.21	0.0013	0.0108	1.68	all_v2.0.13960.C1	LOC501925; similar to solute carrier family 2, member 9 isoform a; K08146 MFS transporter, SP family, solute carrier family 2 (facilitated glucose transporter), member 9
55134	4.19	0.12	0.0001	0.0020	1.68	all_v2.0.392.C2	unclassified
36121	3.78	0.14	0.0002	0.0028	1.67	all_v2.0.5784.C1	Bcl2l10 protein; n=2; Danio rerio|Rep: Bcl2l10 protein - Danio rerio (Zebrafish) (Brachydanio rerio)
47158	3.96	0.13	0.0001	0.0023	1.67	sb_gmnbhkas_0007l19.pDNRF2	Unassigned protein
36286	4.13	0.12	0.0001	0.0021	1.67	sb_gmnbgi_0003c04.pDNRF2	Calumenin b; n=3; Otophysi|Rep: Calumenin b - Danio rerio (Zebrafish) (Brachydanio rerio)
44015	3.98	0.13	0.0001	0.0023	1.67	all_v2.0.11085.C1	Zgc:86905; n=1; Danio rerio|Rep: Zgc:86905 - Danio rerio (Zebrafish) (Brachydanio rerio)
49858	3.79	0.14	0.0002	0.0028	1.67	all_v2.0.2226.C1	unclassified
43195	5.59	0.07	0.0000	0.0013	1.67	all_v2.0.3448.C1	VHSV-induced protein; n=1; Oncorhynchus mykiss|Rep: VHSV-induced protein - Oncorhynchus mykiss (Rainbow trout) (Salmo gairdneri)
51110	2.83	0.19	0.0014	0.0117	1.67	all_v2.0.11720.C1	unclassified
39661	2.80	0.19	0.0015	0.0123	1.66	all_v2.0.4098.C2	Novel protein; n=3; Danio rerio|Rep: Novel protein - Danio rerio (Zebrafish) (Brachydanio rerio)
40720	4.64	0.10	0.0001	0.0016	1.65	all_v2.0.13762.C1	PREDICTED: similar to Nipsnap1 protein; n=1; Monodelphis domestica|Rep: PREDICTED: similar to Nipsnap1 protein - Monodelphis domestica
43485	4.12	0.12	0.0001	0.0021	1.65	all_v2.0.4514.C4	Zgc:112050; n=2; Danio rerio|Rep: Zgc:112050 - Danio rerio (Zebrafish) (Brachydanio rerio)
42564	3.54	0.15	0.0003	0.0036	1.65	all_v2.0.5070.C1	Thymidine phosphorylase precursor (EC 2.4.2.4) (TdRPase) (TP) (Platelet-derived endothelial cell growth factor) (PD-ECGF) (Gliostatin).; n=1; Takifugu rubripes|Rep: Thymidine phosphorylase precursor (EC 2.4.2.4) (TdRPase) (TP) (Platelet-derived endothelial cell growth factor) (PD-ECGF) (Gliostatin). - Takifugu rubripes
40891	3.51	0.16	0.0003	0.0039	1.65	all_v2.0.15259.C1	PREDICTED: similar to protein tyrosine phosphatase, receptor type, B; n=1; Ornithorhynchus anatinus|Rep: PREDICTED: similar to protein tyrosine phosphatase, receptor type, B - Ornithorhynchus anatinus
36080	4.42	0.11	0.0001	0.0017	1.65	sb_gmnbhkas_0015j06.pDNRF2	ATP-sensitive inward rectifier potassium channel 15 (Potassium channel, inwardly rectifying subfamily J member 15) (Inward rectifier K(+) channel Kir4.2) (Kir1.3).; n=1; Takifugu rubripes|Rep: ATP-sensitive inward rectifier potassium

							channel 15 (Potassium channel, inwardly rectifying subfamily J member 15) (Inward rectifier K(+) channel Kir4.2) (Kir1.3). - Takifugu rubripes
41403	3.01	0.20	0.0009	0.0085	1.65	sb_gmnlsfic_0011p03.t7	Proteasome (Prosome, macropain) subunit, alpha type, 6b; n=2; Danio rerio|Rep: Proteasome (Prosome, macropain) subunit, alpha type, 6b - Brachydanio rerio (Zebrafish) (Danio rerio)
39108	2.61	0.25	0.0025	0.0174	1.64	all_v2.0.3508.C1	Mitochondrial import inner membrane translocase subunit Tim13.; n=1; Takifugu rubripes|Rep: Mitochondrial import inner membrane translocase subunit Tim13. - Takifugu rubripes
37498	3.49	0.14	0.0003	0.0039	1.64	all_v2.0.3198.C3	GTP-binding nuclear protein Ran; n=35; Euteleostomi|Rep: GTP-binding nuclear protein Ran - Homo sapiens (Human)
55271	3.50	0.14	0.0003	0.0039	1.64	all_v2.0.2992.C2	unclassified
55394	2.60	0.20	0.0026	0.0176	1.64	all_v2.0.11844.C4	unclassified
47794	2.51	0.23	0.0033	0.0207	1.64	all_v2.0.14226.C1	Unassigned protein
39358	3.24	0.16	0.0005	0.0058	1.64	all_v2.0.362.C2	NOP56; n=5; Danio rerio|Rep: NOP56 - Danio rerio (Zebrafish) (Brachydanio rerio)
54838	2.70	0.20	0.0020	0.0150	1.64	all_v2.0.17312.C1	unclassified
44550	3.66	0.14	0.0002	0.0031	1.63	IRAK41	custom - interleukin-1 receptor-associated kinase 4
36549	2.41	0.24	0.0043	0.0248	1.63	all_v2.0.6292.C1	Co-chaperone protein HscB, mitochondrial precursor (Hsc20) (DnaJ homolog subfamily C member 20).; n=1; Takifugu rubripes|Rep: Co-chaperone protein HscB, mitochondrial precursor (Hsc20) (DnaJ homolog subfamily C member 20). - Takifugu rubripes
39323	4.48	0.10	0.0001	0.0017	1.63	all_v2.0.6027.C9	NFX1-type zinc finger-containing protein 1.; n=1; Takifugu rubripes|Rep: NFX1-type zinc finger-containing protein 1 - Takifugu rubripes
41407	3.80	0.13	0.0002	0.0027	1.63	all_v2.0.9147.C1	Proteasome activator complex subunit 1 (Proteasome activator 28-alpha subunit) (PA28alpha) (PA28a) (Activator of multicatalytic protease subunit 1) (11S regulator complex subunit alpha) (REG-alpha) (Interferon gamma up-regulated I-5111 protein) (IGUP I-51; n=1; Takifugu rubripes|Rep: Proteasome activator complex subunit 1 (Proteasome activator 28-alpha subunit) (PA28alpha) (PA28a) (Activator of multicatalytic protease subunit 1) (11S regulator complex subunit alpha) (REG-alpha) (Interferon gamma up-regulated I-5111 protein) (IGUP I-51 - Takifugu rubripes
43888	3.40	0.14	0.0004	0.0045	1.62	all_v2.0.15537.C1	Zgc:64031; n=3; Danio rerio|Rep: Zgc:64031 - Danio rerio (Zebrafish) (Brachydanio rerio)
40973	2.70	0.23	0.0020	0.0149	1.62	all_v2.0.10058.C1	PREDICTED: similar to Rp42 homolog (pending); n=1; Ornithorhynchus anatinus|Rep: PREDICTED: similar to Rp42 homolog (pending) - Ornithorhynchus anatinus
46931	2.68	0.20	0.0021	0.0156	1.62	sb_gmnlem_0007n11.sp6	Unassigned protein
48343	3.84	0.12	0.0002	0.0026	1.62	sb_gmlbgits_0003d14.sp6	Unassigned protein
51629	4.22	0.10	0.0001	0.0019	1.62	all_v2.0.11166.C1	unclassified

38444	3.96	0.12	0.0001	0.0023	1.62	all_v2.0.10457.C1	Homolog of Homo sapiens Zinc finger CCCH type domain containing protein 1; n=2; Takifugu rubripes|Rep: Homolog of Homo sapiens Zinc finger CCCH type domain containing protein 1 - Takifugu rubripes
45631	4.66	0.09	0.0001	0.0015	1.61	all_v2.0.16338.C1	non-directed
42617	4.92	0.09	0.0000	0.0014	1.61	sb_gmnbpcic_0011f05.pDNRF2	Torsin B precursor (Torsin family 1 member B).; n=1; Takifugu rubripes|Rep: Torsin B precursor (Torsin family 1 member B). - Takifugu rubripes
54782	4.46	0.10	0.0001	0.0017	1.61	all_v2.0.13581.C1	unclassified
37226	2.96	0.17	0.0010	0.0093	1.61	all_v2.0.556.C2	Fibroblast growth factor (Acidic) intracellular binding protein; n=2; Danio rerio|Rep: Fibroblast growth factor (Acidic) intracellular binding protein - Danio rerio (Zebrafish) (Brachydanio rerio)
53017	3.09	0.16	0.0007	0.0074	1.61	all_v2.0.3331.C1	unclassified
39544	3.42	0.14	0.0003	0.0043	1.61	all_v2.0.4218.C1	Novel protein similar to vertebrate transmembrane 4 L six family member 4; n=2; Danio rerio|Rep: Novel protein similar to vertebrate transmembrane 4 L six family member 4 - Danio rerio (Zebrafish) (Brachydanio rerio)
45443	2.96	0.17	0.0010	0.0093	1.61	all_v2.0.7949.C2	MORC3, LOC610219; MORC family CW-type zinc finger 3
51581	4.65	0.09	0.0001	0.0015	1.61	all_v2.0.15506.C1	unclassified
37542	2.85	0.20	0.0013	0.0114	1.61	all_v2.0.8751.C1	Heat shock 10kD protein 1; n=3; Danio rerio|Rep: Heat shock 10kD protein 1 - Danio rerio (Zebrafish) (Brachydanio rerio)
53647	3.71	0.13	0.0002	0.0030	1.60	all_v2.0.18089.C1	unclassified
46560	2.88	0.18	0.0013	0.0108	1.60	sb_gmapte_0011c07.pDNRm13r	Unassigned protein
37520	3.03	0.18	0.0008	0.0082	1.60	all_v2.0.2566.C1	Guanine nucleotide-binding protein-like 3 (Nucleolar GTP-binding protein 3) (Nucleostemin) (E2-induced gene 3-protein) (Novel nucleolar protein 47) (NNP47).; n=1; Takifugu rubripes|Rep: Guanine nucleotide-binding protein-like 3 (Nucleolar GTP-binding protein 3) (Nucleostemin) (E2-induced gene 3-protein) (Novel nucleolar protein 47) (NNP47). - Takifugu rubripes
53739	3.29	0.15	0.0005	0.0053	1.60	all_v2.0.354.C2	unclassified
42664	5.48	0.07	0.0000	0.0013	1.60	sb_gmnlskic_0011d03.pDNRm13r	Transcription factor; n=8; Clupeocephala|Rep: Transcription factor - Danio rerio (Zebrafish) (Brachydanio rerio)
37499	3.26	0.16	0.0005	0.0056	1.60	all_v2.0.3198.C2	GTP-binding nuclear protein Ran; n=35; Euteleostomi|Rep: GTP-binding nuclear protein Ran - Homo sapiens (Human)
54381	4.66	0.09	0.0001	0.0015	1.60	all_v2.0.4316.C1	unclassified
38832	2.99	0.17	0.0009	0.0088	1.60	all_v2.0.17701.C1	LOC558134 protein; n=2; Danio rerio|Rep: LOC558134 protein - Danio rerio (Zebrafish) (Brachydanio rerio)
54777	2.85	0.20	0.0013	0.0113	1.60	all_v2.0.3549.C1	unclassified
52780	2.68	0.20	0.0021	0.0156	1.59	all_v2.0.4435.C1	unclassified
47761	4.91	0.08	0.0001	0.0014	1.59	all_v2.0.7777.C1	Unassigned protein

43876	3.79	0.12	0.0002	0.0028	1.59	all_v2.0.1930.C13	Zgc:63602; n=2; Danio rerio|Rep: Zgc:63602 - Danio rerio (Zebrafish) (Brachydanio rerio)
49133	3.24	0.15	0.0005	0.0057	1.59	all_v2.0.10685.C1	unclassified
45298	5.29	0.07	0.0000	0.0014	1.59	sb_gmapht_0036o07.pDNRF2	LOC728894; similar to gamma-aminobutyric acid (GABA-A) receptor, subunit epsilon
36328	5.10	0.07	0.0000	0.0014	1.59	all_v2.0.16926.C1	CASP8 and FADD-like apoptosis regulator precursor (Cellular FLICE-like inhibitory protein) (c-FLIP) (Caspase-eight-related protein) (Casper) (Caspase-like apoptosis regulatory protein) (CLARP) (MACH-related inducer of toxicity) (MRIT) (Caspase homolog) (C; n=1; Takifugu rubripes|Rep: CASP8 and FADD-like apoptosis regulator precursor (Cellular FLICE-like inhibitory protein) (c-FLIP) (Caspase-eight-related protein) (Casper) (Caspase-like apoptosis regulatory protein) (CLARP) (MACH-related inducer of toxicity) (MRIT) (Caspase homolog) (C - Takifugu rubripes
43137	3.31	0.14	0.0004	0.0051	1.58	all_v2.0.10071.C1	Uroporphyrinogen-III synthase (EC 4.2.1.75) (UROS) (Uroporphyrinogen- III cosynthetase) (Hydroxymethylbilane hydrolyase [cyclizing]) (UROIIIS).; n=1; Takifugu rubripes|Rep: Uroporphyrinogen-III synthase (EC 4.2.1.75) (UROS) (Uroporphyrinogen- III cosynthetase) (Hydroxymethylbilane hydrolyase [cyclizing]) (UROIIIS). - Takifugu rubripes
39484	4.17	0.10	0.0001	0.0020	1.58	all_v2.0.9992.C1	Novel protein similar to vertebrate Mov10, Moloney leukemia virus 10, homolog; n=1; Danio rerio|Rep: Novel protein similar to vertebrate Mov10, Moloney leukemia virus 10, homolog - Danio rerio (Zebrafish) (Brachydanio rerio)
39291	2.63	0.18	0.0024	0.0169	1.58	sb_gmnbmu_0014b24.pDNRF2	Nebulin.; n=1; Takifugu rubripes|Rep: Nebulin. - Takifugu rubripes
45194	3.97	0.11	0.0001	0.0023	1.58	sb_gmnbhkas_0003p09.pDNRF2	LOC570463; similar to Ubiquitin carboxyl-terminal hydrolase 47 (Ubiquitin thioesterase 47) (Ubiquitin-specific-processing protease 47) (Deubiquitinating enzyme 47)
43629	3.64	0.13	0.0002	0.0033	1.58	all_v2.0.9085.C1	Zgc:136962; n=1; Danio rerio|Rep: Zgc:136962 - Danio rerio (Zebrafish) (Brachydanio rerio)
51898	2.56	0.23	0.0029	0.0191	1.58	all_v2.0.5263.C3	unclassified
44256	3.00	0.16	0.0009	0.0086	1.58	all_v2.0.1221.C3	AIG1 domain containing protein
43087	2.95	0.17	0.0010	0.0094	1.58	sb_gmnbbrts_0007a15.pDNRF2	UPI0000ECD032 related cluster; n=1; Gallus gallus|Rep: UPI0000ECD032 UniRef100 entry - Gallus gallus
47039	2.48	0.24	0.0036	0.0220	1.58	sb_gmnbhkas_0001d21.pDNRF2	Unassigned protein
42205	2.43	0.21	0.0041	0.0239	1.58	all_v2.0.3661.C1	Similar to CG3625-PB; n=1; Bos taurus|Rep: Similar to CG3625-PB - Bos taurus (Bovine)
53199	3.49	0.13	0.0003	0.0039	1.57	all_v2.0.4852.C1	unclassified
36398	3.13	0.15	0.0007	0.0070	1.57	sb_gmnlsfas_0003j23.t7	CD3 zeta chain; n=1; Ictalurus punctatus|Rep: CD3 zeta chain - Ictalurus punctatus (Channel catfish)
35770	2.46	0.21	0.0037	0.0225	1.57	all_v2.0.2116.C1	Acyl-CoA synthetase family member 2, mitochondrial precursor; n=2; Danio rerio|Rep: Acyl-CoA synthetase family

							member 2, mitochondrial precursor - Danio rerio (Zebrafish) (Brachydanio rerio)
49199	3.02	0.16	0.0009	0.0084	1.57	all_v2.0.3196.C1	unclassified
36962	2.50	0.19	0.0034	0.0213	1.57	all_v2.0.12651.C1	Dysbindin; n=4; Danio rerio|Rep: Dysbindin - Danio rerio (Zebrafish) (Brachydanio rerio)
46358	2.51	0.20	0.0033	0.0207	1.57	all_v2.0.7414.C2	TNIP3, LOC483847; TNFAIP3 interacting protein 3
46192	4.10	0.10	0.0001	0.0021	1.56	all_v2.0.8585.C1	RVT_1 domain containing protein
54046	4.18	0.10	0.0001	0.0020	1.56	all_v2.0.5705.C1	unclassified
55108	4.30	0.09	0.0001	0.0018	1.56	all_v2.0.8631.C1	unclassified
39936	3.07	0.16	0.0008	0.0077	1.56	all_v2.0.12391.C1	Phosphatidylethanolamine binding protein; n=3; Danio rerio|Rep: Phosphatidylethanolamine binding protein - Danio rerio (Zebrafish) (Brachydanio rerio)
41648	3.08	0.16	0.0007	0.0075	1.56	all_v2.0.15324.C1	PWP2 periodic tryptophan protein homolog; n=2; Danio rerio|Rep: PWP2 periodic tryptophan protein homolog - Danio rerio (Zebrafish) (Brachydanio rerio)
42784	3.40	0.14	0.0004	0.0045	1.56	sb_gmnblits_0022m22.pDNRF2	Transposase; n=27; Anopheles gambiae|Rep: Transposase - Anopheles gambiae (African malaria mosquito)
53096	2.38	0.20	0.0046	0.0263	1.56	all_v2.0.6003.C1	unclassified
53347	3.04	0.15	0.0008	0.0081	1.56	all_v2.0.9197.C1	unclassified
41894	3.80	0.11	0.0002	0.0027	1.55	all_v2.0.2051.C1	Ribosomal protein L32; n=5; Euteleostei|Rep: Ribosomal protein L32 - Oncorhynchus masou formosanus
55097	2.97	0.16	0.0010	0.0091	1.55	all_v2.0.17103.C1	unclassified
50117	2.77	0.17	0.0016	0.0130	1.55	all_v2.0.6258.C1	unclassified
50043	2.62	0.19	0.0025	0.0172	1.55	all_v2.0.12521.C1	unclassified
55349	3.44	0.13	0.0003	0.0042	1.55	all_v2.0.8627.C1	unclassified
45737	3.03	0.14	0.0008	0.0083	1.55	all_v2.0.15887.C1.a	non-directed
45232	4.05	0.10	0.0001	0.0022	1.55	sb_gmapov_0010i17.pDNRm13r	LOC607320; similar to NOL1/NOP2/Sun domain family, member 3
50350	2.74	0.16	0.0018	0.0139	1.55	all_v2.0.6687.C1	unclassified
38619	4.70	0.08	0.0001	0.0015	1.55	all_v2.0.3448.C5	Interferon-inducible protein Gig1; n=1; Psetta maxima|Rep: Interferon-inducible protein Gig1 - Scophthalmus maximus (Turbot)
49472	3.42	0.13	0.0003	0.0043	1.55	all_v2.0.12909.C1	unclassified
42931	3.79	0.11	0.0002	0.0028	1.54	sb_gmnbbr_0015n03.pDNRm13r	Ubiquitin-conjugating enzyme E2 L3; n=32; Euteleostomi|Rep: Ubiquitin-conjugating enzyme E2 L3 - Homo sapiens (Human)
49810	3.27	0.14	0.0005	0.0054	1.54	all_v2.0.15814.C1	unclassified
42202	3.74	0.11	0.0002	0.0029	1.54	all_v2.0.9256.C1	Signal recognition particle receptor subunit alpha (SR-alpha) (Docking protein alpha) (DP-alpha).; n=4; Xenopus tropicalis|Rep: Signal recognition particle receptor subunit alpha (SR-alpha) (Docking protein alpha) (DP-alpha). - Xenopus tropicalis

51254	2.52	0.18	0.0032	0.0205	1.54	all_v2.0.9392.C1	unclassified
44615	2.46	0.19	0.0038	0.0227	1.54	TLR9Cend	custom - toll-like receptor 9
51271	5.11	0.06	0.0000	0.0014	1.54	all_v2.0.15970.C1	unclassified
52097	3.32	0.13	0.0004	0.0050	1.54	all_v2.0.10732.C1	unclassified
43435	2.80	0.16	0.0015	0.0123	1.54	all_v2.0.5852.C1	Zgc:110354; n=3; Danio rerio|Rep: Zgc:110354 - Danio rerio (Zebrafish) (Brachydanio rerio)
40005	2.47	0.20	0.0037	0.0224	1.54	all_v2.0.17201.C1	Plasminogen precursor; n=1; Oncorhynchus mykiss|Rep: Plasminogen precursor - Oncorhynchus mykiss (Rainbow trout) (Salmo gairdneri)
51450	2.47	0.20	0.0037	0.0222	1.54	all_v2.0.12876.C1	unclassified
42836	2.81	0.16	0.0015	0.0122	1.54	sb_gmnbhkas_0004f17.pDNRF2	Tumor necrosis factor receptor-2; n=1; Paralichthys olivaceus|Rep: Tumor necrosis factor receptor-2 - Paralichthys olivaceus (Japanese flounder)
39322	2.88	0.15	0.0012	0.0106	1.53	all_v2.0.6027.C1	NFX1-type zinc finger-containing protein 1.; n=1; Takifugu rubripes|Rep: NFX1-type zinc finger-containing protein 1 - Takifugu rubripes
36265	2.59	0.17	0.0027	0.0179	1.53	all_v2.0.234.C13	Calmodulin; n=24; Eukaryota|Rep: Calmodulin - Homo sapiens (Human)
50815	4.26	0.08	0.0001	0.0019	1.53	all_v2.0.10148.C1	unclassified
49072	3.36	0.13	0.0004	0.0047	1.53	all_v2.0.16355.C1	unclassified
45026	2.86	0.17	0.0013	0.0112	1.53	sb_gmnlsras_0003k15.t7	LOC100030284; similar to asteroid homolog 1
45488	3.12	0.14	0.0007	0.0070	1.53	all_v2.0.11021.C1.a	non-directed
42266	2.66	0.17	0.0022	0.0161	1.53	all_v2.0.15186.C1	sno, strawberry notch homolog 1; n=1; Takifugu rubripes|Rep: sno, strawberry notch homolog 1 - Takifugu rubripes
37834	2.66	0.17	0.0022	0.0161	1.53	all_v2.0.2356.C4	Homolog of Brachydanio rerio Tubulin, alpha 8 like 2.; n=1; Takifugu rubripes|Rep: Homolog of Brachydanio rerio Tubulin, alpha 8 like 2 - Takifugu rubripes
44547	3.56	0.11	0.0003	0.0036	1.53	IRF1OK5	custom - Interferon regulatory factor 10K
35877	3.86	0.10	0.0002	0.0026	1.52	all_v2.0.2896.C1	Alpha-2-macroglobulin; n=1; Sparus aurata|Rep: Alpha-2-macroglobulin - Sparus aurata (Gilthead sea bream)
35880	3.41	0.12	0.0004	0.0044	1.52	sb_gmnllfta_0005j01.t7	Alpha-aminoadipic semialdehyde dehydrogenase; n=12; Eutheria|Rep: Alpha-aminoadipic semialdehyde dehydrogenase - Homo sapiens (Human)
46901	2.80	0.16	0.0015	0.0124	1.52	all_v2.0.143.C1	Unassigned protein
42375	3.63	0.11	0.0002	0.0033	1.52	sb_gmnbspic_0005a23.pDNRF2	SRY-box containing gene 11a; n=1; Gnathonemus petersii|Rep: SRY-box containing gene 11a - Gnathonemus petersii
37655	3.47	0.12	0.0003	0.0041	1.52	sb_gmapov_0006b08.pDNRm13r	Homeobox protein Hox-C13a; n=9; Clupeocephala|Rep: Homeobox protein Hox-C13a - Brachydanio rerio (Zebrafish) (Danio rerio)
45030	3.40	0.12	0.0004	0.0045	1.52	all_v2.0.2894.C4	LOC100031012; similar to very large inducible GTPase 1
40065	2.50	0.18	0.0034	0.0212	1.51	all_v2.0.7210.C1	Poly(RC) binding protein 2; n=3; Euteleostomi|Rep: Poly(RC) binding protein 2 - Danio rerio (Zebrafish) (Brachydanio rerio)
44649	4.83	0.07	0.0001	0.0015	1.51	all_v2.0.661.C6	DKEY-218F9.5, LOC571141; novel protein similar to bloodthirsty (bty)
44663	4.12	0.09	0.0001	0.0021	1.51	all_v2.0.7931.C1	Drf_FH1 multi-domain protein

47223	3.34	0.11	0.0004	0.0049	1.51	sb_gmnlem_0013g13.t7	Unassigned protein
48679	3.11	0.13	0.0007	0.0072	1.51	sb_gmnblits_0037e21.pDNRm13r	Unassigned protein
54855	3.33	0.12	0.0004	0.0049	1.50	all_v2.0.8904.C1	unclassified
41930	2.38	0.20	0.0046	0.0263	1.50	sb_gmnlkfta_0007d04.t7	RING finger protein 145; n=2; Danio rerio|Rep: RING finger protein 145 - Danio rerio (Zebrafish) (Brachydanio rerio)
43270	3.67	0.11	0.0002	0.0031	1.50	all_v2.0.41.C3	Yghl1; n=1; Pagrus major|Rep: Yghl1 - Pagrus major (Red sea bream) (Chrysophrys major)
37633	3.01	0.14	0.0009	0.0084	1.50	all_v2.0.14171.C1	Histidine-rich glycoprotein, putative; n=1; Brugia malayi|Rep: Histidine-rich glycoprotein, putative - Brugia malayi (Filarial nematode worm)
44776	2.91	0.14	0.0011	0.0102	1.50	all_v2.0.18388.C1	EMB2597; EMB2597 (EMBRYO DEFECTIVE 2597)
36380	2.83	0.15	0.0014	0.0118	1.50	all_v2.0.132.C8	CC chemokine type 2; n=1; Gadus morhua|Rep: CC chemokine type 2 - Gadus morhua (Atlantic cod)
47898	4.18	0.08	0.0001	0.0020	1.49	sb_gmnlla_0031c09.t7	Unassigned protein
50192	2.66	0.15	0.0022	0.0160	1.49	all_v2.0.6730.C1	unclassified
41076	3.44	0.11	0.0003	0.0042	1.49	sb_gmnbspic_0005l16.pDNRF2	PREDICTED: similar to TPRD; n=1; Danio rerio|Rep: PREDICTED: similar to TPRD - Danio rerio
49785	2.57	0.17	0.0028	0.0186	1.49	all_v2.0.10614.C1	unclassified
39825	4.59	0.07	0.0001	0.0016	1.49	sb_gmnlsras_0002k15.t7	Out at first protein homolog precursor; n=1; Gallus gallus|Rep: Out at first protein homolog precursor - Gallus gallus (Chicken)
51763	2.87	0.14	0.0013	0.0110	1.49	all_v2.0.2660.C1	unclassified
36306	3.07	0.13	0.0008	0.0077	1.49	all_v2.0.2410.C1	Carboxy-terminal domain RNA polymerase II polypeptide A small phosphatase 1 (EC 3.1.3.16) (Nuclear LIM interactor-interacting factor 3) (NLI-interacting factor 3) (NLI-IF).; n=1; Xenopus tropicalis|Rep: Carboxy-terminal domain RNA polymerase II polypeptide A small phosphatase 1 (EC 3.1.3.16) (Nuclear LIM interactor-interacting factor 3) (NLI-interacting factor 3) (NLI-IF). - Xenopus tropicalis
47982	3.10	0.12	0.0007	0.0073	1.49	sb_gmapht_0015h06.pDNRF2	Unassigned protein
38888	3.66	0.10	0.0002	0.0031	1.49	sb_gmnbbr_0013p04.pDNRF2	Lysyl-tRNA synthetase; n=3; Danio rerio|Rep: Lysyl-tRNA synthetase - Brachydanio rerio (Zebrafish) (Danio rerio)
40887	2.73	0.15	0.0019	0.0143	1.48	all_v2.0.4163.C1	PREDICTED: similar to Protein transport protein SEC61 gamma subunit; n=1; Rattus norvegicus|Rep: PREDICTED: similar to Protein transport protein SEC61 gamma subunit - Rattus norvegicus
46093	2.61	0.15	0.0025	0.0175	1.48	all_v2.0.4918.C1	PTH2 domain containing protein
49606	2.75	0.14	0.0017	0.0136	1.48	all_v2.0.13465.C1	unclassified
37178	2.53	0.18	0.0032	0.0202	1.48	all_v2.0.2512.C1	Fatty acid-binding protein, brain (B-FABP) (Brain lipid-binding protein) (BLBP) (Mammary-derived growth inhibitor related).; n=5; Takifugu rubripes|Rep: Fatty acid-binding protein, brain (B-FABP) (Brain lipid-binding

							protein) (BLBP) (Mammary-derived growth inhibitor related). - Takifugu rubripes
38858	2.44	0.17	0.0039	0.0233	1.48	all_v2.0.3139.C1	Long neurotoxin homolog precursor; n=3; Naja|Rep: Long neurotoxin homolog precursor - Naja atra (Chinese cobra)
41434	4.12	0.08	0.0001	0.0021	1.48	all_v2.0.11758.C1	Proteasome subunit beta type-9 precursor; n=7; Salmonidae|Rep: Proteasome subunit beta type-9 precursor - Oncorhynchus mykiss (Rainbow trout) (Salmo gairdneri)
44858	3.55	0.10	0.0003	0.0036	1.48	all_v2.0.5325.C1	HECT domain containing protein
54357	4.19	0.08	0.0001	0.0020	1.48	all_v2.0.1988.C1	unclassified
37153	3.34	0.11	0.0004	0.0049	1.48	all_v2.0.7254.C1	Family with sequence similarity 50, member A; n=3; Otophysi|Rep: Family with sequence similarity 50, member A - Danio rerio (Zebrafish) (Brachydanio rerio)
48455	2.84	0.14	0.0014	0.0116	1.48	all_v2.0.10330.C1	Unassigned protein
51976	2.58	0.15	0.0027	0.0182	1.48	all_v2.0.5530.C4	unclassified
36789	3.48	0.10	0.0003	0.0040	1.48	sb_gmnlkfic_0001a07.t7	Cytosolic nonspecific dipeptidase; n=3; Percomorpha|Rep: Cytosolic nonspecific dipeptidase - Oreochromis niloticus (Nile tilapia) (Tilapia nilotica)
38459	2.48	0.19	0.0036	0.0220	1.47	all_v2.0.17725.C1	Homolog of Ictalurus punctatus Caspase 8.; n=1; Takifugu rubripes|Rep: Homolog of Ictalurus punctatus Caspase 8 - Takifugu rubripes
52759	2.81	0.15	0.0015	0.0122	1.47	all_v2.0.12814.C1	unclassified
44716	3.01	0.13	0.0009	0.0085	1.47	all_v2.0.10928.C1	DUF1777 multi-domain protein
50983	3.49	0.10	0.0003	0.0039	1.47	all_v2.0.12201.C1	unclassified
49048	4.53	0.06	0.0001	0.0017	1.47	all_v2.0.593.C1	unclassified
51491	3.85	0.09	0.0002	0.0026	1.47	all_v2.0.4690.C1	unclassified
47179	2.42	0.17	0.0042	0.0244	1.47	all_v2.0.14321.C1	Unassigned protein
44794	2.51	0.18	0.0033	0.0207	1.47	sb_gmnbhkas_0005o07.pDNRm13r	FAM79A; family with sequence similarity 79, member A
42840	2.80	0.14	0.0015	0.0124	1.46	all_v2.0.5109.C1	Tumor-associated calcium signal transducer 1 precursor (Major gastrointestinal tumor-associated protein GA733-2) (Epithelial cell surface antigen) (Epithelial glycoprotein) (EGP) (Adenocarcinoma- associated antigen) (KSA) (KS 1/4/2008 antigen) (Cell surface gl; n=1; Takifugu rubripes|Rep: Tumor-associated calcium signal transducer 1 precursor (Major gastrointestinal tumor-associated protein GA733-2) (Epithelial cell surface antigen) (Epithelial glycoprotein) (EGP) (Adenocarcinoma-associated antigen) (KSA) (KS 1/4/2008 antigen) (Cell surface gl - Takifugu rubripes
42501	3.25	0.11	0.0005	0.0056	1.46	all_v2.0.11389.C1	T-complex protein 1 subunit delta; n=4; Tetraodontidae|Rep: T-complex protein 1 subunit delta - Fugu rubripes (Japanese pufferfish) (Takifugu rubripes)
40396	2.60	0.16	0.0026	0.0178	1.46	all_v2.0.134.C2	PREDICTED: similar to deltex 3-like (Drosophila); n=1; Danio rerio|Rep: PREDICTED: similar to deltex 3-like (Drosophila) - Danio rerio

41088	3.02	0.13	0.0009	0.0084	1.46	all_v2.0.3752.C1	PREDICTED: similar to transmembrane protein 7; n=1; Equus caballus|Rep: PREDICTED: similar to transmembrane protein 7 - Equus caballus
46168	2.67	0.14	0.0021	0.0157	1.46	all_v2.0.4749.C2	RNA 3'-terminal phosphate cyclase-like protein.; n=1; Takifugu rubripes
40840	2.81	0.13	0.0015	0.0122	1.46	sb_gmnbhkas_0018n05.pDNRF2	PREDICTED: similar to poly (ADP-ribose) polymerase family, member 11,; n=1; Monodelphis domestica|Rep: PREDICTED: similar to poly (ADP-ribose) polymerase family, member 11, - Monodelphis domestica
38160	3.57	0.09	0.0003	0.0036	1.46	sb_gmnlsfic_0003i11.t7	Homolog of Homo sapiens Membrane interacting protein of RGS16; n=1; Takifugu rubripes|Rep: Homolog of Homo sapiens Membrane interacting protein of RGS16 - Takifugu rubripes
48472	2.92	0.12	0.0011	0.0101	1.45	all_v2.0.18800.C1	Unassigned protein
39469	3.28	0.10	0.0005	0.0054	1.45	all_v2.0.12276.C1	Novel protein similar to vertebrate EF-hand domain family, member D2; n=2; Danio rerio|Rep: Novel protein similar to vertebrate EF-hand domain family, member D2 - Danio rerio (Zebrafish) (Brachydanio rerio)
37749	2.44	0.16	0.0039	0.0233	1.45	sb_gmapov_0007g07.pDNRm13r	Homolog of Brachydanio rerio Nucleolin.; n=1; Takifugu rubripes|Rep: Homolog of Brachydanio rerio Nucleolin. - Takifugu rubripes
40052	2.61	0.16	0.0026	0.0176	1.45	all_v2.0.16639.C1	Poly [ADP-ribose] polymerase 12 (EC 2.4.2.30) (PARP-12) (Zinc finger CCCH domain-containing protein 1).; n=1; Takifugu rubripes|Rep: Poly [ADP-ribose] polymerase 12 (EC 2.4.2.30) (PARP-12) (Zinc finger CCCH domain-containing protein 1). - Takifugu rubripes
41397	3.78	0.08	0.0002	0.0028	1.45	all_v2.0.847.C1	Prostaglandin E synthase 3; n=5; Danio rerio|Rep: Prostaglandin E synthase 3 - Danio rerio (Zebrafish) (Brachydanio rerio)
50525	3.57	0.09	0.0003	0.0036	1.45	all_v2.0.832.C1	unclassified
42301	2.93	0.13	0.0011	0.0100	1.45	all_v2.0.14484.C1	Solute carrier family 25 member 36; n=3; Clupeocephala|Rep: Solute carrier family 25 member 36 - Danio rerio (Zebrafish) (Brachydanio rerio)
50382	3.04	0.12	0.0008	0.0080	1.44	all_v2.0.9227.C1	unclassified
37282	2.59	0.14	0.0027	0.0180	1.44	sb_gmnblits_0007g05.pDNRF2	Fzb-1; n=1; Danio rerio|Rep: Fzb-1 - Brachydanio rerio (Zebrafish) (Danio rerio)
55038	2.38	0.17	0.0047	0.0264	1.44	all_v2.0.3773.C1	unclassified
55532	4.01	0.08	0.0001	0.0022	1.44	all_v2.0.10346.C1	ZNFX1; zinc finger, NFX1-type containing 1
52162	2.52	0.15	0.0032	0.0205	1.44	all_v2.0.11655.C1	unclassified
51980	3.63	0.09	0.0002	0.0033	1.44	all_v2.0.703.C1	unclassified
48995	3.07	0.12	0.0007	0.0076	1.44	sb_gmnlla_0001p14.t7	Unassigned protein
46606	3.19	0.11	0.0006	0.0063	1.44	sb_gmnlem_0011g08.t7	Unassigned protein
50881	2.78	0.13	0.0016	0.0127	1.43	all_v2.0.324.C1	unclassified
38545	2.51	0.16	0.0033	0.0210	1.43	all_v2.0.2319.C2	IgD mRNA for immunoglobulin D; n=1; Takifugu rubripes|Rep: IgD mRNA for immunoglobulin D - Takifugu rubripes
44351	2.38	0.15	0.0047	0.0265	1.43	all_v2.0.693.C1	C2 domain containing protein
49061	3.39	0.10	0.0004	0.0045	1.43	all_v2.0.10961.C1	unclassified

50535	3.31	0.10	0.0004	0.0051	1.43	all_v2.0.8723.C1	unclassified
36923	3.41	0.09	0.0003	0.0044	1.43	sb_gmapov_0012f15.pDNRF2	DnaJ-like subfamily A member 4; n=1; Paralichthys olivaceus|Rep: DnaJ-like subfamily A member 4 - Paralichthys olivaceus (Japanese flounder)
42172	2.92	0.13	0.0011	0.0101	1.43	sb_gmnlla_0045h19.t7	Si:dkey-21k10.1 protein; n=3; Danio rerio|Rep: Si:dkey-21k10.1 protein - Danio rerio (Zebrafish) (Brachydanio rerio)
50297	3.10	0.11	0.0007	0.0073	1.43	all_v2.0.12538.C1	unclassified
50934	3.49	0.09	0.0003	0.0039	1.43	all_v2.0.1022.C3	unclassified
50913	4.77	0.05	0.0001	0.0015	1.43	all_v2.0.3093.C1	unclassified
49047	2.66	0.14	0.0023	0.0162	1.43	all_v2.0.11108.C4	unclassified
39384	2.70	0.14	0.0020	0.0150	1.42	all_v2.0.3955.C1	Novel NACHT domanin containing protein; n=2; Danio rerio|Rep: Novel NACHT domanin containing protein - Danio rerio (Zebrafish) (Brachydanio rerio)
43169	3.17	0.10	0.0006	0.0065	1.42	all_v2.0.927.C1	Vacuolar protein-sorting-associated protein 25; n=6; Clupeocephala|Rep: Vacuolar protein-sorting-associated protein 25 - Danio rerio (Zebrafish) (Brachydanio rerio)
51058	2.64	0.13	0.0023	0.0165	1.42	all_v2.0.11781.C2	unclassified
46677	2.66	0.14	0.0022	0.0161	1.42	all_v2.0.4.C247	Unassigned protein
48710	2.64	0.13	0.0023	0.0165	1.42	all_v2.0.10234.C1	Unassigned protein
43155	3.77	0.08	0.0002	0.0028	1.42	sb_gmnbpcic_0007k03.pDNRF2	Vacuolar protein sorting-associated protein 13B (Cohen syndrome protein 1).; n=1; Takifugu rubripes|Rep: Vacuolar protein sorting-associated protein 13B (Cohen syndrome protein 1). - Takifugu rubripes
39789	2.81	0.12	0.0015	0.0122	1.42	sb_gmnlsras_0001e04.t7	O-GlcNAc transferase variant 4; n=8; Euteleostomi|Rep: O-GlcNAc transferase variant 4 - Danio rerio (Zebrafish) (Brachydanio rerio)
55531	5.08	0.04	0.0000	0.0014	1.42	all_v2.0.13201.C1	ZNF658; zinc finger protein 658; K09228 KRAB domain-containing zinc finger protein
36967	3.00	0.12	0.0009	0.0086	1.42	sb_gmnbhkas_0014o03.pDNRm13r	E3 SUMO-protein ligase RanBP2; n=3; Homo sapiens|Rep: E3 SUMO-protein ligase RanBP2 - Homo sapiens (Human)
35574	3.93	0.07	0.0001	0.0024	1.42	sb_gmnlsric_0003f13.t7	26S proteasome regulatory subunit IV; n=2; Embryophyta|Rep: 26S proteasome regulatory subunit IV - Tortula ruralis (Star moss) (Twisted moss)
48214	3.18	0.10	0.0006	0.0063	1.42	sb_gmnbhkas_0014f23.pDNRm13r	Unassigned protein
37738	3.46	0.09	0.0003	0.0041	1.41	sb_gmnbhkic_0007m23.pDNRF2	Homolog of Brachydanio rerio Microspherule protein 1.; n=1; Takifugu rubripes|Rep: Homolog of Brachydanio rerio Microspherule protein 1 - Takifugu rubripes
54293	3.80	0.08	0.0002	0.0027	1.41	all_v2.0.5441.C1	unclassified
49108	2.50	0.15	0.0034	0.0213	1.41	all_v2.0.210.C1	unclassified
53802	2.70	0.13	0.0020	0.0149	1.41	all_v2.0.6636.C2	unclassified
42254	2.77	0.13	0.0016	0.0130	1.41	all_v2.0.1577.C1	Small ubiquitin-related modifier 3 precursor; n=3; Catarrhini|Rep: Small ubiquitin-related modifier 3 precursor - Homo sapiens (Human)

52306	4.19	0.06	0.0001	0.0020	1.41	all_v2.0.14732.C1	unclassified
39852	2.74	0.13	0.0018	0.0138	1.41	all_v2.0.1325.C1	Pantophysin; n=3; Gadidae|Rep: Pantophysin - Melanogrammus aeglefinus (Haddock)
54648	3.21	0.09	0.0005	0.0060	1.41	all_v2.0.12243.C1	unclassified
50854	2.88	0.11	0.0012	0.0107	1.41	all_v2.0.7384.C1	unclassified
38108	2.63	0.13	0.0024	0.0170	1.41	sb_gmnlbric_0007j22.t7	Homolog of Homo sapiens Inhibitor of nuclear factor kappa-B kinase alpha subunit; n=1; Takifugu rubripes|Rep: Homolog of Homo sapiens Inhibitor of nuclear factor kappa-B kinase alpha subunit - Takifugu rubripes
38165	2.65	0.12	0.0023	0.0163	1.40	sb_gmapov_0007h19.pDNRm13r	Homolog of Homo sapiens MGC4645 protein; n=1; Takifugu rubripes|Rep: Homolog of Homo sapiens MGC4645 protein - Takifugu rubripes
51890	3.26	0.09	0.0005	0.0055	1.40	all_v2.0.13745.C1	unclassified
38483	2.69	0.12	0.0021	0.0152	1.40	all_v2.0.3163.C5	Homolog of Pagrus major Protein-glutamine gamma-glutamyltransferase (EC 2.3.2.13) (Tissue transglutaminase) (TGase C) (TGC) (TG(C)) (Tranglutaminase 2).; n=1; Takifugu rubripes|Rep: Homolog of Pagrus major Protein-glutamine gamma-glutamyltransferase (EC 2.3.2.13) (Tissue transglutaminase) (TGase C) (TGC) (TG(C)) (Tranglutaminase 2). - Takifugu rubripes
40166	2.55	0.14	0.0030	0.0192	1.40	sb_gmnlbric_0007l18.t7	PREDICTED: similar to 24-dehydrocholesterol reductase; n=1; Danio rerio|Rep: PREDICTED: similar to 24-dehydrocholesterol reductase - Danio rerio
54819	2.79	0.12	0.0016	0.0125	1.40	all_v2.0.14839.C1	unclassified
51059	3.00	0.10	0.0009	0.0086	1.40	all_v2.0.690.C5	unclassified
36400	2.39	0.14	0.0045	0.0257	1.40	all_v2.0.7561.C1	CD40; n=1; Paralichthys olivaceus|Rep: CD40 - Paralichthys olivaceus (Japanese flounder)
37641	2.67	0.12	0.0022	0.0159	1.40	all_v2.0.285.C1	Histone deacetylase complex subunit SAP18; n=19; Euteleostomi|Rep: Histone deacetylase complex subunit SAP18 - Homo sapiens (Human)
40559	2.51	0.13	0.0033	0.0208	1.40	sb_gmapht_0017j13.pDNRF2	PREDICTED: similar to Importin alpha-1 subunit (Karyopherin alpha-1 subunit) (SRP1-beta) (RAG cohort protein 2) (Nucleoprotein interactor 1) (NPI-1), partial; n=1; Danio rerio|Rep: PREDICTED: similar to Importin alpha-1 subunit (Karyopherin alpha-1 subunit) (SRP1-beta) (RAG cohort protein 2) (Nucleoprotein interactor 1) (NPI-1), partial - Danio rerio
48510	4.45	0.05	0.0001	0.0017	1.40	sb_gmnlla_0035h19.t7	Unassigned protein
48825	3.06	0.10	0.0008	0.0078	1.40	sb_gmnlla_0004a24.t7	Unassigned protein
55410	2.64	0.13	0.0023	0.0165	1.40	all_v2.0.12831.C1	unclassified
38414	2.93	0.11	0.0011	0.0099	1.40	sb_gmnlem_0008p21.sp6	Homolog of Homo sapiens TRA@ protein; n=8; Takifugu rubripes|Rep: Homolog of Homo sapiens TRA@ protein - Takifugu rubripes
38631	3.71	0.07	0.0002	0.0030	1.40	all_v2.0.8520.C1	Interleukin-11a; n=2; Takifugu rubripes|Rep: Interleukin-11a - Fugu rubripes (Japanese pufferfish) (Takifugu rubripes)

55249	2.47	0.13	0.0036	0.0221	1.39	all_v2.0.3039.C5	unclassified
49081	2.87	0.10	0.0013	0.0109	1.39	all_v2.0.4460.C1	unclassified
39952	2.44	0.15	0.0040	0.0235	1.39	all_v2.0.6525.C1	phosphatidylinositol-specific phospholipase C, X domain containing 1; n=1; Takifugu rubripes|Rep: phosphatidylinositol-specific phospholipase C, X domain containing 1 - Takifugu rubripes
37404	2.41	0.15	0.0043	0.0249	1.39	all_v2.0.10850.C1	Glycerol-3-phosphate dehydrogenase 1-like protein (EC 1.1.1.8).; n=1; Takifugu rubripes|Rep: Glycerol-3-phosphate dehydrogenase 1-like protein (EC 1.1.1.8). - Takifugu rubripes
40138	2.60	0.13	0.0026	0.0176	1.39	all_v2.0.14727.C1	PREDICTED: heat shock 70kDa protein 5 (glucose-regulated protein, 78kDa) isoform 1; n=1; Pan troglodytes|Rep: PREDICTED: heat shock 70kDa protein 5 (glucose-regulated protein, 78kDa) isoform 1 - Pan troglodytes
53993	3.22	0.09	0.0005	0.0060	1.39	all_v2.0.9743.C1	unclassified
42879	2.50	0.13	0.0034	0.0212	1.39	all_v2.0.14074.C1	U3 small nucleolar RNA-associated protein 15 homolog; n=1; Gallus gallus|Rep: U3 small nucleolar RNA-associated protein 15 homolog - Gallus gallus (Chicken)
43024	2.62	0.12	0.0024	0.0171	1.38	all_v2.0.17625.C1	UPI0000D8C23B related cluster; n=1; Danio rerio|Rep: UPI0000D8C23B UniRef100 entry - Danio rerio
41548	2.44	0.13	0.0039	0.0233	1.38	all_v2.0.2626.C1	Protein YIPF6; n=4; Danio rerio|Rep: Protein YIPF6 - Danio rerio (Zebrafish) (Brachydanio rerio)
50340	2.70	0.11	0.0020	0.0150	1.38	all_v2.0.3758.C1	unclassified
48320	3.50	0.08	0.0003	0.0039	1.38	all_v2.0.74.C2	Unassigned protein
51423	2.66	0.12	0.0022	0.0161	1.38	all_v2.0.7996.C1	unclassified
44205	3.08	0.09	0.0007	0.0075	1.38	all_v2.0.8912.C1	[J] COG1358 Ribosomal protein HS6-type (S12/L30/L7a)
55014	3.68	0.07	0.0002	0.0031	1.38	all_v2.0.14410.C1	unclassified
46732	3.55	0.07	0.0003	0.0036	1.38	sb_gmnlem_0027j18.t7	Unassigned protein
42340	2.80	0.11	0.0015	0.0124	1.38	all_v2.0.7989.C1	sphingolipid C4-hydroxylase/delta 4-desaturase; n=1; Takifugu rubripes|Rep: sphingolipid C4-hydroxylase/delta 4-desaturase - Takifugu rubripes
49698	2.74	0.12	0.0018	0.0139	1.38	all_v2.0.7758.C1	unclassified
40018	2.84	0.10	0.0014	0.0115	1.38	all_v2.0.7260.C1	Pol polyprotein; n=1; Nosema bombycis|Rep: Pol polyprotein - Nosema bombycis
47688	2.52	0.12	0.0032	0.0203	1.38	sb_gmnlla_0002n10.t7	Unassigned protein
45108	2.84	0.11	0.0014	0.0114	1.38	all_v2.0.12306.C1	LOC485766; similar to serine palmitoyltransferase, long chain base subunit 2
40216	2.38	0.13	0.0046	0.0263	1.37	sb_gmapte_0010k12.pDNRm13r	PREDICTED: similar to ankyrin 2, neuronal,; n=1; Danio rerio|Rep: PREDICTED: similar to ankyrin 2, neuronal, - Danio rerio
41233	2.63	0.12	0.0024	0.0170	1.37	sb_gmapov_0002e13.pDNRF2	PREDICTED: similar to Zinc finger protein 319; n=1; Danio rerio|Rep: PREDICTED: similar to Zinc finger protein 319 - Danio rerio
41512	3.91	0.06	0.0001	0.0024	1.37	sb_gmapov_0012p05.pDNRm13r	Protein pelota homolog; n=1; Danio rerio|Rep: Protein pelota homolog - Brachydanio rerio (Zebrafish) (Danio

							rerio)
41719	2.57	0.12	0.0028	0.0185	1.37	all_v2.0.12252.C1	RecA homolog Dmc1; n=4; Elopocephala|Rep: RecA homolog Dmc1 - Oreochromis niloticus (Nile tilapia) (Tilapia nilotica)
44658	2.55	0.12	0.0030	0.0194	1.37	sb_gmapht_0008n23.pDNRF2	DNA_pol_V domain containing protein
36171	3.04	0.09	0.0008	0.0081	1.37	sb_gmnbhkic_0003b04.pDNRF2	Biglycan-like protein 3; n=1; Oreochromis niloticus|Rep: Biglycan-like protein 3 - Oreochromis niloticus (Nile tilapia) (Tilapia nilotica)
54214	2.48	0.12	0.0036	0.0220	1.37	all_v2.0.7211.C1	unclassified
41431	4.47	0.04	0.0001	0.0017	1.36	all_v2.0.15734.C1	Proteasome subunit beta type; n=5; Salmonidae|Rep: Proteasome subunit beta type - Salmo salar (Atlantic salmon)
45788	2.53	0.12	0.0032	0.0202	1.36	all_v2.0.11742.C1	non-directed
45507	4.00	0.05	0.0001	0.0022	1.36	all_v2.0.13496.C1	non-directed
36428	2.45	0.13	0.0039	0.0231	1.36	all_v2.0.5302.C1	Cell division cycle 5-like protein; n=18; Amniota|Rep: Cell division cycle 5-like protein - Homo sapiens (Human)
37158	3.45	0.07	0.0003	0.0042	1.36	all_v2.0.11686.C1	Fance protein; n=4; Danio rerio|Rep: Fance protein - Danio rerio (Zebrafish) (Brachydanio rerio)
36002	2.82	0.10	0.0014	0.0119	1.36	all_v2.0.13332.C1	Asialoglycoprotein receptor 1 (ASGPR 1) (ASGP-R 1) (Hepatic lectin H1).; n=1; Takifugu rubripes|Rep: Asialoglycoprotein receptor 1 (ASGPR 1) (ASGP-R 1) (Hepatic lectin H1). - Takifugu rubripes
50069	2.63	0.11	0.0024	0.0170	1.36	all_v2.0.17582.C1	unclassified
52009	2.93	0.09	0.0011	0.0099	1.36	all_v2.0.17459.C1	unclassified
36367	3.08	0.08	0.0007	0.0075	1.36	all_v2.0.1055.C2	Cathepsin L; n=2; Percomorpha|Rep: Cathepsin L - Lates calcarifer (Barramundi)
44069	3.72	0.06	0.0002	0.0029	1.36	all_v2.0.2597.C1	Zgc:92287; n=2; Danio rerio|Rep: Zgc:92287 - Danio rerio (Zebrafish) (Brachydanio rerio)
35937	2.54	0.12	0.0031	0.0197	1.36	all_v2.0.5783.C1	Anterior gradient-2-like protein 2; n=2; Salmo salar|Rep: Anterior gradient-2-like protein 2 - Salmo salar (Atlantic salmon)
41308	2.63	0.11	0.0024	0.0170	1.36	sb_gmnllfta_0002a10.t7	Prmt1 protein; n=2; Danio rerio|Rep: Prmt1 protein - Brachydanio rerio (Zebrafish) (Danio rerio)
49766	2.92	0.09	0.0011	0.0101	1.35	all_v2.0.15335.C1	unclassified
47347	2.40	0.12	0.0044	0.0254	1.35	sb_gmlbgits_0004o24.sp6	Unassigned protein
44888	2.52	0.11	0.0032	0.0205	1.35	sb_gmapov_0013f02.pDNRF2	Hkr3; GLI-Kruppel family member HKR3
37118	2.62	0.11	0.0024	0.0171	1.35	all_v2.0.2012.C1	Eukaryotic translation initiation factor 4E; n=5; Xenopus|Rep: Eukaryotic translation initiation factor 4E - Xenopus laevis (African clawed frog)
42695	2.58	0.11	0.0027	0.0182	1.35	all_v2.0.13546.C1	Translocon-associated protein subunit alpha precursor; n=1; Oncorhynchus mykiss|Rep: Translocon-associated protein subunit alpha precursor - Oncorhynchus mykiss (Rainbow trout) (Salmo gairdneri)
50704	2.71	0.10	0.0019	0.0148	1.35	all_v2.0.4181.C1	unclassified
43609	3.01	0.09	0.0009	0.0084	1.35	sb_gmnbmd_0004p22.t7	Zgc:136585; n=3; Danio rerio|Rep: Zgc:136585 - Brachydanio rerio (Zebrafish) (Danio rerio)
43434	2.53	0.12	0.0031	0.0198	1.35	all_v2.0.14566.C1	Zgc:110354; n=3; Danio rerio|Rep: Zgc:110354 - Danio rerio (Zebrafish) (Brachydanio rerio)

48545	2.79	0.10	0.0016	0.0125	1.34	sb_gmlbgits_0004e18.sp6	Unassigned protein
53244	3.85	0.05	0.0002	0.0026	1.34	all_v2.0.1478.C3	unclassified
37566	2.39	0.12	0.0045	0.0257	1.34	all_v2.0.2479.C1	Heat shock protein 90Ae.; n=1; Takifugu rubripes|Rep: Heat shock protein 90Ae. - Takifugu rubripes
51355	2.38	0.12	0.0046	0.0261	1.34	all_v2.0.154.C1	unclassified
44066	2.81	0.09	0.0015	0.0122	1.34	all_v2.0.7765.C1	Zgc:92254; n=2; Danio rerio|Rep: Zgc:92254 - Danio rerio (Zebrafish) (Brachydanio rerio)
38422	2.48	0.12	0.0036	0.0220	1.34	sb_gmapov_0019e16.pDNRF2	Homolog of Homo sapiens Transport-secretion protein 2.2; n=1; Takifugu rubripes|Rep: Homolog of Homo sapiens Transport-secretion protein 2.2 - Takifugu rubripes
46704	2.38	0.13	0.0047	0.0264	1.34	sb_gmnlla_0034c04.t7	Unassigned protein
35985	3.28	0.07	0.0005	0.0054	1.34	all_v2.0.17834.C1	Armadillo repeat protein deleted in velo-cardio-facial syndrome.; n=1; Takifugu rubripes|Rep: Armadillo repeat protein deleted in velo-cardio-facial syndrome. - Takifugu rubripes
54760	3.00	0.09	0.0009	0.0086	1.34	all_v2.0.5450.C1	unclassified
46490	2.72	0.10	0.0019	0.0144	1.34	sb_gmnbmu_0006k01.pDNRF2	Unassigned protein
51413	2.73	0.10	0.0018	0.0142	1.34	all_v2.0.318.C3	unclassified
50515	2.46	0.11	0.0037	0.0225	1.34	all_v2.0.7899.C1	unclassified
53667	2.70	0.09	0.0020	0.0151	1.34	all_v2.0.2943.C1	unclassified
41285	2.93	0.08	0.0011	0.0098	1.34	sb_gmapov_0003j06.pDNRF2	PREDICTED: transcription elongation factor B (SIII), polypeptide 2 (18kD, elongin B); n=1; Danio rerio|Rep: PREDICTED: transcription elongation factor B (SIII), polypeptide 2 (18kD, elongin B) - Danio rerio
49161	2.61	0.10	0.0025	0.0175	1.33	all_v2.0.7045.C1	unclassified
50493	3.74	0.05	0.0002	0.0029	1.33	all_v2.0.7812.C1	unclassified
54330	2.48	0.12	0.0036	0.0221	1.33	all_v2.0.13602.C1	unclassified
41690	3.57	0.06	0.0003	0.0036	1.33	all_v2.0.901.C1	RAS guanyl-releasing protein 1; n=3; Xenopus|Rep: RAS guanyl-releasing protein 1 - Xenopus laevis (African clawed frog)
55069	2.41	0.11	0.0043	0.0250	1.33	all_v2.0.5005.C1	unclassified
41309	3.13	0.08	0.0007	0.0069	1.33	all_v2.0.1996.C1	Prmt1 protein; n=2; Danio rerio|Rep: Prmt1 protein - Danio rerio (Zebrafish) (Brachydanio rerio)
53627	2.57	0.10	0.0028	0.0187	1.33	all_v2.0.20.C2	unclassified
38998	2.43	0.11	0.0041	0.0241	1.33	sb_gmnlem_0024l03.t7	MGC115621 protein; n=1; Xenopus laevis|Rep: MGC115621 protein - Xenopus laevis (African clawed frog)
37568	2.43	0.11	0.0040	0.0238	1.33	all_v2.0.5461.C1	Heat shock protein 94c.; n=1; Takifugu rubripes|Rep: Heat shock protein 94c. - Takifugu rubripes
49538	2.44	0.11	0.0039	0.0233	1.33	all_v2.0.17441.C1	unclassified
46452	2.56	0.11	0.0029	0.0188	1.33	all_v2.0.3031.C1	Unassigned protein
55082	2.68	0.10	0.0021	0.0155	1.33	all_v2.0.1181.C1	unclassified
36192	2.97	0.08	0.0010	0.0090	1.32	all_v2.0.661.C40	Bloodthirsty; n=2; Notothenioidei|Rep: Bloodthirsty - Chaenocephalus aceratus (White crocodile fish)

55489	2.43	0.10	0.0041	0.0239	1.32	sb_gmapht_0042b22.pDNRm13r	zf-BED domain containing protein
40604	2.39	0.11	0.0045	0.0257	1.32	sb_gmapte_0010i04.pDNRm13r	PREDICTED: similar to KPL2; n=1; Ornithorhynchus anatinus|Rep: PREDICTED: similar to KPL2 - Ornithorhynchus anatinus
50319	3.09	0.07	0.0007	0.0074	1.32	all_v2.0.15552.C1	unclassified
52277	3.44	0.06	0.0003	0.0042	1.32	all_v2.0.10829.C1	unclassified
51417	3.55	0.06	0.0003	0.0036	1.32	all_v2.0.3611.C1	unclassified
49834	3.63	0.05	0.0002	0.0033	1.32	all_v2.0.12788.C1	unclassified
48601	2.52	0.10	0.0032	0.0205	1.32	sb_gmnlla_0045m15.t7	Unassigned protein
37096	2.38	0.11	0.0047	0.0266	1.32	all_v2.0.624.C1	Eukaryotic translation initiation factor 2 subunit 1; n=22; Euteleostomi|Rep: Eukaryotic translation initiation factor 2 subunit 1 - Homo sapiens (Human)
41416	2.76	0.08	0.0017	0.0132	1.32	all_v2.0.15427.C1	Proteasome subunit alpha type; n=2; Tetraodontidae|Rep: Proteasome subunit alpha type - Tetraodon nigroviridis (Green puffer)
45703	2.39	0.11	0.0046	0.0260	1.31	all_v2.0.2158.C2	non-directed
36843	2.50	0.10	0.0034	0.0212	1.31	sb_gmnlsfas_0003o17.t7	Der1-like domain family, member 1; n=4; Danio rerio|Rep: Der1-like domain family, member 1 - Danio rerio (Zebrafish) (Brachydanio rerio)
43891	2.70	0.09	0.0020	0.0151	1.31	all_v2.0.6116.C1	Zgc:64051; n=1; Danio rerio|Rep: Zgc:64051 - Danio rerio (Zebrafish) (Brachydanio rerio)
48805	2.47	0.11	0.0037	0.0222	1.31	sb_gmnlem_0007p04.sp6	Unassigned protein
49134	2.60	0.09	0.0026	0.0176	1.31	all_v2.0.3574.C1	unclassified
38854	2.75	0.09	0.0017	0.0136	1.31	all_v2.0.4197.C1	LOC794166 protein; n=2; Danio rerio|Rep: LOC794166 protein - Danio rerio (Zebrafish) (Brachydanio rerio)
36799	2.73	0.09	0.0018	0.0141	1.31	all_v2.0.4522.C1	D-amino-acid oxidase 1; n=5; Danio rerio|Rep: D-amino-acid oxidase 1 - Danio rerio (Zebrafish) (Brachydanio rerio)
44187	2.80	0.08	0.0015	0.0123	1.31	all_v2.0.10214.C1	Zymogen granule membrane protein 16 precursor (Zymogen granule protein 16) (Secretory lectin ZG16).; n=1; Takifugu rubripes|Rep: Zymogen granule membrane protein 16 precursor (Zymogen granule protein 16) (Secretory lectin ZG16). - Takifugu rubripes
49035	2.88	0.08	0.0012	0.0108	1.31	sb_gmnbgi_0003o02.pDNRF2	Unassigned protein
47677	2.78	0.08	0.0016	0.0128	1.31	sb_gmnbhkic_0004l22.pDNRF2	Unassigned protein
36874	2.67	0.09	0.0022	0.0157	1.31	all_v2.0.11034.C1	disulfide isomerase; n=1; Takifugu rubripes|Rep: disulfide isomerase - Takifugu rubripes
39371	2.53	0.10	0.0032	0.0202	1.31	sb_gmnbhkas_0007i12.pDNRF2	Novel immune-type receptor 4; n=2; Oncorhynchus mykiss|Rep: Novel immune-type receptor 4 - Oncorhynchus mykiss (Rainbow trout) (Salmo gairdneri)
52660	2.57	0.09	0.0028	0.0187	1.31	all_v2.0.14023.C1	unclassified
51854	3.28	0.06	0.0005	0.0054	1.30	all_v2.0.15245.C1	unclassified
39338	2.38	0.10	0.0046	0.0263	1.30	sb_gmnbhkic_0008m05.pDNRF2	NILT2 leucocyte receptor; n=2; Cyprinus carpio|Rep: NILT2 leucocyte receptor - Cyprinus carpio (Common carp)

45283	2.56	0.09	0.0029	0.0189	1.30	all_v2.0.6467.C1	LOC712175; similar to Zinc finger protein 74 (hZNF7)
45221	3.22	0.06	0.0005	0.0060	1.30	sb_gmnbspic_0014i03.pDNRm13r	LOC588946; similar to EF-hand calcium binding domain 5
45657	2.61	0.09	0.0025	0.0174	1.30	all_v2.0.1588.C2	non-directed
51403	2.90	0.07	0.0012	0.0103	1.30	all_v2.0.6524.C1	unclassified
44510	2.55	0.09	0.0030	0.0193	1.30	PKRvB6	custom - double-stranded RNA-dependent protein kinase B
54747	3.04	0.07	0.0008	0.0081	1.29	all_v2.0.8328.C1	unclassified
44100	3.01	0.07	0.0009	0.0085	1.29	all_v2.0.15096.C1	Zgc:92790; n=4; Eumetazoa|Rep: Zgc:92790 - Danio rerio (Zebrafish) (Brachydanio rerio)
44595	3.39	0.05	0.0004	0.0045	1.29	STAT11	custom - Signal transducer and activator of transcription 1
49070	3.22	0.06	0.0005	0.0059	1.29	all_v2.0.7789.C2	unclassified
45071	2.93	0.07	0.0011	0.0100	1.29	all_v2.0.2894.C5	LOC451002; similar to very large inducible GTPase-1
42817	2.45	0.09	0.0039	0.0231	1.29	sb_gmapov_0012m19.pDNRm13r	Tubulin alpha chain; n=36; Fungi/Metazoa group|Rep: Tubulin alpha chain - Octopus dofleini (Giant octopus)
51351	2.73	0.07	0.0018	0.0142	1.29	all_v2.0.2935.C2	unclassified
52032	2.59	0.08	0.0026	0.0179	1.29	all_v2.0.5496.C1	unclassified
40291	2.57	0.09	0.0028	0.0186	1.29	sb_gmnbhkas_0004b06.pDNRm13r	PREDICTED: similar to CARS-Cyp; n=2; Gallus gallus|Rep: PREDICTED: similar to CARS-Cyp - Gallus gallus
54674	2.61	0.08	0.0025	0.0173	1.29	all_v2.0.9920.C1	unclassified
44043	2.57	0.08	0.0028	0.0186	1.28	all_v2.0.2008.C1	Zgc:92027; n=2; Danio rerio|Rep: Zgc:92027 - Danio rerio (Zebrafish) (Brachydanio rerio)
40571	3.18	0.06	0.0006	0.0063	1.28	all_v2.0.1957.C1	PREDICTED: similar to intercellular adhesion molecule 5, telencephalin; n=1; Monodelphis domestica|Rep: PREDICTED: similar to intercellular adhesion molecule 5, telencephalin - Monodelphis domestica
46972	2.91	0.07	0.0011	0.0102	1.28	sb_gmnbhkic_0013n08.pDNRF2	Unassigned protein
45642	2.39	0.09	0.0045	0.0258	1.28	all_v2.0.13397.C1.a	non-directed
39063	2.75	0.07	0.0017	0.0136	1.28	sb_gmnlkfta_0007l18.t7	MHC class Ia antigen; n=2; Gadus morhua|Rep: MHC class Ia antigen - Gadus morhua (Atlantic cod)
45088	2.61	0.08	0.0025	0.0173	1.28	sb_gmnlkric_0002a08.t7	LOC477822; similar to hyaluronan binding protein 2 [EC:3.4.21.-]; K08648 hyaluronan binding protein 2
36329	2.74	0.07	0.0018	0.0139	1.28	all_v2.0.14245.C1	CASP8 and FADD-like apoptosis regulator precursor (Cellular FLICE-like inhibitory protein) (c-FLIP) (Caspase-eight-related protein) (Casper) (Caspase-like apoptosis regulatory protein) (CLARP) (MACH-related inducer of toxicity) (MRIT) (Caspase homolog) (C; n=1; Takifugu rubripes|Rep: CASP8 and FADD-like apoptosis regulator precursor (Cellular FLICE-like inhibitory protein) (c-FLIP) (Caspase-eight-related protein) (Casper) (Caspase-like apoptosis regulatory protein) (CLARP) (MACH-related inducer of toxicity) (MRIT) (Caspase homolog) (C - Takifugu rubripes
43762	3.24	0.05	0.0005	0.0057	1.28	all_v2.0.3465.C	Zgc:158614; n=2; Danio rerio|Rep:

						1	Zgc:158614 - Danio rerio (Zebrafish) (Brachydanio rerio)
42213	2.57	0.08	0.0028	0.0188	1.27	sb_gmnbhkic_0010g06.pDNRF2	Similar to Tetraodon protein product CAG00085; n=1; Oncorhynchus mykiss|Rep: Similar to Tetraodon protein product CAG00085 - Oncorhynchus mykiss (Rainbow trout) (Salmo gairdneri)
38404	3.02	0.06	0.0009	0.0084	1.27	all_v2.0.1538.C1	Homolog of Homo sapiens TiTin isoform novex-2; n=1; Takifugu rubripes|Rep: Homolog of Homo sapiens TiTin isoform novex-2 - Takifugu rubripes
37328	2.47	0.08	0.0036	0.0221	1.27	all_v2.0.12699.C1	Gamma-glutamyltransferase 5 precursor (EC 2.3.2.2) (Gamma-glutamyltranspeptidase 5) (Gamma-glutamyltransferase-like activity 1) (GGT-rel) [Contains: Gamma-glutamyltransferase 5 heavy chain; Gamma- glutamyltransferase 5 light chain].; n=1; Takifugu rubripes|Rep: Gamma-glutamyltransferase 5 precursor (EC 2.3.2.2) (Gamma-glutamyltranspeptidase 5) (Gamma-glutamyltransferase-like activity 1) (GGT-rel) [Contains: Gamma-glutamyltransferase 5 heavy chain; Gamma- glutamyltransferase 5 light chain]. - Takifugu rubripes
41711	2.68	0.07	0.0021	0.0156	1.27	all_v2.0.13005.C1	Ras-related protein Rab-8B; n=17; Euteleostomi|Rep: Ras-related protein Rab-8B - Homo sapiens (Human)
48324	2.48	0.08	0.0036	0.0220	1.27	sb_gmnlla_0007f24.t7	Unassigned protein
47948	2.88	0.06	0.0012	0.0106	1.26	all_v2.0.17446.C1	Unassigned protein
41718	2.49	0.08	0.0035	0.0215	1.26	all_v2.0.12871.C1	RBM39 protein; n=4; Eutheria|Rep: RBM39 protein - Homo sapiens (Human)
35876	2.66	0.07	0.0022	0.0161	1.26	sb_gmapov_0011n05.pDNRF2	Alpha-2-macroglobulin receptor-associated protein precursor; n=6; Catarrhini|Rep: Alpha-2-macroglobulin receptor-associated protein precursor - Homo sapiens (Human)
46381	3.26	0.05	0.0005	0.0056	1.26	all_v2.0.15502.C1	tRNA 2'-phosphotransferase 1 (EC 2.7.1.160).; n=1; Takifugu rubripes
52993	2.50	0.07	0.0034	0.0213	1.26	all_v2.0.6512.C3	unclassified
53182	2.72	0.06	0.0019	0.0145	1.25	all_v2.0.55.C3	unclassified
53591	2.46	0.08	0.0037	0.0225	1.25	all_v2.0.8746.C1	unclassified
42568	2.82	0.06	0.0014	0.0119	1.25	all_v2.0.9385.C2	Thyroid receptor-interacting protein 13 (TRIP-13) (Thyroid hormone receptor interactor 13) (Human papillomavirus type 16 E1 protein- binding protein) (HPV16 E1 protein-binding protein) (16E1-BP).; n=1; Takifugu rubripes|Rep: Thyroid receptor-interacting protein 13 (TRIP-13) (Thyroid hormone receptor interactor 13) (Human papillomavirus type 16 E1 protein- binding protein) (HPV16 E1 protein-binding protein) (16E1-BP). - Takifugu rubripes
49122	2.44	0.07	0.0040	0.0237	1.25	all_v2.0.13533.C1	unclassified
38828	2.58	0.06	0.0027	0.0181	1.25	sb_gmnbmu_0008m08.pDNRm13r	LOC553437 protein; n=2; Danio rerio|Rep: LOC553437 protein - Brachydanio rerio (Zebrafish) (Danio rerio)
41613	2.48	0.07	0.0036	0.0220	1.24	sb_gmnbspic_0003h16.pDNRF2	Putative ATP-dependent RNA helicase DHX33 (EC 3.6.1.-) (DEAH box protein 33).; n=1; Takifugu rubripes|Rep: Putative ATP-dependent RNA helicase DHX33

							(EC 3.6.1.-) (DEAH box protein 33). - Takifugu rubripes
43254	2.51	0.07	0.0033	0.0208	1.24	sb_gmapov_0019a10.pDNRm13r	Wu:fc55e05-prov protein; n=2; Euteleostomi|Rep: Wu:fc55e05-prov protein - Xenopus laevis (African clawed frog)
46261	2.46	0.07	0.0037	0.0225	1.24	all_v2.0.10360.C1	si:rp71-45k5.3; si:rp71-45k5.3
49653	2.86	0.05	0.0013	0.0112	1.24	all_v2.0.11316.C1	unclassified
45302	3.05	0.04	0.0008	0.0078	1.24	sb_gmapov_0028e22.pDNRF2	LOC733975; novel protein similar to B9 protein domain family
36028	2.46	0.07	0.0037	0.0225	1.24	all_v2.0.3.C4	ATP synthase subunit beta, mitochondrial precursor; n=27; Euteleostomi|Rep: ATP synthase subunit beta, mitochondrial precursor - Homo sapiens (Human)
41303	2.40	0.07	0.0044	0.0255	1.23	sb_gmnlskic_0005k05.pDNRm13r	Presequence protease, mitochondrial precursor; n=7; Danio rerio|Rep: Presequence protease, mitochondrial precursor - Brachydanio rerio (Zebrafish) (Danio rerio)
54245	2.87	0.05	0.0013	0.0110	1.23	all_v2.0.435.C1	unclassified
50861	2.81	0.05	0.0015	0.0122	1.22	all_v2.0.13653.C1	unclassified
51142	2.55	0.06	0.0030	0.0194	1.22	all_v2.0.7409.C1	unclassified
50989	2.93	0.04	0.0011	0.0099	1.22	all_v2.0.2653.C1	unclassified
54167	2.69	0.05	0.0021	0.0153	1.22	all_v2.0.3108.C1	unclassified
49688	3.04	0.04	0.0008	0.0081	1.22	all_v2.0.2053.C1	unclassified
51151	2.60	0.05	0.0026	0.0176	1.22	all_v2.0.11681.C1	unclassified
43280	2.43	0.05	0.0040	0.0239	1.19	all_v2.0.3981.C1	ZFP313 protein; n=1; Oncorhynchus mykiss|Rep: ZFP313 protein - Oncorhynchus mykiss (Rainbow trout) (Salmo gairdneri)
40277	2.75	0.03	0.0017	0.0135	1.19	sb_gmnbmu_0014b23.pDNRm13r	PREDICTED: similar to Calcineurin B subunit isoform 1 (Protein phosphatase 2B regulatory subunit 1) (Protein phosphatase 3 regulatory subunit B alpha isoform 1); n=1; Canis familiaris|Rep: PREDICTED: similar to Calcineurin B subunit isoform 1 (Protein phosphatase 2B regulatory subunit 1) (Protein phosphatase 3 regulatory subunit B alpha isoform 1) - Canis familiaris
43142	2.38	0.05	0.0047	0.0265	1.19	all_v2.0.13901.C1	Vacuolar ATP synthase subunit C 1 (EC 3.6.3.14) (V-ATPase subunit C 1) (Vacuolar proton pump subunit C 1).; n=1; Takifugu rubripes|Rep: Vacuolar ATP synthase subunit C 1 (EC 3.6.3.14) (V-ATPase subunit C 1) (Vacuolar proton pump subunit C 1). - Takifugu rubripes
39883	-3.56	0.03	0.0003	0.0036	0.81	sb_gmapov_0018c14.pDNRF2	Peptidase S8 and S53, subtilisin, kexin, sedolisin; n=1; Herpetosiphon aurantiacus ATCC 23779|Rep: Peptidase S8 and S53, subtilisin, kexin, sedolisin - Herpetosiphon aurantiacus ATCC 23779
35591	-3.07	0.05	0.0008	0.0076	0.80	all_v2.0.6634.C1	39S ribosomal protein L2, mitochondrial precursor (L2mt) (MRP-L2).; n=1; Takifugu rubripes|Rep: 39S ribosomal protein L2, mitochondrial precursor (L2mt) (MRP-L2). - Takifugu rubripes
44414	-3.03	0.05	0.0008	0.0082	0.79	all_v2.0.10891.C1	Competence multi-domain protein
40272	-3.10	0.05	0.0007	0.0073	0.79	all_v2.0.1446.C1	PREDICTED: similar to C10ORF6; n=2; Gallus gallus|Rep: PREDICTED: similar to C10ORF6 - Gallus gallus

48062	-3.39	0.04	0.0004	0.0045	0.79	sb_gmnlem_0024j04.t7	Unassigned protein
51711	-3.16	0.05	0.0006	0.0066	0.79	all_v2.0.8976.C1	unclassified
39377	-3.38	0.05	0.0004	0.0046	0.78	sb_gmnblits_0026k02.pDNRF2	Novel KRAB box and zinc finger, C2H2 type domain containing protein; n=4; Mus musculus|Rep: Novel KRAB box and zinc finger, C2H2 type domain containing protein - Mus musculus (Mouse)
40225	-3.06	0.06	0.0008	0.0077	0.78	sb_gmnlsras_0004g22.t7	PREDICTED: similar to APC; n=1; Ornithorhynchus anatinus|Rep: PREDICTED: similar to APC - Ornithorhynchus anatinus
51411	-3.02	0.06	0.0008	0.0083	0.78	all_v2.0.1601.C1	unclassified
46908	-3.15	0.05	0.0006	0.0066	0.78	all_v2.0.8789.C1	Unassigned protein
48747	-3.12	0.06	0.0007	0.0070	0.77	sb_gmnlla_0038l18.t7	Unassigned protein
37298	-3.40	0.06	0.0004	0.0044	0.76	sb_gmnlbfic_0007l13.t7	Gabpa protein; n=3; Danio rerio|Rep: Gabpa protein - Danio rerio (Zebrafish) (Brachydanio rerio)
49253	-3.83	0.05	0.0002	0.0027	0.75	all_v2.0.1504.C1	unclassified
47132	-3.09	0.08	0.0007	0.0075	0.75	all_v2.0.4.C255	Unassigned protein
48597	-3.18	0.07	0.0006	0.0063	0.75	sb_gmnbmu_0002n03.pDNRF2	Unassigned protein
45671	-3.20	0.07	0.0006	0.0061	0.75	all_v2.0.18547.C1	non-directed
40188	-3.07	0.08	0.0008	0.0076	0.74	sb_gmnlsras_0001c11.t7	PREDICTED: similar to Activated in Blocked Unfolded protein response family member (abu-2); n=1; Rattus norvegicus|Rep: PREDICTED: similar to Activated in Blocked Unfolded protein response family member (abu-2) - Rattus norvegicus
41523	-3.17	0.08	0.0006	0.0065	0.74	sb_gmnlskic_0009p24.pDNRF2	Protein phosphatase 5; n=2; Drosophila melanogaster|Rep: Protein phosphatase 5 - Drosophila melanogaster (Fruit fly)
40591	-3.42	0.07	0.0003	0.0043	0.74	all_v2.0.4944.C1	PREDICTED: similar to KIAA0569 protein; n=3; Danio rerio|Rep: PREDICTED: similar to KIAA0569 protein - Danio rerio
43212	-3.33	0.07	0.0004	0.0049	0.74	sb_gmapte_0006b24.pDNRm13r	Vitellogenin B; n=1; Melanogrammus aeglefinus|Rep: Vitellogenin B - Melanogrammus aeglefinus (Haddock)
47796	-3.20	0.08	0.0006	0.0061	0.74	all_v2.0.16090.C1	Unassigned protein
52325	-3.82	0.06	0.0002	0.0027	0.73	all_v2.0.11802.C1	unclassified
42420	-3.08	0.09	0.0007	0.0075	0.73	sb_gmnlbfic_0006b05.t7	Suppressor of defective silencing 3 homolog; n=7; Clupeocephala|Rep: Suppressor of defective silencing 3 homolog - Danio rerio (Zebrafish) (Brachydanio rerio)
47500	-3.79	0.06	0.0002	0.0028	0.73	sb_gmnlla_0035n08.t7	Unassigned protein
35961	-3.09	0.09	0.0007	0.0075	0.73	all_v2.0.1089.C1	Apolipoprotein C-II; n=1; Takifugu rubripes|Rep: Apolipoprotein C-II - Fugu rubripes (Japanese pufferfish) (Takifugu rubripes)
36033	-3.74	0.06	0.0002	0.0029	0.73	all_v2.0.4258.C2	ATP synthase, H+ transporting, mitochondrial F0 complex, subunit c; n=3; Cyprinidae|Rep: ATP synthase, H+ transporting, mitochondrial F0 complex, subunit c - Danio rerio (Zebrafish) (Brachydanio rerio)
49327	-3.22	0.09	0.0005	0.0060	0.73	all_v2.0.7420.C1	unclassified

46436	-3.75	0.07	0.0002	0.0029	0.72	all_v2.0.15699.C1	Unassigned protein
51280	-4.21	0.05	0.0001	0.0019	0.72	all_v2.0.8472.C2	unclassified
52978	-3.20	0.09	0.0006	0.0061	0.72	all_v2.0.7485.C1	unclassified
52072	-3.47	0.08	0.0003	0.0041	0.72	all_v2.0.10070.C1	unclassified
42088	-3.60	0.08	0.0002	0.0034	0.72	all_v2.0.17743.C1	Serine/threonine-protein kinase Nek2; n=13; Eutheria|Rep: Serine/threonine-protein kinase Nek2 - Homo sapiens (Human)
53356	-3.43	0.08	0.0003	0.0043	0.72	all_v2.0.2372.C2	unclassified
40941	-3.35	0.08	0.0004	0.0048	0.72	all_v2.0.3046.C2	PREDICTED: similar to ribosomal protein L15; n=1; Macaca mulatta|Rep: PREDICTED: similar to ribosomal protein L15 - Macaca mulatta
52454	-3.00	0.11	0.0009	0.0086	0.71	all_v2.0.10880.C1	unclassified
46084	-3.02	0.11	0.0009	0.0084	0.71	sb_gmnlsfas_0004c10.t7	prothymosin alpha like-1 protein [Danio rerio]
53882	-3.63	0.09	0.0002	0.0033	0.70	all_v2.0.5809.C1	unclassified
54547	-3.13	0.11	0.0007	0.0069	0.70	all_v2.0.16354.C1	unclassified
44670	-3.65	0.09	0.0002	0.0032	0.69	sb_gmnbhkic_0005f19.pDNRF2	DUF1055 domain containing protein
38822	-3.21	0.11	0.0005	0.0060	0.69	sb_gmnbmd_0007e01.pDNRm13r	LOC553307 protein; n=4; Danio rerio|Rep: LOC553307 protein - Brachydanio rerio (Zebrafish) (Danio rerio)
48418	-3.22	0.11	0.0005	0.0059	0.69	sb_gmnlla_0047b11.t7	Unassigned protein
41210	-3.90	0.08	0.0001	0.0025	0.69	all_v2.0.5638.C1	PREDICTED: similar to VPS13C-2A protein isoform 1; n=3; Bos taurus|Rep: PREDICTED: similar to VPS13C-2A protein isoform 1 - Bos taurus
42046	-3.13	0.11	0.0007	0.0069	0.69	all_v2.0.18763.C1	Selenoprotein P precursor (SeP).; n=1; Takifugu rubripes|Rep: Selenoprotein P precursor (SeP). - Takifugu rubripes
44930	-3.43	0.10	0.0003	0.0043	0.69	all_v2.0.16304.C1	I-set domain containing protein
36386	-3.43	0.10	0.0003	0.0043	0.69	all_v2.0.7753.C2	CC chemokine; n=1; Pseudosciaena crocea (Croceine croaker)|Rep: CC chemokine - Pseudosciaena crocea (Croceine croaker)
52976	-3.06	0.12	0.0008	0.0077	0.68	all_v2.0.1798.C1	unclassified
41743	-3.00	0.13	0.0009	0.0086	0.68	all_v2.0.848.C2	Reticulon 4 receptor; n=5; Danio rerio|Rep: Reticulon 4 receptor - Danio rerio (Zebrafish) (Brachydanio rerio)
36776	-4.07	0.08	0.0001	0.0021	0.68	sb_gmnbpcic_0001o08.pDNRF2	Cytochrome P450 7A1 (Cholesterol 7-alpha-monooxygenase) (CYPVII) (EC 1.14.13.17) (Cholesterol 7-alpha-hydroxylase).; n=1; Takifugu rubripes|Rep: Cytochrome P450 7A1 (Cholesterol 7-alpha-monooxygenase) (CYPVII) (EC 1.14.13.17) (Cholesterol 7-alpha-hydroxylase). - Takifugu rubripes
52493	-4.17	0.08	0.0001	0.0020	0.68	all_v2.0.1280.C2	unclassified
47663	-3.92	0.09	0.0001	0.0024	0.68	sb_gmlbgits_0003f15.sp6	Unassigned protein
45113	-3.64	0.10	0.0002	0.0033	0.68	sb_gmapov_0018o17.pDNRF2	LOC488397; similar to Methyltransferase-like protein 2
44982	-3.28	0.12	0.0005	0.0054	0.67	all_v2.0.4062.C1	LOC100001974; similar to DAZ associated protein 2
40970	-4.15	0.08	0.0001	0.0020	0.67	sb_gmapht_001	PREDICTED: similar to RNA-binding

						6c24.pDNRF2	protein EWS; n=1; Mus musculus|Rep: PREDICTED: similar to RNA-binding protein EWS - Mus musculus
50372	-3.13	0.13	0.0007	0.0069	0.67	all_v2.0.3379.C2	unclassified
53024	-3.56	0.11	0.0003	0.0036	0.67	all_v2.0.5664.C1	unclassified
48311	-3.22	0.13	0.0005	0.0060	0.67	sb_gmnlla_0013d16.t7	Unassigned protein
55159	-3.90	0.10	0.0001	0.0025	0.66	all_v2.0.1965.C1	unclassified
53027	-3.62	0.11	0.0002	0.0033	0.66	all_v2.0.1406.C1	unclassified
45907	-3.67	0.10	0.0002	0.0031	0.66	all_v2.0.5279.C2.a	non-directed
51606	-3.26	0.13	0.0005	0.0056	0.66	all_v2.0.4073.C1	unclassified
42233	-3.12	0.13	0.0007	0.0071	0.65	sb_gmnbpcic_0005o11.pDNRF2	Skint 2 isoform b precursor; n=6; Mus musculus|Rep: Skint 2 isoform b precursor - Mus musculus (Mouse)
45751	-4.33	0.08	0.0001	0.0018	0.65	all_v2.0.17438.C1.a	non-directed
46142	-3.36	0.13	0.0004	0.0047	0.65	sb_gmapte_0013b05.pDNRF2	reverse transcriptase [Oryzias latipes]
43245	-4.34	0.09	0.0001	0.0018	0.65	all_v2.0.2844.C1	WD repeat-containing protein 82; n=16; Euteleostomi|Rep: WD repeat-containing protein 82 - Homo sapiens (Human)
38713	-3.12	0.13	0.0007	0.0070	0.65	all_v2.0.1579.C2	KH domain-containing, RNA-binding, signal transduction-associated protein 1 (p21 Ras GTPase-activating protein-associated p62) (GAP- associated tyrosine phosphoprotein p62) (Src-associated in mitosis 68 kDa protein) (Sam68) (p68).; n=1; Takifugu rubripes|Rep: KH domain-containing, RNA-binding, signal transduction-associated protein 1 (p21 Ras GTPase-activating protein-associated p62) (GAP- associated tyrosine phosphoprotein p62) (Src-associated in mitosis 68 kDa protein) (Sam68) (p68). - Takifugu rubripes
51999	-3.39	0.12	0.0004	0.0045	0.65	all_v2.0.10328.C1	unclassified
45693	-3.26	0.14	0.0005	0.0055	0.65	all_v2.0.18093.C1	non-directed
50810	-3.15	0.14	0.0006	0.0067	0.65	all_v2.0.4720.C1	unclassified
46695	-3.70	0.11	0.0002	0.0030	0.65	all_v2.0.5132.C1	Unassigned protein
41925	-3.22	0.14	0.0005	0.0059	0.65	sb_gmnlmfta_0004f17.t7	RING finger protein 11; n=20; Euteleostomi|Rep: RING finger protein 11 - Homo sapiens (Human)
50513	-3.27	0.14	0.0005	0.0055	0.64	all_v2.0.6080.C2	unclassified
51502	-4.24	0.09	0.0001	0.0019	0.64	all_v2.0.16235.C1	unclassified
51036	-3.27	0.15	0.0005	0.0054	0.64	all_v2.0.6393.C1	unclassified
37140	-3.09	0.15	0.0007	0.0075	0.64	all_v2.0.2749.C1	Extracellular matrix protein 1 precursor (Secretory component p85).; n=1; Takifugu rubripes|Rep: Extracellular matrix protein 1 precursor (Secretory component p85). - Takifugu rubripes
53699	-3.09	0.16	0.0007	0.0075	0.64	all_v2.0.11216.C1	unclassified
53248	-3.14	0.15	0.0006	0.0068	0.63	all_v2.0.5766.C1	unclassified
43896	-4.10	0.10	0.0001	0.0021	0.63	all_v2.0.16541.C1	Zgc:64130; n=1; Danio rerio|Rep: Zgc:64130 - Danio rerio (Zebrafish) (Brachydanio rerio)

51181	-3.92	0.12	0.0001	0.0024	0.63	all_v2.0.16785.C1	unclassified
51568	-4.44	0.09	0.0001	0.0017	0.63	all_v2.0.6708.C1	unclassified
36880	-3.05	0.15	0.0008	0.0078	0.63	sb_gmnlsric_0003a20.t7	DNA damage-binding protein 1; n=21; Tetrapoda|Rep: DNA damage-binding protein 1 - Homo sapiens (Human)
42217	-4.54	0.09	0.0001	0.0016	0.62	sb_gmnbspic_0013h24.pDNRm13r	Simple type II keratin K8a; n=3; Oncorhynchus|Rep: Simple type II keratin K8a - Oncorhynchus mykiss (Rainbow trout) (Salmo gairdneri)
41460	-3.00	0.17	0.0009	0.0085	0.62	all_v2.0.10923.C2	Protein FAM13C1.; n=1; Xenopus tropicalis|Rep: Protein FAM13C1. - Xenopus tropicalis
37459	-4.01	0.11	0.0001	0.0022	0.62	all_v2.0.5186.C1	GPI ethanolamine phosphate transferase 1 (EC 2.-.-.-) (Phosphatidylinositol-glycan biosynthesis class N protein) (PIG-N) (MCD4 homolog).; n=1; Takifugu rubripes|Rep: GPI ethanolamine phosphate transferase 1 (EC 2.-.-.-) (Phosphatidylinositol-glycan biosynthesis class N protein) (PIG-N) (MCD4 homolog). - Takifugu rubripes
38986	-4.18	0.11	0.0001	0.0020	0.62	all_v2.0.2756.C1	Methyl-CpG binding protein MBD3; n=3; Xenopus|Rep: Methyl-CpG binding protein MBD3 - Xenopus laevis (African clawed frog)
38411	-3.20	0.16	0.0006	0.0061	0.62	sb_gmapht_0010p17.pDNRF2	Homolog of Homo sapiens TiTin isoform novex-2; n=1; Takifugu rubripes|Rep: Homolog of Homo sapiens TiTin isoform novex-2 - Takifugu rubripes
36585	-4.35	0.10	0.0001	0.0018	0.61	all_v2.0.18719.C1	Collagen alpha-1(I) chain precursor (Alpha-1 type I collagen).; n=1; Takifugu rubripes|Rep: Collagen alpha-1(I) chain precursor (Alpha-1 type I collagen). - Takifugu rubripes
51529	-3.37	0.15	0.0004	0.0046	0.61	all_v2.0.6456.C1	unclassified
53664	-3.18	0.17	0.0006	0.0063	0.61	all_v2.0.9611.C1	unclassified
38008	-3.35	0.15	0.0004	0.0048	0.61	sb_gmnbmd_0014k10.pDNRm13r	Homolog of Homo sapiens Calpain 1, large [catalytic] subunit; n=1; Takifugu rubripes|Rep: Homolog of Homo sapiens Calpain 1, large [catalytic] subunit - Takifugu rubripes
35883	-3.79	0.13	0.0002	0.0028	0.61	all_v2.0.2916.C1	Alpha-enolase; n=4; Rattus norvegicus|Rep: Alpha-enolase - Rattus norvegicus (Rat)
35733	-3.20	0.17	0.0005	0.0061	0.61	sb_gmnbmu_0001m08.pDNRF2	Acetylcholine receptor subunit alpha precursor; n=2; Clupeocephala|Rep: Acetylcholine receptor subunit alpha precursor - Brachydanio rerio (Zebrafish) (Danio rerio)
44357	-3.92	0.12	0.0001	0.0024	0.61	all_v2.0.4599.C1	Caldesmon multi-domain protein
46425	-4.18	0.12	0.0001	0.0020	0.60	all_v2.0.13812.C1	Unassigned protein
41688	-3.47	0.15	0.0003	0.0041	0.60	all_v2.0.4385.C1	Ras GTPase-activating-like protein IQGAP2.; n=1; Takifugu rubripes|Rep: Ras GTPase-activating-like protein IQGAP2. - Takifugu rubripes
47021	-3.47	0.15	0.0003	0.0041	0.60	sb_gmnbhkas_0005f08.pDNRF2	Unassigned protein
35935	-4.21	0.12	0.0001	0.0020	0.60	all_v2.0.3080.C1	Annexin max3; n=1; Oryzias latipes|Rep: Annexin max3 - Oryzias latipes (Medaka fish) (Japanese ricefish)
37449	-3.82	0.13	0.0002	0.0027	0.60	sb_gmnlpbia_0002g08.t7	Golgi-specific brefeldin A-resistance guanine nucleotide exchange factor 1; n=29; Theria|Rep: Golgi-specific brefeldin

							A-resistance guanine nucleotide exchange factor 1 - Homo sapiens (Human)
46669	-3.90	0.13	0.0001	0.0025	0.60	sb_gmapht_0029n18.pDNRF2	Unassigned protein
51982	-4.32	0.11	0.0001	0.0018	0.60	all_v2.0.6362.C1	unclassified
37695	-3.54	0.15	0.0003	0.0037	0.60	all_v2.0.14920.C1	Homolog of Brachydanio rerio Cyclin D1.; n=1; Takifugu rubripes|Rep: Homolog of Brachydanio rerio Cyclin D1. - Takifugu rubripes
46750	-3.99	0.13	0.0001	0.0023	0.59	sb_gmnbspic_0013h20.pDNRF2	Unassigned protein
52562	-3.30	0.18	0.0004	0.0052	0.59	all_v2.0.8055.C1	unclassified
45607	-3.02	0.19	0.0009	0.0084	0.59	all_v2.0.11441.C1	non-directed
51735	-4.34	0.12	0.0001	0.0018	0.59	all_v2.0.9513.C1	unclassified
50500	-3.25	0.18	0.0005	0.0056	0.59	all_v2.0.9176.C1	unclassified
36693	-3.81	0.14	0.0002	0.0027	0.59	all_v2.0.6166.C1	CUB and sushi domain-containing protein 3 precursor (CUB and sushi multiple domains protein 3).; n=1; Takifugu rubripes|Rep: CUB and sushi domain-containing protein 3 precursor (CUB and sushi multiple domains protein 3). - Takifugu rubripes
43747	-4.78	0.11	0.0001	0.0015	0.58	sb_gmnlsric_0002i16.t7	Zgc:158393; n=5; Danio rerio|Rep: Zgc:158393 - Brachydanio rerio (Zebrafish) (Danio rerio)
43654	-3.92	0.15	0.0001	0.0024	0.58	all_v2.0.5364.C1	Zgc:153093; n=2; Danio rerio|Rep: Zgc:153093 - Danio rerio (Zebrafish) (Brachydanio rerio)
43904	-3.49	0.19	0.0003	0.0040	0.57	all_v2.0.16778.C1	Zgc:65861; n=1; Danio rerio|Rep: Zgc:65861 - Danio rerio (Zebrafish) (Brachydanio rerio)
50459	-3.56	0.17	0.0003	0.0036	0.57	all_v2.0.1012.C1	unclassified
54377	-4.17	0.14	0.0001	0.0020	0.56	all_v2.0.82.C1	unclassified
50418	-3.24	0.20	0.0005	0.0057	0.56	all_v2.0.7784.C1	unclassified
49561	-4.21	0.14	0.0001	0.0020	0.55	all_v2.0.3485.C1	unclassified
49797	-3.69	0.18	0.0002	0.0031	0.55	all_v2.0.4315.C1	unclassified
49079	-3.19	0.24	0.0006	0.0062	0.55	all_v2.0.8699.C1	unclassified
49726	-3.11	0.21	0.0007	0.0071	0.55	all_v2.0.9180.C1	unclassified
35934	-5.37	0.10	0.0000	0.0013	0.55	all_v2.0.3080.C3	Annexin max3; n=1; Oryzias latipes|Rep: Annexin max3 - Oryzias latipes (Medaka fish) (Japanese ricefish)
50184	-3.02	0.21	0.0009	0.0084	0.54	all_v2.0.7282.C2	unclassified
39310	-4.54	0.14	0.0001	0.0016	0.54	all_v2.0.13511.C1	Neuroblast differentiation-associated protein AHNAK (Desmoyokin) (Fragments).; n=1; Takifugu rubripes|Rep: Neuroblast differentiation-associated protein AHNAK (Desmoyokin) (Fragments). - Takifugu rubripes
37939	-3.85	0.17	0.0002	0.0026	0.54	sb_gmnlem_0014k01.sp6	Homolog of Gallus gallus Stathmin.; n=1; Takifugu rubripes|Rep: Homolog of Gallus gallus Stathmin. - Takifugu rubripes
48737	-4.10	0.16	0.0001	0.0021	0.54	sb_gmlbgits_0004l14.sp6	Unassigned protein
37254	-3.54	0.19	0.0003	0.0037	0.53	all_v2.0.2576.C1	Four and a half LIM domains a; n=3; Danio rerio|Rep: Four and a half LIM domains a - Danio rerio (Zebrafish) (Brachydanio rerio)

35890	-5.36	0.11	0.0000	0.0014	0.52	sb_gmnbmu_0014g13.pDNRF2	Amine oxidase [flavin-containing]; n=1; Oncorhynchus mykiss|Rep: Amine oxidase [flavin-containing] - Oncorhynchus mykiss (Rainbow trout) (Salmo gairdneri)
54441	-3.33	0.25	0.0004	0.0049	0.52	all_v2.0.10503.C1	unclassified
41032	-5.91	0.10	0.0000	0.0013	0.52	all_v2.0.6546.C1	PREDICTED: similar to spermatogenesis and oogenesis specific basic helix-loop-helix 1; n=1; Rattus norvegicus|Rep: PREDICTED: similar to spermatogenesis and oogenesis specific basic helix-loop-helix 1 - Rattus norvegicus
37518	-5.11	0.13	0.0000	0.0014	0.51	sb_gmnlsfic_0002k09.t7	Guanine nucleotide-binding protein G(o) subunit alpha 2; n=18; Tetrapoda|Rep: Guanine nucleotide-binding protein G(o) subunit alpha 2 - Homo sapiens (Human)
55146	-3.51	0.22	0.0003	0.0038	0.51	all_v2.0.712.C1	unclassified
37965	-5.05	0.14	0.0000	0.0014	0.50	all_v2.0.9443.C1	Homolog of Homo sapiens Alanine aminotransferase; n=1; Takifugu rubripes|Rep: Homolog of Homo sapiens Alanine aminotransferase - Takifugu rubripes
42849	-3.67	0.20	0.0002	0.0031	0.50	all_v2.0.1438.C1	Type 1 collagen alpha 2; n=1; Paralichthys olivaceus|Rep: Type 1 collagen alpha 2 - Paralichthys olivaceus (Japanese flounder)
37428	-3.01	0.28	0.0009	0.0084	0.50	all_v2.0.11762.C1	glycosyltransferase 1 domain containing 1; n=1; Takifugu rubripes|Rep: glycosyltransferase 1 domain containing 1 - Takifugu rubripes
54918	-3.54	0.22	0.0003	0.0037	0.50	all_v2.0.897.C1	unclassified
42517	-3.10	0.29	0.0007	0.0073	0.48	all_v2.0.2825.C1	Tetraspanin-6 (Tspan-6) (Transmembrane 4 superfamily member 6) (T245 protein) (Tetraspanin TM4-D) (A15 homolog).; n=1; Takifugu rubripes|Rep: Tetraspanin-6 (Tspan-6) (Transmembrane 4 superfamily member 6) (T245 protein) (Tetraspanin TM4-D) (A15 homolog). - Takifugu rubripes
49669	-3.16	0.26	0.0006	0.0066	0.48	all_v2.0.13190.C1	unclassified
48024	-5.63	0.13	0.0000	0.0013	0.48	sb_gmnbpcic_0007c20.pDNRm13r	Unassigned protein
45826	-3.25	0.31	0.0005	0.0056	0.47	all_v2.0.15558.C1	non-directed
36958	-3.42	0.26	0.0003	0.0043	0.47	all_v2.0.5448.C1	Dynein light chain roadblock-type 2; n=9; Tetrapoda|Rep: Dynein light chain roadblock-type 2 - Homo sapiens (Human)
37172	-3.57	0.25	0.0003	0.0036	0.47	all_v2.0.492.C1	Fatty acid binding protein 11; n=1; Danio rerio|Rep: Fatty acid binding protein 11 - Danio rerio (Zebrafish) (Brachydanio rerio)
49819	-3.36	0.27	0.0004	0.0047	0.46	all_v2.0.11870.C1	unclassified
51386	-3.07	0.28	0.0008	0.0077	0.45	all_v2.0.3084.C1	unclassified
39361	-4.10	0.24	0.0001	0.0021	0.44	sb_gmapte_0010h11.pDNRF2	Novel AMP-binding enzyme domain containing protein; n=2; Danio rerio|Rep: Novel AMP-binding enzyme domain containing protein - Danio rerio (Zebrafish) (Brachydanio rerio)
47511	-3.18	0.36	0.0006	0.0063	0.43	sb_gmnlla_0025g11.t7	Unassigned protein
38596	-4.81	0.21	0.0001	0.0015	0.42	all_v2.0.8452.C1	Interferon induced protein 2; n=1; Ictalurus punctatus|Rep: Interferon induced protein 2 - Ictalurus punctatus (Channel catfish)
42848	-4.10	0.26	0.0001	0.0021	0.39	all_v2.0.1438.C	Type 1 collagen alpha 2; n=1; Paralichthys

						2	olivaceus|Rep: Type 1 collagen alpha 2 - Paralichthys olivaceus (Japanese flounder)
45392	-6.82	0.14	0.0000	0.0012	0.38	all_v2.0.341.C5	Metallothio domain containing protein
50333	-4.02	0.30	0.0001	0.0022	0.37	all_v2.0.6062.C1	unclassified
38960	-3.00	0.41	0.0009	0.0086	0.35	all_v2.0.8721.C1	Membrane cofactor protein precursor (CD46 antigen) (Trophoblast leukocyte common antigen) (TLX).; n=1; Xenopus tropicalis|Rep: Membrane cofactor protein precursor (CD46 antigen) (Trophoblast leukocyte common antigen) (TLX). - Xenopus tropicalis
42857	-9.70	0.11	0.0000	0.0012	0.33	all_v2.0.7963.C1	Type I keratin S8; n=1; Oncorhynchus mykiss|Rep: Type I keratin S8 - Oncorhynchus mykiss (Rainbow trout) (Salmo gairdneri)
47254	-3.26	0.57	0.0005	0.0055	0.30	sb_gmnlskic_0009m15.pDNRm13r	Unassigned protein

[a]Fold-changes are presented as outputted by siggenes and were calculated as pIC/PBS

Supplemental Table S5. 246 genes differentially expressed between fish injected with pIC or PBS at 16°C and sampled at 24HPI

Probe ID	d-value	Stdev	Raw p	q-value	Fold Change[a]	Sequence Name	Gene Name
38604	13.72	0.36	0.0000	0.0015	38.85	all_v2.0.14.CB2	Interferon stimulated gene 15; n=1; Gadus morhua|Rep: Interferon stimulated gene 15 - Gadus morhua (Atlantic cod)
45882	9.62	0.50	0.0000	0.0016	31.10	all_v2.0.5246.C1	non-directed
38605	11.71	0.38	0.0000	0.0015	25.29	all_v2.0.14.C13	Interferon stimulated gene 15; n=1; Gadus morhua|Rep: Interferon stimulated gene 15 - Gadus morhua (Atlantic cod)
41316	6.11	0.54	0.0000	0.0025	12.55	all_v2.0.1009.C5	Probable ATP-dependent helicase LGP2 (EC 3.6.1.-) (Protein D11Lgp2 homolog).; n=1; Takifugu rubripes|Rep: Probable ATP-dependent helicase LGP2 (EC 3.6.1.-) (Protein D11Lgp2 homolog). - Takifugu rubripes
38614	13.59	0.24	0.0000	0.0015	12.35	all_v2.0.7062.C2	Interferon stimulated gene 15; n=1; Gadus morhua|Rep: Interferon stimulated gene 15 - Gadus morhua (Atlantic cod)
44590	6.34	0.50	0.0000	0.0023	12.13	DXH4a	custom - probable ATP-dependent RNA helicase DHX
38611	6.83	0.48	0.0000	0.0019	11.80	all_v2.0.1767.C1	Interferon stimulated gene 15; n=1; Gadus morhua|Rep: Interferon stimulated gene 15 - Gadus morhua (Atlantic cod)
48390	5.91	0.62	0.0000	0.0027	11.53	all_v2.0.15354.C1	Unassigned protein
47164	4.91	0.81	0.0000	0.0051	11.19	all_v2.0.6027.C6	Unassigned protein
38609	12.44	0.24	0.0000	0.0015	10.49	all_v2.0.14.C3	Interferon stimulated gene 15; n=1; Gadus morhua|Rep: Interferon stimulated gene 15 - Gadus morhua (Atlantic cod)
38356	5.37	0.60	0.0000	0.0036	9.88	all_v2.0.415.C6	Homolog of Homo sapiens Spastic ataxia of Charlevoix-Saguenay; n=1; Takifugu rubripes|Rep: Homolog of Homo sapiens Spastic ataxia of Charlevoix-Saguenay - Takifugu rubripes
38613	11.08	0.26	0.0000	0.0015	9.45	all_v2.0.14.CB5	Interferon stimulated gene 15; n=1; Gadus morhua|Rep: Interferon stimulated gene 15 - Gadus morhua (Atlantic cod)
38606	12.80	0.22	0.0000	0.0015	9.30	all_v2.0.14.CB4	Interferon stimulated gene 15; n=1; Gadus morhua|Rep: Interferon stimulated gene 15 - Gadus morhua (Atlantic cod)
38608	15.21	0.17	0.0000	0.0015	8.87	all_v2.0.14.C12	Interferon stimulated gene 15; n=1; Gadus morhua|Rep: Interferon stimulated gene 15 - Gadus morhua (Atlantic cod)
44453	5.01	0.75	0.0000	0.0045	7.63	CK10a	custom - CC chemokine
44589	4.45	0.56	0.0001	0.0080	7.32	DXH3b	custom - probable ATP-dependent RNA helicase DHX
44448	4.92	0.71	0.0000	0.0050	7.28	CK10ad	custom - CC chemokine
43785	3.45	0.59	0.0009	0.0255	7.25	all_v2.0.2417.C1	Zgc:162304 protein; n=1; Danio rerio|Rep: Zgc:162304 protein - Danio rerio (Zebrafish) (Brachydanio rerio)
38638	5.30	0.48	0.0000	0.0037	6.92	all_v2.0.638.C1	Interleukin-8 variant 2; n=3; Ictalurus punctatus|Rep: Interleukin-8 variant 2 - Ictalurus punctatus (Channel catfish)
35718	8.67	0.29	0.0000	0.0016	6.12	sb_gmnbhkas_0013h13.pDNRF2	A disintegrin and metalloproteinase domain 8; n=1; Danio rerio|Rep: A disintegrin and metalloproteinase domain 8 - Brachydanio rerio (Zebrafish) (Danio rerio)
47329	9.48	0.24	0.0000	0.0016	5.81	all_v2.0.415.C3	Unassigned protein
44588	5.29	0.38	0.0000	0.0037	5.22	DXH2b	custom - probable ATP-dependent RNA helicase DHX
41269	5.55	0.40	0.0000	0.0033	5.07	sb_gmnlbfic_0004o20.t7	PREDICTED: similar to zinc finger, NFX1-type containing 1; n=3; Laurasiatheria|Rep: PREDICTED: similar to zinc finger, NFX1-

							type containing 1 - Equus caballus
41314	4.70	0.47	0.0001	0.0062	5.06	all_v2.0.5598.C1	Probable ATP-dependent helicase LGP2 (EC 3.6.1.-) (Protein D11Lgp2 homolog).; n=1; Takifugu rubripes|Rep: Probable ATP-dependent helicase LGP2 (EC 3.6.1.-) (Protein D11Lgp2 homolog). - Takifugu rubripes
44859	8.17	0.26	0.0000	0.0016	4.98	all_v2.0.314.C1	HECT domain containing protein
46200	5.90	0.37	0.0000	0.0027	4.90	all_v2.0.14575.C1	SACS; sacsin
38603	8.30	0.23	0.0000	0.0016	4.61	all_v2.0.14.CB7	Interferon stimulated gene 15; n=1; Gadus morhua|Rep: Interferon stimulated gene 15 - Gadus morhua (Atlantic cod)
38612	6.39	0.32	0.0000	0.0023	4.41	all_v2.0.14.CB3	Interferon stimulated gene 15; n=1; Gadus morhua|Rep: Interferon stimulated gene 15 - Gadus morhua (Atlantic cod)
39328	7.51	0.25	0.0000	0.0016	4.38	all_v2.0.2213.C1	NFX1-type zinc finger-containing protein 1; n=5; Eutheria|Rep: NFX1-type zinc finger-containing protein 1 - Homo sapiens (Human)
38285	5.11	0.38	0.0000	0.0041	4.18	sb_gmnlbfic_0007k16.t7	Homolog of Homo sapiens Probable ATP-dependent helicase LGP2; n=1; Takifugu rubripes|Rep: Homolog of Homo sapiens Probable ATP-dependent helicase LGP2 - Takifugu rubripes
48258	10.35	0.16	0.0000	0.0015	4.01	all_v2.0.14926.C1	Unassigned protein
38358	5.51	0.37	0.0000	0.0033	3.91	all_v2.0.9190.C1	Homolog of Homo sapiens Spastic ataxia of Charlevoix-Saguenay; n=1; Takifugu rubripes|Rep: Homolog of Homo sapiens Spastic ataxia of Charlevoix-Saguenay - Takifugu rubripes
38354	5.60	0.35	0.0000	0.0032	3.78	all_v2.0.707.C4	Homolog of Homo sapiens Spastic ataxia of Charlevoix-Saguenay; n=1; Takifugu rubripes|Rep: Homolog of Homo sapiens Spastic ataxia of Charlevoix-Saguenay - Takifugu rubripes
47384	6.52	0.28	0.0000	0.0022	3.78	all_v2.0.707.C1	Unassigned protein
44465	4.15	0.40	0.0002	0.0119	3.76	1AY614591_Gadus morhua CC chemokine type 2 mRNA, complete cds	custom - CC chemokine
44467	5.00	0.34	0.0000	0.0046	3.69	2AY614591_Gadus morhua CC chemokine type 2 mRNA, complete cds	custom - CC chemokine
38639	5.25	0.30	0.0000	0.0037	3.60	all_v2.0.638.CB1	Interleukin-8 variant 2; n=3; Ictalurus punctatus|Rep: Interleukin-8 variant 2 - Ictalurus punctatus (Channel catfish)
44471	5.70	0.29	0.0000	0.0031	3.60	1AY614592_Gadus morhua CC chemokine type 3 mRNA, complete cds	custom - CC chemokine
44456	4.81	0.34	0.0001	0.0055	3.51	CK2c5	custom - CC chemokine
38359	5.53	0.33	0.0000	0.0033	3.50	all_v2.0.415.C5	Homolog of Homo sapiens Spastic ataxia of Charlevoix-Saguenay; n=1; Takifugu rubripes|Rep: Homolog of Homo sapiens Spastic ataxia of Charlevoix-Saguenay -

							Takifugu rubripes
44910	7.56	0.21	0.0000	0.0016	3.48	all_v2.0.3619.C2	Ifi-6-16 multi-domain protein
47292	3.91	0.46	0.0003	0.0157	3.45	all_v2.0.5356.C4	Unassigned protein
37854	3.46	0.43	0.0009	0.0253	3.41	sb_gmnlsfic_0002b12.t7	Homolog of Carassius auratus Interferon-inducible protein Gig2.; n=4; Takifugu rubripes|Rep: Homolog of Carassius auratus Interferon-inducible protein Gig2. - Takifugu rubripes
41313	3.83	0.40	0.0004	0.0171	3.38	all_v2.0.1009.C1	Probable ATP-dependent helicase LGP2 (EC 3.6.1.-) (Protein D11Lgp2 homolog).; n=1; Takifugu rubripes|Rep: Probable ATP-dependent helicase LGP2 (EC 3.6.1.-) (Protein D11Lgp2 homolog). - Takifugu rubripes
37456	3.94	0.40	0.0003	0.0152	3.36	all_v2.0.93.C1	Goose-type lysozyme 2; n=1; Gadus morhua|Rep: Goose-type lysozyme 2 - Gadus morhua (Atlantic cod)
46226	8.14	0.18	0.0000	0.0016	3.35	all_v2.0.415.C1	Serpentine_recp domain containing protein
44454	4.74	0.33	0.0001	0.0059	3.26	1AY614590_Gadus morhua CC chemokine type 1 mRNA, complete cds	custom - CC chemokine
44470	5.18	0.29	0.0000	0.0039	3.20	CK2c1d	custom - CC chemokine
44446	5.25	0.28	0.0000	0.0037	3.09	CK2c3	custom - CC chemokine
44464	3.97	0.40	0.0003	0.0148	3.04	CK10add	custom - CC chemokine
44324	5.95	0.23	0.0000	0.0027	3.04	all_v2.0.1123.C1	Atrophin-1 multi-domain protein
44584	3.88	0.35	0.0004	0.0164	3.00	DXH1a	custom - probable ATP-dependent RNA helicase DHX
41942	5.75	0.24	0.0000	0.0031	2.99	all_v2.0.8660.C1	RING finger protein 213; n=3; Homo sapiens|Rep: RING finger protein 213 - Homo sapiens (Human)
36381	6.40	0.22	0.0000	0.0023	2.98	all_v2.0.132.C9	CC chemokine type 2; n=1; Gadus morhua|Rep: CC chemokine type 2 - Gadus morhua (Atlantic cod)
42820	3.88	0.33	0.0004	0.0163	2.93	all_v2.0.1349.C1	Tubulin beta-2A chain; n=106; Eukaryota|Rep: Tubulin beta-2A chain - Homo sapiens (Human)
44463	4.17	0.32	0.0002	0.0117	2.85	CK2c4	custom - CC chemokine
45838	4.03	0.32	0.0003	0.0137	2.76	all_v2.0.13440.C1	non-directed
44472	5.18	0.24	0.0000	0.0039	2.72	2AY614592_Gadus morhua CC chemokine type 3 mRNA, complete cds	custom - CC chemokine
44586	3.73	0.32	0.0005	0.0186	2.71	DXH1b	custom - probable ATP-dependent RNA helicase DHX
43197	6.25	0.20	0.0000	0.0024	2.70	all_v2.0.3448.C4	VHSV-induced protein; n=1; Oncorhynchus mykiss|Rep: VHSV-induced protein - Oncorhynchus mykiss (Rainbow trout) (Salmo gairdneri)
47156	3.43	0.42	0.0009	0.0261	2.69	all_v2.0.617.C5	Unassigned protein
55231	5.53	0.22	0.0000	0.0033	2.65	all_v2.0.8766.C1	unclassified
48764	6.36	0.19	0.0000	0.0023	2.65	all_v2.0.3141.C3	Unassigned protein
47410	4.55	0.27	0.0001	0.0070	2.64	sb_gmnlsfic_	Unassigned protein

						0004h02.t7	
38670	5.02	0.27	0.0000	0.0044	2.64	all_v2.0.3891.C1	Isoform 2 of Q5RI56 ; n=2; Danio rerio|Rep: Isoform 2 of Q5RI56 - Danio rerio (Zebrafish) (Brachydanio rerio)
44450	3.59	0.35	0.0007	0.0221	2.62	CK2c6	custom - CC chemokine
38607	6.76	0.17	0.0000	0.0019	2.61	all_v2.0.7062.C1	Interferon stimulated gene 15; n=1; Gadus morhua|Rep: Interferon stimulated gene 15 - Gadus morhua (Atlantic cod)
39975	5.78	0.21	0.0000	0.0029	2.60	all_v2.0.4370.C3	Phospholipase D4; n=3; Murinae|Rep: Phospholipase D4 - Mus musculus (Mouse)
46199	4.61	0.30	0.0001	0.0066	2.59	all_v2.0.2412.C1	SACS; sacsin
44065	3.46	0.37	0.0009	0.0251	2.55	all_v2.0.15004.C1	Zgc:92249; n=2; Danio rerio|Rep: Zgc:92249 - Danio rerio (Zebrafish) (Brachydanio rerio)
44884	4.73	0.25	0.0001	0.0061	2.52	sb_gmapov_0019d14.pDNRF2	Hexb; hexosaminidase B [EC:3.2.1.52]; K01207 beta-N-acetylhexosaminidase
46202	4.47	0.28	0.0001	0.0078	2.50	sb_gmnbmd_0010a21.pDNRm13r	Sacs_predicted; sacsin (predicted)
47613	3.56	0.37	0.0007	0.0228	2.48	sb_gmnbbr_0013m15.pDNRm13r	Unassigned protein
43361	6.80	0.17	0.0000	0.0019	2.46	all_v2.0.2975.C2	Zgc:103420; n=3; Cyprinidae|Rep: Zgc:103420 - Danio rerio (Zebrafish) (Brachydanio rerio)
46576	6.47	0.18	0.0000	0.0022	2.46	all_v2.0.4.C213	Unassigned protein
37542	5.84	0.19	0.0000	0.0028	2.45	all_v2.0.8751.C1	Heat shock 10kD protein 1; n=3; Danio rerio|Rep: Heat shock 10kD protein 1 - Danio rerio (Zebrafish) (Brachydanio rerio)
44919	4.25	0.29	0.0002	0.0107	2.43	all_v2.0.17051.C1	Igsf2_predicted; immunoglobulin superfamily, member 2 (predicted); K06522 immunoglobulin superfamily, member 2/3/2008
39108	6.98	0.15	0.0000	0.0018	2.37	all_v2.0.3508.C1	Mitochondrial import inner membrane translocase subunit Tim13.; n=1; Takifugu rubripes|Rep: Mitochondrial import inner membrane translocase subunit Tim13. - Takifugu rubripes
36277	4.16	0.28	0.0002	0.0119	2.37	sb_gmapht_0009a19.pDNRF2	Calr protein; n=5; Danio rerio|Rep: Calr protein - Brachydanio rerio (Zebrafish) (Danio rerio)
40846	5.14	0.21	0.0000	0.0039	2.36	all_v2.0.4119.C1	PREDICTED: similar to Poly [ADP-ribose] polymerase 14 (PARP-14) (B aggressive lymphoma protein 2); n=1; Danio rerio|Rep: PREDICTED: similar to Poly [ADP-ribose] polymerase 14 (PARP-14) (B aggressive lymphoma protein 2) - Danio rerio
44371	3.68	0.36	0.0005	0.0199	2.35	all_v2.0.2805.C1	caspc; caspase c
44458	4.15	0.28	0.0002	0.0119	2.35	CK2c1	custom - CC chemokine
42545	4.57	0.23	0.0001	0.0069	2.35	all_v2.0.6888.C1	Thioredoxin; n=1; Ictalurus punctatus|Rep: Thioredoxin - Ictalurus punctatus (Channel catfish)
41407	6.04	0.16	0.0000	0.0026	2.29	all_v2.0.9147.C1	Proteasome activator complex subunit 1 (Proteasome activator 28-alpha subunit) (PA28alpha) (PA28a) (Activator of multicatalytic protease subunit 1) (11S regulator complex subunit alpha) (REG-alpha) (Interferon gamma up-regulated I-5111 protein) (IGUP I-51; n=1; Takifugu rubripes|Rep: Proteasome activator complex subunit 1 (Proteasome activator 28-alpha subunit) (PA28alpha) (PA28a) (Activator of multicatalytic protease subunit 1) (11S regulator complex subunit alpha) (REG-

							alpha) (Interferon gamma up-regulated I-5111 protein) (IGUP I-51 - Takifugu rubripes
44461	4.76	0.21	0.0001	0.0057	2.26	2AY614590_ Gadus morhua CC chemokine type 1 mRNA, complete cds	custom - CC chemokine
37299	5.65	0.18	0.0000	0.0031	2.25	all_v2.0.3679 .C1	Gadd45al protein; n=2; Danio rerio|Rep: Gadd45al protein - Danio rerio (Zebrafish) (Brachydanio rerio)
46563	4.30	0.24	0.0002	0.0104	2.23	sb_gmnlskic_ 0009n01.pDN Rm13r	Unassigned protein
45600	3.48	0.34	0.0008	0.0250	2.21	all_v2.0.5490 .C1.a	non-directed
41565	7.23	0.13	0.0000	0.0017	2.19	sb_gmnbhkic _0002c04.pD NRF2	Protein-tyrosine sulfotransferase 1; n=2; Danio rerio|Rep: Protein-tyrosine sulfotransferase 1 - Brachydanio rerio (Zebrafish) (Danio rerio)
36805	3.99	0.24	0.0003	0.0145	2.18	sb_gmnlpfic_ 0002o09.t7	DCMP deaminase; n=1; Danio rerio|Rep: DCMP deaminase - Danio rerio (Zebrafish) (Brachydanio rerio)
39326	4.18	0.23	0.0002	0.0117	2.14	all_v2.0.9595 .C1	NFX1-type zinc finger-containing protein 1.; n=1; Takifugu rubripes|Rep: NFX1-type zinc finger-containing protein 1 - Takifugu rubripes
49270	4.10	0.26	0.0002	0.0126	2.13	all_v2.0.1047 0.C1	unclassified
40543	3.54	0.29	0.0007	0.0232	2.13	all_v2.0.1220 .C1	PREDICTED: similar to hCG28999,; n=1; Monodelphis domestica|Rep: PREDICTED: similar to hCG28999, - Monodelphis domestica
44598	4.70	0.21	0.0001	0.0062	2.12	STAT14	custom - Signal transducer and activator of transcription 1
46276	5.20	0.18	0.0000	0.0038	2.11	sb_gmnbmd_ 0010h13.pDN Rm13r	SMC_N domain containing protein
36190	4.63	0.19	0.0001	0.0065	2.11	sb_gmapht_0 014i15.pDNR F2	Bloodthirsty; n=2; Notothenioidei|Rep: Bloodthirsty - Chaenocephalus aceratus (White crocodile fish)
54777	4.93	0.20	0.0000	0.0050	2.10	all_v2.0.3549 .C1	unclassified
40235	3.65	0.28	0.0006	0.0208	2.09	sb_gmnlkfic_ 0003l10.t7	PREDICTED: similar to ATPase, aminophospholipid transporter-like, Class I, type 8A, member 2; n=1; Monodelphis domestica|Rep: PREDICTED: similar to ATPase, aminophospholipid transporter-like, Class I, type 8A, member 2 - Monodelphis domestica
54933	4.71	0.19	0.0001	0.0062	2.08	all_v2.0.5297 .C2	unclassified
38355	4.79	0.20	0.0001	0.0056	2.07	all_v2.0.8243 .C2	Homolog of Homo sapiens Spastic ataxia of Charlevoix-Saguenay; n=1; Takifugu rubripes|Rep: Homolog of Homo sapiens Spastic ataxia of Charlevoix-Saguenay - Takifugu rubripes
36612	3.82	0.24	0.0004	0.0171	2.07	all_v2.0.1248 1.C1	Complement component C3; n=1; Paralichthys olivaceus|Rep: Complement component C3 - Paralichthys olivaceus (Japanese flounder)
45862	3.82	0.24	0.0004	0.0171	2.06	all_v2.0.1657 6.C1	non-directed
47248	3.79	0.25	0.0004	0.0176	2.06	all_v2.0.3503 .C1	Unassigned protein
38599	4.00	0.24	0.0003	0.0143	2.06	all_v2.0.1614 5.C1	Interferon regulatory factor 10; n=7; Danio rerio|Rep: Interferon regulatory factor 10 -

							Danio rerio (Zebrafish) (Brachydanio rerio)
41414	3.52	0.30	0.0008	0.0240	2.05	sb_gmnlsfic_0008p05.t7	Proteasome subunit alpha type 6-like protein; n=1; Xenopsylla cheopis|Rep: Proteasome subunit alpha type 6-like protein - Xenopsylla cheopis (oriental rat flea)
44655	6.33	0.14	0.0000	0.0023	2.05	all_v2.0.392.C1	DMP1 domain containing protein
36311	4.22	0.21	0.0002	0.0110	2.05	all_v2.0.12206.C1	Cardiac troponin T; n=14; Danio rerio|Rep: Cardiac troponin T - Danio rerio (Zebrafish) (Brachydanio rerio)
39974	6.90	0.12	0.0000	0.0019	2.05	all_v2.0.16283.C1	Phospholipase D4 (EC 3.1.4.4) (PLD 4) (Choline phosphatase 4) (Phosphatidylcholine-hydrolyzing phospholipase D4).; n=1; Takifugu rubripes|Rep: Phospholipase D4 (EC 3.1.4.4) (PLD 4) (Choline phosphatase 4) (Phosphatidylcholine-hydrolyzing phospholipase D4). - Takifugu rubripes
51332	5.46	0.16	0.0000	0.0034	2.04	all_v2.0.4629.C1	unclassified
46887	3.79	0.24	0.0004	0.0176	2.02	sb_gmnbpcic_0013o02.pDNRF2	Unassigned protein
43196	3.65	0.27	0.0006	0.0208	2.02	all_v2.0.3448.C3	VHSV-induced protein; n=1; Oncorhynchus mykiss|Rep: VHSV-induced protein - Oncorhynchus mykiss (Rainbow trout) (Salmo gairdneri)
37853	4.39	0.19	0.0001	0.0090	2.01	all_v2.0.7494.C1	Homolog of Carassius auratus Interferon-inducible protein Gig2.; n=4; Takifugu rubripes|Rep: Homolog of Carassius auratus Interferon-inducible protein Gig2. - Takifugu rubripes
41421	3.97	0.22	0.0003	0.0148	2.00	all_v2.0.1046.C1	Proteasome subunit alpha type-2; n=3; Xenopus|Rep: Proteasome subunit alpha type-2 - Xenopus laevis (African clawed frog)
39325	5.07	0.17	0.0000	0.0042	1.99	all_v2.0.9595.C2	NFX1-type zinc finger-containing protein 1.; n=1; Takifugu rubripes|Rep: NFX1-type zinc finger-containing protein 1 - Takifugu rubripes
46586	5.73	0.15	0.0000	0.0031	1.99	sb_gmnlem_0013j07.sp6	Unassigned protein
52110	3.42	0.24	0.0009	0.0262	1.98	all_v2.0.8876.C1	unclassified
39204	4.09	0.21	0.0002	0.0127	1.97	sb_gmnlmfta_0003l11.t7	Myomesin-2 (M-protein) (165 kDa titin-associated protein) (165 kDa connectin-associated protein).; n=1; Takifugu rubripes|Rep: Myomesin-2 (M-protein) (165 kDa titin-associated protein) (165 kDa connectin-associated protein). - Takifugu rubripes
55194	3.64	0.24	0.0006	0.0208	1.97	all_v2.0.9305.C1	unclassified
50215	3.89	0.21	0.0003	0.0161	1.96	all_v2.0.5974.C1	unclassified
53370	3.85	0.23	0.0004	0.0170	1.96	all_v2.0.11557.C2	unclassified
52791	3.79	0.22	0.0004	0.0176	1.95	all_v2.0.2818.C1	unclassified
42908	3.99	0.20	0.0003	0.0145	1.95	all_v2.0.2624.C2	Ubiquitin carrier protein; n=1; Tetrahymena thermophila SB210|Rep: Ubiquitin carrier protein - Tetrahymena thermophila SB210
41164	3.94	0.20	0.0003	0.0151	1.94	sb_gmnbspic_0006i05.pDNRm13r	PREDICTED: similar to tubby super-family protein isoform 2; n=2; Monodelphis domestica|Rep: PREDICTED: similar to tubby super-family protein isoform 2 - Monodelphis domestica
42748	5.15	0.16	0.0000	0.0039	1.94	all_v2.0.9596.C1	Transmembrane protein 7; n=1; Siniperca chuatsi|Rep: Transmembrane protein 7 -

							Siniperca chuatsi (Chinese perch)
36949	3.60	0.23	0.0006	0.0217	1.93	all_v2.0.3352.C2	Dual specificity protein phosphatase 21 (EC 3.1.3.48) (EC 3.1.3.16) (Low molecular weight dual specificity phosphatase 21).; n=1; Takifugu rubripes|Rep: Dual specificity protein phosphatase 21 (EC 3.1.3.48) (EC 3.1.3.16) (Low molecular weight dual specificity phosphatase 21). - Takifugu rubripes
38695	3.84	0.21	0.0004	0.0170	1.92	all_v2.0.7632.C1	K14 protein; n=1; Xenopus laevis|Rep: K14 protein - Xenopus laevis (African clawed frog)
47576	3.73	0.21	0.0005	0.0186	1.91	sb_gmnbspic_0005b23.pDNRF2	Unassigned protein
37762	3.47	0.25	0.0009	0.0250	1.91	sb_gmapov_0003k05.pDNRm13r	Homolog of Brachydanio rerio Phosphoribosyl pyrophosphate synthetase-associated protein 2.; n=1; Takifugu rubripes|Rep: Homolog of Brachydanio rerio Phosphoribosyl pyrophosphate synthetase-associated protein 2 - Takifugu rubripes
38024	3.64	0.23	0.0006	0.0208	1.90	sb_gmnlsfas_0004l17.t7	Homolog of Homo sapiens Chloride intracellular channel protein 4; n=1; Takifugu rubripes|Rep: Homolog of Homo sapiens Chloride intracellular channel protein 4 - Takifugu rubripes
43007	3.65	0.23	0.0006	0.0208	1.90	sb_gmapov_0013f18.pDNRF2	UPI000065EC34 related cluster; n=1; Takifugu rubripes|Rep: UPI000065EC34 UniRef100 entry - Takifugu rubripes
44546	5.07	0.15	0.0000	0.0042	1.86	IRF1OK4	custom - Interferon regulatory factor 10K
36388	3.75	0.21	0.0005	0.0182	1.86	sb_gmnblits_0034k09.pDNRF2	CCAAT/enhancer binding protein alpha; n=1; Oncorhynchus mykiss|Rep: CCAAT/enhancer binding protein alpha - Oncorhynchus mykiss (Rainbow trout) (Salmo gairdneri)
41423	3.69	0.20	0.0005	0.0197	1.86	all_v2.0.10513.C1	Proteasome subunit alpha type-6; n=12; Tetrapoda|Rep: Proteasome subunit alpha type-6 - Mus musculus (Mouse)
48995	4.85	0.15	0.0001	0.0053	1.85	sb_gmnlla_0001p14.t7	Unassigned protein
45719	3.47	0.22	0.0009	0.0250	1.84	all_v2.0.15866.C1.a	non-directed
51453	3.55	0.22	0.0007	0.0228	1.83	all_v2.0.1144.C1	unclassified
52606	3.70	0.22	0.0005	0.0194	1.82	all_v2.0.5996.C2	unclassified
43746	3.59	0.23	0.0007	0.0221	1.81	all_v2.0.4016.C1	Zgc:158393; n=3; Danio rerio|Rep: Zgc:158393 - Danio rerio (Zebrafish) (Brachydanio rerio)
51914	6.24	0.11	0.0000	0.0024	1.81	all_v2.0.17634.C1	unclassified
37566	7.67	0.08	0.0000	0.0016	1.80	all_v2.0.2479.C1	Heat shock protein 90Ae.; n=1; Takifugu rubripes|Rep: Heat shock protein 90Ae. - Takifugu rubripes
41810	4.71	0.15	0.0001	0.0062	1.80	sb_gmnbhkic_0006h11.pDNRF2	Reverse transcriptase-like protein; n=1; Paralichthys olivaceus|Rep: Reverse transcriptase-like protein - Paralichthys olivaceus (Japanese flounder)
40236	3.95	0.18	0.0003	0.0150	1.79	sb_gmapov_0016j14.pDNRF2	PREDICTED: similar to ATPase, H+ transporting, lysosomal V0 subunit a isoform 1 isoform 2; n=1; Canis familiaris|Rep: PREDICTED: similar to ATPase, H+ transporting, lysosomal V0 subunit a isoform 1 isoform 2 - Canis familiaris
51382	3.62	0.20	0.0006	0.0214	1.79	all_v2.0.5897.C3	unclassified
36870	4.24	0.17	0.0002	0.0108	1.79	all_v2.0.15702.C1	Disabled homolog 2-interacting protein (DAB2-interacting protein) (DAB2 interaction protein) (ASK-interacting protein

							1).; n=1; Takifugu rubripes|Rep: Disabled homolog 2-interacting protein (DAB2-interacting protein) (DAB2 interaction protein) (ASK-interacting protein 1). - Takifugu rubripes
54050	3.83	0.19	0.0004	0.0171	1.78	all_v2.0.9224.C1	unclassified
49763	3.65	0.20	0.0006	0.0208	1.78	all_v2.0.7999.C1	unclassified
36407	6.00	0.11	0.0000	0.0026	1.78	all_v2.0.1864.C7	CD9 protein; n=1; Oncorhynchus mykiss|Rep: CD9 protein - Oncorhynchus mykiss (Rainbow trout) (Salmo gairdneri)
43221	4.03	0.17	0.0003	0.0137	1.78	all_v2.0.10924.C1	Voltage-dependent N-type calcium channel subunit alpha-1B (Voltage- gated calcium channel subunit alpha Cav2.2) (Calcium channel, L type, alpha-1 polypeptide isoform 5) (Brain calcium channel III) (BIII).; n=1; Takifugu rubripes|Rep: Voltage-dependent N-type calcium channel subunit alpha-1B (Voltage- gated calcium channel subunit alpha Cav2.2) (Calcium channel, L type, alpha-1 polypeptide isoform 5) (Brain calcium channel III) (BIII). - Takifugu rubripes
40416	6.16	0.10	0.0000	0.0025	1.76	all_v2.0.2962.C1	PREDICTED: similar to dynein, light chain, LC8-type 2; n=1; Ornithorhynchus anatinus|Rep: PREDICTED: similar to dynein, light chain, LC8-type 2 - Ornithorhynchus anatinus
38353	3.93	0.19	0.0003	0.0152	1.76	all_v2.0.415.C2	Homolog of Homo sapiens Spastic ataxia of Charlevoix-Saguenay; n=1; Takifugu rubripes|Rep: Homolog of Homo sapiens Spastic ataxia of Charlevoix-Saguenay - Takifugu rubripes
53852	3.97	0.19	0.0003	0.0148	1.76	all_v2.0.6339.C1	unclassified
41014	3.64	0.18	0.0006	0.0208	1.75	all_v2.0.14158.C1	PREDICTED: similar to Snf2-related CBP activator protein; n=1; Mus musculus|Rep: PREDICTED: similar to Snf2-related CBP activator protein - Mus musculus
37073	3.96	0.17	0.0003	0.0148	1.74	sb_gmnbspic_0005b03.pDNRF2	EPSTI1 protein; n=3; Bos taurus|Rep: EPSTI1 protein - Bos taurus (Bovine)
54000	3.40	0.21	0.0010	0.0270	1.73	all_v2.0.7891.C1	unclassified
41829	5.24	0.12	0.0000	0.0037	1.72	sb_gmnblits_0018p08.pDNRF2	RFK protein; n=2; Bos taurus|Rep: RFK protein - Bos taurus (Bovine)
49538	3.57	0.18	0.0007	0.0223	1.72	all_v2.0.17441.C1	unclassified
36186	4.44	0.14	0.0001	0.0082	1.70	all_v2.0.661.C2	Bloodthirsty; n=2; Notothenioidei|Rep: Bloodthirsty - Chaenocephalus aceratus (White crocodile fish)
41418	3.76	0.17	0.0005	0.0182	1.69	all_v2.0.659.C1	Proteasome subunit alpha type; n=8; Euteleostomi|Rep: Proteasome subunit alpha type - Danio rerio (Zebrafish) (Brachydanio rerio)
46324	3.58	0.17	0.0007	0.0222	1.69	all_v2.0.16151.C1	TFIIS domain containing protein
35828	4.80	0.13	0.0001	0.0056	1.68	all_v2.0.10754.C1	AHA1, activator of heat shock protein ATPase homolog 1, like; n=2; Danio rerio|Rep: AHA1, activator of heat shock protein ATPase homolog 1, like - Danio rerio (Zebrafish) (Brachydanio rerio)
40980	3.85	0.17	0.0004	0.0170	1.68	sb_gmapov_0018g11.pDNRF2	PREDICTED: similar to scavenger receptor cysteine-rich protein precursor, partial; n=1; Strongylocentrotus purpuratus|Rep: PREDICTED: similar to scavenger receptor cysteine-rich protein precursor, partial - Strongylocentrotus purpuratus

48417	4.11	0.16	0.0002	0.0126	1.68	sb_gmnbpcic_0009d03.pDNRm13r	Unassigned protein
51421	4.31	0.14	0.0001	0.0102	1.68	all_v2.0.4626.C1	unclassified
38014	4.63	0.13	0.0001	0.0065	1.66	all_v2.0.16634.C1	Homolog of Homo sapiens Cathepsin K precursor; n=1; Takifugu rubripes|Rep: Homolog of Homo sapiens Cathepsin K precursor - Takifugu rubripes
36426	4.10	0.14	0.0002	0.0126	1.66	all_v2.0.8306.C2	Cell division cycle 42; n=12; Coelomata|Rep: Cell division cycle 42 - Danio rerio (Zebrafish) (Brachydanio rerio)
38436	4.25	0.14	0.0002	0.0107	1.66	sb_gmapov_0005h22.pDNRF2	Homolog of Homo sapiens Vacuolar sorting protein 54 long isoform; n=1; Takifugu rubripes|Rep: Homolog of Homo sapiens Vacuolar sorting protein 54 long isoform - Takifugu rubripes
36468	4.63	0.13	0.0001	0.0066	1.66	all_v2.0.495.C1	Chaperonin containing TCP1, subunit 5; n=4; Clupeocephala|Rep: Chaperonin containing TCP1, subunit 5 - Danio rerio (Zebrafish) (Brachydanio rerio)
41937	5.25	0.11	0.0000	0.0037	1.65	all_v2.0.7491.C2	RING finger protein 213.; n=1; Gallus gallus|Rep: RING finger protein 213 - Gallus gallus
43598	4.18	0.14	0.0002	0.0117	1.63	sb_gmnbmu_0001b07.pDNRm13r	Zgc:136346; n=7; Danio rerio|Rep: Zgc:136346 - Brachydanio rerio (Zebrafish) (Danio rerio)
44956	3.54	0.17	0.0007	0.0232	1.63	all_v2.0.18221.C1	KRAS, LOC418207; v-Ki-ras2 Kirsten rat sarcoma viral oncogene homolog; K07827 GTPase Kras
35923	5.86	0.09	0.0000	0.0027	1.62	all_v2.0.4393.C1	Annexin 4; n=3; Clupeocephala|Rep: Annexin 4 - Danio rerio (Zebrafish) (Brachydanio rerio)
38178	4.67	0.12	0.0001	0.0062	1.62	all_v2.0.14775.C1	Homolog of Homo sapiens Mucin 2 precursor; n=1; Takifugu rubripes|Rep: Homolog of Homo sapiens Mucin 2 precursor - Takifugu rubripes
51542	3.48	0.17	0.0008	0.0250	1.61	all_v2.0.6459.C1	unclassified
48638	3.50	0.18	0.0008	0.0243	1.61	all_v2.0.8600.C1	Unassigned protein
42017	4.22	0.13	0.0002	0.0110	1.60	sb_gmapov_0005b18.pDNRF2	Scavenger receptor class B type I; n=2; Salmonidae|Rep: Scavenger receptor class B type I - Salmo salar (Atlantic salmon)
35957	3.47	0.17	0.0009	0.0250	1.60	all_v2.0.15919.C1	Apolipoprotein A-IV4; n=3; Takifugu rubripes|Rep: Apolipoprotein A-IV4 - Fugu rubripes (Japanese pufferfish) (Takifugu rubripes)
38545	3.55	0.16	0.0007	0.0230	1.60	all_v2.0.2319.C2	IgD mRNA for immunoglobulin D; n=1; Takifugu rubripes|Rep: IgD mRNA for immunoglobulin D - Takifugu rubripes
36185	4.69	0.11	0.0001	0.0062	1.59	all_v2.0.661.C20	Bloodthirsty; n=2; Notothenioidei|Rep: Bloodthirsty - Chaenocephalus aceratus (White crocodile fish)
41404	3.87	0.14	0.0004	0.0165	1.59	all_v2.0.17673.C1	Proteasome (Prosome, macropain) subunit, beta type, 2; n=2; Otophysi|Rep: Proteasome (Prosome, macropain) subunit, beta type, 2 - Danio rerio (Zebrafish) (Brachydanio rerio)
46893	4.08	0.13	0.0002	0.0128	1.59	all_v2.0.7095.C1	Unassigned protein
40136	4.98	0.11	0.0000	0.0047	1.59	sb_gmapov_0004f15.pDNRF2	PREDICTED: Gallus gallus similar to Vasohibin (LOC423369), mRNA.; n=1; Gallus gallus|Rep: PREDICTED: Gallus gallus similar to Vasohibin (LOC423369), mRNA. - Gallus gallus
41109	3.46	0.17	0.0009	0.0253	1.59	sb_gmnbmd_0005c12.pDNRm13r	PREDICTED: similar to transposase; n=1; Danio rerio|Rep: PREDICTED: similar to transposase - Danio rerio
36757	4.50	0.12	0.0001	0.0074	1.58	all_v2.0.1015	Cytochrome c oxidase subunit VIa

						.C1	polypeptide 1; n=2; Danio rerio|Rep: Cytochrome c oxidase subunit VIa polypeptide 1 - Danio rerio (Zebrafish) (Brachydanio rerio)
42555	3.91	0.14	0.0003	0.0157	1.58	sb_gmlbgits_0003f16.sp6	Tho2; n=5; Danio rerio|Rep: Tho2 - Danio rerio (Zebrafish) (Brachydanio rerio)
36016	3.82	0.15	0.0004	0.0171	1.58	all_v2.0.7212.C1	ATP synthase coupling factor 6-like protein; n=1; Bufo gargarizans|Rep: ATP synthase coupling factor 6-like protein - Bufo bufo gargarizans (Asian toad)
41409	4.06	0.14	0.0002	0.0131	1.57	all_v2.0.379.C1	Proteasome activator subunit 2; n=3; Clupeocephala|Rep: Proteasome activator subunit 2 - Danio rerio (Zebrafish) (Brachydanio rerio)
36195	4.28	0.12	0.0002	0.0106	1.56	sb_gmnlbfic_0007a23.t7	Bloodthirsty; n=2; Notothenioidei|Rep: Bloodthirsty - Chaenocephalus aceratus (White crocodile fish)
55484	3.83	0.14	0.0004	0.0171	1.56	sb_gmapov_0019c03.pDNRm13r	ywhaqa; tyrosine 3-monooxygenase/tryptophan 5-monooxygenase activation protein, theta polypeptide a; K06630 tyrosine 3-monooxygenase/tryptophan 5-monooxygenase activation protein
42502	3.60	0.14	0.0006	0.0217	1.56	all_v2.0.17605.C1	T-complex protein 1, alpha subunit; n=6; Danio rerio|Rep: T-complex protein 1, alpha subunit - Danio rerio (Zebrafish) (Brachydanio rerio)
36813	3.58	0.15	0.0007	0.0222	1.55	sb_gmapte_0005h11.pDNRF2	DEAD (Asp-Glu-Ala-Asp) box polypeptide 23; n=9; Euteleostomi|Rep: DEAD (Asp-Glu-Ala-Asp) box polypeptide 23 - Danio rerio (Zebrafish) (Brachydanio rerio)
51062	4.81	0.10	0.0001	0.0055	1.55	all_v2.0.8508.C1	unclassified
50754	3.63	0.14	0.0006	0.0212	1.53	all_v2.0.5172.C1	unclassified
48810	4.08	0.12	0.0002	0.0128	1.53	all_v2.0.7149.C2	Unassigned protein
38910	4.02	0.12	0.0003	0.0138	1.53	all_v2.0.16286.C1	Male-specific protein; n=1; Sarotherodon galilaeus|Rep: Male-specific protein - Sarotherodon galilaeus (mango tilapia)
39798	3.49	0.15	0.0008	0.0247	1.53	all_v2.0.8253.C1	Opioid growth factor receptor; n=2; Salmo salar|Rep: Opioid growth factor receptor - Salmo salar (Atlantic salmon)
53714	3.41	0.15	0.0010	0.0265	1.53	all_v2.0.3972.C1	unclassified
40508	3.83	0.13	0.0004	0.0171	1.53	all_v2.0.5811.C1	PREDICTED: similar to glutathione S-transferase mu isoform 1; n=1; Danio rerio|Rep: PREDICTED: similar to glutathione S-transferase mu isoform 1 - Danio rerio
45010	5.94	0.07	0.0000	0.0027	1.52	all_v2.0.4885.C1	LOC100017761; similar to 90-kDa heat shock protein
50881	3.53	0.15	0.0007	0.0232	1.52	all_v2.0.324.C1	unclassified
39626	3.70	0.14	0.0005	0.0194	1.52	all_v2.0.4.CB27	Novel protein; n=2; Danio rerio|Rep: Novel protein - Danio rerio (Zebrafish) (Brachydanio rerio)
44865	3.82	0.13	0.0004	0.0171	1.52	all_v2.0.8362.C1	HERC5, LOC478474; hect domain and RLD 5
44008	3.48	0.14	0.0008	0.0249	1.52	all_v2.0.3117.C1	Zgc:86833; n=3; Clupeocephala|Rep: Zgc:86833 - Danio rerio (Zebrafish) (Brachydanio rerio)
51353	3.70	0.13	0.0005	0.0194	1.51	all_v2.0.10898.C1	unclassified
36922	3.54	0.13	0.0007	0.0232	1.51	all_v2.0.5225.C1	DnaJ-like subfamily A member 4; n=1; Paralichthys olivaceus|Rep: DnaJ-like subfamily A member 4 - Paralichthys olivaceus (Japanese flounder)
44759	5.42	0.08	0.0000	0.0034	1.50	sb_gmnblits_	Dynein_light domain containing protein

						0002l17.pDNRF2	
48472	3.61	0.14	0.0006	0.0217	1.49	all_v2.0.18800.C1	Unassigned protein
51340	3.69	0.12	0.0005	0.0197	1.48	all_v2.0.12995.C1	unclassified
44545	4.29	0.10	0.0002	0.0105	1.48	IRF1OK3	custom - Interferon regulatory factor 10K
42664	4.53	0.10	0.0001	0.0072	1.48	sb_gmnlskic_0011d03.pDNRm13r	Transcription factor; n=8; Clupeocephala|Rep: Transcription factor - Danio rerio (Zebrafish) (Brachydanio rerio)
46456	3.77	0.12	0.0004	0.0178	1.48	all_v2.0.4526.C3	Unassigned protein
49041	3.53	0.14	0.0008	0.0232	1.48	sb_gmnlla_0034i05.t7	Unassigned protein
38512	3.45	0.12	0.0009	0.0254	1.47	all_v2.0.8649.C1	HRAS-like suppressor 3; n=1; Perca flavescens|Rep: HRAS-like suppressor 3 - Perca flavescens (Yellow perch)
42922	4.60	0.09	0.0001	0.0068	1.47	sb_gmnlpras_0001f12.t7	Ubiquitin; n=6; Eukaryota|Rep: Ubiquitin - Tetrahymena pyriformis
49027	3.72	0.12	0.0005	0.0190	1.46	all_v2.0.17852.C1	Unassigned protein
48587	3.80	0.12	0.0004	0.0175	1.46	sb_gmlbgits_0001h03.t7	Unassigned protein
43079	4.61	0.09	0.0001	0.0066	1.46	sb_gmapht_0004g05.pDNRF2	UPI0000E4E275 related cluster; n=1; Danio rerio|Rep: UPI0000E4E275 UniRef100 entry - Danio rerio
40263	3.78	0.11	0.0004	0.0178	1.46	all_v2.0.661.C24	PREDICTED: similar to bloodthirsty; n=3; Danio rerio|Rep: PREDICTED: similar to bloodthirsty - Danio rerio
48510	3.81	0.11	0.0004	0.0173	1.46	sb_gmnlla_0035h19.t7	Unassigned protein
39825	3.98	0.10	0.0003	0.0147	1.45	sb_gmnlsras_0002k15.t7	Out at first protein homolog precursor; n=1; Gallus gallus|Rep: Out at first protein homolog precursor - Gallus gallus (Chicken)
52660	3.68	0.11	0.0005	0.0198	1.45	all_v2.0.14023.C1	unclassified
51485	3.50	0.13	0.0008	0.0243	1.44	all_v2.0.16707.C1	unclassified
45725	3.82	0.11	0.0004	0.0171	1.43	all_v2.0.15739.C1.a	non-directed
45026	3.70	0.11	0.0005	0.0194	1.43	sb_gmnlsras_0003k15.t7	LOC100030284; similar to asteroid homolog 1
41431	5.15	0.07	0.0000	0.0039	1.43	all_v2.0.15734.C1	Proteasome subunit beta type; n=5; Salmonidae|Rep: Proteasome subunit beta type - Salmo salar (Atlantic salmon)
43222	3.95	0.10	0.0003	0.0149	1.42	all_v2.0.11174.C1	Warm temperature acclimation-related 65 kDa protein; n=1; Dicentrarchus labrax|Rep: Warm temperature acclimation-related 65 kDa protein - Dicentrarchus labrax (European sea bass)
37573	3.91	0.10	0.0003	0.0157	1.42	sb_gmnlkfta_0003o06.t7	Heat shock protein HSP 90-alpha; n=8; Clupeocephala|Rep: Heat shock protein HSP 90-alpha - Danio rerio (Zebrafish) (Brachydanio rerio)
39321	4.02	0.10	0.0003	0.0138	1.42	all_v2.0.6027.C5	NFX1-type zinc finger-containing protein 1.; n=1; Takifugu rubripes|Rep: NFX1-type zinc finger-containing protein 1 - Takifugu rubripes
42817	4.25	0.08	0.0002	0.0107	1.38	sb_gmapov_0012m19.pDNRm13r	Tubulin alpha chain; n=36; Fungi/Metazoa group|Rep: Tubulin alpha chain - Octopus dofleini (Giant octopus)
44207	3.96	0.09	0.0003	0.0148	1.37	sb_gmnlem_0016n13.t7	[K] COG1958 Small nuclear ribonucleoprotein (snRNP) homolog
37574	3.93	0.09	0.0003	0.0152	1.37	all_v2.0.1961.C1	Heat shock protein HSP 90-beta; n=13; Euteleostomi|Rep: Heat shock protein HSP 90-beta - Danio rerio (Zebrafish) (Brachydanio rerio)
37745	3.49	0.10	0.0008	0.0245	1.36	sb_gmapov_0020h11.pDN	Homolog of Brachydanio rerio Novel ABC transporter similar to human multidrug-

						RF2	resistance proteins (MRP).; n=1; Takifugu rubripes|Rep: Homolog of Brachydanio rerio Novel ABC transporter similar to human multidrug-resistance proteins (MRP). - Takifugu rubripes
38765	3.56	0.10	0.0007	0.0226	1.36	sb_gmnbpcic_0003i03.pDNRm13r	Leucine rich repeat containing 50; n=3; Danio rerio|Rep: Leucine rich repeat containing 50 - Danio rerio (Zebrafish) (Brachydanio rerio)
44088	4.88	0.06	0.0001	0.0052	1.35	all_v2.0.5218.C1	Zgc:92655; n=1; Danio rerio|Rep: Zgc:92655 - Danio rerio (Zebrafish) (Brachydanio rerio)
39904	3.87	0.08	0.0004	0.0167	1.33	all_v2.0.6912.C1	Peptidyl-prolyl cis-trans isomerase; n=6; Danio rerio|Rep: Peptidyl-prolyl cis-trans isomerase - Danio rerio (Zebrafish) (Brachydanio rerio)
36584	4.40	0.06	0.0001	0.0087	1.33	all_v2.0.4715.C1	Cold-inducible RNA-binding protein (Glycine-rich RNA-binding protein CIRP) (A18 hnRNP).; n=1; Takifugu rubripes|Rep: Cold-inducible RNA-binding protein (Glycine-rich RNA-binding protein CIRP) (A18 hnRNP). - Takifugu rubripes
37707	3.50	0.08	0.0008	0.0243	1.33	sb_gmapov_0029c16.pDNRF2	Homolog of Brachydanio rerio Fem1c.; n=1; Takifugu rubripes|Rep: Homolog of Brachydanio rerio Fem1c. - Takifugu rubripes
36265	4.81	0.06	0.0001	0.0055	1.33	all_v2.0.234.C13	Calmodulin; n=24; Eukaryota|Rep: Calmodulin - Homo sapiens (Human)
45467	5.06	0.05	0.0000	0.0042	1.32	all_v2.0.1658.C1	Myosin_tail_1 multi-domain protein
42133	3.45	0.08	0.0009	0.0253	1.31	sb_gmapht_0014k02.pDNRF2	SET domain-containing protein 8; n=2; Xenopus tropicalis|Rep: SET domain-containing protein 8 - Xenopus tropicalis (Western clawed frog) (Silurana tropicalis)
36062	3.47	0.08	0.0009	0.0250	1.30	all_v2.0.6364.C1	ATP-binding cassette, sub-family F (GCN20), member 1; n=3; Clupeocephala|Rep: ATP-binding cassette, sub-family F (GCN20), member 1 - Danio rerio (Zebrafish) (Brachydanio rerio)
55532	4.14	0.06	0.0002	0.0121	1.30	all_v2.0.10346.C1	ZNFX1; zinc finger, NFX1-type containing 1
36019	3.47	0.08	0.0009	0.0250	1.30	all_v2.0.2622.C1	ATP synthase gamma chain; n=4; Cyprinidae|Rep: ATP synthase gamma chain - Danio rerio (Zebrafish) (Brachydanio rerio)
38293	3.69	0.07	0.0005	0.0197	1.28	all_v2.0.15532.C1	Homolog of Homo sapiens Proprotein convertase subtilisin/kexin type 5 precursor; n=1; Takifugu rubripes|Rep: Homolog of Homo sapiens Proprotein convertase subtilisin/kexin type 5 precursor - Takifugu rubripes
45192	-5.72	0.07	0.0000	0.0031	0.68	all_v2.0.13771.C1	LOC570025; similar to potassium channel tetramerisation domain containing 1
39452	-5.51	0.15	0.0000	0.0033	0.51	sb_gmapht_0021i12.pDNRm13r	Novel protein similar to vertebrate ATPase, Ca++ transporting, cardiac muscle, slow twitch 2; n=3; Danio rerio|Rep: Novel protein similar to vertebrate ATPase, Ca++ transporting, cardiac muscle, slow twitch 2 - Brachydanio rerio (Zebrafish) (Danio rerio)
49079	-6.24	0.13	0.0000	0.0024	0.51	all_v2.0.8699.C1	unclassified

[a]Fold-changes are presented as outputted by siggenes and were calculated as pIC/PBS

Supplemental Table S6a. 29 genes differentially expressed between fish injected with PBS at 10°C and sampled at 6HPI compared to non-injected fish held at 10°C

Probe ID	d-value	Stdev	Raw p	q-value	Fold Change[a]	Sequence Name	Gene Name
49496	3.45	0.50	0.0001	0.0439	5.36	all_v2.0.17205.C1	unclassified
49256	3.62	0.75	0.0000	0.0439	5.27	all_v2.0.8761.C1	unclassified
43843	3.42	0.73	0.0001	0.0439	4.47	sb_gmnbpcic_0011c15.pDNRF2	Zgc:55941; n=3; Danio rerio|Rep: Zgc:55941 - Danio rerio (Zebrafish) (Brachydanio rerio)
39583	3.36	0.70	0.0001	0.0485	3.88	sb_gmnbhkas_0002m19.pDNRF2	Novel protein; n=2; Danio rerio|Rep: Novel protein - Brachydanio rerio (Zebrafish) (Danio rerio)
46438	3.51	0.34	0.0000	0.0439	2.60	all_v2.0.15803.C1	Unassigned protein
46031	3.06	0.35	0.0002	0.0611	2.35	sb_gmnbspic_0004i16.pDNRF2	PREDICTED: similar to CG3168-PA, isoform A [Tribolium castaneum]
38189	3.10	0.20	0.0002	0.0573	1.93	all_v2.0.9763.C1	Homolog of Homo sapiens N2B-Titin Isoform.; n=1; Takifugu rubripes|Rep: Homolog of Homo sapiens N2B-Titin Isoform. - Takifugu rubripes
49988	3.13	0.21	0.0002	0.0573	1.86	all_v2.0.9043.C1	unclassified
36701	3.69	0.14	0.0000	0.0439	1.79	all_v2.0.2115.C1	C-X-C chemokine receptor type 4 (CXC-R4) (CXCR-4) (Stromal cell- derived factor 1 receptor) (SDF-1 receptor) (Fusin) (Leukocyte-derived seven transmembrane domain receptor) (LESTR) (LCR1) (FB22) (NPYRL) (HM89) (CD184 antigen).; n=1; Takifugu rubripes|Rep: C-X-C chemokine receptor type 4 (CXC-R4) (CXCR-4) (Stromal cell- derived factor 1 receptor) (SDF-1 receptor) (Fusin) (Leukocyte-derived seven transmembrane domain receptor) (LESTR) (LCR1) (FB22) (NPYRL) (HM89) (CD184 antigen). - Takifugu rubripes
45703	3.05	0.18	0.0002	0.0611	1.77	all_v2.0.2158.C2	non-directed
43360	3.56	0.14	0.0000	0.0439	1.73	all_v2.0.2123.C1	Zgc:103414; n=2; Danio rerio|Rep: Zgc:103414 - Danio rerio (Zebrafish) (Brachydanio rerio)
45561	3.07	0.16	0.0002	0.0611	1.72	all_v2.0.206.C1	non-directed
55365	3.67	0.12	0.0000	0.0439	1.71	all_v2.0.16353.C1	unclassified
40998	3.21	0.15	0.0001	0.0552	1.66	sb_gmapht_0026m01.pDNRm13r	PREDICTED: similar to shugoshin-like 1 isoform A2; n=1; Danio rerio|Rep: PREDICTED: similar to shugoshin-like 1 isoform A2 - Danio rerio
47083	3.12	0.12	0.0002	0.0573	1.55	sb_gmapht_0017e09.pDNRF2	Unassigned protein
41731	3.11	0.08	0.0002	0.0573	1.42	sb_gmnblits_0001b02.pDNRF2	Renin binding protein; n=2; Danio rerio|Rep: Renin binding protein - Brachydanio rerio (Zebrafish) (Danio rerio)
37564	3.17	0.06	0.0001	0.0552	1.38	all_v2.0.16020.C1	Heat shock protein 90; n=13; Euteleostomi|Rep: Heat shock protein 90 - Scophthalmus maximus (Turbot)
38314	3.42	0.04	0.0001	0.0439	1.34	sb_gmapov_0007l16.pDNRF2	Homolog of Homo sapiens Ribonuclease P 40kDa subunit; n=1; Takifugu rubripes|Rep: Homolog of Homo sapiens Ribonuclease P 40kDa subunit - Takifugu rubripes
46638	-3.17	0.13	0.0001	0.0552	0.63	sb_gmnlla_0011k07.t7	Unassigned protein
37831	-3.19	0.14	0.0001	0.0552	0.60	sb_gmnlsfas_0002e11.t7	Homolog of Brachydanio rerio Transmembrane 9 superfamily member 2.; n=1; Takifugu rubripes|Rep: Homolog of

41726	-3.30	0.15	0.0001	0.0495	0.59	all_v2.0.13271.C1	Brachydanio rerio Transmembrane 9 superfamily member 2 - Takifugu rubripes Regulator of G-protein signaling 7-binding protein (R7 family-binding protein).; n=1; Takifugu rubripes|Rep: Regulator of G-protein signaling 7-binding protein (R7 family-binding protein). - Takifugu rubripes
45582	-3.18	0.16	0.0001	0.0552	0.58	all_v2.0.3806.C1.a	non-directed
55515	-3.71	0.16	0.0000	0.0439	0.53	sb_gmnbspic_0008o09.pDNRm13r	zgc:63779 [EC:1.-.-.-]; K05544 tRNA-dihydrouridine synthase 3
46595	-3.63	0.18	0.0000	0.0439	0.51	sb_gmapht_0011j22.pDNRF2	Unassigned protein
39382	-3.68	0.18	0.0000	0.0439	0.50	all_v2.0.16966.C1	Novel NACHT domain containing protein; n=1; Danio rerio|Rep: Novel NACHT domain containing protein - Danio rerio (Zebrafish) (Brachydanio rerio)
51763	-3.32	0.30	0.0001	0.0495	0.43	all_v2.0.2660.C1	unclassified
42925	-3.20	0.39	0.0001	0.0552	0.39	all_v2.0.9463.C1	Ubiquitin-conjugating enzyme E2 D2 (EC 6.3.2.19) (Ubiquitin-protein ligase D2) (Ubiquitin carrier protein D2) (Ubiquitin-conjugating enzyme E2-17 kDa 2) (E2(17)KB 2); n=32; Euteleostomi|Rep: Ubiquitin-conjugating enzyme E2 D2 (EC 6.3.2.19) (Ubiquitin-protein ligase D2) (Ubiquitin carrier protein D2) (Ubiquitin-conjugating enzyme E2-17 kDa 2) (E2(17)KB 2) - Homo sapiens (Human)
38901	-3.47	0.32	0.0001	0.0439	0.38	all_v2.0.3514.C1	MAK16-like protein RBM13; n=2; Danio rerio|Rep: MAK16-like protein RBM13 - Danio rerio (Zebrafish) (Brachydanio rerio)
38791	-3.65	0.51	0.0000	0.0439	0.21	all_v2.0.8167.C1	LIM and senescent cell antigen-like-containing domain protein 1; n=22; Euteleostomi|Rep: LIM and senescent cell antigen-like-containing domain protein 1 - Homo sapiens (Human)

[a]Fold-changes are presented as outputted by siggenes and were calculated as pIC/PBS

Supplemental Table S6b. 40 genes differentially expressed between fish injected with PBS at 16°C and sampled at 6HPI compared to non-injected fish held at 16°C

Probe ID	d-value	Stdev	Raw p	q-value	Fold Change[a]	Sequence Name	Gene Name
38707	4.07	0.18	0.0000	0.0264	2.10	all_v2.0.382.C2	Keratin, type I cytoskeletal 18; n=1; Oncorhynchus mykiss|Rep: Keratin, type I cytoskeletal 18 - Oncorhynchus mykiss (Rainbow trout) (Salmo gairdneri)
36479	4.68	0.11	0.0000	0.0189	1.92	all_v2.0.3017.C1	chemokine (C-C motif) ligand 12; n=2; Rattus norvegicus|Rep: chemokine (C-C motif) ligand 12 - Rattus norvegicus
53922	-3.42	0.08	0.0001	0.0318	0.66	all_v2.0.9849.C1	unclassified
35834	-3.76	0.07	0.0000	0.0318	0.66	all_v2.0.14969.C1	Alanine-glyoxylate aminotransferase; n=3; Danio rerio|Rep: Alanine-glyoxylate aminotransferase - Danio rerio (Zebrafish) (Brachydanio rerio)
51062	-3.22	0.11	0.0001	0.0396	0.65	all_v2.0.8508.C1	unclassified
35673	-3.67	0.09	0.0000	0.0318	0.63	sb_gmnbmd_0015b19.pDNRm13r	60S ribosomal protein L18a; n=7; Clupeocephala|Rep: 60S ribosomal protein L18a - Brachydanio rerio (Zebrafish) (Danio rerio)
47408	-3.53	0.10	0.0001	0.0318	0.63	sb_gmnbhkic_0012e23.pDNRm13r	Unassigned protein
47382	-3.14	0.13	0.0002	0.0397	0.63	sb_gmapht_0030g24.pDNRF2	Unassigned protein
40857	-3.70	0.10	0.0000	0.0318	0.61	sb_gmapov_0025b10.pDNRF2	PREDICTED: similar to potassium voltage-gated channel KQT-like protein 4, partial; n=1; Danio rerio|Rep: PREDICTED: similar to potassium voltage-gated channel KQT-like protein 4, partial - Danio rerio
53832	-4.06	0.09	0.0000	0.0264	0.61	all_v2.0.7066.C3	unclassified
55312	-3.47	0.13	0.0001	0.0318	0.60	all_v2.0.9193.C1	unclassified
35728	-3.14	0.16	0.0002	0.0397	0.59	all_v2.0.4284.C1	Abhydrolase domain-containing protein 2-B; n=2; Danio rerio|Rep: Abhydrolase domain-containing protein 2-B - Danio rerio (Zebrafish) (Brachydanio rerio)
35697	-3.22	0.16	0.0001	0.0396	0.58	all_v2.0.3652.C1	60S ribosomal protein L35a; n=2; Otophysi|Rep: 60S ribosomal protein L35a - Ictalurus punctatus (Channel catfish)
36948	-3.45	0.15	0.0001	0.0318	0.57	all_v2.0.956.C1	Dual specificity protein phosphatase 2 (EC 3.1.3.48) (EC 3.1.3.16) (Dual specificity protein phosphatase PAC-1).; n=1; Takifugu rubripes|Rep: Dual specificity protein phosphatase 2 (EC 3.1.3.48) (EC 3.1.3.16) (Dual specificity protein phosphatase PAC-1). - Takifugu rubripes
48898	-3.13	0.18	0.0002	0.0397	0.55	all_v2.0.2088.C2	Unassigned protein
53553	-3.61	0.16	0.0001	0.0318	0.55	all_v2.0.17041.C1	unclassified
37289	-3.47	0.17	0.0001	0.0318	0.55	sb_gmnbhkas_0019c20.pDNRF2	G protein-coupled receptor 155; n=1; Takifugu rubripes|Rep: G protein-coupled receptor 155 - Fugu rubripes (Japanese pufferfish) (Takifugu rubripes)
38629	-3.36	0.22	0.0001	0.0318	0.51	sb_gmnlskic_0014o15.pDNRm13r	Interleukin-1 receptor-associated kinase 4 (EC 2.7.11.1) (IRAK-4) (NY- REN-64 antigen).; n=1; Takifugu rubripes|Rep: Interleukin-1 receptor-associated kinase 4 (EC 2.7.11.1) (IRAK-4) (NY- REN-64 antigen). - Takifugu rubripes
42231	-3.22	0.23	0.0002	0.0396	0.50	all_v2.0.3901.C1	Skeletal muscle myosin heavy chain light meromyosin; n=10; Clupeocephala|Rep:

							Skeletal muscle myosin heavy chain light meromyosin - Clupea harengus (Atlantic herring)
48640	-3.14	0.24	0.0002	0.0397	0.50	sb_gmnlla_0018p21.t7	Unassigned protein
46452	-3.41	0.21	0.0001	0.0318	0.49	all_v2.0.3031.C1	Unassigned protein
40061	-3.35	0.21	0.0001	0.0318	0.48	sb_gmnbbrts_0007b11.pDNRF2	Poly(A) polymerase gamma; n=4; Cyprinidae|Rep: Poly(A) polymerase gamma - Brachydanio rerio (Zebrafish) (Danio rerio)
37768	-3.37	0.26	0.0001	0.0318	0.46	all_v2.0.7228.C1	Homolog of Brachydanio rerio Prosaposin.; n=1; Takifugu rubripes|Rep: Homolog of Brachydanio rerio Prosaposin. - Takifugu rubripes
39382	-3.39	0.27	0.0001	0.0318	0.45	all_v2.0.16966.C1	Novel NACHT domain containing protein; n=1; Danio rerio|Rep: Novel NACHT domain containing protein - Danio rerio (Zebrafish) (Brachydanio rerio)
37831	-3.36	0.28	0.0001	0.0318	0.44	sb_gmnlsfas_0002e11.t7	Homolog of Brachydanio rerio Transmembrane 9 superfamily member 2.; n=1; Takifugu rubripes|Rep: Homolog of Brachydanio rerio Transmembrane 9 superfamily member 2 - Takifugu rubripes
46595	-3.69	0.21	0.0000	0.0318	0.44	sb_gmapht_0011j22.pDNRF2	Unassigned protein
36177	-3.13	0.33	0.0002	0.0397	0.43	sb_gmnlem_0010g13.t7	Birc4 protein; n=7; Danio rerio|Rep: Birc4 protein - Danio rerio (Zebrafish) (Brachydanio rerio)
38545	-3.14	0.33	0.0002	0.0397	0.43	all_v2.0.2319.C2	IgD mRNA for immunoglobulin D; n=1; Takifugu rubripes|Rep: IgD mRNA for immunoglobulin D - Takifugu rubripes
42166	-4.33	0.21	0.0000	0.0223	0.42	all_v2.0.7654.C1	Si:dkey-103i16.1 protein; n=3; Danio rerio|Rep: Si:dkey-103i16.1 protein - Danio rerio (Zebrafish) (Brachydanio rerio)
46707	-3.53	0.30	0.0001	0.0318	0.42	sb_gmnbhkic_0016o08.pDNRm13r	Unassigned protein
41730	-4.76	0.22	0.0000	0.0189	0.36	sb_gmapov_0017j04.pDNRm13r	Regulatory-associated protein of mTOR (Raptor) (P150 target of rapamycin (TOR)-scaffold protein); n=24; Tetrapoda|Rep: Regulatory-associated protein of mTOR (Raptor) (P150 target of rapamycin (TOR)-scaffold protein) - Homo sapiens (Human)
47560	-3.49	0.37	0.0001	0.0318	0.35	sb_gmapte_0001n01.pDNRm13r	Unassigned protein
38794	-3.91	0.31	0.0000	0.0310	0.34	all_v2.0.10412.C1	LIM homeobox 8; n=4; Euteleostomi|Rep: LIM homeobox 8 - Danio rerio (Zebrafish) (Brachydanio rerio)
39112	-3.16	0.55	0.0002	0.0397	0.28	all_v2.0.1343.C1	Mitochondrial import inner membrane translocase subunit Tim23; n=3; Otophysi|Rep: Mitochondrial import inner membrane translocase subunit Tim23 - Danio rerio (Zebrafish) (Brachydanio rerio)
48085	-3.64	0.54	0.0000	0.0318	0.26	sb_gmnlla_0019f11.t7	Unassigned protein
38791	-3.11	0.58	0.0002	0.0401	0.23	all_v2.0.8167.C1	LIM and senescent cell antigen-like-containing domain protein 1; n=22; Euteleostomi|Rep: LIM and senescent cell antigen-like-containing domain protein 1 - Homo sapiens (Human)

[a]Fold-changes are presented as outputted by siggenes and were calculated as pIC/PBS

Supplemental Table S6c. 18 genes differentially expressed between fish injected with PBS at 10°C and sampled at 24HPI compared to non-injected fish held at 10°C

Probe ID	d-value	Stdev	Raw p	q-value	Fold Change[a]	Sequence Name	Gene Name
50050	4.42	0.24	0.0000	0.0367	2.54	all_v2.0.1891.C1	unclassified
39412	3.75	0.25	0.0001	0.0727	2.25	all_v2.0.3466.C1	Novel protein similar to human and mouse origin recognition complex, subunit 6 homolog-like; n=1; Danio rerio|Rep: Novel protein similar to human and mouse origin recognition complex, subunit 6 homolog-like - Danio rerio (Zebrafish) (Brachydanio rerio)
48489	5.28	0.14	0.0000	0.0129	2.06	sb_gmnlem_0003i16.sp6	Unassigned protein
35661	3.38	0.20	0.0002	0.0862	1.87	all_v2.0.10965.C2	5-aminolevulinate synthase, erythroid-specific, mitochondrial precursor (EC 2.3.1.37) (5-aminolevulinic acid synthase) (Delta- aminolevulinate synthase) (Delta-ALA synthetase) (ALAS-E).; n=1; Takifugu rubripes|Rep: 5-aminolevulinate synthase, erythroid-specific, mitochondrial precursor (EC 2.3.1.37) (5-aminolevulinic acid synthase) (Delta- aminolevulinate synthase) (Delta-ALA synthetase) (ALAS-E). - Takifugu rubripes
39388	3.65	0.15	0.0001	0.0727	1.69	all_v2.0.12658.C1	Novel protein containing a short chain dehydrogenase domain; n=3; Danio rerio|Rep: Novel protein containing a short chain dehydrogenase domain - Danio rerio (Zebrafish) (Brachydanio rerio)
44195	4.22	0.11	0.0000	0.0449	1.66	sb_gmnlem_0025b10.t7	[E] COG0421 Spermidine synthase
38355	3.99	0.12	0.0000	0.0631	1.62	all_v2.0.8243.C2	Homolog of Homo sapiens Spastic ataxia of Charlevoix-Saguenay; n=1; Takifugu rubripes|Rep: Homolog of Homo sapiens Spastic ataxia of Charlevoix-Saguenay - Takifugu rubripes
44726	4.63	0.07	0.0000	0.0356	1.51	all_v2.0.4995.C2	DUF1891 domain containing protein
49865	3.33	0.11	0.0002	0.0862	1.49	all_v2.0.190.C1	unclassified
49958	3.40	0.11	0.0002	0.0862	1.49	all_v2.0.4323.C1	unclassified
41096	3.43	0.08	0.0001	0.0862	1.40	all_v2.0.1554.C7	PREDICTED: similar to transposase (putative); n=1; Danio rerio|Rep: PREDICTED: similar to transposase (putative) - Danio rerio
36247	3.90	0.06	0.0000	0.0631	1.38	all_v2.0.7703.C1	Calcium binding and coiled-coil domain 2; n=5; Danio rerio|Rep: Calcium binding and coiled-coil domain 2 - Danio rerio (Zebrafish) (Brachydanio rerio)
51110	-3.54	0.12	0.0001	0.0727	0.66	all_v2.0.11720.C1	unclassified
41491	-3.55	0.13	0.0001	0.0727	0.63	all_v2.0.871.C1	Protein mago nashi homolog 2; n=25; Coelomata|Rep: Protein mago nashi homolog 2 - Homo sapiens (Human)

46595	-3.85	0.14	0.0000	0.0631	0.58	sb_gmapht_0011j22.pDNRF2	Unassigned protein
39382	-3.54	0.19	0.0001	0.0727	0.54	all_v2.0.16966.C1	Novel NACHT domain containing protein; n=1; Danio rerio|Rep: Novel NACHT domain containing protein - Danio rerio (Zebrafish) (Brachydanio rerio)
42813	-3.56	0.20	0.0001	0.0727	0.51	all_v2.0.5869.C1	Tryptophan hydroxylase 2; n=4; Danio rerio|Rep: Tryptophan hydroxylase 2 - Danio rerio (Zebrafish) (Brachydanio rerio)
37525	-3.53	0.22	0.0001	0.0727	0.51	all_v2.0.3167.C1	H/ACA ribonucleoprotein complex subunit 3; n=1; Danio rerio|Rep: H/ACA ribonucleoprotein complex subunit 3 - Danio rerio (Zebrafish) (Brachydanio rerio)

[a]Fold-changes are presented as outputted by siggenes and were calculated as pIC/PBS

Supplemental Table S6d. 97 genes differentially expressed between fish injected with PBS at 16°C and sampled at 24HPI compared to non-injected fish held at 16°C

Probe ID	d-value	Stdev	Raw p	q-value	Fold Change[a]	Sequence Name	Gene Name
44102	4.93	0.27	0.0000	0.0135	2.93	all_v2.0.6017.C3	Zgc:92811; n=1; Danio rerio|Rep: Zgc:92811 - Danio rerio (Zebrafish) (Brachydanio rerio)
41271	5.13	0.20	0.0000	0.0119	2.36	all_v2.0.16856.C1	PREDICTED: similar to Zinc finger, TTF-type; HAT dimerisation; Nucleic acid-binding, OB-fold; n=1; Nasonia vitripennis|Rep: PREDICTED: similar to Zinc finger, TTF-type; HAT dimerisation; Nucleic acid-binding, OB-fold - Nasonia vitripennis
49334	6.25	0.13	0.0000	0.0099	1.99	all_v2.0.10309.C2	unclassified
38154	4.67	0.15	0.0001	0.0161	1.78	all_v2.0.420.C1	Homolog of Homo sapiens Mammalian ependymin related protein-1 precursor; n=1; Takifugu rubripes|Rep: Homolog of Homo sapiens Mammalian ependymin related protein-1 precursor - Takifugu rubripes
50596	6.03	0.11	0.0000	0.0099	1.77	all_v2.0.13909.C1	unclassified
42753	4.54	0.11	0.0001	0.0174	1.56	all_v2.0.16847.C1	Transmembrane protein HTP-1; n=1; Danio rerio|Rep: Transmembrane protein HTP-1 - Danio rerio (Zebrafish) (Brachydanio rerio)
36183	4.47	0.11	0.0001	0.0180	1.56	sb_gmnlem_0014h23.t7	Blocked early in transport 1 homolog; n=1; Danio rerio|Rep: Blocked early in transport 1 homolog - Danio rerio (Zebrafish) (Brachydanio rerio)
48344	5.69	0.08	0.0000	0.0099	1.50	sb_gmnlkfas_0003n17.t7	Unassigned protein
40642	4.74	0.08	0.0001	0.0149	1.43	sb_gmapht_0014o12.pDNRF2	PREDICTED: similar to Matrin 3; n=2; Canis familiaris|Rep: PREDICTED: similar to Matrin 3 - Canis familiaris
46532	4.49	0.06	0.0001	0.0180	1.33	sb_gmnlem_0016p11.t7	Unassigned protein
50359	5.01	0.05	0.0000	0.0131	1.32	all_v2.0.10936.C1	unclassified
44647	4.56	0.06	0.0001	0.0172	1.30	all_v2.0.614.C1	dhx15; DEAH (Asp-Glu-Ala-His) box polypeptide 15
42052	-3.87	0.06	0.0003	0.0257	0.80	sb_gmapov_0016g07.pDNRF2	Selenoprotein W2a; n=3; Danio rerio|Rep: Selenoprotein W2a - Brachydanio rerio (Zebrafish) (Danio rerio)
35705	-3.76	0.06	0.0004	0.0269	0.79	all_v2.0.988.C1	60S ribosomal protein L7a; n=39; Euteleostomi|Rep: 60S ribosomal protein L7a - Fugu rubripes (Japanese pufferfish) (Takifugu rubripes)
39758	-3.89	0.07	0.0003	0.0256	0.77	all_v2.0.2758.C2	Nucleolar RNA helicase 2 (EC 3.6.1.-) (Nucleolar RNA helicase II) (Nucleolar RNA helicase Gu) (RH II/Gu) (Gu-alpha) (DEAD box protein 21).; n=1; Takifugu rubripes|Rep: Nucleolar RNA helicase 2 (EC 3.6.1.-) (Nucleolar RNA helicase II) (Nucleolar RNA helicase Gu) (RH II/Gu) (Gu-alpha) (DEAD box protein 21). - Takifugu rubripes

42683	-4.34	0.06	0.0001	0.0204	0.77	all_v2.0.14639.C1	Transketolase-like protein 2 (EC 2.2.1.1).; n=1; Takifugu rubripes|Rep: Transketolase-like protein 2 (EC 2.2.1.1). - Takifugu rubripes
42261	-5.47	0.04	0.0000	0.0099	0.77	all_v2.0.9140.C1	Smoothelin-C.; n=1; Gallus gallus|Rep: Smoothelin-C. - Gallus gallus
35674	-5.79	0.04	0.0000	0.0099	0.76	all_v2.0.1527.C1	60S ribosomal protein L18a; n=8; Clupeocephala|Rep: 60S ribosomal protein L18a - Ictalurus punctatus (Channel catfish)
43616	-3.93	0.08	0.0003	0.0242	0.75	sb_gmnbhkas_0015l23.pDNRF2	Zgc:136770; n=3; Danio rerio|Rep: Zgc:136770 - Danio rerio (Zebrafish) (Brachydanio rerio)
36265	-3.92	0.08	0.0003	0.0248	0.74	all_v2.0.234.C13	Calmodulin; n=24; Eukaryota|Rep: Calmodulin - Homo sapiens (Human)
36262	-3.97	0.08	0.0003	0.0242	0.74	all_v2.0.9474.C1	Calmodulin (CaM).; n=1; Canis lupus familiaris|Rep: Calmodulin (CaM). - Canis familiaris
41967	-5.25	0.06	0.0000	0.0117	0.73	all_v2.0.7960.C1	RNA-binding protein PNO1; n=1; Oryzias latipes|Rep: RNA-binding protein PNO1 - Oryzias latipes (Medaka fish) (Japanese ricefish)
41908	-3.77	0.10	0.0004	0.0268	0.72	all_v2.0.3157.C3	Ribosomal protein S2; n=8; Euteleostomi|Rep: Ribosomal protein S2 - Danio rerio (Zebrafish) (Brachydanio rerio)
35742	-3.95	0.09	0.0003	0.0242	0.72	all_v2.0.4656.C1	Acidic leucine-rich nuclear phosphoprotein 32 family member A; n=1; Danio rerio|Rep: Acidic leucine-rich nuclear phosphoprotein 32 family member A - Danio rerio (Zebrafish) (Brachydanio rerio)
43009	-3.90	0.09	0.0003	0.0253	0.72	sb_gmnbhkas_0007m19.pDNRF2	UPI000069E497 related cluster; n=1; Xenopus tropicalis|Rep: UPI000069E497 UniRef100 entry - Xenopus tropicalis
37705	-3.81	0.10	0.0004	0.0267	0.72	all_v2.0.10051.C1	Homolog of Brachydanio rerio Eukaryotic translation elongation factor 1 beta 2.; n=1; Takifugu rubripes|Rep: Homolog of Brachydanio rerio Eukaryotic translation elongation factor 1 beta 2 - Takifugu rubripes
52886	-3.78	0.10	0.0004	0.0267	0.72	all_v2.0.3166.C1	unclassified
36877	-4.12	0.09	0.0002	0.0218	0.71	all_v2.0.16110.C1	DNA (cytosine-5)-methyltransferase 1 (EC 2.1.1.37) (Dnmt1) (DNA methyltransferase HsaI) (DNA MTase HsaI) (MCMT) (M.HsaI).; n=1; Takifugu rubripes|Rep: DNA (cytosine-5)-methyltransferase 1 (EC 2.1.1.37) (Dnmt1) (DNA methyltransferase HsaI) (DNA MTase HsaI) (MCMT) (M.HsaI). - Takifugu rubripes
42610	-5.00	0.07	0.0000	0.0131	0.71	sb_gmnlkfic_0005i17.t7	Toll-like receptor 3-like protein; n=2; Oncorhynchus mykiss|Rep: Toll-like receptor 3-like protein - Oncorhynchus mykiss (Rainbow trout) (Salmo gairdneri)
35669	-3.95	0.10	0.0003	0.0242	0.70	all_v2.0.3432.C1	60S ribosomal protein L10; n=7; Theria|Rep: 60S ribosomal protein L10 - Homo sapiens (Human)
41974	-4.33	0.09	0.0001	0.020	0.70	sb_gmnbh	Rpl3-prov protein; n=6;

				4		kic_0017h23.pDNRm13r	Euteleostomi|Rep: Rpl3-prov protein - Xenopus laevis (African clawed frog)
35673	-4.23	0.10	0.0001	0.0204	0.69	sb_gmnbmd_0015b19.pDNRm13r	60S ribosomal protein L18a; n=7; Clupeocephala|Rep: 60S ribosomal protein L18a - Brachydanio rerio (Zebrafish) (Danio rerio)
46058	-4.47	0.10	0.0001	0.0180	0.69	sb_gmnblists_0018a21.pDNRm13r	PREDICTED: similar to ribosomal protein S2 [Homo sapiens] ref|XP_001126232.1| PREDICTED: similar to ribosomal protein S2 [Homo sapiens]
35704	-4.11	0.11	0.0002	0.0218	0.68	all_v2.0.988.C3	60S ribosomal protein L7a; n=11; Amniota|Rep: 60S ribosomal protein L7a - Gallus gallus (Chicken)
51414	-5.42	0.08	0.0000	0.0101	0.67	all_v2.0.2338.C1	unclassified
48328	-3.94	0.12	0.0003	0.0242	0.67	sb_gmnbgi_0004b17.pDNRm13r	Unassigned protein
36672	-4.86	0.09	0.0000	0.0135	0.67	all_v2.0.12537.C1	CREB-binding protein (EC 2.3.1.48).; n=4; Amniota|Rep: CREB-binding protein (EC 2.3.1.48). - Gallus gallus
44782	-4.71	0.10	0.0001	0.0151	0.67	all_v2.0.5759.C1	erythrocyte binding protein
45515	-3.94	0.12	0.0003	0.0242	0.66	all_v2.0.8577.C1	non-directed
41975	-4.22	0.11	0.0002	0.0204	0.66	sb_gmnbhkic_0017h23.pDNRF2	Rpl3-prov protein; n=6; Euteleostomi|Rep: Rpl3-prov protein - Xenopus laevis (African clawed frog)
48274	-4.44	0.11	0.0001	0.0189	0.66	sb_gmlbgits_0004a19.sp6	Unassigned protein
42980	-4.26	0.11	0.0001	0.0204	0.66	sb_gmapht_0033g21.pDNRm13r	UPI0000567126 related cluster; n=2; Danio rerio|Rep: UPI0000567126 UniRef100 entry - Danio rerio
39084	-4.01	0.12	0.0002	0.0242	0.66	all_v2.0.837.C1	Mid1-interacting protein 1 (Gastrulation-specific G12-like protein) (Mid1-interacting G12-like protein) (Protein STRAIT11499).; n=1; Takifugu rubripes|Rep: Mid1-interacting protein 1 (Gastrulation-specific G12-like protein) (Mid1-interacting G12-like protein) (Protein STRAIT11499). - Takifugu rubripes
52549	-3.80	0.14	0.0004	0.0267	0.65	all_v2.0.4100.C1	unclassified
40387	-4.25	0.12	0.0001	0.0204	0.65	sb_gmnllrta_0003a06.t7	PREDICTED: similar to cytoplasmic beta-actin; n=2; Homo/Pan/Gorilla group|Rep: PREDICTED: similar to cytoplasmic beta-actin - Homo sapiens
37007	-5.69	0.08	0.0000	0.0099	0.65	all_v2.0.234.C15	Elongation factor 1-alpha; n=2; Gadus morhua|Rep: Elongation factor 1-alpha - Gadus morhua (Atlantic cod)
50837	-4.52	0.11	0.0001	0.0174	0.65	all_v2.0.3307.C2	unclassified
49731	-3.81	0.13	0.0004	0.0267	0.65	all_v2.0.9379.C1	unclassified
48831	-4.01	0.13	0.0002	0.024	0.64	sb_gmnbh	Unassigned protein

				2		kic_0011n05.pDNRF2	
54908	-3.74	0.15	0.0004	0.0273	0.64	all_v2.0.2435.C2	unclassified
46270	-3.82	0.13	0.0004	0.0267	0.64	sb_gmapov_0011p13.pDNRF2	Slc24a4; solute carrier family 24 (sodium/potassium/calcium exchanger), member 4
40185	-4.06	0.13	0.0002	0.0230	0.64	Cod actin	PREDICTED: similar to Actin, cytoplasmic 2 (Gamma-actin); n=1; Rattus norvegicus|Rep: PREDICTED: similar to Actin, cytoplasmic 2 (Gamma-actin) - Rattus norvegicus
42435	-5.15	0.11	0.0000	0.0119	0.62	all_v2.0.4777.C1	SWI/SNF-related matrix-associated actin-dependent regulator of chromatin subfamily A containing DEAD/H box 1 (EC 3.6.1.-) (ATP- dependent helicase 1) (hHEL1).; n=2; Gallus gallus|Rep: SWI/SNF-related matrix-associated actin-dependent regulator of chromatin subfamily A containing DEAD/H box 1 (EC 3.6.1.-) (ATP- dependent helicase 1) (hHEL1). - Gallus gallus
41780	-3.75	0.15	0.0004	0.0269	0.62	sb_gmapte_0013b04.pDNRm13r	Reverse transcriptase; n=6; Fundulus heteroclitus|Rep: Reverse transcriptase - Fundulus heteroclitus (Killifish) (Mummichog)
43903	-3.94	0.15	0.0003	0.0242	0.62	all_v2.0.17667.C1	Zgc:64214; n=2; Danio rerio|Rep: Zgc:64214 - Danio rerio (Zebrafish) (Brachydanio rerio)
37745	-5.63	0.10	0.0000	0.0099	0.62	sb_gmapov_0020h11.pDNRF2	Homolog of Brachydanio rerio Novel ABC transporter similar to human multidrug-resistance proteins (MRP).; n=1; Takifugu rubripes|Rep: Homolog of Brachydanio rerio Novel ABC transporter similar to human multidrug-resistance proteins (MRP). - Takifugu rubripes
44008	-3.85	0.15	0.0003	0.0257	0.62	all_v2.0.3117.C1	Zgc:86833; n=3; Clupeocephala|Rep: Zgc:86833 - Danio rerio (Zebrafish) (Brachydanio rerio)
45466	-4.28	0.13	0.0001	0.0204	0.62	all_v2.0.9014.C1	Myosin_tail_1 multi-domain protein
49397	-3.76	0.15	0.0004	0.0268	0.61	all_v2.0.5648.C2	unclassified
40037	-4.24	0.14	0.0001	0.0204	0.60	all_v2.0.15772.C1	Pol-like protein; n=1; Danio rerio|Rep: Pol-like protein - Danio rerio (Zebrafish) (Brachydanio rerio)
53802	-3.73	0.17	0.0004	0.0273	0.60	all_v2.0.6636.C2	unclassified
51485	-3.96	0.16	0.0003	0.0242	0.60	all_v2.0.16707.C1	unclassified
42233	-4.08	0.15	0.0002	0.0225	0.60	sb_gmnbpcic_0005o11.pDNRF2	Skint 2 isoform b precursor; n=6; Mus musculus|Rep: Skint 2 isoform b precursor - Mus musculus (Mouse)
41068	-3.88	0.17	0.0003	0.0256	0.60	all_v2.0.1797.C1	PREDICTED: similar to thymosin, beta 4; n=1; Canis lupus familiaris|Rep: PREDICTED: similar to thymosin, beta 4 - Canis familiaris
53602	-4.24	0.16	0.0001	0.0204	0.59	all_v2.0.1269.C1	unclassified

43495	-3.97	0.16	0.0003	0.0242	0.59	sb_gmnbhkic_0020c19.pDNRF2	Zgc:112166; n=4; Danio rerio|Rep: Zgc:112166 - Danio rerio (Zebrafish) (Brachydanio rerio)
41357	-3.96	0.17	0.0003	0.0242	0.59	all_v2.0.2785.C1	Probable signal peptidase complex subunit 2; n=2; Danio rerio|Rep: Probable signal peptidase complex subunit 2 - Danio rerio (Zebrafish) (Brachydanio rerio)
42804	-3.97	0.17	0.0003	0.0242	0.58	all_v2.0.9458.C1	Troponin 1-like protein; n=1; Sparus aurata|Rep: Troponin 1-like protein - Sparus aurata (Gilthead sea bream)
38737	-3.99	0.16	0.0002	0.0242	0.58	all_v2.0.4631.C1	Kynurenine/alpha-aminoadipate aminotransferase mitochondrial precursor (KAT/AadAT) (Kynurenine--oxoglutarate transaminase II) (EC 2.6.1.7) (Kynurenine aminotransferase II) (Kynurenine--oxoglutarate aminotransferase II) (2-aminoadipate transaminase) (EC 2.; n=2; Danio rerio|Rep: Kynurenine/alpha-aminoadipate aminotransferase mitochondrial precursor (KAT/AadAT) (Kynurenine--oxoglutarate transaminase II) (EC 2.6.1.7) (Kynurenine aminotransferase II) (Kynurenine--oxoglutarate aminotransferase II) (2-aminoadipate transaminase) (EC 2 - Danio rerio
46550	-3.79	0.17	0.0004	0.0267	0.58	sb_gmnbgi_0003o02.pDNRm13r	Unassigned protein
36879	-5.15	0.13	0.0000	0.0119	0.57	sb_gmnbhkas_0001i05.pDNRm13r	DNA (Cytosine-5)-methyltransferase; n=8; Danio rerio|Rep: DNA (Cytosine-5)-methyltransferase - Brachydanio rerio (Zebrafish) (Danio rerio)
53580	-4.40	0.17	0.0001	0.0196	0.56	all_v2.0.15396.C2	unclassified
42531	-5.58	0.12	0.0000	0.0099	0.56	sb_gmnbpcic_0010g10.pDNRm13r	Thiamine transporter 2 (ThTr-2) (ThTr2) (Solute carrier family 19 member 3).; n=1; Takifugu rubripes|Rep: Thiamine transporter 2 (ThTr-2) (ThTr2) (Solute carrier family 19 member 3). - Takifugu rubripes
40754	-4.78	0.16	0.0001	0.0145	0.54	all_v2.0.16046.C1	PREDICTED: similar to opioid receptor-like protein Zf-orl; n=1; Monodelphis domestica|Rep: PREDICTED: similar to opioid receptor-like protein Zf-orl - Monodelphis domestica
36813	-4.21	0.18	0.0002	0.0207	0.54	sb_gmapte_0005h11.pDNRF2	DEAD (Asp-Glu-Ala-Asp) box polypeptide 23; n=9; Euteleostomi|Rep: DEAD (Asp-Glu-Ala-Asp) box polypeptide 23 - Danio rerio (Zebrafish) (Brachydanio rerio)
39044	-4.73	0.16	0.0001	0.0150	0.53	all_v2.0.1284.C13	MHC class I antigene; n=1; Salmo salar|Rep: MHC class I antigene - Salmo salar (Atlantic salmon)
43544	-5.13	0.16	0.0000	0.0119	0.52	all_v2.0.8240.C1	Zgc:113227; n=2; Danio rerio|Rep: Zgc:113227 - Danio rerio (Zebrafish) (Brachydanio rerio)
45721	-5.08	0.16	0.0000	0.012	0.52	all_v2.0.1	non-directed

				8		1198.C1.a	
45419	-4.57	0.17	0.0001	0.0172	0.52	sb_gmapov_0021e20.pDNRF2	MGC52541; similar to nucleoporin 214kDa
43930	-4.07	0.22	0.0002	0.0228	0.51	all_v2.0.826.C1	Zgc:73143; n=2; Danio rerio|Rep: Zgc:73143 - Danio rerio (Zebrafish) (Brachydanio rerio)
39391	-4.33	0.21	0.0001	0.0204	0.51	sb_gmnbhkic_0003n12.pDNRF2	Novel protein containing an Acyl-CoA dehydrogenase, N-terminal and C-terminal domain; n=2; Danio rerio|Rep: Novel protein containing an Acyl-CoA dehydrogenase, N-terminal and C-terminal domain - Brachydanio rerio (Zebrafish) (Danio rerio)
41106	-7.42	0.10	0.0000	0.0035	0.51	sb_gmnbpcic_0004a22.pDNRF2	PREDICTED: similar to transposase; n=1; Danio rerio|Rep: PREDICTED: similar to transposase - Danio rerio
39253	-4.05	0.23	0.0002	0.0230	0.50	all_v2.0.6799.C1	NADH dehydrogenase [ubiquinone] 1 alpha subcomplex subunit 1; n=4; Murinae|Rep: NADH dehydrogenase [ubiquinone] 1 alpha subcomplex subunit 1 - Mus musculus (Mouse)
54733	-4.83	0.20	0.0000	0.0138	0.48	all_v2.0.13534.C1	unclassified
37456	-3.85	0.27	0.0003	0.0257	0.47	all_v2.0.93.C1	Goose-type lysozyme 2; n=1; Gadus morhua|Rep: Goose-type lysozyme 2 - Gadus morhua (Atlantic cod)
36388	-5.75	0.17	0.0000	0.0099	0.47	sb_gmnblits_0034k09.pDNRF2	CCAAT/enhancer binding protein alpha; n=1; Oncorhynchus mykiss|Rep: CCAAT/enhancer binding protein alpha - Oncorhynchus mykiss (Rainbow trout) (Salmo gairdneri)
48182	-4.32	0.24	0.0001	0.0204	0.46	sb_gmnlla_0020p21.t7	Unassigned protein
52342	-3.71	0.28	0.0004	0.0274	0.46	all_v2.0.878.C1	unclassified
44918	-4.32	0.23	0.0001	0.0204	0.45	all_v2.0.5891.C6	IGHG1; similar to Ig gamma-1 chain C region
36870	-4.58	0.23	0.0001	0.0172	0.44	all_v2.0.15702.C1	Disabled homolog 2-interacting protein (DAB2-interacting protein) (DAB2 interaction protein) (ASK-interacting protein 1).; n=1; Takifugu rubripes|Rep: Disabled homolog 2-interacting protein (DAB2-interacting protein) (DAB2 interaction protein) (ASK-interacting protein 1). - Takifugu rubripes
54419	-4.65	0.28	0.0001	0.0161	0.39	all_v2.0.4355.C1	unclassified
52981	-3.79	0.40	0.0004	0.0267	0.36	all_v2.0.10153.C1	unclassified
46563	-4.88	0.31	0.0000	0.0135	0.31	sb_gmnlskic_0009n01.pDNRm13r	Unassigned protein
55182	-7.65	0.21	0.0000	0.0035	0.27	all_v2.0.11630.C1	unclassified
43331	-5.49	0.31	0.0000	0.0099	0.27	sb_gmnbspic_0015g05.pDNR	Zgc:101729; n=3; Danio rerio|Rep: Zgc:101729 - Danio rerio (Zebrafish) (Brachydanio rerio)

						F2	
40869	-3.88	0.51	0.0003	0.0256	0.20	sb_gmnbspic_0014p07.pDNRF2	PREDICTED: similar to Pre-mRNA processing splicing factor 8 (Splicing factor Prp8) (PRP8 homolog) (220 kDa U5 snRNP-specific protein) (p220) isoform 13; n=4; Deuterostomia|Rep: PREDICTED: similar to Pre-mRNA processing splicing factor 8 (Splicing factor Prp8) (PRP8 homolog) (220 kDa U5 snRNP-specific protein) (p220) isoform 13 - Canis familiaris
35888	-4.88	0.44	0.0000	0.0135	0.18	all_v2.0.5134.C1	Ambpl protein; n=5; Danio rerio|Rep: Ambpl protein - Danio rerio (Zebrafish) (Brachydanio rerio)

[a]Fold-changes are presented as outputted by siggenes and were calculated as pIC/PBS

Supplemental Table S7. 290 Genes differentially expressed between fish injected with pIC at 10°C and 16°C and sampled at 6HPI

Probe Id	d-value	Stdev	Raw p	q-value	Fold Change[a]	Sequence Name	Gene Name
38356	6.07	0.50	0.0000	0.0026	11.14	all_v2.0.415.C6	Homolog of Homo sapiens Spastic ataxia of Charlevoix-Saguenay; n=1; Takifugu rubripes
38613	4.40	0.57	0.0002	0.0092	8.45	all_v2.0.14.CB5	Interferon stimulated gene 15; n=1; Gadus morhua
38354	8.79	0.31	0.0000	0.0016	8.02	all_v2.0.707.C4	Homolog of Homo sapiens Spastic ataxia of Charlevoix-Saguenay; n=1; Takifugu rubripes
38638	5.85	0.44	0.0000	0.0026	7.90	all_v2.0.638.C1	Interleukin-8 variant 2; n=3; Ictalurus punctatus
47384	7.35	0.37	0.0000	0.0019	7.71	all_v2.0.707.C1	Unassigned protein
38358	7.33	0.36	0.0000	0.0019	7.45	all_v2.0.9190.C1	Homolog of Homo sapiens Spastic ataxia of Charlevoix-Saguenay; n=1; Takifugu rubripes
44448	5.66	0.44	0.0000	0.0027	7.29	CK10ad	custom - CC chemokine
44453	6.42	0.39	0.0000	0.0022	7.20	CK10a	custom - CC chemokine
46200	8.02	0.31	0.0000	0.0018	7.00	all_v2.0.14575.C1	SACS; sacsin
47164	14.93	0.16	0.0000	0.0016	6.72	all_v2.0.6027.C6	Unassigned protein
44590	7.90	0.33	0.0000	0.0018	6.58	DXH4a	custom - probable ATP-dependent RNA helicase DHX
38651	4.99	0.48	0.0001	0.0050	6.19	sb_gmapht_0023a03.pDNRF2	Intraflagellar transport protein 52; n=4; Danio rerio
47329	6.29	0.41	0.0000	0.0024	6.13	all_v2.0.415.C3	Unassigned protein
38359	8.15	0.28	0.0000	0.0018	6.01	all_v2.0.415.C5	Homolog of Homo sapiens Spastic ataxia of Charlevoix-Saguenay; n=1; Takifugu rubripes
44859	7.55	0.30	0.0000	0.0018	5.92	all_v2.0.314.C1	HECT domain containing protein
38611	5.37	0.43	0.0000	0.0035	5.90	all_v2.0.1767.C1	Interferon stimulated gene 15; n=1; Gadus morhua
38639	5.20	0.42	0.0000	0.0041	5.88	all_v2.0.638.CB1	Interleukin-8 variant 2; n=3; Ictalurus punctatus
38357	9.36	0.24	0.0000	0.0016	5.70	all_v2.0.8243.C1	Homolog of Homo sapiens Spastic ataxia of Charlevoix-Saguenay; n=1; Takifugu rubripes
46226	6.75	0.34	0.0000	0.0022	5.68	all_v2.0.415.C1	Serpentine_recp domain containing protein
38604	7.28	0.32	0.0000	0.0019	5.62	all_v2.0.14.CB2	Interferon stimulated gene 15; n=1; Gadus morhua
41316	9.24	0.25	0.0000	0.0016	5.61	all_v2.0.1009.C5	Probable ATP-dependent helicase LGP2 (EC 3.6.1.-) (Protein D11Lgp2 homolog).; n=1; Takifugu rubripes
38605	5.94	0.39	0.0000	0.0026	5.58	all_v2.0.14.C13	Interferon stimulated gene 15; n=1; Gadus morhua
41269	9.53	0.24	0.0000	0.0016	5.56	sb_gmnlbfic_0004o20.t7	PREDICTED: similar to zinc finger, NFX1-type containing 1; n=3; Laurasiatheria
43201	6.42	0.34	0.0000	0.0022	5.43	all_v2.0.9330.C1	Viperin-like protein; n=1; Channa argus
44180	8.24	0.26	0.0000	0.0018	5.24	all_v2.0.1266.C1	ZPC4; n=2; Oryzias latipes
38606	5.56	0.39	0.0000	0.0029	5.20	all_v2.0.14.CB4	Interferon stimulated gene 15; n=1; Gadus morhua
44589	8.33	0.26	0.0000	0.0018	5.06	DXH3b	custom - probable ATP-dependent RNA helicase DHX
38608	6.30	0.33	0.0000	0.0024	4.80	all_v2.0.14.C12	Interferon stimulated gene 15; n=1; Gadus morhua
38609	5.45	0.38	0.0000	0.0032	4.67	all_v2.0.14.C3	Interferon stimulated gene 15; n=1; Gadus morhua
46199	8.81	0.22	0.0000	0.0016	4.51	all_v2.0.2412.C1	SACS; sacsin
53704	5.66	0.38	0.0000	0.0027	4.34	all_v2.0.1006.C1	unclassified

46202	6.71	0.28	0.0000	0.0022	4.24	sb_gmnbmd_0010a21.pDNRm13r	Sacs_predicted; sacsin (predicted)
44464	5.57	0.31	0.0000	0.0028	3.86	CK10add	custom - CC chemokine
38614	6.34	0.28	0.0000	0.0023	3.81	all_v2.0.7062.C2	Interferon stimulated gene 15; n=1; Gadus morhua
45361	5.47	0.31	0.0000	0.0032	3.78	all_v2.0.34.C2	LOC797694; similar to solute carrier family 25 member 5 protein
38603	5.09	0.34	0.0001	0.0046	3.77	all_v2.0.14.CB7	Interferon stimulated gene 15; n=1; Gadus morhua
41314	5.78	0.30	0.0000	0.0027	3.77	all_v2.0.5598.C1	Probable ATP-dependent helicase LGP2 (EC 3.6.1.-) (Protein D11Lgp2 homolog).; n=1; Takifugu rubripes
38612	5.77	0.31	0.0000	0.0027	3.76	all_v2.0.14.CB3	Interferon stimulated gene 15; n=1; Gadus morhua
44588	8.54	0.20	0.0000	0.0018	3.73	DXH2b	custom - probable ATP-dependent RNA helicase DHX
47156	6.73	0.27	0.0000	0.0022	3.66	all_v2.0.617.C5	Unassigned protein
44584	9.44	0.17	0.0000	0.0016	3.55	DXH1a	custom - probable ATP-dependent RNA helicase DHX
36525	3.73	0.40	0.0007	0.0203	3.53	all_v2.0.16620.C1	Claudin 30; n=1; Oreochromis mossambicus
55502	4.24	0.37	0.0002	0.0111	3.43	all_v2.0.7892.C1	zgc:110548; zgc:110548
43196	5.76	0.28	0.0000	0.0027	3.35	all_v2.0.3448.C3	VHSV-induced protein; n=1; Oncorhynchus mykiss
37299	4.28	0.33	0.0002	0.0105	3.19	all_v2.0.3679.C1	Gadd45al protein; n=2; Danio rerio
52069	4.96	0.30	0.0001	0.0051	3.13	all_v2.0.6954.C2	unclassified
43197	4.86	0.33	0.0001	0.0054	3.13	all_v2.0.3448.C4	VHSV-induced protein; n=1; Oncorhynchus mykiss
44624	5.17	0.27	0.0000	0.0043	3.03	Codviperin1	custom - viperin
54090	7.69	0.18	0.0000	0.0018	3.02	all_v2.0.2294.C1	unclassified
42073	4.44	0.30	0.0002	0.0088	2.93	all_v2.0.15254.C1	Serine hydrolase-like protein 2 (EC 3.1.-.-).; n=1; Takifugu rubripes
38617	4.03	0.34	0.0004	0.0137	2.80	all_v2.0.13188.C1	Interferon-inducible GTPase 5 (EC 3.6.5.-) (Immunity-related GTPase cinema 1).; n=3; Takifugu rubripes
38353	5.85	0.22	0.0000	0.0026	2.78	all_v2.0.415.C2	Homolog of Homo sapiens Spastic ataxia of Charlevoix-Saguenay; n=1; Takifugu rubripes
41829	6.48	0.21	0.0000	0.0022	2.77	sb_gmnblits_0018p08.pDNRF2	RFK protein; n=2; Bos taurus
55096	3.54	0.37	0.0010	0.0260	2.76	all_v2.0.1736.C2	unclassified
44591	7.02	0.19	0.0000	0.0021	2.74	DXH4b	custom - probable ATP-dependent RNA helicase DHX
41942	6.23	0.21	0.0000	0.0025	2.73	all_v2.0.8660.C1	RING finger protein 213; n=3; Homo sapiens
48258	5.72	0.23	0.0000	0.0027	2.72	all_v2.0.14926.C1	Unassigned protein
39328	3.94	0.38	0.0004	0.0152	2.67	all_v2.0.2213.C1	NFX1-type zinc finger-containing protein 1; n=5; Eutheria
38695	4.62	0.27	0.0001	0.0070	2.66	all_v2.0.7632.C1	K14 protein; n=1; Xenopus laevis
41595	5.75	0.22	0.0000	0.0027	2.65	sb_gmapov_0028l22.pDNRF2	PRP4 pre-mRNA processing factor 4 homolog; n=2; Danio rerio
41313	5.47	0.23	0.0000	0.0032	2.58	all_v2.0.1009.C1	Probable ATP-dependent helicase LGP2 (EC 3.6.1.-) (Protein D11Lgp2 homolog).; n=1; Takifugu rubripes
44585	5.72	0.22	0.0000	0.0027	2.56	DXH2a	custom - probable ATP-dependent RNA helicase DHX
49575	4.44	0.26	0.0002	0.0088	2.52	all_v2.0.3263.C1	unclassified
37904	7.33	0.16	0.0000	0.0019	2.52	sb_gmnbbrts_0003k13.pDNRF2	Homolog of Gallus gallus Double-stranded RNA-activated protein kinase.; n=1; Takifugu rubripes

41027	4.84	0.26	0.0001	0.0055	2.50	sb_gmlbgits_0004k02.sp6	PREDICTED: similar to SPAC167.01; n=1; Danio rerio
44546	4.86	0.23	0.0001	0.0054	2.48	IRF1OK4	custom - Interferon regulatory factor 10K
51382	4.16	0.27	0.0003	0.0119	2.48	all_v2.0.5897.C3	unclassified
41582	3.89	0.30	0.0005	0.0168	2.47	all_v2.0.10019.C1	Protocadherin 2G25; n=1; Takifugu rubripes
39291	7.23	0.16	0.0000	0.0019	2.46	sb_gmnbmu_0014b24.pDNRF2	Nebulin.; n=1; Takifugu rubripes
44336	8.19	0.13	0.0000	0.0018	2.44	sb_gmapht_0037b08.pDNRF2	bloodthirsty [Notothenia coriiceps]
39490	5.18	0.22	0.0000	0.0042	2.44	all_v2.0.5879.C2	Novel protein similar to vertebrate nebulin; n=3; Danio rerio
44375	3.81	0.29	0.0006	0.0184	2.44	all_v2.0.16663.C1	cct5; chaperonin containing TCP1, subunit 5 (epsilon); K09497 T-complex protein 1 subunit epsilon
38598	4.58	0.25	0.0001	0.0072	2.43	all_v2.0.1580.C7	Interferon regulatory factor 1; n=1; Channa argus
38285	6.29	0.18	0.0000	0.0025	2.41	sb_gmnlbfic_0007k16.t7	Homolog of Homo sapiens Probable ATP-dependent helicase LGP2; n=1; Takifugu rubripes
55231	4.35	0.25	0.0002	0.0098	2.39	all_v2.0.8766.C1	unclassified
41940	4.48	0.25	0.0001	0.0085	2.36	sb_gmnbspic_0015c08.pDNRF2	RING finger protein 213.; n=1; Gallus gallus
48479	3.84	0.32	0.0005	0.0177	2.34	all_v2.0.6321.C4	Unassigned protein
38352	3.99	0.27	0.0004	0.0142	2.34	all_v2.0.272.C2	Homolog of Homo sapiens SOUL protein; n=1; Takifugu rubripes
54933	4.08	0.27	0.0003	0.0128	2.33	all_v2.0.5297.C2	unclassified
44543	4.15	0.26	0.0003	0.0119	2.32	IRF1OK1	custom - Interferon regulatory factor 10K
51171	3.63	0.35	0.0008	0.0230	2.32	all_v2.0.12475.C1	unclassified
49387	3.58	0.32	0.0009	0.0243	2.31	all_v2.0.12515.C1	unclassified
38260	4.86	0.21	0.0001	0.0054	2.28	all_v2.0.16638.C1	Homolog of Homo sapiens PhosPholiPase C-like 3; n=1; Takifugu rubripes
41418	3.86	0.26	0.0005	0.0174	2.26	all_v2.0.659.C1	Proteasome subunit alpha type; n=8; Euteleostomi
37059	3.76	0.27	0.0006	0.0197	2.26	all_v2.0.420.C2	Ependymin; n=1; Perca flavescens
54821	4.62	0.22	0.0001	0.0070	2.24	all_v2.0.10668.C2	unclassified
45325	5.62	0.18	0.0000	0.0027	2.23	all_v2.0.14677.C1	LOC764254; similar to transposase
38552	4.73	0.22	0.0001	0.0063	2.21	all_v2.0.10046.C1	ilvB (bacterial acetolactate synthase)-like; n=1; Takifugu rubripes
54239	4.05	0.24	0.0003	0.0134	2.19	all_v2.0.11344.C1	unclassified
51636	6.72	0.15	0.0000	0.0022	2.19	all_v2.0.1410.C1	unclassified
40489	4.51	0.22	0.0001	0.0080	2.18	sb_gmnbbr_0003e16.pDNRF2	PREDICTED: similar to G protein-coupled receptor 97; n=2; Canis familiaris
38042	6.36	0.15	0.0000	0.0022	2.18	all_v2.0.894.C5	Homolog of Homo sapiens Deoxyribonuclease gamma precursor; n=1; Takifugu rubripes
36186	5.21	0.19	0.0000	0.0041	2.17	all_v2.0.661.C2	Bloodthirsty; n=2; Notothenioidei
39326	4.33	0.23	0.0002	0.0099	2.17	all_v2.0.9595.C1	NFX1-type zinc finger-containing protein 1.; n=1; Takifugu rubripes
38655	4.74	0.20	0.0001	0.0063	2.15	all_v2.0.1909.C3	IRF7; n=1; Siniperca chuatsi
43192	4.82	0.21	0.0001	0.0057	2.15	sb_gmnlbric_0006j07.t7	Vesicular glutamate transporter 2.2; n=3; Danio rerio
53852	7.37	0.12	0.0000	0.0019	2.13	all_v2.0.6339.C1	unclassified
36195	5.65	0.16	0.0000	0.0027	2.11	sb_gmnlbfic_0007a23.t7	Bloodthirsty; n=2; Notothenioidei

38094	4.12	0.24	0.0003	0.0123	2.11	sb_gmnbhkic_0005c22.pDNRF2	Homolog of Homo sapiens HECT domain and RCC1-like domain protein 3; n=1; Takifugu rubripes
40216	4.68	0.21	0.0001	0.0067	2.10	sb_gmapte_0010k12.pDNRm13r	PREDICTED: similar to ankyrin 2, neuronal,; n=1; Danio rerio
44283	4.87	0.19	0.0001	0.0054	2.10	sb_gmnlmfta_0004i04.t7	ARL6IP6; ADP-ribosylation-like factor 6 interacting protein 6
38607	3.82	0.24	0.0005	0.0181	2.10	all_v2.0.7062.C1	Interferon stimulated gene 15; n=1; Gadus morhua
37060	4.84	0.20	0.0001	0.0055	2.09	sb_gmnlsras_0003k23.t7	Eph-like receptor tyrosine kinase rtk5; n=4; Danio rerio
41014	5.62	0.17	0.0000	0.0027	2.08	all_v2.0.14158.C1	PREDICTED: similar to Snf2-related CBP activator protein; n=1; Mus musculus
43007	4.92	0.20	0.0001	0.0051	2.08	sb_gmapov_0013f18.pDNRF2	UPI000065EC34 related cluster; n=1; Takifugu rubripes
40846	4.00	0.24	0.0004	0.0142	2.07	all_v2.0.4119.C1	PREDICTED: similar to Poly [ADP-ribose] polymerase 14 (PARP-14) (B aggressive lymphoma protein 2); n=1; Danio rerio
36311	4.67	0.20	0.0001	0.0067	2.05	all_v2.0.12206.C1	Cardiac troponin T; n=14; Danio rerio
36190	5.60	0.16	0.0000	0.0028	2.05	sb_gmapht_0014i15.pDNRF2	Bloodthirsty; n=2; Notothenioidei
54421	5.26	0.17	0.0000	0.0041	2.04	all_v2.0.1129.C1	unclassified
41103	5.65	0.16	0.0000	0.0027	2.04	sb_gmnbhkic_0006n17.pDNRF2	PREDICTED: similar to transposase, partial; n=1; Strongylocentrotus purpuratus
44587	4.95	0.18	0.0001	0.0051	2.04	DXH3a	custom - probable ATP-dependent RNA helicase DHX
40019	4.72	0.19	0.0001	0.0063	2.03	sb_gmnbhkas_0006d14.pDNRF2	Pol polyprotein; n=2; Walleye epidermal hyperplasia virus 2
55269	5.32	0.16	0.0000	0.0037	2.03	all_v2.0.9958.C1	unclassified
38171	3.66	0.25	0.0008	0.0222	2.03	all_v2.0.4661.C1	Homolog of Homo sapiens Microtubule-associated protein 2 isoforM 3; n=1; Takifugu rubripes
46586	3.48	0.25	0.0011	0.0278	2.02	sb_gmnlem_0013j07.sp6	Unassigned protein
47806	3.60	0.26	0.0009	0.0243	2.02	sb_gmnsul1_0001h03.pDNRF2	Unassigned protein
47020	4.54	0.20	0.0001	0.0077	2.02	all_v2.0.526.C2	Unassigned protein
36185	4.72	0.19	0.0001	0.0063	2.01	all_v2.0.661.C20	Bloodthirsty; n=2; Notothenioidei
44586	5.49	0.16	0.0000	0.0031	2.00	DXH1b	custom - probable ATP-dependent RNA helicase DHX
42842	6.12	0.14	0.0000	0.0026	2.00	sb_gmnlmfta_0005n11.t7	Twinfilin-2; n=2; Danio rerio
38670	4.95	0.18	0.0001	0.0051	2.00	all_v2.0.3891.C1	Isoform 2 of Q5RI56 ; n=2; Danio rerio
39662	4.30	0.20	0.0002	0.0102	1.99	all_v2.0.6694.C2	Novel protein; n=3; Danio rerio
55314	4.48	0.19	0.0001	0.0085	1.98	all_v2.0.4690.C2	unclassified
55171	7.43	0.11	0.0000	0.0019	1.97	all_v2.0.14663.C1	unclassified
39325	5.47	0.16	0.0000	0.0032	1.96	all_v2.0.9595.C2	NFX1-type zinc finger-containing protein 1.; n=1; Takifugu rubripes
49072	3.47	0.25	0.0012	0.0285	1.96	all_v2.0.16355.C1	unclassified
45046	5.05	0.16	0.0001	0.0047	1.95	sb_gmapht_0020j08.pDNRF2	LOC420518; similar to putative tyrosine phosphatase
38154	3.74	0.22	0.0006	0.0202	1.95	all_v2.0.420.C1	Homolog of Homo sapiens Mammalian ependymin related protein-1 precursor; n=1; Takifugu rubripes
41096	3.55	0.23	0.0010	0.0256	1.94	all_v2.0.1554.C7	PREDICTED: similar to transposase (putative); n=1; Danio rerio
54507	4.19	0.20	0.0003	0.0116	1.93	all_v2.0.6751.C1	unclassified

40670	4.80	0.17	0.0001	0.0058	1.93	all_v2.0.14446.C1	PREDICTED: similar to mitochondrial carrier triple repeat 6; n=1; Monodelphis domestica
41317	5.22	0.16	0.0000	0.0041	1.92	all_v2.0.1165.C4	Probable ATP-dependent helicase LGP2 (EC 3.6.1.-) (Protein D11Lgp2 homolog).; n=1; Takifugu rubripes
36317	4.56	0.17	0.0001	0.0074	1.92	all_v2.0.15280.C1	Carnitine palmitoyltransferase II; n=2; Danio rerio
46878	4.01	0.21	0.0004	0.0142	1.92	sb_gmnbhkic_0007o03.pDNRF2	Unassigned protein
43876	3.70	0.22	0.0007	0.0211	1.91	all_v2.0.1930.C13	Zgc:63602; n=2; Danio rerio
44655	6.45	0.12	0.0000	0.0022	1.91	all_v2.0.392.C1	DMP1 domain containing protein
44396	5.45	0.15	0.0000	0.0032	1.90	all_v2.0.6100.C1	CHCHD5; coiled-coil-helix-coiled-coil-helix domain containing 5
44579	4.19	0.21	0.0003	0.0116	1.90	3MKK4_MAP2K3	custom - Mitogen-activated protein kinase kinase 4
51353	3.82	0.21	0.0005	0.0181	1.89	all_v2.0.10898.C1	unclassified
52706	5.69	0.13	0.0000	0.0027	1.88	all_v2.0.17371.C1	unclassified
37831	4.12	0.19	0.0003	0.0123	1.87	sb_gmnlsfas_0002e11.t7	Homolog of Brachydanio rerio Transmembrane 9 superfamily member 2.; n=1; Takifugu rubripes
36625	8.19	0.09	0.0000	0.0018	1.87	sb_gmnbpcic_0006c06.pDNRm13r	Complement component C9; n=1; Paralichthys olivaceus
36426	5.22	0.15	0.0000	0.0041	1.85	all_v2.0.8306.C2	Cell division cycle 42; n=12; Coelomata
43642	5.22	0.14	0.0000	0.0041	1.84	all_v2.0.5071.C1	Zgc:152953; n=2; Danio rerio
43012	3.84	0.19	0.0005	0.0176	1.84	all_v2.0.9212.C1	UPI0000D8BA1C related cluster; n=1; Danio rerio
51332	4.08	0.19	0.0003	0.0129	1.84	all_v2.0.4629.C1	unclassified
44544	3.51	0.22	0.0011	0.0271	1.81	IRF1OK2	custom - Interferon regulatory factor 10K
44548	4.24	0.18	0.0002	0.0111	1.81	1Interferon_regulatory_factor_7	custom - interferon_regulatory_factor_7
46157	3.96	0.20	0.0004	0.0151	1.80	sb_gmnbhkas_0009m02.pDNRm13r	RGD1564081_predicted; similar to novel protein similar to human oligophrenin 1 (OPHN1) (predicted)
52488	5.06	0.14	0.0001	0.0047	1.80	all_v2.0.10916.C1	unclassified
55211	5.36	0.13	0.0000	0.0035	1.78	all_v2.0.6615.C1	unclassified
37232	3.50	0.21	0.0011	0.0273	1.78	all_v2.0.4884.C1	FK506 binding protein 4; n=3; Danio rerio
47613	3.77	0.20	0.0006	0.0192	1.76	sb_gmnbbr_0013m15.pDNRm13r	Unassigned protein
46953	6.32	0.11	0.0000	0.0024	1.76	sb_gmnbhkic_0007o03.pDNRm13r	Unassigned protein
39119	4.13	0.18	0.0003	0.0123	1.75	all_v2.0.5993.C1	Mitochondrial Rho GTPase 1-A; n=1; Danio rerio
36789	4.35	0.15	0.0002	0.0098	1.74	sb_gmnlkfic_0001a07.t7	Cytosolic nonspecific dipeptidase; n=3; Percomorpha
43195	4.16	0.16	0.0003	0.0119	1.73	all_v2.0.3448.C1	VHSV-induced protein; n=1; Oncorhynchus mykiss
47411	6.26	0.10	0.0000	0.0025	1.72	sb_gmnlskic_0010l20.pDNRm13r	Unassigned protein
52606	4.22	0.16	0.0002	0.0113	1.71	all_v2.0.5996.C2	unclassified
42585	4.06	0.17	0.0003	0.0131	1.71	all_v2.0.2335.C1	Titin a; n=3; Danio rerio
38160	4.73	0.13	0.0001	0.0063	1.71	sb_gmnlsfic_00	Homolog of Homo sapiens Membrane

						03i11.t7	interacting protein of RGS16; n=1; Takifugu rubripes
37698	4.17	0.16	0.0003	0.0119	1.70	sb_gmnbpcic_0001e04.pDNRm13r	Homolog of Brachydanio rerio Cyclin L ania-6a.; n=2; Takifugu rubripes
44547	3.95	0.16	0.0004	0.0152	1.70	IRF1OK5	custom - Interferon regulatory factor 10K
40263	6.16	0.10	0.0000	0.0026	1.70	all_v2.0.661.C24	PREDICTED: similar to bloodthirsty; n=3; Danio rerio
41937	4.06	0.17	0.0003	0.0131	1.69	all_v2.0.7491.C2	RING finger protein 213.; n=1; Gallus gallus
49782	4.87	0.13	0.0001	0.0054	1.68	all_v2.0.17197.C1	unclassified
39505	4.00	0.17	0.0004	0.0142	1.68	sb_gmapov_0018l09.pDNRF2	Novel protein similar to vertebrate phosphoribosyl pyrophosphate amidotransferase; n=3; Danio rerio
38444	3.75	0.17	0.0006	0.0197	1.68	all_v2.0.10457.C1	Homolog of Homo sapiens Zinc finger CCCH type domain containing protein 1; n=2; Takifugu rubripes
36189	5.73	0.11	0.0000	0.0027	1.68	all_v2.0.661.C31	Bloodthirsty; n=2; Notothenioidei
51450	4.47	0.15	0.0001	0.0085	1.68	all_v2.0.12876.C1	unclassified
40930	4.44	0.15	0.0002	0.0088	1.67	sb_gmnbspic_0012b01.pDNRF2	PREDICTED: similar to retinoblastoma-associated protein 140; n=2; Canis lupus familiaris
36193	3.57	0.18	0.0009	0.0247	1.67	all_v2.0.661.C39	Bloodthirsty; n=2; Notothenioidei
52340	4.17	0.16	0.0003	0.0119	1.66	all_v2.0.3337.C1	unclassified
51059	4.00	0.15	0.0004	0.0142	1.65	all_v2.0.690.C5	unclassified
40018	5.74	0.10	0.0000	0.0027	1.65	all_v2.0.7260.C1	Pol polyprotein; n=1; Nosema bombycis
47248	3.63	0.19	0.0008	0.0229	1.65	all_v2.0.3503.C1	Unassigned protein
38619	4.12	0.14	0.0003	0.0123	1.63	all_v2.0.3448.C5	Interferon-inducible protein Gig1; n=1; Psetta maxima
45146	4.97	0.12	0.0001	0.0051	1.62	sb_gmnbhkic_0005i22.pDNRm13r	LOC555735; similar to DNA methyltransferase
46309	4.75	0.12	0.0001	0.0062	1.62	sb_gmapht_0002g19.pDNRF2	TatC domain containing protein
49141	3.59	0.17	0.0009	0.0243	1.62	all_v2.0.14559.C1	unclassified
45123	3.51	0.17	0.0010	0.0270	1.62	all_v2.0.13960.C1	LOC501925; similar to solute carrier family 2, member 9 isoform a; K08146 MFS transporter, SP family, solute carrier family 2 (facilitated glucose transporter), member 9
37707	6.42	0.08	0.0000	0.0022	1.62	sb_gmapov_0029c16.pDNRF2	Homolog of Brachydanio rerio Fem1c.; n=1; Takifugu rubripes
48214	6.41	0.09	0.0000	0.0022	1.61	sb_gmnbhkas_0014f23.pDNRm13r	Unassigned protein
42021	3.85	0.15	0.0005	0.0174	1.61	all_v2.0.9962.C1	Sdf1a; n=2; Danio rerio
49723	6.05	0.09	0.0000	0.0026	1.60	all_v2.0.4732.C1	unclassified
38355	4.02	0.15	0.0004	0.0140	1.60	all_v2.0.8243.C2	Homolog of Homo sapiens Spastic ataxia of Charlevoix-Saguenay; n=1; Takifugu rubripes
39626	6.12	0.09	0.0000	0.0026	1.60	all_v2.0.4.CB27	Novel protein; n=2; Danio rerio
52780	4.07	0.14	0.0003	0.0131	1.59	all_v2.0.4435.C1	unclassified
41164	5.80	0.09	0.0000	0.0027	1.59	sb_gmnbspic_0006i05.pDNRm13r	PREDICTED: similar to tubby super-family protein isoform 2; n=2; Monodelphis domestica
43017	3.45	0.17	0.0012	0.0291	1.59	sb_gmnbmd_0011m22.pDNRF2	UPI0000D8BB84 related cluster; n=3; Danio rerio

47445	3.68	0.15	0.0007	0.0217	1.59	sb_gmnbmd_0004h11.t7	Unassigned protein
41827	3.65	0.16	0.0008	0.0223	1.58	sb_gmapov_0017j24.pDNRF2	Rfc5 protein; n=5; Clupeocephala
55194	3.74	0.16	0.0006	0.0202	1.58	all_v2.0.9305.C1	unclassified
44663	6.46	0.08	0.0000	0.0022	1.58	all_v2.0.7931.C1	Drf_FH1 multi-domain protein
45143	4.12	0.13	0.0003	0.0123	1.58	all_v2.0.8322.C2	LOC540789; similar to poly (ADP-ribose) polymerase family, member 14
47368	5.35	0.10	0.0000	0.0035	1.57	sb_gmnlla_0036m01.t7	Unassigned protein
44550	4.32	0.13	0.0002	0.0100	1.57	IRAK41	custom - interleukin-1 receptor-associated kinase 4
55249	3.83	0.15	0.0005	0.0181	1.57	all_v2.0.3039.C5	unclassified
42860	3.56	0.16	0.0009	0.0250	1.56	all_v2.0.7093.C1	Type II inositol-1,4,5-trisphosphate 5-phosphatase precursor (EC 3.1.3.36) (Phosphoinositide 5-phosphatase) (5PTase) (75 kDa inositol polyphosphate-5-phosphatase).; n=2; Gallus gallus
48417	3.72	0.16	0.0007	0.0206	1.56	sb_gmnbpcic_0009d03.pDNRm13r	Unassigned protein
51062	3.82	0.14	0.0005	0.0181	1.55	all_v2.0.8508.C1	unclassified
50297	4.07	0.13	0.0003	0.0131	1.55	all_v2.0.12538.C1	unclassified
35880	6.84	0.07	0.0000	0.0022	1.55	sb_gmnllfta_0005j01.t7	Alpha-aminoadipic semialdehyde dehydrogenase; n=12; Eutheria
48803	4.24	0.13	0.0002	0.0111	1.54	sb_gmapte_0006b21.pDNRF2	Unassigned protein
41718	3.75	0.14	0.0006	0.0199	1.53	all_v2.0.12871.C1	RBM39 protein; n=4; Eutheria
51908	3.73	0.14	0.0007	0.0203	1.53	all_v2.0.14478.C1	unclassified
50011	6.41	0.07	0.0000	0.0022	1.53	all_v2.0.1566.C1	unclassified
44015	4.15	0.12	0.0003	0.0119	1.52	all_v2.0.11085.C1	Zgc:86905; n=1; Danio rerio
42386	3.52	0.14	0.0010	0.0267	1.52	all_v2.0.14020.C1	STEAP family member 4; n=1; Oncorhynchus mykiss
49081	5.45	0.09	0.0000	0.0032	1.51	all_v2.0.4460.C1	unclassified
44256	4.36	0.11	0.0002	0.0096	1.51	all_v2.0.1221.C3	AIG1 domain containing protein
46412	3.77	0.13	0.0006	0.0192	1.51	all_v2.0.10562.C1	Ubiquitin family protein
47749	3.87	0.13	0.0005	0.0171	1.51	sb_gmnbmu_0003o07.pDNRF2	Unassigned protein
40051	4.88	0.10	0.0001	0.0054	1.51	all_v2.0.7433.C1	Poly [ADP-ribose] polymerase 12 (EC 2.4.2.30) (PARP-12) (Zinc finger CCCH domain-containing protein 1).; n=1; Takifugu rubripes
39384	3.94	0.12	0.0004	0.0152	1.50	all_v2.0.3955.C1	Novel NACHT domanin containing protein; n=2; Danio rerio
52513	4.32	0.11	0.0002	0.0100	1.49	all_v2.0.14032.C1	unclassified
54433	3.85	0.12	0.0005	0.0174	1.48	all_v2.0.2698.C2	unclassified
41562	4.00	0.12	0.0004	0.0142	1.48	sb_gmnlmfta_0004n10.t7	Protein-tyrosine phosphatase-like member A; n=9; Eutheria
46415	3.60	0.14	0.0009	0.0242	1.48	sb_gmnlla_0040g13.t7	U-edititng enzyme APOBEC-2 gb
36194	3.59	0.13	0.0009	0.0243	1.48	all_v2.0.12049.C1	Bloodthirsty; n=2; Notothenioidei
42375	3.95	0.12	0.0004	0.0152	1.47	sb_gmnbspic_0005a23.pDNRF2	SRY-box containing gene 11a; n=1; Gnathonemus petersii
55247	4.39	0.10	0.0002	0.0093	1.47	all_v2.0.5047.C	unclassified

						1	
44195	4.31	0.10	0.0002	0.0101	1.47	sb_gmnlem_0025b10.t7	[E] COG0421 Spermidine synthase
39971	3.72	0.13	0.0007	0.0206	1.47	all_v2.0.10807.C1	phospholipase C delta 3; n=1; Takifugu rubripes
43079	3.99	0.11	0.0004	0.0142	1.46	sb_gmapht_0004g05.pDNRF2	UPI0000E4E275 related cluster; n=1; Danio rerio
42172	5.58	0.07	0.0000	0.0028	1.46	sb_gmnlla_0045h19.t7	Si:dkey-21k10.1 protein; n=3; Danio rerio
41894	4.66	0.09	0.0001	0.0067	1.45	all_v2.0.2051.C1	Ribosomal protein L32; n=5; Euteleostei
51902	4.91	0.09	0.0001	0.0052	1.45	all_v2.0.11621.C1	unclassified
38489	3.49	0.13	0.0011	0.0276	1.45	all_v2.0.2131.C1	Homolog of Salmo salar Pentraxin precursor.; n=1; Takifugu rubripes
36367	4.28	0.10	0.0002	0.0105	1.45	all_v2.0.1055.C2	Cathepsin L; n=2; Percomorpha
50298	3.66	0.12	0.0008	0.0222	1.45	all_v2.0.17232.C1	unclassified
42028	3.55	0.13	0.0010	0.0256	1.44	sb_gmnbhkic_0012l24.pDNRF2	Secernin 3; n=3; Tetraodontidae
36407	4.12	0.10	0.0003	0.0123	1.44	all_v2.0.1864.C7	CD9 protein; n=1; Oncorhynchus mykiss
43951	4.89	0.08	0.0001	0.0053	1.44	sb_gmnbpcic_0010g12.pDNRF2	Zgc:77292; n=2; Danio rerio
35894	3.65	0.12	0.0008	0.0223	1.43	all_v2.0.16939.C1	Aminopeptidase N; n=2; Pseudopleuronectes americanus
54855	3.85	0.11	0.0005	0.0175	1.43	all_v2.0.8904.C1	unclassified
43202	4.93	0.08	0.0001	0.0051	1.42	all_v2.0.9330.C2	Viperin-like protein; n=1; Channa argus
55134	4.05	0.10	0.0003	0.0134	1.42	all_v2.0.392.C2	unclassified
38436	3.58	0.11	0.0009	0.0243	1.41	sb_gmapov_0005h22.pDNRF2	Homolog of Homo sapiens Vacuolar sorting protein 54 long isoform; n=1; Takifugu rubripes
49022	4.05	0.09	0.0003	0.0133	1.41	sb_gmnlskic_0009d23.pDNRm13r	Unassigned protein
36328	3.80	0.11	0.0006	0.0185	1.40	all_v2.0.16926.C1	CASP8 and FADD-like apoptosis regulator precursor (Cellular FLICE-like inhibitory protein) (c-FLIP) (Caspase-eight-related protein) (Casper) (Caspase-like apoptosis regulatory protein) (CLARP) (MACH-related inducer of toxicity) (MRIT) (Caspase homolog) (C; n=1; Takifugu rubripes
44066	4.16	0.09	0.0003	0.0119	1.40	all_v2.0.7765.C1	Zgc:92254; n=2; Danio rerio
40840	4.37	0.09	0.0002	0.0095	1.39	sb_gmnbhkas_0018n05.pDNRF2	PREDICTED: similar to poly (ADP-ribose) polymerase family, member 11,; n=1; Monodelphis domestica
48696	3.77	0.10	0.0006	0.0192	1.39	all_v2.0.40.C3	Unassigned protein
36150	3.49	0.11	0.0011	0.0275	1.39	sb_gmapov_0026e24.pDNRF2	Beta-glucuronidase precursor; n=1; Sus scrofa
38854	3.59	0.10	0.0009	0.0243	1.38	all_v2.0.4197.C1	LOC794166 protein; n=2; Danio rerio
52110	4.31	0.08	0.0002	0.0101	1.37	all_v2.0.8876.C1	unclassified
38561	3.51	0.11	0.0011	0.0271	1.37	all_v2.0.16357.C1	Importin alpha; n=1; Pagrus major
38422	3.67	0.10	0.0007	0.0220	1.37	sb_gmapov_0019e16.pDNRF2	Homolog of Homo sapiens Transport-secretion protein 2.2; n=1; Takifugu rubripes
38263	3.52	0.10	0.0010	0.0270	1.36	all_v2.0.10484.C1	Homolog of Homo sapiens Placenta-specific gene 8 protein; n=1; Takifugu rubripes
43674	5.11	0.06	0.0001	0.0046	1.36	all_v2.0.13048.C1	Zgc:153384; n=1; Danio rerio
53244	3.77	0.09	0.0006	0.0193	1.36	all_v2.0.1478.C3	unclassified

39323	3.65	0.10	0.0008	0.0223	1.36	all_v2.0.6027.C9	NFX1-type zinc finger-containing protein 1.; n=1; Takifugu rubripes
37498	3.57	0.10	0.0009	0.0247	1.36	all_v2.0.3198.C3	GTP-binding nuclear protein Ran; n=35; Euteleostomi
52971	4.32	0.08	0.0002	0.0100	1.36	all_v2.0.7146.C1	unclassified
47948	3.88	0.09	0.0005	0.0169	1.35	all_v2.0.17446.C1	Unassigned protein
53976	4.43	0.07	0.0002	0.0088	1.34	all_v2.0.13765.C1	unclassified
44431	4.12	0.08	0.0003	0.0123	1.34	CASP106	custom - caspase 10, apoptosis-related cysteine peptidase
42664	4.75	0.06	0.0001	0.0062	1.33	sb_gmnlskic_0011d03.pDNRm13r	Transcription factor; n=8; Clupeocephala
42329	3.96	0.08	0.0004	0.0151	1.32	all_v2.0.9973.C1	Sorting nexin-25.; n=2; Gallus gallus
39741	4.63	0.06	0.0001	0.0070	1.32	all_v2.0.11115.C1	Nuclear receptor coactivator 7.; n=1; Gallus gallus
36901	3.68	0.08	0.0007	0.0218	1.32	all_v2.0.5271.C1	DNA-directed RNA polymerase I subunit RPA49 (RNA polymerase I- associated factor 53) (RNA polymerase I polypeptide E) (RNA polymerase I-associated factor 1) (A49).; n=1; Takifugu rubripes
45101	3.65	0.08	0.0008	0.0223	1.31	all_v2.0.12734.C1	LOC480945; similar to LINE-1 reverse transcriptase homolog
51606	3.75	0.08	0.0006	0.0197	1.31	all_v2.0.4073.C1	unclassified
39654	4.93	0.06	0.0001	0.0051	1.31	all_v2.0.4.CB39	Novel protein; n=3; Danio rerio
38114	4.16	0.07	0.0003	0.0119	1.30	all_v2.0.3580.C6	Homolog of Homo sapiens Inter-alpha (globulIn) InhIbItor H3; n=1; Takifugu rubripes
46677	4.16	0.07	0.0003	0.0119	1.30	all_v2.0.4.C247	Unassigned protein
39586	3.70	0.07	0.0007	0.0211	1.29	all_v2.0.14243.C1	Novel protein; n=2; Danio rerio
36187	6.02	0.04	0.0000	0.0026	1.29	sb_gmnbspic_0012h03.pDNRm13r	Bloodthirsty; n=2; Notothenioidei
44937	3.85	0.07	0.0005	0.0174	1.28	all_v2.0.5180.C1	Itgax; integrin alpha X; K06462 integrin alpha X
49145	3.57	0.07	0.0009	0.0247	1.27	all_v2.0.14514.C1	unclassified
40724	4.04	0.06	0.0003	0.0134	1.27	sb_gmapht_0004l21.pDNRF2	PREDICTED: similar to NOD3 protein; n=1; Danio rerio
52165	3.51	0.07	0.0011	0.0271	1.26	all_v2.0.2593.C1	unclassified
54622	3.62	0.07	0.0008	0.0237	1.26	all_v2.0.15445.C1	unclassified
51980	4.09	0.05	0.0003	0.0128	1.25	all_v2.0.703.C1	unclassified
45088	3.51	0.06	0.0010	0.0270	1.24	sb_gmnlkric_0002a08.t7	LOC477822; similar to hyaluronan binding protein 2 [EC:3.4.21.-]; K08648 hyaluronan binding protein 2
54167	3.48	0.05	0.0011	0.0277	1.19	all_v2.0.3108.C1	unclassified
40407	3.65	0.04	0.0008	0.0223	1.17	all_v2.0.17488.C1	PREDICTED: similar to DNA-directed RNA polymerase II largest subunit; n=1; Homo sapiens
48016	3.60	0.04	0.0009	0.0242	1.16	all_v2.0.4.C264	Unassigned protein
48778	-5.04	0.04	0.0001	0.0049	0.80	sb_gmnbhkas_0017b15.pDNRm13r	Unassigned protein
51863	-4.92	0.06	0.0001	0.0051	0.76	all_v2.0.3829.C1	unclassified
42500	-5.15	0.06	0.0000	0.0045	0.74	sb_gmnbhkic_0011p05.pDNRm13r	T-complex protein 1 subunit delta; n=21; Tetrapoda
45360	-6.02	0.06	0.0000	0.0026	0.71	all_v2.0.1480.C1	LOC797694; similar to solute carrier family 25 member 5 protein

49813	-4.97	0.10	0.0001	0.0051	0.64	all_v2.0.2128.C1	unclassified
35984	-4.86	0.13	0.0001	0.0054	0.60	sb_gmnbmu_0001o21.pDNRF2	Ariadne homolog, ubiquitin-conjugating enzyme E2 binding protein, 1 like; n=6; Euteleostomi
39895	-7.36	0.08	0.0000	0.0019	0.59	all_v2.0.3146.C1	Peptidyl-prolyl cis-trans isomerase; n=2; Tetraodontidae
39896	-4.81	0.17	0.0001	0.0057	0.54	all_v2.0.3146.C2	Peptidyl-prolyl cis-trans isomerase; n=2; Tetraodontidae
53859	-4.81	0.19	0.0001	0.0057	0.50	all_v2.0.5016.C2	unclassified
49585	-5.10	0.25	0.0001	0.0046	0.41	all_v2.0.115.C1	unclassified
38067	-5.09	0.26	0.0001	0.0046	0.38	sb_gmnbmu_0012g06.pDNRm13r	Homolog of Homo sapiens FK506 binding protein 10 precursor; n=1; Takifugu rubripes

[a]Fold-chages are presented as outputted by siggenes and were calculated as pIC/PBS

Supplemental Table S8. 339 Genes differentially expressed between fish injected with pIC at 10°C and 16°C and sampled at 24HPI

Probe ID	d-value	Stdev	Raw p	q-value	Fold Change[a]	Sequence Name	Gene Name
53704	6.25	0.30	0.0000	0.0028	4.10	all_v2.0.1006.C1	unclassified
45392	9.53	0.14	0.0000	0.0026	3.16	all_v2.0.341.C5	Metallothio domain containing protein
42545	4.29	0.22	0.0001	0.0089	2.17	all_v2.0.6888.C1	Thioredoxin; n=1; Ictalurus punctatus
55146	4.65	0.21	0.0001	0.0062	2.12	all_v2.0.712.C1	unclassified
43363	4.90	0.16	0.0000	0.0053	1.92	all_v2.0.5853.C1	Zgc:103456; n=1; Danio rerio
51914	5.10	0.15	0.0000	0.0044	1.89	all_v2.0.17634.C1	unclassified
50372	4.72	0.15	0.0001	0.0062	1.81	all_v2.0.3379.C2	unclassified
51982	4.98	0.13	0.0000	0.0050	1.77	all_v2.0.6362.C1	unclassified
37555	4.26	0.16	0.0001	0.0091	1.77	all_v2.0.3403.C1	Heat shock protein 47; n=1; Oncorhynchus mykiss
35767	5.04	0.13	0.0000	0.0047	1.75	all_v2.0.2545.C1	Activating transcription factor 7-interacting protein 1; n=2; Danio rerio
55263	6.86	0.08	0.0000	0.0028	1.73	all_v2.0.655.C1	unclassified
37572	4.15	0.15	0.0002	0.0102	1.67	all_v2.0.4885.C2	Heat shock protein HSP 90-alpha; n=8; Clupeocephala
45010	6.48	0.08	0.0000	0.0028	1.66	all_v2.0.4885.C1	LOC100017761; similar to 90-kDa heat shock protein
37564	5.29	0.11	0.0000	0.0040	1.66	all_v2.0.16020.C1	Heat shock protein 90; n=13; Euteleostomi
55159	4.18	0.14	0.0002	0.0099	1.65	all_v2.0.1965.C1	unclassified
37573	5.99	0.09	0.0000	0.0032	1.65	sb_gmnlkfta_0003o06.t7	Heat shock protein HSP 90-alpha; n=8; Clupeocephala
51529	4.13	0.15	0.0002	0.0104	1.65	all_v2.0.6456.C1	unclassified
40546	4.94	0.11	0.0000	0.0051	1.64	all_v2.0.2840.C1	PREDICTED: similar to heat shock protein 1, beta isoform 1; n=1; Apis mellifera
40508	5.12	0.10	0.0000	0.0044	1.61	all_v2.0.5811.C1	PREDICTED: similar to glutathione S-transferase mu isoform 1; n=1; Danio rerio
37563	7.91	0.05	0.0000	0.0028	1.57	all_v2.0.10007.C1	Heat shock protein 90; n=13; Euteleostomi
37566	4.58	0.11	0.0001	0.0065	1.55	all_v2.0.2479.C1	Heat shock protein 90Ae.; n=1; Takifugu rubripes
43036	5.57	0.08	0.0000	0.0034	1.54	sb_gmnlskic_0007p20.pDNRm13r	UPI0000D8CC26 related cluster; n=2; Danio rerio
50202	4.59	0.10	0.0001	0.0065	1.54	all_v2.0.11081.C1	unclassified
36470	4.90	0.09	0.0000	0.0053	1.54	all_v2.0.11401.C1	Chaperonin containing TCP1, subunit 6A; n=3; Euteleostomi
37574	5.62	0.08	0.0000	0.0034	1.52	all_v2.0.1961.C1	Heat shock protein HSP 90-beta; n=13; Euteleostomi
36468	4.33	0.10	0.0001	0.0088	1.49	all_v2.0.495.C1	Chaperonin containing TCP1, subunit 5; n=4; Clupeocephala
44902	7.28	0.05	0.0000	0.0028	1.47	sb_gmnblits_0026m11.pDNRF2	HSP90 multi-domain protein
37523	4.86	0.08	0.0001	0.0054	1.47	all_v2.0.10969.C1	Guanylate kinase 1; n=2; Danio rerio
41479	5.25	0.08	0.0000	0.0040	1.47	sb_gmnbhkas_0016k04.pDNRF2	Protein kinase C delta type (EC 2.7.11.13) (nPKC-delta).; n=1; Takifugu rubripes
45113	4.64	0.08	0.0001	0.0062	1.44	sb_gmapov_0018o17.pDNRF2	LOC488397; similar to Methyltransferase-like protein 2
45907	4.62	0.08	0.0001	0.0064	1.41	all_v2.0.5279.C2.a	non-directed

42467	4.94	0.06	0.0000	0.0051	1.37	sb_gmnbspic_0010b12.pDNRm13r	TANK-binding kinase 1; n=1; Danio rerio
52113	4.64	0.07	0.0001	0.0063	1.36	all_v2.0.6041.C1	unclassified
37544	4.19	0.07	0.0002	0.0097	1.35	sb_gmnlkfta_0002e06.t7	Heat shock 70kDa protein 8; n=1; Danio rerio
45645	4.42	0.06	0.0001	0.0082	1.31	all_v2.0.8202.C1	non-directed
39377	4.59	0.05	0.0001	0.0065	1.30	sb_gmnblits_0026k02.pDNRF2	Novel KRAB box and zinc finger, C2H2 type domain containing protein; n=4; Mus musculus
37878	-4.43	0.03	0.0001	0.0082	0.83	sb_gmnlskic_0014i20.pDNRm13r	Homolog of Fugu rubripes Topoisomerase I beta.; n=1; Takifugu rubripes
55532	-3.45	0.06	0.0008	0.0215	0.81	all_v2.0.10346.C1	ZNFX1; zinc finger, NFX1-type containing 1
44823	-3.74	0.05	0.0004	0.0157	0.81	sb_gmnbmd_0002f22.pDNRF2	GGT1, GGT; gamma-glutamyltransferase 1 [EC:2.3.2.2]; K00681 gamma-glutamyltranspeptidase
52234	-3.44	0.06	0.0008	0.0217	0.80	all_v2.0.8483.C1	unclassified
54760	-4.17	0.05	0.0002	0.0100	0.80	all_v2.0.5450.C1	unclassified
42617	-3.31	0.07	0.0011	0.0264	0.79	sb_gmnbpcic_0011f05.pDNRF2	Torsin B precursor (Torsin family 1 member B).; n=1; Takifugu rubripes
37158	-3.59	0.06	0.0006	0.0181	0.79	all_v2.0.11686.C1	Fance protein; n=4; Danio rerio
46972	-3.94	0.06	0.0003	0.0128	0.79	sb_gmnbhkic_0013n08.pDNRF2	Unassigned protein
36028	-3.37	0.07	0.0010	0.0242	0.79	all_v2.0.3.C4	ATP synthase subunit beta, mitochondrial precursor; n=27; Euteleostomi
46159	-3.67	0.07	0.0005	0.0166	0.78	all_v2.0.1652.C1	Rh-like protein [Apis mellifera] gb
50643	-3.36	0.08	0.0010	0.0244	0.78	all_v2.0.11230.C1	unclassified
49070	-3.81	0.07	0.0004	0.0146	0.78	all_v2.0.7789.C2	unclassified
40563	-3.60	0.07	0.0006	0.0179	0.78	sb_gmapht_0006g21.pDNRF2	PREDICTED: similar to Inositol 1,4,5-trisphosphate receptor type 2 (Type 2 inositol 1,4,5-trisphosphate receptor) (Type 2 InsP3 receptor) (IP3 receptor isoform 2) (InsP3R2) isoform 1; n=2; Eutheria
38816	-3.41	0.08	0.0009	0.0226	0.77	sb_gmapov_0014f14.pDNRF2	LOC496786 protein; n=1; Xenopus tropicalis
51403	-3.88	0.07	0.0003	0.0136	0.77	all_v2.0.6524.C1	unclassified
49961	-3.36	0.08	0.0010	0.0245	0.77	all_v2.0.12262.C1	unclassified
37118	-3.31	0.09	0.0011	0.0263	0.76	all_v2.0.2012.C1	Eukaryotic translation initiation factor 4E; n=5; Xenopus
47291	-3.46	0.08	0.0008	0.0215	0.76	all_v2.0.606.C2	Unassigned protein
42375	-3.48	0.08	0.0008	0.0212	0.76	sb_gmnbspic_0005a23.pDNRF2	SRY-box containing gene 11a; n=1; Gnathonemus petersii
42817	-3.83	0.07	0.0004	0.0143	0.76	sb_gmapov_0012m19.pDNRm13r	Tubulin alpha chain; n=36; Fungi/Metazoa group
36080	-3.36	0.09	0.0010	0.0245	0.76	sb_gmnbhkas_0015j06.pDNRF2	ATP-sensitive inward rectifier potassium channel 15 (Potassium channel, inwardly rectifying subfamily J member 15) (Inward rectifier K(+) channel Kir4.2) (Kir1.3).; n=1; Takifugu rubripes
47830	-3.33	0.09	0.0011	0.0254	0.76	sb_gmapht_0013l09.pDNRm13r	Unassigned protein
39371	-5.00	0.05	0.0000	0.0048	0.75	sb_gmnbhkas_0007i12.pDNRF2	Novel immune-type receptor 4; n=2; Oncorhynchus mykiss

45624	-3.74	0.08	0.0004	0.0157	0.75	all_v2.0.17285.C1.a	non-directed
39577	-3.51	0.09	0.0007	0.0201	0.75	all_v2.0.15015.C1	Novel protein; n=13; Danio rerio
46732	-3.28	0.10	0.0012	0.0274	0.74	sb_gmnlem_0027j18.t7	Unassigned protein
38998	-3.76	0.08	0.0004	0.0155	0.74	sb_gmnlem_0024l03.t7	MGC115621 protein; n=1; Xenopus laevis
52306	-5.19	0.05	0.0000	0.0042	0.74	all_v2.0.14732.C1	unclassified
54046	-3.47	0.09	0.0008	0.0212	0.74	all_v2.0.5705.C1	unclassified
42696	-3.89	0.08	0.0003	0.0136	0.74	all_v2.0.6415.C2	Translocon-associated protein subunit gamma; n=14; Euteleostomi
45298	-3.71	0.09	0.0004	0.0160	0.74	sb_gmapht_0036o07.pDNRF2	LOC728894; similar to gamma-aminobutyric acid (GABA-A) receptor, subunit epsilon
36551	-3.79	0.08	0.0004	0.0151	0.74	all_v2.0.2681.C1	Cofilin-1; n=15; Eutheria
46762	-3.33	0.10	0.0011	0.0254	0.74	sb_gmnbgi_0004p23.pDNRm13r	Unassigned protein
35849	-3.55	0.10	0.0006	0.0189	0.74	all_v2.0.11586.C1	Alkaline ceramidase 2 (EC 3.5.1.23) (AlkCDase 2) (Acylsphingosine deacylase 3-like) (N-acylsphingosine amidohydrolase 3-like).; n=1; Takifugu rubripes
49596	-3.93	0.08	0.0003	0.0130	0.74	all_v2.0.2907.C1	unclassified
37738	-3.74	0.09	0.0004	0.0157	0.74	sb_gmnbhkic_0007m23.pDNRF2	Homolog of Brachydanio rerio Microspherule protein 1.; n=1; Takifugu rubripes
39323	-3.29	0.10	0.0012	0.0273	0.74	all_v2.0.6027.C9	NFX1-type zinc finger-containing protein 1.; n=1; Takifugu rubripes
39258	-3.79	0.09	0.0004	0.0151	0.73	all_v2.0.9983.C1	NADH dehydrogenase [ubiquinone] 1 beta subcomplex subunit 1 (EC 1.6.5.3) (EC 1.6.99.3) (NADH-ubiquinone oxidoreductase MNLL subunit) (Complex I-MNLL) (CI-MNLL).; n=1; Takifugu rubripes
44663	-4.76	0.06	0.0001	0.0061	0.73	all_v2.0.7931.C1	Drf_FH1 multi-domain protein
51433	-3.32	0.11	0.0011	0.0259	0.73	all_v2.0.649.C1	unclassified
37289	-3.30	0.11	0.0011	0.0266	0.73	sb_gmnbhkas_0019c20.pDNRF2	G protein-coupled receptor 155; n=1; Takifugu rubripes
44550	-3.88	0.09	0.0003	0.0136	0.73	IRAK41	custom - interleukin-1 receptor-associated kinase 4
43052	-3.65	0.10	0.0005	0.0168	0.73	all_v2.0.17120.C1	UPI0000D8DF8E related cluster; n=1; Danio rerio
40604	-3.34	0.11	0.0011	0.0253	0.73	sb_gmapte_0010i04.pDNRm13r	PREDICTED: similar to KPL2; n=1; Ornithorhynchus anatinus
43147	-3.65	0.10	0.0005	0.0168	0.73	all_v2.0.4262.C1	Vacuolar ATP synthase subunit S1 precursor (EC 3.6.3.14) (V-ATPase subunit S1) (V-ATPase S1 accessory protein) (V-ATPase Ac45 subunit) (XAP-3).; n=1; Takifugu rubripes
45377	-3.70	0.10	0.0005	0.0162	0.73	all_v2.0.5627.C1	Mak16 multi-domain protein
47368	-3.38	0.11	0.0009	0.0236	0.72	sb_gmnlla_0036m01.t7	Unassigned protein
49161	-3.36	0.11	0.0010	0.0244	0.72	all_v2.0.7045.C1	unclassified
40263	-3.81	0.09	0.0004	0.0146	0.72	all_v2.0.661.C24	PREDICTED: similar to bloodthirsty; n=3; Danio rerio
51306	-3.41	0.11	0.0009	0.0226	0.72	all_v2.0.8881.C1	unclassified
52359	-3.76	0.10	0.0004	0.0155	0.72	all_v2.0.1914.C1	unclassified
54212	-3.37	0.11	0.0010	0.0242	0.72	all_v2.0.14096.C1	unclassified
39846	-3.34	0.11	0.0011	0.0253	0.72	all_v2.0.10280.C2	Palmitoyl-protein thioesterase 1 precursor (EC 3.1.2.22) (PPT-1) (Palmitoyl-protein hydrolase 1).;

							n=1; Takifugu rubripes
43609	-4.61	0.07	0.0001	0.0064	0.72	sb_gmnbmd_0004p22.t7	Zgc:136585; n=3; Danio rerio
44865	-3.59	0.11	0.0006	0.0181	0.72	all_v2.0.8362.C1	HERC5, LOC478474; hect domain and RLD 5
49061	-3.87	0.09	0.0003	0.0138	0.72	all_v2.0.10961.C1	unclassified
49108	-3.97	0.09	0.0003	0.0125	0.72	all_v2.0.210.C1	unclassified
47299	-3.86	0.10	0.0003	0.0139	0.71	sb_gmnbhkic_0005d21.pDNRF2	Unassigned protein
44547	-3.70	0.10	0.0005	0.0161	0.71	IRF1OK5	custom - Interferon regulatory factor 10K
39952	-4.13	0.09	0.0002	0.0104	0.71	all_v2.0.6525.C1	phosphatidylinositol-specific phospholipase C, X domain containing 1; n=1; Takifugu rubripes
37631	-3.52	0.12	0.0007	0.0197	0.70	all_v2.0.15318.C1	histidine triad nucleotide binding protein 3; n=1; Takifugu rubripes
44649	-5.09	0.07	0.0000	0.0044	0.70	all_v2.0.661.C6	DKEY-218F9.5, LOC571141; novel protein similar to bloodthirsty (bty)
37707	-3.99	0.10	0.0002	0.0122	0.70	sb_gmapov_0029c16.pDNRF2	Homolog of Brachydanio rerio Fem1c.; n=1; Takifugu rubripes
42362	-5.02	0.07	0.0000	0.0047	0.70	all_v2.0.1697.C1	Splicing factor, arginine/serine-rich 11; n=3; Cyprinidae
40844	-3.44	0.13	0.0008	0.0219	0.69	all_v2.0.8322.C3	PREDICTED: similar to Poly [ADP-ribose] polymerase 14 (PARP-14) (B aggressive lymphoma protein 2); n=1; Danio rerio
50515	-3.51	0.12	0.0007	0.0201	0.69	all_v2.0.7899.C1	unclassified
42922	-3.64	0.12	0.0005	0.0170	0.69	sb_gmnlpras_0001f12.t7	Ubiquitin; n=6; Eukaryota
52606	-3.47	0.12	0.0008	0.0212	0.69	all_v2.0.5996.C2	unclassified
54589	-3.27	0.13	0.0012	0.0274	0.69	all_v2.0.12596.C1	unclassified
45375	-3.27	0.14	0.0012	0.0275	0.69	sb_gmnbmu_0008h11.pDNRF2	Macro domain containing protein
48343	-3.61	0.12	0.0006	0.0178	0.69	sb_gmlbgits_0003d14.sp6	Unassigned protein
47982	-3.78	0.11	0.0004	0.0151	0.69	sb_gmapht_0015h06.pDNRF2	Unassigned protein
49472	-5.72	0.06	0.0000	0.0034	0.68	all_v2.0.12909.C1	unclassified
41103	-3.61	0.12	0.0006	0.0178	0.68	sb_gmnbhkic_0006n17.pDNRF2	PREDICTED: similar to transposase, partial; n=1; Strongylocentrotus purpuratus
50977	-3.65	0.12	0.0005	0.0168	0.68	all_v2.0.9975.C1	unclassified
39626	-4.65	0.09	0.0001	0.0062	0.68	all_v2.0.4.CB27	Novel protein; n=2; Danio rerio
41068	-3.83	0.11	0.0004	0.0143	0.68	all_v2.0.1797.C1	PREDICTED: similar to thymosin, beta 4; n=1; Canis lupus familiaris
37203	-3.71	0.12	0.0005	0.0161	0.68	all_v2.0.1097.C3	Ferritin, heavy subunit; n=2; Salmonidae
53004	-3.51	0.13	0.0007	0.0201	0.68	all_v2.0.547.C1	unclassified
38155	-4.31	0.10	0.0001	0.0089	0.68	sb_gmapov_0016d17.pDNRF2	Homolog of Homo sapiens Mannosyl (Alpha-1,3-)-glycoprotein beta-1,4-N-acetylglucosaminyltransferase, isoenzyme B, isoform 1; n=2; Clupeocephala
50391	-4.25	0.10	0.0002	0.0091	0.68	all_v2.0.2069.C2	unclassified
36901	-6.72	0.05	0.0000	0.0028	0.68	all_v2.0.5271.C1	DNA-directed RNA polymerase I subunit RPA49 (RNA polymerase I- associated factor 53) (RNA polymerase I polypeptide E) (RNA polymerase I-associated factor 1) (A49).; n=1; Takifugu rubripes
55314	-3.44	0.14	0.0008	0.0218	0.68	all_v2.0.4690.C2	unclassified
40337	-3.59	0.13	0.0006	0.0182	0.68	sb_gmnlbric_0003k18.t7	PREDICTED: similar to cGMP phosphodiesterase A2; n=1; Ornithorhynchus anatinus
36185	-4.35	0.10	0.0001	0.0088	0.68	all_v2.0.661.C2	Bloodthirsty; n=2; Notothenioidei

						0	
38073	-3.60	0.13	0.0006	0.0180	0.67	sb_gmnbspic_0009c10.pDNRF2	Homolog of Homo sapiens G protein-coupled receptor 112; n=1; Takifugu rubripes
37698	-3.94	0.11	0.0003	0.0128	0.67	sb_gmnbpcic_0001e04.pDNRm13r	Homolog of Brachydanio rerio Cyclin L ania-6a.; n=2; Takifugu rubripes
45232	-3.74	0.13	0.0004	0.0157	0.67	sb_gmapov_0010i17.pDNRm13r	LOC607320; similar to NOL1/NOP2/Sun domain family, member 3
54777	-4.56	0.10	0.0001	0.0068	0.67	all_v2.0.3549.C1	unclassified
41397	-3.57	0.14	0.0006	0.0184	0.67	all_v2.0.847.C1	Prostaglandin E synthase 3; n=5; Danio rerio
48320	-4.75	0.10	0.0001	0.0062	0.67	all_v2.0.74.C2	Unassigned protein
51104	-3.40	0.14	0.0009	0.0232	0.67	all_v2.0.14207.C2	unclassified
44015	-4.66	0.09	0.0001	0.0062	0.66	all_v2.0.11085.C1	Zgc:86905; n=1; Danio rerio
50132	-5.27	0.08	0.0000	0.0040	0.66	all_v2.0.6591.C1	unclassified
41526	-4.44	0.10	0.0001	0.0082	0.66	sb_gmapte_0011n16.pDNRF2	Protein ripply1; n=2; Danio rerio
46430	-4.69	0.10	0.0001	0.0062	0.66	all_v2.0.14645.C1	Unassigned protein
53454	-3.69	0.13	0.0005	0.0162	0.66	all_v2.0.10597.C1	unclassified
38160	-4.07	0.12	0.0002	0.0113	0.66	sb_gmnlsfic_0003i11.t7	Homolog of Homo sapiens Membrane interacting protein of RGS16; n=1; Takifugu rubripes
36317	-3.27	0.16	0.0012	0.0276	0.66	all_v2.0.15280.C1	Carnitine palmitoyltransferase II; n=2; Danio rerio
46576	-5.86	0.07	0.0000	0.0032	0.66	all_v2.0.4.C213	Unassigned protein
55349	-4.03	0.12	0.0002	0.0119	0.66	all_v2.0.8627.C1	unclassified
45980	-3.61	0.13	0.0006	0.0179	0.66	sb_gmnbmd_0011d22.pDNRF2	PDZ domain containing 2 [Mus musculus]
35957	-4.01	0.13	0.0002	0.0121	0.66	all_v2.0.15919.C1	Apolipoprotein A-IV4; n=3; Takifugu rubripes
40720	-4.34	0.11	0.0001	0.0088	0.65	all_v2.0.13762.C1	PREDICTED: similar to Nipsnap1 protein; n=1; Monodelphis domestica
41899	-5.17	0.09	0.0000	0.0042	0.65	all_v2.0.16108.C1	Ribosomal protein L5b; n=1; Ictalurus punctatus
43437	-3.51	0.14	0.0007	0.0201	0.65	all_v2.0.18305.C1	Zgc:110373; n=2; Danio rerio
41184	-3.57	0.15	0.0006	0.0184	0.65	sb_gmnlbfic_0007g21.t7	PREDICTED: similar to ubiquitin specific protease 24; n=2; Mus musculus
41088	-4.73	0.10	0.0001	0.0062	0.65	all_v2.0.3752.C1	PREDICTED: similar to transmembrane protein 7; n=1; Equus caballus
36409	-3.56	0.15	0.0006	0.0188	0.65	sb_gmapov_0017a02.pDNRF2	Cdc14a protein; n=4; Danio rerio
37498	-3.45	0.14	0.0008	0.0217	0.65	all_v2.0.3198.C3	GTP-binding nuclear protein Ran; n=35; Euteleostomi
38075	-5.30	0.09	0.0000	0.0040	0.65	all_v2.0.18643.C1	Homolog of Homo sapiens G protein-coupled receptor 64; n=1; Takifugu rubripes
43197	-4.18	0.13	0.0002	0.0099	0.64	all_v2.0.3448.C4	VHSV-induced protein; n=1; Oncorhynchus mykiss
44544	-4.42	0.11	0.0001	0.0082	0.64	IRF1OK2	custom - Interferon regulatory factor 10K
48162	-4.21	0.12	0.0002	0.0095	0.64	sb_gmnlpbia_0002b13.t7	Unassigned protein
42748	-4.29	0.12	0.0001	0.0089	0.64	all_v2.0.9596.C1	Transmembrane protein 7; n=1; Siniperca chuatsi
44375	-4.06	0.12	0.0002	0.0114	0.64	all_v2.0.16663.C1	cct5; chaperonin containing TCP1, subunit 5 (epsilon); K09497 T-complex protein 1 subunit epsilon
41902	-3.83	0.14	0.0004	0.0143	0.64	all_v2.0.1081.C6	Ribosomal protein L9e; n=4; Endopterygota
51243	-3.28	0.17	0.0012	0.0273	0.64	all_v2.0.1415.C3	unclassified

46901	-3.63	0.15	0.0005	0.0174	0.64	all_v2.0.143.C1	Unassigned protein
48258	-3.66	0.15	0.0005	0.0168	0.64	all_v2.0.14926.C1	Unassigned protein
35793	-3.28	0.17	0.0012	0.0274	0.64	sb_gmnlkfas_0002n19.t7	Adenosine kinase a; n=2; Danio rerio
53852	-5.05	0.10	0.0000	0.0047	0.64	all_v2.0.6339.C1	unclassified
36195	-5.15	0.10	0.0000	0.0043	0.64	sb_gmnlbfic_0007a23.t7	Bloodthirsty; n=2; Notothenioidei
54894	-4.05	0.14	0.0002	0.0117	0.64	all_v2.0.2012.C2	unclassified
49932	-4.36	0.12	0.0001	0.0087	0.63	all_v2.0.16740.C1	unclassified
55271	-3.72	0.14	0.0004	0.0160	0.63	all_v2.0.2992.C2	unclassified
39974	-4.42	0.12	0.0001	0.0082	0.63	all_v2.0.16283.C1	Phospholipase D4 (EC 3.1.4.4) (PLD 4) (Choline phosphatase 4) (Phosphatidylcholine-hydrolyzing phospholipase D4).; n=1; Takifugu rubripes
36338	-3.47	0.16	0.0008	0.0212	0.63	all_v2.0.7453.C2	Caspase-1; n=1; Dicentrarchus labrax
36556	-4.63	0.11	0.0001	0.0063	0.63	sb_gmnlem_0023e09.sp6	coiled-coil domain containing 59; n=1; Takifugu rubripes
36193	-3.61	0.17	0.0006	0.0178	0.62	all_v2.0.661.C39	Bloodthirsty; n=2; Notothenioidei
36186	-4.80	0.11	0.0001	0.0058	0.62	all_v2.0.661.C2	Bloodthirsty; n=2; Notothenioidei
43698	-3.48	0.18	0.0008	0.0210	0.62	all_v2.0.3261.C1	Zgc:153659; n=3; Danio rerio
55108	-4.32	0.13	0.0001	0.0088	0.62	all_v2.0.8631.C1	unclassified
43137	-3.30	0.19	0.0012	0.0268	0.62	all_v2.0.10071.C1	Uroporphyrinogen-III synthase (EC 4.2.1.75) (UROS) (Uroporphyrinogen- III cosynthetase) (Hydroxymethylbilane hydrolyase [cyclizing]) (UROIIIS).; n=1; Takifugu rubripes
47158	-5.84	0.09	0.0000	0.0032	0.62	sb_gmnbhkas_0007l19.pDNRF2	Unassigned protein
45506	-4.21	0.14	0.0002	0.0095	0.61	all_v2.0.9616.C1.a	non-directed
41116	-5.00	0.11	0.0000	0.0048	0.61	all_v2.0.2090.C1	PREDICTED: similar to transposase; n=1; Danio rerio
52630	-4.66	0.12	0.0001	0.0062	0.61	all_v2.0.8295.C1	unclassified
37904	-4.00	0.15	0.0002	0.0121	0.61	sb_gmnbbrts_0003k13.pDNRF2	Homolog of Gallus gallus Double-stranded RNA-activated protein kinase.; n=1; Takifugu rubripes
53017	-3.69	0.16	0.0005	0.0162	0.61	all_v2.0.3331.C1	unclassified
44336	-4.87	0.12	0.0000	0.0054	0.61	sb_gmapht_0037b08.pDNRF2	bloodthirsty [Notothenia coriiceps]
42784	-4.31	0.14	0.0001	0.0089	0.61	sb_gmnblits_0022m22.pDNRF2	Transposase; n=27; Anopheles gambiae
54819	-3.65	0.17	0.0005	0.0168	0.61	all_v2.0.14839.C1	unclassified
38095	-4.31	0.13	0.0001	0.0088	0.61	all_v2.0.6540.C1	Homolog of Homo sapiens Hemicentin; n=1; Takifugu rubripes
39187	-3.58	0.18	0.0006	0.0184	0.61	all_v2.0.6091.C2	Myc-regulated DEAD box protein; n=1; Oncorhynchus mykiss
42678	-3.71	0.16	0.0005	0.0160	0.61	all_v2.0.9799.C2	Transglutaminase; n=1; Oreochromis niloticus
43642	-4.65	0.12	0.0001	0.0062	0.61	all_v2.0.5071.C1	Zgc:152953; n=2; Danio rerio
45180	-3.52	0.18	0.0007	0.0197	0.61	sb_gmnblits_0013l06.pDNRm13r	LOC568642; similar to elongation of very long chain fatty acids (FEN1/Elo2, SUR4/Elo3, yeast)-like 4
52706	-4.24	0.15	0.0002	0.0092	0.60	all_v2.0.17371.C1	unclassified
35854	-3.72	0.17	0.0004	0.0160	0.60	all_v2.0.1645.C	Alkylated repair protein alkB homolog 5.; n=2;

						1	Gallus gallus
44579	-4.29	0.13	0.0001	0.0089	0.60	3MKK4_MAP2K3	custom - Mitogen-activated protein kinase kinase 4
47390	-4.52	0.13	0.0001	0.0072	0.60	sb_gmnlem_0005d01.t7	Unassigned protein
52084	-4.28	0.15	0.0001	0.0089	0.60	all_v2.0.4473.C1	unclassified
41628	-4.68	0.12	0.0001	0.0062	0.60	all_v2.0.10802.C1	Putative helicase MOV-10 (EC 3.6.1.-) (Moloney leukemia virus 10 protein).; n=1; Takifugu rubripes
53189	-3.36	0.20	0.0010	0.0244	0.60	all_v2.0.11205.C1	unclassified
48129	-3.47	0.18	0.0008	0.0212	0.60	all_v2.0.14003.C1	Unassigned protein
44665	-3.69	0.17	0.0005	0.0162	0.60	all_v2.0.13567.C1	Drf_FH1 multi-domain protein
40891	-4.85	0.12	0.0001	0.0054	0.60	all_v2.0.15259.C1	PREDICTED: similar to protein tyrosine phosphatase, receptor type, B; n=1; Ornithorhynchus anatinus
47135	-3.79	0.16	0.0004	0.0151	0.60	all_v2.0.1651.C1	Unassigned protein
42931	-6.05	0.09	0.0000	0.0032	0.60	sb_gmnbbr_0015n03.pDNRm13r	Ubiquitin-conjugating enzyme E2 L3; n=32; Euteleostomi
54087	-3.66	0.18	0.0005	0.0168	0.60	all_v2.0.4115.C1	unclassified
43485	-3.44	0.19	0.0008	0.0218	0.60	all_v2.0.4514.C4	Zgc:112050; n=2; Danio rerio
42929	-3.37	0.19	0.0010	0.0240	0.60	all_v2.0.473.C1	Ubiquitin-conjugating enzyme E2 D4; n=11; Euteleostomi
41997	-3.88	0.17	0.0003	0.0136	0.59	all_v2.0.10159.C1	S100-like protein; n=1; Monopterus albus
52910	-3.41	0.20	0.0009	0.0226	0.59	all_v2.0.999.C1	unclassified
41395	-3.92	0.16	0.0003	0.0130	0.59	all_v2.0.7668.C1	prospero homeobox 2; n=1; Takifugu rubripes
35894	-3.87	0.17	0.0003	0.0137	0.59	all_v2.0.16939.C1	Aminopeptidase N; n=2; Pseudopleuronectes americanus
54462	-3.95	0.16	0.0003	0.0128	0.59	all_v2.0.4285.C1	unclassified
38607	-3.53	0.19	0.0007	0.0197	0.59	all_v2.0.7062.C1	Interferon stimulated gene 15; n=1; Gadus morhua
50272	-3.68	0.20	0.0005	0.0165	0.58	all_v2.0.17016.C1	unclassified
39505	-3.55	0.20	0.0006	0.0189	0.58	sb_gmapov_0018l09.pDNRF2	Novel protein similar to vertebrate phosphoribosyl pyrophosphate amidotransferase; n=3; Danio rerio
45123	-3.38	0.20	0.0010	0.0236	0.58	all_v2.0.13960.C1	LOC501925; similar to solute carrier family 2, member 9 isoform a; K08146 MFS transporter, SP family, solute carrier family 2 (facilitated glucose transporter), member 9
43012	-5.06	0.12	0.0000	0.0047	0.58	all_v2.0.9212.C1	UPI0000D8BA1C related cluster; n=1; Danio rerio
47847	-3.66	0.19	0.0005	0.0168	0.57	all_v2.0.14003.C2	Unassigned protein
51325	-4.59	0.15	0.0001	0.0065	0.57	all_v2.0.1095.C1	unclassified
43109	-4.27	0.16	0.0001	0.0090	0.57	sb_gmnbpcic_0011h19.pDNRm13r	UPI00015A65AA related cluster; n=1; Danio rerio
47485	-3.40	0.21	0.0009	0.0232	0.57	all_v2.0.10276.C1	Unassigned protein
48810	-5.45	0.12	0.0000	0.0038	0.57	all_v2.0.7149.C2	Unassigned protein
38411	-3.72	0.19	0.0004	0.0160	0.57	sb_gmapht_0010p17.pDNRF2	Homolog of Homo sapiens TiTin isoform novex-2; n=1; Takifugu rubripes
52488	-3.41	0.22	0.0009	0.0226	0.57	all_v2.0.10916.C1	unclassified
42754	-5.50	0.12	0.0000	0.0037	0.57	sb_gmnbhkas_0008c11.pDNRF2	Transport-associated protein; n=3; Salmonidae

51353	-5.27	0.12	0.0000	0.0040	0.57	all_v2.0.10898.C1	unclassified
44517	-3.41	0.22	0.0009	0.0226	0.57	FAAD4c	custom - Fas (TNFRSF6)-associated via death domain
45143	-5.65	0.12	0.0000	0.0034	0.57	all_v2.0.8322.C2	LOC540789; similar to poly (ADP-ribose) polymerase family, member 14
52608	-3.68	0.18	0.0005	0.0165	0.57	all_v2.0.5157.C1	unclassified
37946	-3.30	0.23	0.0012	0.0268	0.56	all_v2.0.2234.C1	Homolog of Gallus gallus Voltage-dependent anion channel.; n=1; Takifugu rubripes
51681	-3.31	0.21	0.0011	0.0266	0.56	all_v2.0.9964.C1	unclassified
36339	-4.32	0.16	0.0001	0.0088	0.56	all_v2.0.7453.C3	Caspase-1; n=1; Dicentrarchus labrax
47050	-4.52	0.16	0.0001	0.0072	0.56	sb_gmapte_0012e14.pDNRF2	Unassigned protein
41920	-3.76	0.19	0.0004	0.0155	0.56	sb_gmapov_0012l04.pDNRF2	Ribulose-5-phosphate-3-epimerase; n=1; Danio rerio
36190	-3.58	0.21	0.0006	0.0183	0.56	sb_gmapht_0014i15.pDNRF2	Bloodthirsty; n=2; Notothenioidei
45146	-3.91	0.19	0.0003	0.0132	0.56	sb_gmnbhkic_0005i22.pDNRm13r	LOC555735; similar to DNA methyltransferase
47640	-5.21	0.13	0.0000	0.0042	0.56	sb_gmnbhkic_0015l18.pDNRm13r	Unassigned protein
38352	-4.29	0.18	0.0001	0.0089	0.56	all_v2.0.272.C2	Homolog of Homo sapiens SOUL protein; n=1; Takifugu rubripes
43192	-3.92	0.20	0.0003	0.0130	0.56	sb_gmnlbric_0006j07.t7	Vesicular glutamate transporter 2.2; n=3; Danio rerio
38788	-4.78	0.14	0.0001	0.0060	0.56	all_v2.0.1155.C2	Lily-type lectin; n=1; Platycephalus indicus
40847	-5.26	0.13	0.0000	0.0040	0.56	all_v2.0.8322.C1	PREDICTED: similar to Poly [ADP-ribose] polymerase 14 (PARP-14) (B aggressive lymphoma protein 2); n=1; Danio rerio
39325	-6.36	0.10	0.0000	0.0028	0.55	all_v2.0.9595.C2	NFX1-type zinc finger-containing protein 1.; n=1; Takifugu rubripes
36797	-4.72	0.15	0.0001	0.0062	0.55	all_v2.0.2166.C2	Cytotoxic and regulatory T cell protein; n=1; Oncorhynchus mykiss
37475	-3.35	0.22	0.0010	0.0246	0.55	all_v2.0.11071.C1	Gravin; n=2; Danio rerio
41492	-5.40	0.13	0.0000	0.0039	0.55	sb_gmnbgi_0004b13.pDNRF2	Protein MAK10 homolog; n=4; Clupeocephala
44545	-3.27	0.22	0.0012	0.0276	0.55	IRF1OK3	custom - Interferon regulatory factor 10K
54421	-4.98	0.15	0.0000	0.0050	0.55	all_v2.0.1129.C1	unclassified
38612	-3.64	0.22	0.0005	0.0170	0.55	all_v2.0.14.CB3	Interferon stimulated gene 15; n=1; Gadus morhua
38598	-3.97	0.20	0.0003	0.0125	0.55	all_v2.0.1580.C7	Interferon regulatory factor 1; n=1; Channa argus
38670	-5.14	0.14	0.0000	0.0043	0.55	all_v2.0.3891.C1	Isoform 2 of Q5RI56 ; n=2; Danio rerio
46586	-4.58	0.16	0.0001	0.0065	0.54	sb_gmnlem_0013j07.sp6	Unassigned protein
44546	-5.02	0.14	0.0000	0.0047	0.54	IRF1OK4	custom - Interferon regulatory factor 10K
40670	-3.88	0.21	0.0003	0.0136	0.54	all_v2.0.14446.C1	PREDICTED: similar to mitochondrial carrier triple repeat 6; n=1; Monodelphis domestica
45349	-4.21	0.18	0.0002	0.0095	0.54	all_v2.0.16273.C1	LOC793474; similar to stonustoxin alpha-subunit
45352	-3.28	0.24	0.0012	0.0274	0.54	sb_gmnbmd_0007i19.pDNRF2	LOC793817; similar to pol polyprotein
48304	-3.85	0.19	0.0003	0.0142	0.54	sb_gmnlla_0024h22.t7	Unassigned protein
45162	-4.39	0.18	0.0001	0.0085	0.54	all_v2.0.7368.C2	LOC561785; similar to very large inducible GTPase 1
36160	-3.72	0.23	0.0004	0.0160	0.54	all_v2.0.6011.C2	Beta-ureidopropionase (EC 3.5.1.6) (Beta-alanine synthase) (N- carbamoyl-beta-alanine amidohydrolase) (BUP-1).; n=1; Takifugu rubripes
44624	-4.49	0.18	0.0001	0.0074	0.53	Codviperin1	custom - viperin

50298	-5.18	0.14	0.0000	0.0042	0.53	all_v2.0.17232.C1	unclassified
54199	-3.99	0.19	0.0003	0.0122	0.53	all_v2.0.7185.C1	unclassified
46402	-4.40	0.19	0.0001	0.0084	0.53	sb_gmapht_0004b19.pDNRm13r	TTN; cardiac titin
47761	-8.34	0.08	0.0000	0.0026	0.53	all_v2.0.7777.C1	Unassigned protein
42860	-4.06	0.20	0.0002	0.0114	0.52	all_v2.0.7093.C1	Type II inositol-1,4,5-trisphosphate 5-phosphatase precursor (EC 3.1.3.36) (Phosphoinositide 5-phosphatase) (5PTase) (75 kDa inositol polyphosphate-5-phosphatase).; n=2; Gallus gallus
43939	-4.00	0.21	0.0002	0.0121	0.52	all_v2.0.2274.C1	Zgc:73351; n=1; Danio rerio
42223	-3.78	0.23	0.0004	0.0152	0.52	all_v2.0.3958.C1	Single-stranded DNA-binding protein, mitochondrial precursor (Mt-SSB) (MtSSB) (PWP1-interacting protein 17).; n=1; Takifugu rubripes
44458	-3.29	0.25	0.0012	0.0273	0.52	CK2c1	custom - CC chemokine
44371	-3.90	0.20	0.0003	0.0134	0.52	all_v2.0.2805.C1	caspc; caspase c
38614	-4.35	0.20	0.0001	0.0088	0.52	all_v2.0.7062.C2	Interferon stimulated gene 15; n=1; Gadus morhua
46051	-8.12	0.09	0.0000	0.0026	0.52	sb_gmnbhkic_0019j15.pDNRF2	PREDICTED: similar to Poly [ADP-ribose] polymerase 14 (PARP-14) (B aggressive lymphoma protein 2), partial [Danio rerio]
36525	-3.92	0.20	0.0003	0.0130	0.52	all_v2.0.16620.C1	Claudin 30; n=1; Oreochromis mossambicus
43476	-6.42	0.12	0.0000	0.0028	0.52	all_v2.0.11065.C1	Zgc:111879 protein; n=4; Danio rerio
48557	-3.99	0.20	0.0002	0.0121	0.52	sb_gmnbspic_0009m06.pDNRm13r	Unassigned protein
51629	-4.17	0.21	0.0002	0.0100	0.51	all_v2.0.11166.C1	unclassified
39658	-3.57	0.26	0.0006	0.0185	0.50	all_v2.0.5240.C2	Novel protein; n=3; Danio rerio
47164	-3.51	0.26	0.0007	0.0201	0.50	all_v2.0.6027.C6	Unassigned protein
41829	-6.58	0.12	0.0000	0.0028	0.50	sb_gmnblits_0018p08.pDNRF2	RFK protein; n=2; Bos taurus
51171	-3.36	0.31	0.0010	0.0244	0.50	all_v2.0.12475.C1	unclassified
46423	-3.71	0.23	0.0005	0.0161	0.50	all_v2.0.12194.C1	Unassigned protein
44123	-4.26	0.20	0.0002	0.0091	0.49	all_v2.0.8502.C1	Zinc finger CCHC domain-containing protein 9.; n=1; Takifugu rubripes
49575	-5.94	0.15	0.0000	0.0032	0.49	all_v2.0.3263.C1	unclassified
41014	-4.51	0.21	0.0001	0.0072	0.49	all_v2.0.14158.C1	PREDICTED: similar to Snf2-related CBP activator protein; n=1; Mus musculus
40679	-4.62	0.20	0.0001	0.0064	0.48	all_v2.0.4387.C1	PREDICTED: similar to mKIAA1931 protein; n=1; Monodelphis domestica
52069	-6.32	0.14	0.0000	0.0028	0.48	all_v2.0.6954.C2	unclassified
51371	-4.68	0.21	0.0001	0.0062	0.48	all_v2.0.5978.C1	unclassified
49723	-5.74	0.16	0.0000	0.0034	0.48	all_v2.0.4732.C1	unclassified
44587	-4.00	0.25	0.0002	0.0121	0.48	DXH3a	custom - probable ATP-dependent RNA helicase DHX
41315	-3.69	0.26	0.0005	0.0162	0.48	all_v2.0.5598.C2	Probable ATP-dependent helicase LGP2 (EC 3.6.1.-) (Protein D11Lgp2 homolog).; n=1; Takifugu rubripes
47806	-3.93	0.26	0.0003	0.0130	0.47	sb_gmnsul1_0001h03.pDNRF2	Unassigned protein
51636	-4.06	0.25	0.0002	0.0114	0.47	all_v2.0.1410.C1	unclassified

49008	-5.59	0.17	0.0000	0.0034	0.47	sb_gmnlla_0002h23.t7	Unassigned protein
41269	-4.31	0.25	0.0001	0.0088	0.46	sb_gmnlbfic_0004o20.t7	PREDICTED: similar to zinc finger, NFX1-type containing 1; n=3; Laurasiatheria
40397	-3.28	0.30	0.0012	0.0273	0.46	all_v2.0.6028.C2	PREDICTED: similar to deltex 3-like (Drosophila); n=1; Danio rerio
43596	-3.57	0.26	0.0006	0.0185	0.46	all_v2.0.8338.C1	Zgc:136229; n=4; Danio rerio
48537	-3.82	0.30	0.0004	0.0146	0.45	sb_gmnbhkas_0017n11.pDNRm13r	Unassigned protein
49317	-5.60	0.18	0.0000	0.0034	0.45	all_v2.0.13805.C1	unclassified
55051	-4.25	0.23	0.0002	0.0091	0.45	all_v2.0.9309.C1	unclassified
39077	-5.86	0.17	0.0000	0.0032	0.44	all_v2.0.431.C7	Microtubule aggregate protein homolog; n=1; Perca flavescens
47827	-4.89	0.22	0.0000	0.0053	0.44	sb_gmnbgi_0007a11.pDNRm13r	Unassigned protein
43557	-7.66	0.12	0.0000	0.0028	0.44	all_v2.0.2666.C5	Zgc:113527; n=4; Danio rerio
44543	-4.14	0.26	0.0002	0.0102	0.44	IRF1OK1	custom - Interferon regulatory factor 10K
44584	-3.47	0.35	0.0008	0.0212	0.44	DXH1a	custom - probable ATP-dependent RNA helicase DHX
38042	-3.50	0.36	0.0007	0.0206	0.44	all_v2.0.894.C5	Homolog of Homo sapiens Deoxyribonuclease gamma precursor; n=1; Takifugu rubripes
51818	-3.59	0.36	0.0006	0.0181	0.43	all_v2.0.11455.C1	unclassified
44859	-7.40	0.14	0.0000	0.0028	0.43	all_v2.0.314.C1	HECT domain containing protein
44448	-3.42	0.36	0.0009	0.0223	0.43	CK10ad	custom - CC chemokine
47049	-3.88	0.28	0.0003	0.0136	0.43	sb_gmnbhkas_0002l17.pDNRF2	Unassigned protein
52906	-5.82	0.18	0.0000	0.0032	0.42	all_v2.0.2709.C1	unclassified
50281	-3.73	0.33	0.0004	0.0159	0.42	all_v2.0.7444.C1	unclassified
41314	-3.74	0.35	0.0004	0.0157	0.41	all_v2.0.5598.C1	Probable ATP-dependent helicase LGP2 (EC 3.6.1.-) (Protein D11Lgp2 homolog).; n=1; Takifugu rubripes
43201	-4.10	0.29	0.0002	0.0108	0.41	all_v2.0.9330.C1	Viperin-like protein; n=1; Channa argus
35753	-4.28	0.28	0.0001	0.0090	0.41	sb_gmnlsras_0003g09.t7	Actin-binding vascular maintenance protein; n=5; Danio rerio
39328	-5.73	0.20	0.0000	0.0034	0.41	all_v2.0.2213.C1	NFX1-type zinc finger-containing protein 1; n=5; Eutheria
50467	-3.26	0.44	0.0013	0.0280	0.41	all_v2.0.1074.C1	unclassified
38611	-3.48	0.41	0.0008	0.0209	0.40	all_v2.0.1767.C1	Interferon stimulated gene 15; n=1; Gadus morhua
42028	-4.00	0.29	0.0002	0.0121	0.40	sb_gmnbhkic_0012l24.pDNRF2	Secernin 3; n=3; Tetraodontidae
40054	-5.82	0.20	0.0000	0.0032	0.40	all_v2.0.2666.C4	Poly [ADP-ribose] polymerase 15 (EC 2.4.2.30) (PARP-15) (B-aggressive lymphoma protein 3).; n=1; Takifugu rubripes
45351	-4.10	0.33	0.0002	0.0108	0.40	all_v2.0.1010.C8	LOC793474; similar to stonustoxin alpha-subunit
38606	-5.63	0.22	0.0000	0.0034	0.40	all_v2.0.14.CB4	Interferon stimulated gene 15; n=1; Gadus morhua
38603	-5.68	0.22	0.0000	0.0034	0.39	all_v2.0.14.CB7	Interferon stimulated gene 15; n=1; Gadus morhua
52407	-4.35	0.30	0.0001	0.0088	0.39	all_v2.0.4472.C1	unclassified
38605	-5.37	0.24	0.0000	0.0039	0.39	all_v2.0.14.C13	Interferon stimulated gene 15; n=1; Gadus morhua
38608	-6.15	0.20	0.0000	0.0028	0.38	all_v2.0.14.C12	Interferon stimulated gene 15; n=1; Gadus morhua
38609	-5.80	0.23	0.0000	0.0032	0.37	all_v2.0.14.C3	Interferon stimulated gene 15; n=1; Gadus morhua

47613	-5.59	0.23	0.0000	0.0034	0.37	sb_gmnbbr_0013m15.pDNRm13r	Unassigned protein
47376	-3.73	0.34	0.0004	0.0159	0.37	all_v2.0.15415.C1	Unassigned protein
41316	-3.45	0.47	0.0008	0.0217	0.37	all_v2.0.1009.C5	Probable ATP-dependent helicase LGP2 (EC 3.6.1.-) (Protein D11Lgp2 homolog).; n=1; Takifugu rubripes
55535	-4.75	0.29	0.0001	0.0062	0.37	all_v2.0.15359.C1	zp3a.1; zona pellucida glycoprotein 3a.1
44283	-4.35	0.29	0.0001	0.0088	0.35	sb_gmnlmfta_0004i04.t7	ARL6IP6; ADP-ribosylation-like factor 6 interacting protein 6
47410	-4.86	0.29	0.0000	0.0054	0.35	sb_gmnlsfic_0004h02.t7	Unassigned protein
49247	-5.48	0.26	0.0000	0.0038	0.35	all_v2.0.7226.C1	unclassified
44588	-4.23	0.38	0.0002	0.0093	0.34	DXH2b	custom - probable ATP-dependent RNA helicase DHX
41043	-4.03	0.41	0.0002	0.0119	0.34	all_v2.0.1010.C10	PREDICTED: similar to stonustoxin alpha-subunit; n=3; Danio rerio
40667	-6.31	0.23	0.0000	0.0028	0.33	all_v2.0.431.C8	PREDICTED: similar to microtubule aggregate protein homolog; n=2; Danio rerio
38604	-6.23	0.23	0.0000	0.0028	0.33	all_v2.0.14.CB2	Interferon stimulated gene 15; n=1; Gadus morhua
45583	-5.25	0.28	0.0000	0.0040	0.32	all_v2.0.2772.C15	non-directed
49585	-5.10	0.30	0.0000	0.0044	0.31	all_v2.0.115.C1	unclassified
42073	-6.66	0.23	0.0000	0.0028	0.31	all_v2.0.15254.C1	Serine hydrolase-like protein 2 (EC 3.1.-.-).; n=1; Takifugu rubripes
38675	-3.55	0.57	0.0007	0.0190	0.29	all_v2.0.9761.C1	Isoform S of Q76IC6 ; n=1; Rattus norvegicus
47462	-4.31	0.44	0.0001	0.0088	0.29	sb_gmnbhkas_0017n11.pDNRF2	Unassigned protein
38651	-5.12	0.32	0.0000	0.0044	0.29	sb_gmapht_0023a03.pDNRF2	Intraflagellar transport protein 52; n=4; Danio rerio
47445	-3.67	0.46	0.0005	0.0165	0.27	sb_gmnbmd_0004h11.t7	Unassigned protein
38613	-4.13	0.41	0.0002	0.0104	0.26	all_v2.0.14.CB5	Interferon stimulated gene 15; n=1; Gadus morhua
44180	-6.24	0.35	0.0000	0.0028	0.20	all_v2.0.1266.C1	ZPC4; n=2; Oryzias latipes
38638	-5.94	0.42	0.0000	0.0032	0.18	all_v2.0.638.C1	Interleukin-8 variant 2; n=3; Ictalurus punctatus
38639	-7.49	0.33	0.0000	0.0028	0.16	all_v2.0.638.CB1	Interleukin-8 variant 2; n=3; Ictalurus punctatus
45361	-6.47	0.39	0.0000	0.0028	0.15	all_v2.0.34.C2	LOC797694; similar to solute carrier family 25 member 5 protein

[a]Fold-changes are presented as outputted by siggenes and were calculated as 16°C/10°C

Supplemental Table S9. 96 genes reproducibly significantly differentially expressed between fish injected with pIC at 10°C and 16°C and sampled at 6HPI and fish injected with pIC at 10°C and 16°C and sampled at 24HPI

Probe ID	Gene Name	Fold-Change[a] 6HPI	Fold-Change[a] 24HPI
35894	Aminopeptidase N; n=2; Pseudopleuronectes americanus	1.43	0.59
44283	ARL6IP6; ADP-ribosylation-like factor 6 interacting protein 6	2.10	0.35
44336	bloodthirsty [Notothenia coriiceps]	2.44	0.61
36193	Bloodthirsty; n=2; Notothenioidei	1.67	0.62
36190	Bloodthirsty; n=2; Notothenioidei	2.05	0.56
36195	Bloodthirsty; n=2; Notothenioidei	2.11	0.64
36185	Bloodthirsty; n=2; Notothenioidei	2.01	0.68
36186	Bloodthirsty; n=2; Notothenioidei	2.17	0.62
36317	Carnitine palmitoyltransferase II; n=2; Danio rerio	1.92	0.66
44375	cct5; chaperonin containing TCP1, subunit 5 (epsilon); K09497 T-complex protein 1 subunit epsilon	2.44	0.64
36525	Claudin 30; n=1; Oreochromis mossambicus	3.53	0.52
44448	custom - CC chemokine	7.29	0.43
44544	custom - Interferon regulatory factor 10K	1.81	0.64
44547	custom - Interferon regulatory factor 10K	1.70	0.71
44546	custom - Interferon regulatory factor 10K	2.48	0.54
44543	custom - Interferon regulatory factor 10K	2.32	0.44
44550	custom - interleukin-1 receptor-associated kinase 4	1.57	0.73
44579	custom - Mitogen-activated protein kinase kinase 4	1.90	0.60
44588	custom - probable ATP-dependent RNA helicase DHX	3.73	0.34
44584	custom - probable ATP-dependent RNA helicase DHX	3.55	0.44
44587	custom - probable ATP-dependent RNA helicase DHX	2.04	0.48
44624	custom - viperin	3.03	0.53
36901	DNA-directed RNA polymerase I subunit RPA49 (RNA polymerase I-associated factor 53) (RNA polymerase I polypeptide E) (RNA polymerase I-associated factor 1) (A49).; n=1; Takifugu rubripes	1.32	0.68
44663	Drf_FH1 multi-domain protein	1.58	0.73
37498	GTP-binding nuclear protein Ran; n=35; Euteleostomi	1.36	0.65
44859	HECT domain containing protein	5.92	0.43
37698	Homolog of Brachydanio rerio Cyclin L ania-6a.; n=2; Takifugu rubripes	1.70	0.67
37707	Homolog of Brachydanio rerio Fem1c.; n=1; Takifugu rubripes	1.62	0.70
37904	Homolog of Gallus gallus Double-stranded RNA-activated protein kinase.; n=1; Takifugu rubripes	2.52	0.61
38042	Homolog of Homo sapiens Deoxyribonuclease gamma precursor; n=1; Takifugu rubripes	2.18	0.44
38160	Homolog of Homo sapiens Membrane interacting protein of RGS16; n=1; Takifugu rubripes	1.71	0.66
38352	Homolog of Homo sapiens SOUL protein; n=1; Takifugu rubripes	2.34	0.56
38598	Interferon regulatory factor 1; n=1; Channa argus	2.43	0.55
38611	Interferon stimulated gene 15; n=1; Gadus morhua	5.90	0.40
38612	Interferon stimulated gene 15; n=1; Gadus morhua	3.76	0.55
38604	Interferon stimulated gene 15; n=1; Gadus morhua	5.62	0.33
38609	Interferon stimulated gene 15; n=1; Gadus morhua	4.67	0.37
38606	Interferon stimulated gene 15; n=1; Gadus morhua	5.20	0.40
38614	Interferon stimulated gene 15; n=1; Gadus morhua	3.81	0.52
38613	Interferon stimulated gene 15; n=1; Gadus morhua	8.45	0.26
38607	Interferon stimulated gene 15; n=1; Gadus morhua	2.10	0.59

38608	Interferon stimulated gene 15; n=1; Gadus morhua	4.80	0.38
38605	Interferon stimulated gene 15; n=1; Gadus morhua	5.58	0.39
38603	Interferon stimulated gene 15; n=1; Gadus morhua	3.77	0.39
38638	Interleukin-8 variant 2; n=3; Ictalurus punctatus	7.90	0.18
38639	Interleukin-8 variant 2; n=3; Ictalurus punctatus	5.88	0.16
38651	Intraflagellar transport protein 52; n=4; Danio rerio	6.19	0.29
38670	Isoform 2 of Q5RI56 ; n=2; Danio rerio	2.00	0.55
45123	LOC501925; similar to solute carrier family 2, member 9 isoform a; K08146 MFS transporter, SP family, solute carrier family 2 (facilitated glucose transporter), member 9	1.62	0.58
45143	LOC540789; similar to poly (ADP-ribose) polymerase family, member 14	1.58	0.57
45146	LOC555735; similar to DNA methyltransferase	1.62	0.56
45361	LOC797694; similar to solute carrier family 25 member 5 protein	3.78	0.15
39328	NFX1-type zinc finger-containing protein 1; n=5; Eutheria	2.67	0.41
39323	NFX1-type zinc finger-containing protein 1.; n=1; Takifugu rubripes	1.36	0.74
39325	NFX1-type zinc finger-containing protein 1.; n=1; Takifugu rubripes	1.96	0.55
39505	Novel protein similar to vertebrate phosphoribosyl pyrophosphate amidotransferase; n=3; Danio rerio	1.68	0.58
39626	Novel protein; n=2; Danio rerio	1.60	0.68
40263	PREDICTED: similar to bloodthirsty; n=3; Danio rerio	1.70	0.72
40670	PREDICTED: similar to mitochondrial carrier triple repeat 6; n=1; Monodelphis domestica	1.93	0.54
41014	PREDICTED: similar to Snf2-related CBP activator protein; n=1; Mus musculus	2.08	0.49
41103	PREDICTED: similar to transposase, partial; n=1; Strongylocentrotus purpuratus	2.04	0.68
41269	PREDICTED: similar to zinc finger, NFX1-type containing 1; n=3; Laurasiatheria	5.56	0.46
41314	Probable ATP-dependent helicase LGP2 (EC 3.6.1.-) (Protein D11Lgp2 homolog).; n=1; Takifugu rubripes	3.77	0.41
41316	Probable ATP-dependent helicase LGP2 (EC 3.6.1.-) (Protein D11Lgp2 homolog).; n=1; Takifugu rubripes	5.61	0.37
41829	RFK protein; n=2; Bos taurus	2.77	0.50
42028	Secernin 3; n=3; Tetraodontidae	1.44	0.40
42073	Serine hydrolase-like protein 2 (EC 3.1.-.-).; n=1; Takifugu rubripes	2.93	0.31
42375	SRY-box containing gene 11a; n=1; Gnathonemus petersii	1.47	0.76
42860	Type II inositol-1,4,5-trisphosphate 5-phosphatase precursor (EC 3.1.3.36) (Phosphoinositide 5-phosphatase) (5PTase) (75 kDa inositol polyphosphate-5-phosphatase).; n=2; Gallus gallus	1.56	0.52
48258	Unassigned protein	2.72	0.64
47368	Unassigned protein	1.57	0.72
47613	Unassigned protein	1.76	0.37
46586	Unassigned protein	2.02	0.54
47806	Unassigned protein	2.02	0.47
47164	Unassigned protein	6.72	0.50
47445	Unassigned protein	1.59	0.27
54421	unclassified	2.04	0.55
53852	unclassified	2.13	0.64
52069	unclassified	3.13	0.48
49575	unclassified	2.52	0.49
49723	unclassified	1.60	0.48
51353	unclassified	1.89	0.57
50298	unclassified	1.45	0.53
51171	unclassified	2.32	0.50
51636	unclassified	2.19	0.47

52606	unclassified	1.71	0.69
52488	unclassified	1.80	0.57
55314	unclassified	1.98	0.68
52706	unclassified	1.88	0.60
43012	UPI0000D8BA1C related cluster; n=1; Danio rerio	1.84	0.58
43192	Vesicular glutamate transporter 2.2; n=3; Danio rerio	2.15	0.56
43197	VHSV-induced protein; n=1; Oncorhynchus mykiss	3.13	0.64
43201	Viperin-like protein; n=1; Channa argus	5.43	0.41
43642	Zgc:152953; n=2; Danio rerio	1.84	0.61
44015	Zgc:86905; n=1; Danio rerio	1.52	0.66
44180	ZPC4; n=2; Oryzias latipes	5.24	0.20

[a]Fold-changes are presented as outputted by siggenes and were calculated as 16°C/10°C
