# Supplementary material for: A moderate increase in ambient temperature modulates the Atlantic cod (Gadus morhua) spleen transcriptome response to intraperitoneal viral mimic injection
Source: BMC Genomics. 2012 Aug 28;13:431. doi: 10.1186/1471-2164-13-431 (PMC3534563; doi:10.1186/1471-2164-13-431)
Supplement: Additional file 2 — Figure S1. Putative type I interferon activation via Toll-like receptors (TLRs) in Atlantic cod. [file 1471-2164-13-431-S2.pdf]

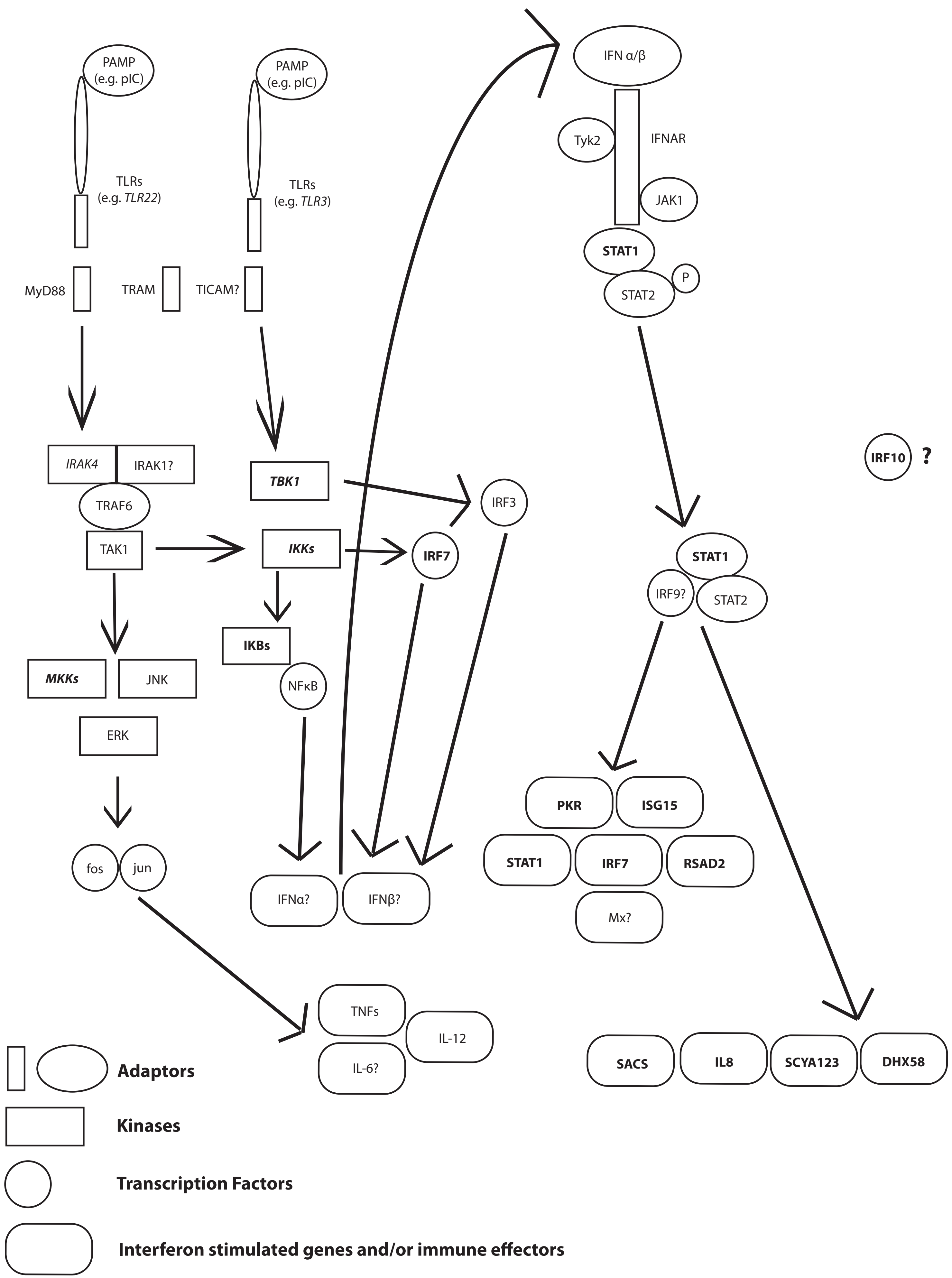

PAMP
(e.g. pIC)
PAMP
(e.g. pIC)
IFN α/β
Tyk2
IFNAR
TLRs
(e.g. TLR22)
TLRs
(e.g. TLR3)
JAK1
STAT1
P
STAT2
MyD88
TRAM
TICAM?
IRF10
?
IRAK4
IRAK1?
TBK1
TRAF6
IRF3
TAK1
IKKs
IRF7
STAT1
IRF9?
STAT2
IKBs
MKKs
JNK
NFκB
ERK
PKR
ISG15
STAT1
IRF7
RSAD2
fos
jun
IFNα?
IFNβ?
Mx?
TNFs
IL-12
IL-6?
SACS
IL8
SCYA123
DHX58
Adaptors
Kinases
Transcription Factors
Interferon stimulated genes and/or immune effectors

Supplemental Figure 1 - Putative type I interferon activation via Toll-like receptors (TLRs) in Atlantic cod. Figure adapted from Rebl et al. [41] and Noppert et al. [54]. The orthology between same-named Atlantic cod and mammalian genes must be validated, and all gene interactions (represented by arrows) are based on the mammalian literature and must be confirmed by experimental research for Atlantic cod. Genes in bold were identified as significantly differentially expressed in at least one microarray comparison and confirmed by QPCR; genes in bold-italics were identified as significantly differentially expressed in at least one microarray comparison but were not subjected to QPCR; genes in italics were identified as significantly differentially expressed in at least one microarray comparison and were subjected but not confirmed by QPCR. A question mark beside a gene indicates that it could not be found by nominal search in either the Ensembl annotation of the Atlantic Cod genome (http://ensembl.org/Gadus_morhua) or the Atlantic Cod Genomics and Broodstock Development Project EST database (http://www.codgene.ca). The bold question mark indicates genes found in Atlantic Cod with no known human orthologs. PAMP = pathogen associated molecular pattern; pIC = polyriboinosinic polyribocytidylic acid; TRL = Toll-like receptor; MyD88 = Myeloid differentiation primary response gene (88); TRAM = TRIF-related adaptor molecule; TICAM = TIR domain-containing adaptor molecule; IRAK = Interleukin-1 receptor associated kinase; TAK1 = TGF-beta activated kinase 1; TRAF6 = TNF receptor-asspciated factor 6, E3 ubiquitin protein ligase; TBK1 = TANK-binding kinase 1; IKK = Inhibitor of NFκB kinases; IRF = Interferon regulatory factor; IKB = Inhibitor of NFκB; NFκB = Nuclear factor of kappa gene enhancer in B cells; MKK = Mitogen activated protein kinase; ERK = Extracellular signal-regulated kinase; JNK = JUN n-terminal kinase; IFN = Interferon; fos = FBJ murine osteosarcoma viral oncogene homolog; jun = JUN proto-oncogene; IFNAR = Interferon (alpha, beta and omega) receptor; Tyk2 = Tyrosine kinase 2; JAK1 = Janus kinase 1 ; STAT = Signal transducer and activator of transcription; PKR = Double-stranded RNA activate protein kinase; ISG = Interferon stimulated gene; RSAD2 = Radical-S-adenosyl containing methionine domain containing 2; Mx = Myxovirus (influenza virus) resistance, interferon-inducible protein; SACS = Sacsin; IL = Interleukin; SCYA = Small-inducible chemokine; DHX58 = DEXH (Asp-Glu-X-His) box polypeptide 58; TNF = Tumor necrosis factor.
